# Synthesis of new asparagine-based glycopeptides for future scanning tunneling microscopy investigations

**DOI:** 10.3762/bjoc.16.80

**Published:** 2020-04-30

**Authors:** Laura Sršan, Thomas Ziegler

**Affiliations:** 1 Institute of Organic Chemistry, University of Tübingen, Auf der Morgenstelle 18, 72076 Tübingen, Germany

**Keywords:** amino acids, asparagine, carbohydrates, glycopeptides, peptidomimetics

## Abstract

For investigations on the biological functions of oligosaccharides and peptidomimetics, new asparagine-based mono- and disaccharides containing glycopeptides were prepared in solution. The applicability of two common peptide coupling reagents, using an orthogonal Fmoc/*t*-Bu strategy along with acetyl protecting groups for the carbohydrate moiety, was studied. Thus, the prepared libraries of glycopeptides were designed as model systems of cell surfaces for future investigations by combined preparative mass spectroscopy and scanning tunneling microscopy (STM) using soft-landing electrospray beam deposition (ES-IBD), on metal surfaces.

## Introduction

Glycopeptides are generally found on virtually every eukaryotic and prokaryotic cell surface. So far, the study of glycopeptide interactions with other proteins gave some insight into important biological functions, for instance, intracellular communication, cell–cell recognition, immune response, and pathogenesis [1–4]. Glycosylation is also considered to be one of the most important post-translational modification (PTM) since more than half of all human proteins are glycopeptides or glycoproteins [5]. Therefore, understanding how glycopeptides interact on an intra- and intermolecular level is highly important for resolving some of the most challenging topics in medicinal chemistry [4,6–7]. Due to the microheterogeneity of naturally occurring glycopeptides, which is the reason for the hampered isolation of these structures in a pure form from biological material, there is a great interest in the synthesis of structurally defined peptidomimetic libraries for further biological and medicinal investigation [4,8]. Recently, the characterization of heterogeneous mixtures of glycoconjugates was simplified by the development of novel MS-supported methods [9–11]. Nevertheless, the complexity of the aforementioned macromolecules caused by PTM still constitutes a technical challenge. They can only be applied to small amounts of pure material, which is insufficient for extended biological investigations [4,6]. Hitherto, a variety of glycopeptides are used for medicinal applications, for example, in anti-HIV therapy, MUC1-based antitumor vaccines, or as antibiotics [12–14]. Especially glycans bearing noncanonical amino acids, which can only be introduced into a peptide by organic synthesis, are suitable for cancer therapy since they show better resistance to enzymatic degradation in comparison with naturally occurring amino acids [14]. Thus, there is still a great effort in finding new potential drugs derived from glycopeptides [4,15].

By using preparative mass spectrometry (pMS) combined with STM on submolecular-resolution peptides and carbohydrates can be investigated regarding their self-assembly on metal surfaces [16–17]. For such novel MS applications, a new technology, i.e., ES-IBD, has been developed. ES-IBD furthermore allows for the integrity of the material [18]. The application of these new techniques to synthetic glycopeptides is expected to provide new insight into the mechanisms of the interaction of glycopeptides with the cell surface [16].

Our focus lay on the preparation of glycopeptide libraries containing ʟ-asparagine since many saccharides on the cell surface are *N*-glycosidically linked to this amino acid [8]. The most abundant sugars found in these asparagine-linked structures are *N*-acetylglycosylamines, glucose, galactose, mannose, cellobiose, lactose, and maltose [19]. Therefore, we intended to prepare glycopeptide structures containing these sugars in a stepwise way, up to tripeptides. An orthogonal Fmoc/*t*-Bu protecting group strategy was chosen along with the frequently used onium salts HBTU and HATU as coupling reagents [20]. The canonical amino acids ʟ-phenylalanine, ʟ-tryptophane, and ʟ-alanine were chosen in order to allow for different side chain motifs that, in turn, would increase the suitability for the following investigation via STM. Due to the aromatic backbone and known interaction of the compounds with Cu(100) and Au(111), ʟ-Phe and ʟ-Trp should be distinguishable in STM from the remaining building blocks [16,18,21]. ʟ-Ala seemed to be an ideal supplement for the synthesis of these new glycoconjugates since there was no additional functional group that would require an additional protection orthogonal to the Fmoc/*t*-Bu protocol and the acetyl groups of the sugar moieties. However, numerous other natural or unnatural amino acids, functionalized or not, could be used for this reaction protocol.

## Results and Discussion

**Scheme 1:**
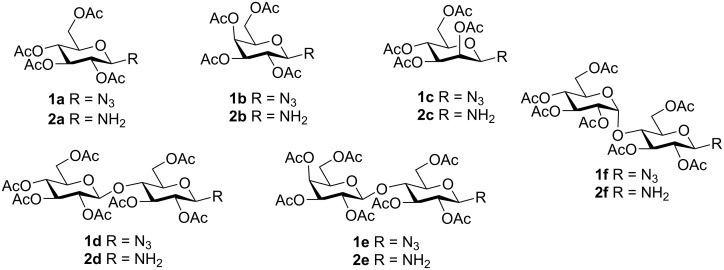
Description of the starting materials **1a–f** and **2a–f**.

For the synthesis of the anticipated glycopeptides, we started from the respective fully acetylated β-ᴅ-glycosyl azides **1a**–**f** in the gluco, galacto, manno, cello, lacto, and malto series (Scheme 1). These glycosyl azides were prepared from the corresponding halogenoses by the treatment with 1.2 equivalents of sodium azide in aqueous acetone according to literature procedures [22–26]. In the case of 2,3,4,6-tetra-*O*-acetyl-α-ᴅ-mannopyranosyl bromide, however, this procedure only resulted in a complete hydrolysis of the halogenose. Under optimized reaction conditions using Moyle’s procedure (2.1 equivalents NaN_3_ in DMF) [23], the azide **1c** could be obtained in 33% yield (compared to the reported 13%). Attempts to prepare the corresponding α-ᴅ-mannosyl azide under various previously described conditions [27–28] failed in our hands though.

Next, the glycosyl azides **1a**–**f** were converted into the corresponding glycosylamines **2a**–**f** by hydrogenation with Pd on charcoal in ethyl acetate. Since an anomeric mixture of glycosylamines was obtained in most cases, with a strong predominance of the corresponding β-anomers, and TLC analysis showed no formation of other unwanted side products during hydrogenation, the latter amines were used for the next step without further purification. In all cases, only the β-anomeric glycosylpeptides were obtained from the anomeric mixtures of amines (see below).

**Scheme 2:**
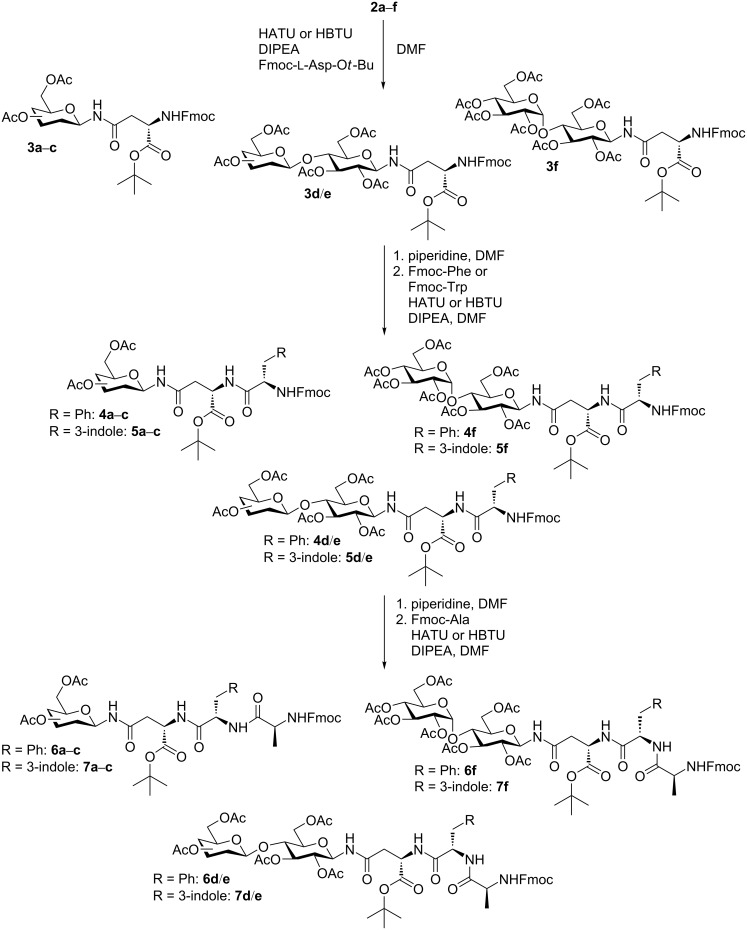
Peptide coupling reactions, including the previous Fmoc cleavage.

The glycosylated aspartic acids **3** (except for **3d** and **3f**) were prepared previously with the help of different types of coupling reagents, such as benzotriazol-1-yloxytris(dimethylamino)phosphonium hexafluorophosphate (BOP) or diisopropyl carbodiimide (DIC) and additives, such as 1-hydroxybenzotriazole (HOBt) or triethylphosphine. [29–31] However, in our hands, none of the described methods reached the high yields that we previously obtained with the onium salt-mediated peptide coupling protocol with *O*-(7-azabenzotriazol-1-yl)-*N*,*N*,*N*′,*N*′-tetramethyluronium hexafluorophosphate (HATU) or with 2-(1*H*-benzotriazol-1-yl)-1,1,3,3-tetramethyluronium hexafluorophosphate (HBTU, Scheme 2 and Table 1) [6,32]. Here, we compared the condensation with HBTU and HATU for the preparation of the glycosyl amino acids **3a**–**f** since previous studies showed HATU to be more efficient than HBTU in similar cases [33–35]. Table 1 summarizes the obtained yields for conversions **2**→**3**, **3**→**4**, **3**→**5**, **4**→**6**, and **5**→**7** (Scheme 2). As can be seen in Table 1, the yields for all condensations with HBTU and HATU are in a comparable range. Only for the cellobiosyl Asp amino acid **3f** (Table 1, entry 6), the lactosylated Asp–Phe–Ala tripeptide **6e** (Table 1, entry 23), and the galactosylated Asp–Trp dipeptide **5b** (Table 1, entry 14), HATU gave a significantly higher yield than HBTU.

**Table 1:** Yields of the peptide coupling reactions and comparison of HBTU and HATU.

entry	compound	HBTU	HATU		entry	compound	HBTU	HATU

1	**3a**	91%	82%		19	**6a**	82%	75%
2	**3b**	92%	84%		20	**6b**	76%	72%
3	**3c**	77%	80%		21	**6c**	64%	60%
4	**3d**	61%	79%		22	**6d**	75%	59%
5	**3e**	63%	61%		23	**6e**	50%	70%
6	**3f**	56%	86%		24	**6f**	50%	78%

7	**4a**	81%	76%		25	**7a**	60%	68%
8	**4b**	67%	73%		26	**7b**	76%	79%
9	**4c**	72%	80%		27	**7c**	76%	58%
10	**4d**	68%	80%		28	**7d**	60%	68%
11	**4e**	76%	79%		29	**7e**	75%	65%
12	**4f**	78%	67%		30	**7f**	58%	50%

13	**5a**	53%	66%					
14	**5b**	52%	75%					
15	**5c**	89%	62%					
16	**5d**	38%	59%					
17	**5e**	78%	66%					
18	**5f**	59%	73%					

The Fmoc cleavage of the compounds **3**–**5** was performed with piperidine in DMF (20 vol %) [36]. The reaction products were not isolated and purified but instead immediately used for the succeeding peptide coupling step to give the dipeptides **4** and **5** from the glycosylated asparagines **3**, and the tripeptides **6** and **7** from **4** and **5**, respectively (Scheme 2). The yields of the glycosylated di- and tripeptides were moderate to good (Table 1, entries 7–30). For the coupling of Fmoc-Trp instead of Fmoc-Phe, an improved yield could be observed when using HATU instead of HBTU. Although it is well known that Fmoc-protected tryptophane can lead to unwanted side products when the NH group in the indole ring remains unprotected, we decided to refrain from additional protection of the indole moiety because the removal of that protecting group would have reduced the overall yield in the end.

**Scheme 3:**
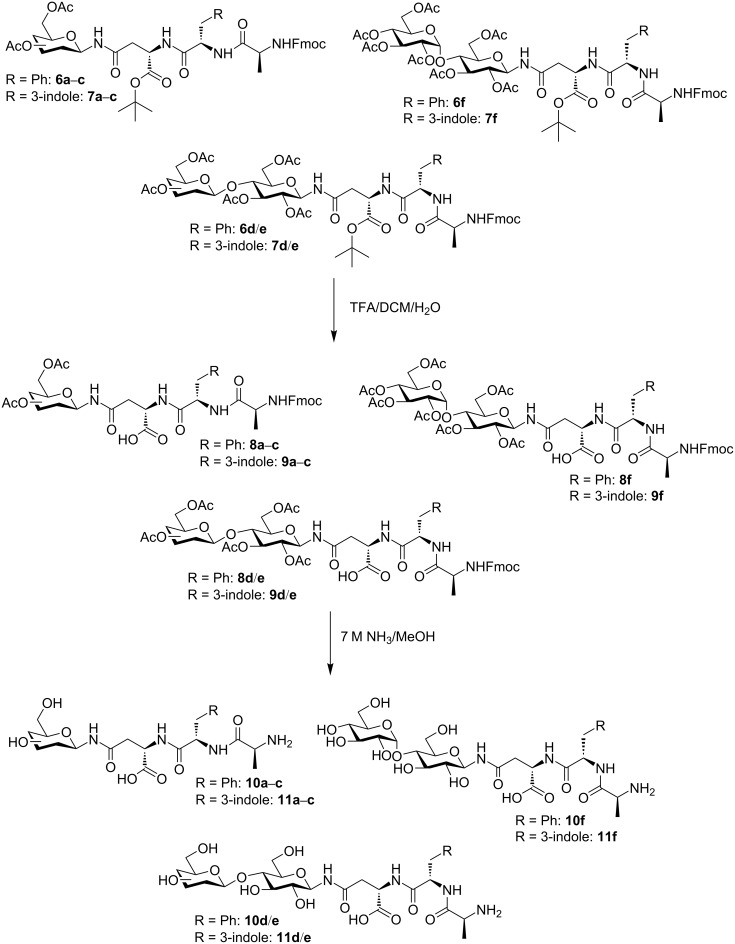
Cleavage of the fully protected peptides **6** and **7**.

The treatment of the glycosylated tripeptides **6a**–**f** and **7a**–**f** with a mixture of TFA, DCM, and H_2_O (10:10:1) [31] afforded the partially deprotected acids **8a**–**f** and **9a**–**f** in quantitative yields. The final deprotection of both the base-labile acetyl and Fmoc-protecting groups was achieved by the treatment with 7 M NH_3_ in MeOH, which afforded the free glycopeptides **10a**–**f** and **11a**–**f** in good to excellent yields (Scheme 3 and Table 2). Since chromatographic purification of the unprotected glycopeptides on silica gel would have been rather difficult due to their zwitterionic character, our simple deprotection strategy with NH_3_ in MeOH allowed the crude product to precipitate directly from the reaction mixture, followed by the isolation through simple filtration. After washing the residue with petroleum ether, the compounds **10** and **11** were obtained in pure form.

**Table 2:** Final deprotection yields of **10a**–**f** and **11a**–**f**.

entry	compound	yield

1	**10a**	98%
2	**10b**	96%
3	**10c**	66%
4	**10d**	61%
5	**10e**	80%
6	**10f**	99%
7	**11a**	92%
8	**11b**	99%
9	**11c**	77%
10	**11d**	49%
11	**11e**	99%
12	**11f**	87%

## Conclusion

We described the efficient chemical synthesis of a series of new glycopeptides containing asparagine, tryptophane, alanine, and phenylalanine linked to various saccharides, such as glucose, galactose, mannose, cellobiose, lactose, and maltose. We further compared two common peptide coupling procedures that use HBTU and HATU, respectively. We showed that the use of HATU reduced the reaction times but did not always result in higher yields, as anticipated from the literature. A simple cleavage protocol that did not necessitate any further purification step allowed for the preparation of the unprotected glycopeptides in solution. The investigation of the self assembly of such glycopeptides on metal surfaces will be performed via pMS and STM in further studies.

## Supporting Information

File 1 General methods, experimental procedures, and product characterization data of the compounds **1a**–**f** to **11a**–**f**.

File 2 NMR spectra of the compounds **1a**–**f** to **11a**–**f**.
