# Supplementary material for: Synthesis of new asparagine-based glycopeptides for future scanning tunneling microscopy investigations
Source: Beilstein J Org Chem. 2020 Apr 30;16:888–94. doi: 10.3762/bjoc.16.80 (PMC7214877; doi:10.3762/bjoc.16.80)
Supplement: File 1 — General methods, experimental procedures, and product characterization data of the compounds 1a–f to 11a–f. [file Beilstein_J_Org_Chem-16-888-s001.pdf]

BEILSTEIN JOURNAL OF ORGANIC CHEMISTRY

Supporting Information

for

Synthesis of new asparagine-based glycopeptides for future scanning tunneling microscopy investigations

Laura Sršan and Thomas Ziegler

Beilstein J. Org. Chem. **2020,** *16,* 888–894. doi:10.3762/bjoc.16.80

General methods, experimental procedures, and product characterization data of the compounds 1a–f to 11a–f

License and Terms: This is a supporting information file under the terms of the Creative Commons Attribution License (http://creativecommons.org/licenses/by/4.0). Please note that the reuse, redistribution and reproduction in particular requires that the authors and source are credited.
The license is subject to the *Beilstein Journal of Organic Chemistry* terms and conditions: (https://www.beilstein-journals.org/bjoc)

General Information

All solvents were dried and/or distilled prior to use. The reaction progress was monitored by TLC on Polygram Sil G/UV silica gel plates from Machery & Nagel. The visualization was performed by charring with H_2SO_4 (5% in EtOH), molybdenum blue, or by using UV light. Flash column chromatography was performed on silica gel (0.032–0.063 mm) from Machery & Nagel, and a HD 2-400 pump from Besta with a Dynamax UV-1 absorbance detector was used. NMR spectra were recorded on a Bruker Avance III HD 400, a Bruker Avance III HD 300 NanoBay, a Bruker Avance III HDX 600, or a Bruker Avance III HDX 700. Chemical shifts δ are given in ppm and coupling constants in Hz. The residual proton signal of the deuterated solvent was used as the internal standard. High-resolution mass spectra were recorded on a Bruker Daltonics maxis 4G. Elemental analyses were performed on HEKA-tech Euro EA 3000. Melting points were determined on a Melting Point M-560 from Büchi and are uncorrected. Optical rotations were measured with a Perkin-Elmer Polarimeter 341.

Due to the polar structure and free functional groups of **8a**–**f** to **11a**–**f** where hydrogen bonds can occur, we disclaimed elemental analyses since they would be unrepresentative.

Experimental data

General Procedure A for the synthesis of 1a/b/d/e/f

To a solution of the glycosyl halide (1.0 equiv) in acetone (4 mL per 1.0 mmol), NaN_3 (1.2 equiv) dissolved in water (1 mL per 1.0 mmol) was added, and this was stirred for 16 h at room temperature. After the solvent was evaporated, the residue was taken up in DCM and washed with water (3 × 20 mL). The combined organic phases were dried over Na_2SO_4 and reduced in vacuo. The crude product was recrystallized from EtOH and obtained as a colorless, crystalline solid. The analytical characterization was done according to the literature [1-8].

General Procedure B for the synthesis of 2a–f

1a–f (1.0 equiv), respectively, and Pd (10 wt % on charcoal) were suspended in ethyl acetate (10 mL per 1.0 mmol), and this was stirred under a H_2 atmosphere. After the complete conversion of the starting material, as monitored by TLC anlysis (1 to 24 h), the crude mixture was filtered over celite and washed with ethyl acetate. After evaporation of the solvent, the colorless solid was used without further purification. The analytical characterization of **2a**/**b**/**d**/**e** was according to literature and/or has been fully completed [1,9-12].

General Procedure C for the synthesis of 3a–f

Under a N_2 atmosphere, a solution of HBTU (Procedure C1) or HATU (Procedure C2, 1.1 equiv) and the free amino acid (1.1 equiv) in dry DMF (5 mL per 1.0 mmol) were added to an icecold solution of the free amine in dry DMF (3 mL per 1.0 mmol), followed by the addition of dry DIPEA (1.3 equiv). The reaction mixture was allowed to warm to room temperature. After the complete conversion (HBTU: 15 to 18 h, HATU: 1 to 2 h), as monitored by TLC

analysis, the solvent was evaporated in vacuo. The residue was taken up in ethyl acetate and washed with aq citric acid (10%), aq $NaHCO_3$ solution, and brine (each 3 × 20 mL). The combined organic layers were dried over Na_2SO_4 and concentrated in vacuo. The crude product was purified by column chromatography (PE/EE 2:3→1:2) to give a colorless or yellowish solid. The analytical characterization of **3a**–**c**/**e** was done according to the literature [13].

General Procedure D for the synthesis of 4a–f to **7a–f**

After 10 minutes of stirring the protected peptide in piperidine/DMF (20 vol %, 15 mL per 1.0 mmol, the progress was monitored by TLC analysis), the solvent was evaporated in vacuo and the crude product was used without further purification. After at least 1 h of drying in a high vacuum, the General Procedure for **7** (HBTU: Procedure D1; HATU: Procedure D2) was repeated.

General Procedure E for the synthesis of 8a–f to **9a–f**

The peptide was stirred in a solution of DCM, TFA, and water (10:10:1). After the full conversion (TLC), the solvent was removed in a flow of N_2. The residue was dissolved in a mixture of acetonitrile/water (1:1) and lyophilized. The product was isolated as a fluffy white, yellow, or red solid.

General Procedure F for the synthesis of 10a–f to **11a–f**

The unprotected acid was stirred for approximately 65 h in a 7 M NH_3 solution in MeOH (5 mL per 1.0 mmol). The precipitated solid was filtered off and washed with petroleum ether. Where no precipitation occurred, the solvent was evaporated. The product was then isolated by

trituating the residue with petroleum ether and extraction with water. After lyophilization of the aqueous solution (with added acetonitrile in case of a low solubility), the fully deprotected glycopeptide was obtained as a white to yellowish solid.

2,3,4,6-Tetra-*O*-acetyl-β-D-mannopyranosyl azide (1c)[14]

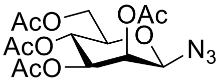

Under a N_2 atmosphere, 2,3,4,6-tetra-*O*-acetyl-β-D-galactopyranosyl bromide (2.04 g, 4.97 mmol, 1.0 equiv) and NaN_3 (678 mg, 10.4 mmol, 2.1 equiv) were dissolved in dry DMF (20 mL). The solution was stirred for 15 h at 80 °C. After evaporation of the solvent, the residue was taken up in ethyl acetate (50 mL). The inorganic, insoluble residue was filtrated off. The organic phase was washed with a saturated $NaHCO_3$ solution (3 × 30 mL), a 5 wt % HCl solution (2 × 20 mL), and brine (3 × 30 mL). The combined organic phases were dried over Na_2SO_4 and the solvent was removed in vacuo. The crude product was recrystallized from EtOH to afford **1c** (611 mg, 1.64 mmol, 33%) as a colorless, crystalline solid.

^{1}H NMR (400 MHz, $CDCl_3$): δ = 5.43 (dd, $J_{2,3}$ = 3.3 Hz, $J_{2,1}$ = 1.1 Hz, 1H, H-2), 5.21–5.28 (m, 1H, H-4), 5.03 (dd, $J_{3,4}$ = 10.1 Hz, $J_{3,2}$ = 3.2 Hz, 1H, H-3), 4.72 (d, $J_{1,2}$ = 1.2 Hz, 1H, H-1), 4.27 (dd, $J_{6a,6b}$ = 12.4 Hz, $J_{6a,5}$ = 5.6 Hz, 1H, H-6a), 4.17–4.22 (dd, $J_{6b,6a}$ = 12.4 Hz, $J_{6b,5}$ = 2.5 Hz, 1H, H-6b), 3.75 (ddd, $J_{5,4}$ = 9.9 Hz, $J_{5,6a}$ = 5.6 Hz, $J_{5,6b}$ = 2.6 Hz, 1H, H-5), 2.20, 2.10, 2.04, 1.98 (s, 12H, CH_3).

^{13}C NMR (75 MHz, $CDCl_3$): δ = 170.6, 169.9, 169.5 (AcC=O), 85.1 (C-1), 74.6 (C-5), 70.9 (C-3), 69.2 (C-2), 65.3 (C-4), 62.3 (C-6), 20.7, 20.6, 20.5 (CH_3).

m.p. 123 °C (EtOH) (literature 124 °C)[15]. $[\boldsymbol{\alpha}]_{\mathbf{D}}^{\mathbf{20}}$ = – 76.0 (c = 1, $CHCl_3$). **Mass** Anal. Calcd for $C_{14}H_{19}N_3O_9$ $[M+Na]^+$: m/z 396.10; MS found $[M+Na]^+$: m/z 396.06.

2,3,4,6-Tetra-*O*-acetyl-β-D-glucopyranosylamine (2a)

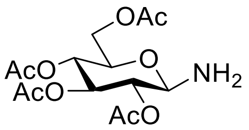

According to General Procedure B, **2a** was obtained from **1a** as a colorless solid. Yield: 99%.

^{1}H NMR (300 MHz, $CDCl_3$): δ = 5.17–5.26 (m, 1H, H-3), 4.98–5.06 (m, 1H, H-4), 4.78–4.86 (m, 1H, H-2), 4.17–4.25 (m, 2H, H-1, H-6a), 4.05–4.13 (m, 1H, H-6b), 3.64–3.71 (ddd, J = 7.2 Hz, J = 4.8 Hz, J = 2.4 Hz, 1H, H-5), 2.34 (br. s., 2H, NH_2), 2.07, 2.05, 2.00, 1.99 (s, 12H, CH_3).

^{13}C NMR (75 MHz, $CDCl_3$): δ = 170.7, 170.2, 170.2, 169.5 (C=O), 84.9 (C-1), 73.1 (C-3), 72.7 (C-5), 72.0 (C-2), 68.7 (C-4), 62.3 (C-6), 20.8, 20.7, 20.6, 20.6 (CH_3).

$[\alpha]_D^{20}$ = + 16.0 (c = 1, $CHCl_3$). **Mass** Anal. Calcd for $C_{14}H_{21}NO_9$ $[M+Na]^+$: m/z 370.31; MS found $[M+Na]^+$: m/z 370.20.

2,3,4,6-Tetra-*O*-acetyl-β-D-galactopyranosylamine (2b)

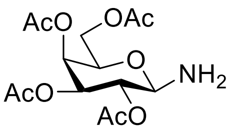

According to General Procedure B, **2b** was obtained from **1b** as a colorless solid. Yield: 99%.

^{1}H NMR (400 MHz, $CDCl_3$): δ = 5.38 (dd, $J_{4,3}$ = 2.9 Hz, $J_{4,5}$ = 0.9 Hz, 1H, H-4), 4.98–5.07 (m, 2H, H-2, H-3), 4.15 (d, $J_{1,2}$ = 8.3 Hz, 1H, H-1), 4.07–4.10 (m, 2H, H-6a, H-6b), 3.88 (td, $J_{5,6}$ = 6.6 Hz, $J_{5,4}$ = 1.0 Hz, 1H, H-5), 2.14, 2.06, 2.04, 1.97 (s, 12H, CH_3).

^{13}C NMR (101 MHz, $CDCl_3$): δ = 170.5, 170.2, 170.1 (AcC=O), 85.3 (C-1), 71.4 (C-5), 71.3 (C-3), 69.7 (C-2), 67.6 (C-4), 61.8 (C-6), 20.9, 20.7, 20.7, 20.6 (CH_3).

$[\alpha]_D^{20}$ = +26.4 (c = 1, $CHCl_3$). **Mass** Anal. Calcd for $C_{14}H_{21}NO_9$ $[M+Na]^+$: m/z 370.31; MS found $[M+Na]^+$: m/z 370.08.

2,3,4,6-Tetra-*O*-acetyl-β-D-mannopyranosylamine (2c)

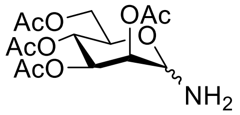

α:β 1:10

According to General Procedure B, **2c** was obtained from **1c** in an anomeric ratio of α:β 1:10 as a colorless solid. Yield: 99%.

^{1}H NMR (400 MHz, $CDCl_3$): δ = 5.39 (dd, $J_{2,3}$ = 3.2 Hz, $J_{2,1}$ = 0.7 Hz, 1H, β-H-2), 5.32 (dd, J = 9.2 Hz, J = 3.3 Hz, 1H, α-H-3), 5.14–5.25 (m, 3H, α-H-2, α-H-4, β-H-4), 5.05 (dd, $J_{3,4}$ = 10.2 Hz, $J_{3,2}$ = 3.3 Hz, 1H, β-H-3), 4.87–4.89 (d, $J_{1,2}$ = 2.5 Hz, 1H, α-H-1), 4.45 (d, $J_{1,2}$ = 1.0 Hz, 1H, β-H-1), 4.27–4.35 (m, 2H, α-H-6a, α-H-6b), 4.24 (dd, $J_{6a,6b}$ = 12.2 Hz, $J_{6a,5}$ = 5.6 Hz, 1H, H-6a), 4.10 (dd, $J_{6b,6a}$ = Hz, $J_{6b,5}$ = 2.3 Hz, 1H, β-H-6b), 3.65 (ddd, $J_{5,4}$ = 8.1 Hz, $J_{5,6a}$ = 5.6 Hz, $J_{5,6b}$ = 2.3 Hz, 2H, α-H-5, β-H-5), 2.14–2.20 (m, 5H, β-CH_3, NH_2), 2.14 (s, 3H, α-CH_3), 2.09 (s, 3H, β-CH_3), 2.05 (s, 3H, α-CH_3), 2.02 (s, 3H, β-CH_3), 2.00 (s, 3H, α-CH_3), 1.96 (s, 3H, β-CH_3).

^{13}C NMR (101 MHz, $CDCl_3$): δ = 170.7, 170.2, 170.1, 169.7 (AcC=O), 82.2 (β-C-1), 81.0 (α-C-1), 73.3 (β-C-5), 72.0 (β-C-3), 70.7 (β-C-2), 69.0 (α-C-3), 68.3 (α-C-5), 66.7 (α-C-2, α-C-4), 65.8 (β-C-4), 62.8 (β-C-6), 62.5 (α-C-6), 20.8, 20.8, 20.7, 20.6 (CH_3).

Mass Anal. Calcd for $C_{14}H_{21}NO_9$ $[M+Na]^+$: m/z 370.11085; HRMS found $[M+Na]^+$: m/z 370.11083.

2,2',3,3',4',6,6'-Hepta-*O*-acetyl-β-D-cellobiosylamine (2d)

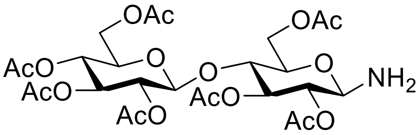

According to General Procedure B, **2d** was obtained from **1d** as a colorless solid. Yield: 99%.

^{1}H NMR (400 MHz, $CDCl_3$): δ = 5.16–5.22 (m, 1H, H-3), 5.08–5.15 (m, 1H, H-3'), 5.01–5.08 (m, 1H, H-4'), 4.87–4.93 (m, 1H, H-2'), 4.69–4.76 (m, 1H, H-2), 4.43–4.53 (m, 2H, H-6a, H-1'), 4.35 (dd, $J_{6a',6b'}$ = 12.5 Hz, $J_{6a',5'}$ = 4.3 Hz, 1H, H-6a'), 4.15 (d, $J_{1,2}$ = 9.0 Hz, 1H, H-1), 4.05–4.11 (m, 1H, H-6b), 4.00–4.05 (m, 1H, H-6b'), 3.66–3.73 (m, 1H, H-4), 3.63 (ddd, $J_{5',4'}$ = 9.8 Hz, $J_{5',6a}$ = 4.3 Hz, $J_{5',6b'}$ = 2.3 Hz, 1H, H-5'), 3.57 (ddd, *J* = 9.8 Hz, *J* = 4.9 Hz, *J* = 1.7 Hz, 1H, H-5), 2.11, 2.07, 2.04, 2.01, 2.00, 1.99, 1.96 (s, 21H, CH_3).

^{13}C NMR (101 MHz, $CDCl_3$): δ = 170.5, 170.4, 170.4, 170.2, 169.7, 169.3, 169.0 (AcC=O), 100.8 (C-1'), 84.5 (C-1), 76.8 (C-4), 73.7 (C-5), 72.9 (C-3'), 72.6 (C-3), 72.3 (C-2), 71.9 (C-5'), 71.6 (C-2'), 67.8 (C-4'), 62.2 (C-6), 61.5 (C-6'), 20.9, 20.8, 20.6, 20.5, 20.5 (CH_3).

$[\alpha]_D^{20}$ = – 2.5 (c = 0.5, $CHCl_3$). **Mass** Anal. Calcd for $C_{26}H_{37}N_3O_{17}$ $[M+H]^+$: m/z 636.31; MS found $[M+H]^+$: m/z 684.18.

2,2',3,3',4',6,6'-Hepta-*O*-acetyl-β-D-lactosylamine (2e)

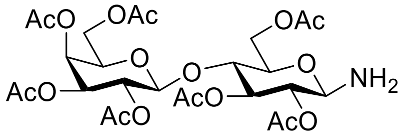

According to General Procedure B, **2e** was obtained from **1e** as a colorless solid. Yield: 99%.

^{1}H NMR (400 MHz, $CDCl_3$): δ = 5.33 (dd, J = 3.3 Hz, J = 0.9 Hz, 1H, H-4'), 5.18–5.24 (m, 1H, H-3), 5.09 (dd, J = 10.4 Hz, J = 7.9 Hz, 1H, H-2'), 4.93 (dd, J = 10.4 Hz, J = 3.4 Hz, 1H, H-3'), 4.70–4.76 (m, 1H, H-2), 4.42–4.47 (m, 2H, H-6a, H-1'), 4.16 (d, $J_{1,2}$ = 9.0 Hz, 1H, H-1), 4.03–4.14 (m, 3H, H-6b, H-6a', H-6b'), 3.82–3.88 (m, 1H, H-5'), 3.69–3.76 (m, 1H, H-4), 3.59 (ddd, J = 9.9 Hz, J = 5.1 Hz, J = 2.0 Hz, 1H, H-5), 2.13, 2.11, 2.05, 2.04, 2.03, 2.02, 1.94 (s, 21H, CH_3).

^{13}C NMR (101 MHz, $CDCl_3$): δ = 170.5, 170.4, 170.3, 170.1, 170.0, 169.6, 169.0 (AcC=O), 101.0 (C-1'), 84.6 (C-1), 76.6 (C-4), 73.6 (C-5), 72.9 (C-3), 72.4 (C-2), 71.0 (C-3'), 70.6 (C-5'), 69.1 (C-2'), 66.6 (C-4'), 62.3 (C-6), 60.8 (C-6'), 20.9, 20.8, 20.6, 20.5 (CH_3).

$[\alpha]_D^{20}$ = + 4.7 (c = 0.5, $CHCl_3$). **Mass** Anal. Calcd for $C_{26}H_{37}N_3O_{17}$ $[M+Na]^+$: m/z 636.31; MS found $[M+H]^+$: m/z 636.12.

2,2',3,3',4',6,6'-Hepta-*O*-acetyl-β-D-maltosylamine (2f)

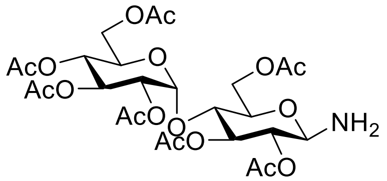

According to General Procedure B, **2f** was obtained from **1f** as a colorless solid. Yield: 82%.

^{1}H NMR (400 MHz, $CDCl_3$): δ = 5.39 (d, $J_{1‘,2‘}$ = 4.0 Hz, 1H, H-1‘), 5.34 (dd, $J_{3‘,2‘}$ = 10.4 Hz, $J_{3‘,4‘}$ = 9.7 Hz, 1H, H-3‘), 5.25–5.31 (m, 1H, H-3), 5.00–5.07 (m, 1H, H-4‘), 4.82–4.87 (dd, $J_{2‘,3‘}$ = 10.5 Hz, $J_{2‘,1‘}$ = 4.0 Hz, H-2‘), 4.65–4.72 (m, 1H, H-2), 4.46 (dd, $J_{6a,6b}$ = 12.1 Hz, $J_{6a,5}$ = 2.4 Hz, 1H, H-6a), 4.21–4.28 (m, 2H, H-1, H-6a‘), 4.17 (dd, $J_{6b,6a}$ = 12.1 Hz, $J_{6b,5}$ = 4.4 Hz, 1H, H-6b), 4.03 (dd, $J_{6b‘,6a‘}$ = 12.5 Hz, $J_{6b‘,5‘}$ = 2.2 Hz, 1H, H-6b‘), 3.91–3.98 (m, 2H, H-4, H-5‘), 3.67 (ddd, $J_{5,4}$ = 7.2 Hz, $J_{5,6b}$ = 4.5 Hz, $J_{5,6a}$ = 2.2 Hz, 1H, H-5), 2.13, 2.08, 2.04, 2.03, 2.01, 1.99, 1.98 (s, 21H, CH_3).

^{13}C NMR (101 MHz, $CDCl_3$): δ = 170.6, 170.6, 170.5, 170.4, 170.0, 169.9, 169.4 (AcC=O), 95.5 (C-1’), 83.7 (C-1), 75.5 (C-3), 73.2 (C-5), 72.8, 72.7 (C-2, C-4), 69.9 (C-2’), 69.3 (C-3’), 68.5 (C-5’), 67.9 (C-4’), 63.0 (C-6), 61.4 (C-6’), 20.9, 20.8, 20.7, 20.6, 20.6 (CH_3).

$[\alpha]_D^{20}$ = + 38.6 (c = 0.5, $CHCl_3$). **Mass** Anal. Calcd for $C_{26}H_{37}N_3O_{17}$ $[M+H]^+$: m/z 636.21343; HRMS found $[M+Na]^+$: m/z 636.21422.

***N*^{2}-[(9*H*-Fluoren-9-ylmethoxy)carbonyl]-*N*-(2,3,4,6-tetra-*O*-acetyl-β-D-glucopyranosyl)-L-asparagine *tert*-butyl ester (3a)**

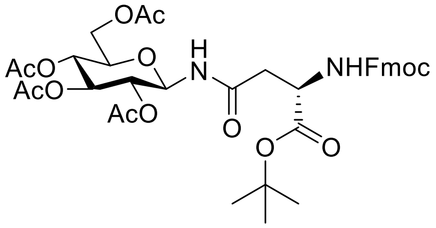

According to General Procedure C, **3a** was obtained from **2a** and Fmoc-L-Asp-O*t*-Bu as a colorless solid. Yield: 91% (Procedure C1)/82% (Procedure C2).

^{1}H NMR (400 MHz, $CDCl_3$): δ = 7.75, 7.59 (d, *J* = 7.5 Hz, 4H, Fmoc-H_{arom}), 7.36–7.40, 7.26–7.33 (m, 4H, Fmoc-H_{arom}), 6.41 (d, *J* = 9.0 Hz, 1H, C-1-NH), 5.92 (dd, *J* = 8.6 Hz, 1H, CHNHFmoc), 5.27–5.34 (m, 1H, H-3), 5.20–5.25 (m, 1H, H-1), 5.02–5.08 (m,

1H, H-4), 4.88–4.94 (m, 1H, H-2), 4.45–4.53 (m, 1H, CHNHFmoc), 4.38–4.45 (m, 1H, Fmoc-CH), 4.25–4.34 (m, 2H, Fmoc-CH_2, H-6a), 4.18–4.24 (m, 1H, Fmoc-CH_2), 4.00–4.06 (m, 1H, H-6b), 3.78 (ddd, *J* = 6.2 Hz, *J* = 4.2 Hz, *J* = 2.1 Hz, 1H, H-5), 2.65–2.89 (m, 2H, Asn-CH_2), 2.06, 2.02, 2.01 (s, 12H, CH_3), 1.44 (s, 9H, $C(CH_3)_3$).

^{13}C NMR (101 MHz, $CDCl_3$): δ = 171.2, 170.7, 170.5, 169.9, 169.7, 169.5 (AcC=O, ^{t}BuC=O, NHC=O), 156.1 (NHC=O), 143.9, 143.7 ($C_{q,arom}$), 141.2, 141.3, 127.7, 127.1, 125.1, 120.0 (Fmoc-C_{arom}), 82.5 ($\underline{C}(CH_3)_3$), 78.1 (C-1), 73.7 (C-5), 72.6 (C-3), 70.6 (C-2), 68.0 (C-4), 67.2 (Fmoc-CH_2), 61.5 (C-6), 51.0 (CHNHFmoc), 47.1 (Fmoc-CH), 38.0 (Asn-CH_2), 27.9 ($C(\underline{C}H_3)_3$), 20.7, 20.6, 20.5 (CH_3).

$[\alpha]_D^{20}$ = + 17.1 (c = 1, $CHCl_3$). **Mass** Anal. Calcd for $C_{37}H_{44}N_2O_{14}$ $[M+Na]^+$: m/z 763.26847; HRMS found $[M+Na]^+$: m/z 763.26856.

N^2-[(9*H*-Fluoren-9-ylmethoxy)carbonyl]-*N*-(2,3,4,6-tetra-*O*-acetyl-β-D-galactopyranosyl)-L-asparagine-*tert*-butyl ester (3b)

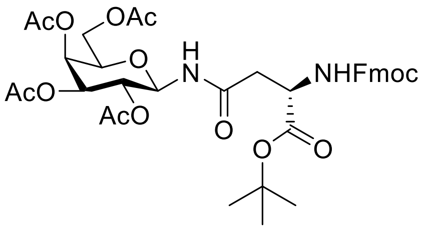

According to General Procedure C, **3b** was obtained from **2b** and Fmoc-L-Asp-O*t*-Bu as a colorless solid. Yield: 92% (Procedure C1)/84% (Procedure C2).

^{1}H NMR (400 MHz, $CDCl_3$): δ = 7.75, 7.59 (d, *J* = 7.5 Hz, 4H, Fmoc-H_{arom}), 7.35–7.41, 7.26–7.33 (m, 4H, Fmoc-H_{arom}), 6.41 (d, *J* = 9.0 Hz, 1H, C-1-NH), 5.93 (dd, *J* = 8.6 Hz, 1H, CHNHFmoc), 5.41 – 5.44 (m, 1H, H-3), 5.17–5.24 (m, 1H, H-1), 5.05–5.16 (m, 2H, H-4, H-2), 4.46–4.56 (m, 1H, CHNHFmoc), 4.42 (dd, *J* = 10.4 Hz, *J* = 7.6 Hz, 1H, Fmoc-CH_2),

4.26–4.34 (m, 1H, Fmoc-CH_2), 4.18–4.24 (m, 1H, Fmoc-CH), 4.04–4.16 (m, 2H, H-6a, H-6b), 3.97–4.04 (m, 1H, H-5), 2.86 (dd, J = 16.4 Hz, J = 4.2 Hz, 1H, Asn-CH_2), 2.71 (dd, J = 16.4 Hz, J = 4.0 Hz, 1H, Asn-CH_2), 2.12, 2.07, 2.01, 1.99 (s, 12H, CH_3), 1.44 (s, 9H, $C(CH_3)_3$).

^{13}C NMR (101 MHz, $CDCl_3$): δ = 171.5, 170.6, 170.3, 170.0, 169.8 (AcC=O, ^{t}BuC=O, NHC=O), 156.1 (NHC=O), 143.9, 143.7, 141.3 ($C_{q,arom}$), 127.7, 127.1, 125.1, 120.0 (Fmoc-C_{arom}), 82.4 ($\underline{C}(CH_3)_3$), 78.4 (C-1), 72.4 (C-5), 70.7, 68.3 (C-4, C-2), 67.2 (Fmoc-CH_2), 67.1 (C-3), 61.1 (C-6), 51.0 (CHNHFmoc), 47.1 (Fmoc-CH), 38.0 (Asn-CH_2), 27.8 ($C(\underline{C}H_3)_3$), 20.7, 20.6, 20.6, 20.5 (CH_3).

$[\alpha]_D^{20}$ = + 11.5 (c = 1, $CHCl_3$). **Mass** Anal. Calcd for $C_{37}H_{44}N_2O_{14}$ $[M+Na]^+$: m/z 763.27; HRMS found $[M+Na]^+$: m/z 763.26778.

N^2-[(9*H*-Fluoren-9-ylmethoxy)carbonyl]-*N*-(2,3,4,6-tetra-*O*-acetyl-β-D-mannopyranosyl)-L-asparagine-*tert*-butyl ester (3c)

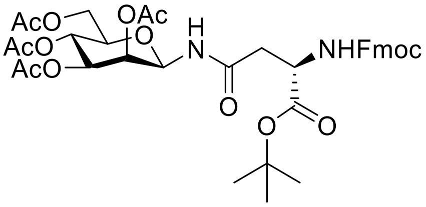

According to General Procedure C, **3c** was obtained from **2c** and Fmoc-L-Asp-O*t*-Bu as a colorless solid. Yield: 77% (Procedure C1)/80% (Procedure C2).

^{1}H NMR (400 MHz, $CDCl_3$): δ = 7.75, 7.58 (d, J = 7.5 Hz, 4H, Fmoc-H_{arom}), 7.36–7.42, 7.27–7.33 (m, 4H, Fmoc-H_{arom}), 6.48 (d, J = 9.1 Hz, 1H, C-1-NH), 5.87 (d, J = 7.3 Hz, 1H, CHN$\underline{H}$Fmoc), 5.49–5.55 (m, 1H, H-1), 5.36 (dd, $J_{2,3}$ = 3.1 Hz, $J_{2,1}$ = 0.8 Hz, 1H, H-2), 5.18–5.26 (m, 1H, H-4), 5.09 (dd, $J_{3,4}$ = 10.1 Hz, $J_{3,2}$ = 3.3 Hz, 1H, H-3), 4.35–4.46 (m, 2H,

Fmoc-CH_2, C<u>H</u>NHFmoc), 4.25–4.35 (m, 2H, Fmoc-CH_2, H-6a), 4.17–4.24 (m, 1H, Fmoc-CH), 3.99–4.06 (m, 1H, H-6b), 3.73 (ddd, $J_{5,4}$ = 7.1 Hz, $J_{5,6}$ = 4.9 Hz, $J_{5,6}$ = 2.1 Hz, 1H, H-5), 2.86 (d, J = 15.7 Hz, J = 2.5 Hz, 1H, Asn-CH_2), 2.77 (dd, J = 16.4 Hz, J = 3.8 Hz, 1H, Asn-CH_2), 2.23, 2.03, 2.02, 1.97 (s, 12H, CH3), 1.46 (s, 9H, $C(CH_3)_3$).

^{13}C NMR (101 MHz, $CDCl_3$): δ = 170.6, 170.4, 169.9, 169.6, 169.6, 169.5 (AcC=O, ^{t}BuC=O, NHC=O), 143.8, 143.7, 141.3 ($C_{q,arom}$), 127.7, 127.1, 125.1, 120.0 (Fmoc-C_{arom}), 82.9 (<u>C</u>$(CH_3)_3$), 75.9 (C-1), 74.2 (C-5), 71.5 (C-3), 69.9 (C-2), 67.3 (Fmoc-CH_2), 65.0 (C-4), 62.1 (C-6), 51.0 (CHNHFmoc), 47.1 (Fmoc-CH), 38.6 (Asn-CH_2), 27.9 (C(<u>C</u>$H_3)_3$), 20.9, 20.7, 20.7, 20.5 (CH_3).

$[\alpha]_D^{20}$ = + 1.2 (c = 1, $CHCl_3$). **Mass** Anal. Calcd for $C_{37}H_{44}N_2O_{14}$ $[M+Na]^+$: m/z 763.26847; HRMS found $[M+Na]^+$: m/z 763.26842.

***N*^{2}-[(9*H*-Fluoren-9-ylmethoxy)carbonyl]-*N*-(2,2',3,3',4,6,6'-hepta-*O*-acetyl-β-D-cellobiosyl)-L-asparagine-*tert*-butyl ester (3d)**

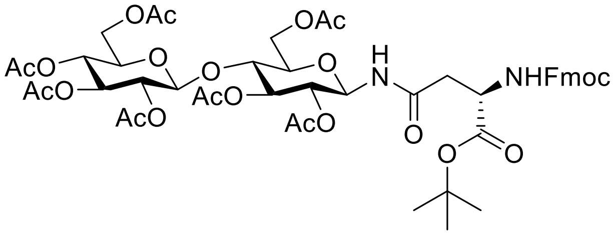

According to General Procedure C, **3d** was obtained from **2d** and Fmoc-L-Asp-O*t*-Bu as a colorless solid. Yield: 61% (Procedure C1)/79% (Procedure C2).

^{1}H NMR (400 MHz, $CDCl_3$): δ = 7.75, 7.58 (d, J = 7.5 Hz, 4H, Fmoc-H_{arom}), 7.35–7.41, 7.26–7.32 (m, 4H, Fmoc-H_{arom}), 6.33 (d, J = 8.9 Hz, 1H, C-1-NH), 5.92 (d, J = 8.7 Hz, 1H, CHN<u>H</u>Fmoc), 5.23–5.31 (m, 1H, H-3), 5.14–5.20 (m, 1H, H-1), 5.05–5.14 (m, 1H, H-3'), 5.02–5.08 (m, 1H, H-4'), 4.91 (dd, J = 9.0 Hz, J = 8.1 Hz, 1H, H-2'), 4.79–4.85 (m, 1H, H-2),

4.39–4.50 (m, 4H, CHNHFmoc, Fmoc-CH_2, H-1‘, H-6a‘), 4.35 (dd, J = 12.5 Hz, J = 4.4 Hz, 1H, Fmoc-CH_2), 4.25–4.32 (m, 1H, H-6b‘), 4.18–4.24 (m, 1H, H-Fmoc-CH), 4.07–4.14 (m, 1H, H-6a), 4.03 (dd, $J_{6b,6a}$ = 12.5 Hz, $J_{6b,5}$ = 2.1 Hz, 1H, H-6b), 3.67–3.77 (m, 2H, H-4, H-5), 3.61–3.66 (m, 1H, H-5‘), 2.81 (d, J = 16.5 Hz, J = 4.3 Hz, 1H, Asn-CH_2), 2.68 (d, J = 16.3 Hz, J = 3.8 Hz, 1H, Asn-CH_2), 2.07, 2.04, 2.02, 2.01, 1.99, 1.97 (s, 21H, CH_3), 1.43 (s, 9H, $C(CH_3)_3$).

^{13}C NMR (101 MHz, $CDCl_3$): δ = 171.4, 170.6, 170.5, 170.2, 169.7, 169.4, 169.3, 169.0 (AcC=O, ^{t}BuC=O, NHC=O), 156.1 (NHC=O), 143.8, 143.7, 141.2 ($C_{q,arom}$), 127.7, 127.0, 125.1, 120.0 (Fmoc-C_{arom}), 100.6 (C-1‘), 82.5 ($\underline{C}(CH_3)_3$), 78.0 (C-1), 76.1 (C-5), 74.6 (C-4), 72.9 (C-3‘), 72.0, 72.0 (C-3, C-5‘), 71.5 (C-2‘), 70.8 (C-2), 67.8 (C-4‘), 67.2 (Fmoc-CH_2), 61.7, 61.6 (C-6, C-6‘), 51.0 (CHNHFmoc), 47.1 (Fmoc-CH), 38.0 (Asn-CH_2), 27.8 ($C(\underline{C}H_3)_3$), 20.8, 20.6, 20.5 (CH_3).

$[\alpha]_D^{20}$ = + 1.0 (c = 1, $CHCl_3$). **Mass** Anal. Calcd for $C_{49}H_{60}N_2O_{22}$ $[M+Na]^+$: m/z 1051.35299; HRMS found $[M+Na]^+$: m/z 1051.35278.

N^2-[(9H-Fluoren-9-ylmethoxy)carbonyl]-N-(2,2’,3,3’,4’,6,6’-hepta-O-acetyl-β-D-lactosyl)-L-asparagine-*tert*-butyl ester (3e)

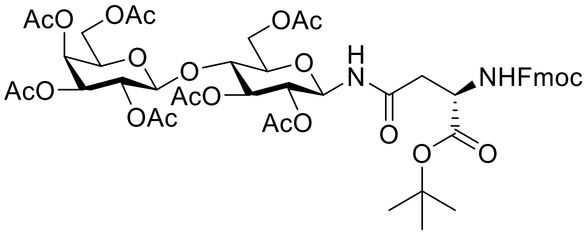

According to General Procedure C, **3e** was obtained from **2e** and Fmoc-L-Asp-Ot-Bu as a colorless solid. Yield: 63% (Procedure C1)/61% (Procedure C2).

^{1}**H NMR** (300 MHz, $CDCl_3$): δ = 7.75, 7.58 (d, *J* = 7.3 Hz, 4H, Fmoc-H_{arom}), 7.35–7.42, 7.26–7.33 (m, 4H, Fmoc-H_{arom}), 6.33 (d, *J* = 9.0 Hz, 1H, C-1-NH), 5.82 (d, *J* = 8.4 Hz, 1H, CHNHFmoc), 5.34 (dd, $J_{4‘,3‘}$ = 3.4 Hz, $J_{4‘,5‘}$ = 0.7 Hz, 1H, H-4‘), 5.25–5.32 (m, 1H, H-3), 5.13– 5.22 (m, 1H, H-1), 5.09 (dd, $J_{2‘,3‘}$ = 10.4 Hz, $J_{2‘,1‘}$ = 7.8 Hz, 1H, H-2‘), 4.93 (dd, $J_{3‘,2‘}$ = 10.5 Hz, $J_{3‘,4‘}$ = 3.4 Hz, 1H, H-3‘), 4.77–4.86 (m, 1H, H-2), 4.35–4.52 (m, 4H, CHNHFmoc, H-6a, Fmoc-CH_2, H-1‘), 4.25–4.34 (m, 1H, Fmoc-CH_2), 4.22 (dd, *J* = 14.2 Hz, *J* = 7.1 Hz, 1H, Fmoc-CH), 4.01–4.16 (m, 3H, H-6b, H-6a‘, H-6b‘), 3.81–3.89 (m, 1H, H-5‘), 3.65–3.80 (m, 2H, H-5, H-4), 2.83 (dd, *J* = 16.3 Hz, *J* = 4.2 Hz, 1H, Asn-CH_2), 2.66 (dd, *J* = 16.1 Hz, *J* = 3.9 Hz, 1H, Asn-CH_2), 2.14, 2.06, 2.05, 2.04, 2.03, 1.95 (s, 21H, CH_3), 1.43 (s, 9H, $C(CH_3)_3$).

^{13}**C NMR** (75 MHz, $CDCl_3$): δ = 171.4, 170.6, 170.3, 170.3, 170.1, 170.1, 169.3, 169.0 (AcC=O, ^{t}BuC=O, NHC=O), 156.1 (NHC=O), 143.9, 143.7, 141.3 ($C_{q,arom}$), 127.7, 127.1, 125.1, 120.0 (Fmoc-C_{arom}), 100.9 (C-1‘), 82.5 ($\underline{C}(CH_3)_3$), 78.0 (C-1), 75.9 (C-4), 74.6 (C-5), 72.2 (C-3), 71.0, 70.9 (C-2, C-3‘), 70.7 (C-4‘), 69.0 (C-2‘), 67.2 (Fmoc-CH_2), 66.6 (C-4‘), 61.9 (C-6), 60.8 (C-6‘), 51.0 (CHNHFmoc), 47.1 (Fmoc-CH), 38.0 (Asn-CH_2), 27.8 ($C(\underline{C}H_3)_3$), 20.8, 20.7, 20.6, 20.6, 20.5 (CH_3).

$[\alpha]_D^{20}$ = + 6.3 (c = 1, $CHCl_3$). **Mass** Anal. Calcd for $C_{49}H_{60}N_2O_{22}$ $[M+Na]^+$: m/z 1051.35299; HRMS found $[M+Na]^+$: m/z 1051.35263.

N^2-[(9*H*-Fluoren-9-ylmethoxy)carbonyl]-*N*-(2,2',3,3',4,6,6'-hepta-*O*-acetyl-β-D-maltosyl) -L-asparagine-*tert*-butyl ester (3f)

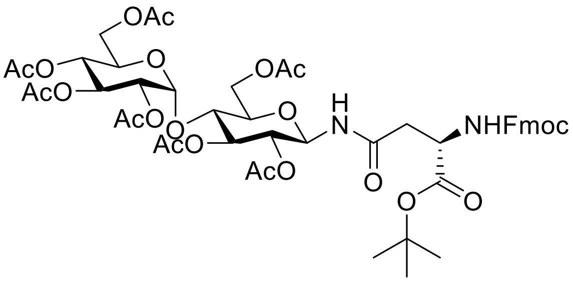

According to General Procedure C, **3f** was obtained from **2f** and Fmoc-L-Asp-O*t*-Bu as a colorless solid. Yield: 56% (Procedure C1)/86% (Procedure C2).

^{1}H NMR (400 MHz, $CDCl_3$): δ = 7.75, 7.59 (d, *J* = 7.5 Hz, 4H, Fmoc-H_{arom}), 7.35–7.42, 7.27–7.32 (m, 4H, Fmoc-H_{arom}), 6.29 (d, *J* = 9.3 Hz, 1H, C-1-NH), 5.94 (d, *J* = 8.4 Hz, 1H, CHN<u>H</u>Fmoc), 5.38 (d, $J_{1',2'}$ = 4.2 Hz, 1H, H-1'), 5.31–5.37 (m, 2H, H-3, H-3'), 5.20–5.28 (m, 1H, H-1), 5.01–5.08 (m, 1H, H-4'), 4.85 (dd, $J_{2',3'}$ = 10.5 Hz, $J_{2',1'}$ = 4.0 Hz, 1H, H-2'), 4.71–4.78 (m, 1H, H-2), 4.44–4.51 (m, 1H, C<u>H</u>NHFmoc), 4.36–4.43 (m, 2H, Fmoc-CH_2, H-6a), 4.26–4.34 (dd, *J* = 10.2 Hz, *J* = 7.2 Hz, 1H, Fmoc-CH_2), 4.17–4.24 (m, 3H, Fmoc-CH, H-6b, H-6a'), 4.02 (dd, $J_{6b',6a'}$ = 12.4 Hz, $J_{6b',5'}$ = 1.5 Hz, 1H, H-6b'), 3.93–3.99 (m, 1H, H-4), 3.87–3.93 (m, 1H, H-5'), 3.76 (ddd, $J_{5,4}$ = 9.5 Hz, $J_{5,6}$ = 3.7 Hz, $J_{5,6}$ = 2.5 Hz, 1H, H-5), 2.81 (dd, *J* = 16.4 Hz, *J* = 3.9 Hz, 1H, Asn-CH_2), 2.69 (dd, *J* = 16.5 Hz, *J* = 3.9 Hz, 1H, Asn-CH_2), 2.08, 2.05, 2.03, 2.01, 1.99 (s, 21H, CH_3), 1.43 (s, 9H, $C(CH_3)_3$).

^{13}C NMR (101 MHz, $CDCl_3$): δ = 171.2, 170.7, 170.5, 170.4, 169.9, 169.7, 169.5 (AcC=O, ^{t}BuC=O, NHC=O), 156.1 (NHC=O), 143.8, 143.7, 141.2 ($C_{q,arom}$), 127.7, 127.1, 125.1, 120.0 (Fmoc-C_{arom}), 95.6 (C-1'), 82.5 (<u>C</u>$(CH_3)_3$), 77.6 (C-1), 74.9 (C-3), 74.0 (C-5), 72.4 (C-4), 71.3 (C-2), 69.9 (C-2'), 69.3 (C-3'), 68.6 (C-5'), 67.9 (C-4'), 67.2 (Fmoc-CH_2), 62.6 (C-6), 61.4 (C-6'), 50.9 (CHNHFmoc), 47.1 (Fmoc-CH), 38.0 (Asn-CH_2), 27.8 (C(<u>C</u>$H_3)_3$), 20.9, 20.8, 20.7, 20.6 (CH_3).

$[\alpha]_D^{20}$ = + 65.2 (c = 1, $CHCl_3$). **Mass** Anal. Calcd for $C_{49}H_{60}N_2O_{22}$ $[M+Na]^+$: m/z 1051.35299; HRMS found $[M+Na]^+$: m/z 1051.35262.

***N*²-[*N*²-[(9*H*-Fluoren-9-ylmethoxy)carbonyl]phenylalaninyl]-*N*-(2,3,4,6-tetra-*O*-acetyl-β-D-glucopyranosyl)-L-asparagine-*tert*-butyl ester (4a)**

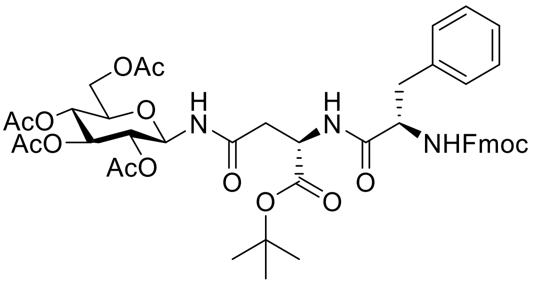

According to General Procedure D, **4a** was obtained from **3a** and Fmoc-L-Phe-OH as a colorless solid. Yield: 81% (Procedure D1)/76% (Procedure D2).

¹H NMR (400 MHz, $CDCl_3$): δ = 7.74 (d, *J* = 7.6 Hz, 2H, Fmoc-H_{arom}), 7.48–7.57, 7.36–7.40 (m, 4H, Fmoc-H_{arom}), 7.26–7.31 (m, 4H, Fmoc-H_{arom}, Ph-H_{arom}), 7.18–7.24 (m, 3H, Ph-H_{arom}), 7.05 (d, *J* = 7.2 Hz, 1H, CHNHPhe), 6.57 (d, *J* = 8.8 Hz, 1H, C-1-NH), 5.46 (d, *J* = 6.8 Hz, 1H, CHNHAsn), 5.21–5.30 (m, 1H, H-3), 5.1 –5.16 (m, 1H, H-1), 4.97–5.02 (m, 1H, H-4), 4.89–4.96 (m, 1H, H-2), 4.60–4.66 (m, 1H, CHNHFmoc), 4.47–4.53 (m, 1H, CHNHAsn), 4.35–4.43 (m, 1H, Fmoc-CH_2), 4.25–4.35 (m, 2H, Fmoc-CH_2, H-6a), 4.14–4.19 (m, 1H, Fmoc-CH), 3.97 3.97 (dd, $J_{6b,6a}$ = 12.4 Hz, *J* = 1.5 Hz, 1H, H-6b), 3.68–3.72 (m, 1H, H-5), 3.20, (dd, *J* = 12.7 Hz, *J* = 2.0 Hz, 1H, Asn-CH_2) 2.99 (dd, *J* = 13.0 Hz, *J* = 8.1 Hz, 1H, Asn-CH_2), 2.77 (dd, *J* = 15.8 Hz, *J* = 3.9 Hz, 1H, Phe-CH_2), 2.69 (dd, *J* = 16.1 Hz, *J* = 4.2 Hz, 1H, Phe-CH_2), 2.04, 2.02, 1.98 (s, 12H, CH_3), 1.43 (s, 9H, $C(CH_3)_3$).

¹³C NMR (101 MHz, $CDCl_3$): δ = 170.9, 170.7, 170.4, 169.9, 169.5, 169.0 (AcC=O, *ᵗ*BuC=O, NHC=O), 155.9 (NHC=O), 143.8, 141.2, 136.1 ($C_{q,arom}$), 129.3, 128.7 (Ph-C_{arom}), 127.7 (Fmoc-C_{arom}), 127.1 (Ph-C_{arom}), 125.1, 119.9 (Fmoc-C_{arom}), 82.7 ($C(CH_3)_3$), 78.1 (C-1), 73.8

(C-5), 72.7 (C-3), 70.4 (C-2), 68.2 (C-4), 67.0 (Fmoc-CH_2), 61.4 (C-6), 56.0 (CHNHAsn), 49.5 (CHNHFmoc), 47.1 (Fmoc-CH), 37.9 (Asn-CH_2), 37.7 (Phe-CH_2), 27.8 ($C(\underline{C}H_3)_3$), 20.7, 20.6, 20.6, 20.5 (CH_3).

$[\alpha]_D^{20}$ = + 2.9 (c = 1, $CHCl_3$). **Mass** Anal. Calcd for $C_{46}H_{53}N_3O_{15}$ $[M+Na]^+$: m/z 910.33689; HRMS found $[M+Na]^+$: m/z 910.33544. **Anal**. Calcd for $C_{49}H_{60}N_2O_{22}$ C: 62.22%, H: 6.02%, N: 4.73%; found C: 61.81%, H: 6.03%, N: 4.76%.

N^2-[N^2-[(9*H*-Fluoren-9-ylmethoxy)carbonyl]phenylalaninyl]-*N*-(2,3,4,6-tetra-*O*-acetyl-β-D-galactopyranosyl)-L-asparagine-*tert*-butyl ester (4b)

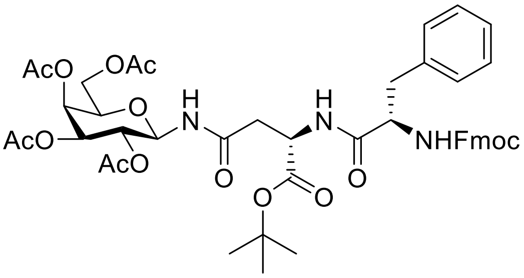

According to General Procedure D, **4b** was obtained from **3b** and Fmoc-L-Phe-OH as a colorless solid. Yield: 67% (Procedure D1)/73% (Procedure D2).

^{1}H NMR (400 MHz, $CDCl_3$): δ = 7.74 (d, *J* = 7.6 Hz, 2H, Fmoc-H_{arom}), 7.48–7.57, 7.34–7.41 (m, 4H, Fmoc-H_{arom}), 7.25–7.31 (m, 4H, Fmoc-H_{arom}, Ph-H_{arom}), 7.19–7.25 (m, 3H, Ph-H_{arom}), 7.11 (d, *J* = 6.6 Hz, 1H, CHN<u>H</u>Fmoc), 6.60–6.69 (m, 1H, C-1-NH), 5.53 (d, *J* = 7.5 Hz, 1H, CHN<u>H</u>Asn), 5.35–5.39 (m, 1H, H-3), 5.03–5.16 (m, 3H, H-1, H-2, H-4), 4.60–4.70 (m, 1H, C<u>H</u>NHFmoc), 4.44–4.54 (m, 1H, C<u>H</u>NHAsn), 4.35–4.43 (m, 1H, Fmoc-CH_2), 4.19–4.33 (m, 2H, Fmoc-CH_2, H-6a), 4.14–4.19 (m, 1H, Fmoc-CH), 3.99 (dd, $J_{6b,6a}$ = 11.3 Hz, $J_{6b,5}$ = 5.7 Hz, 1H, H-6b), 3.86–3.93 (m, 1H, H-5), 3.21 (dd, *J* = 12.8 Hz, *J* = 2.9 Hz, 1H, Asn-CH_2), 2.99 (dd, *J* = 13.0 Hz, *J* = 8.6 Hz, 1H, Asn-CH_2), 2.67–2.83 (m, 2H, Phe-CH_2), 2.06, 2.02, 1.96, 1.94 (s, 12H, CH_3), 1.44 (s, 9H, $C(CH_3)_3$).

^{13}C NMR (101 MHz, $CDCl_3$): δ = 171.1, 170.9, 170.8, 170.6, 170.1, 169.8, 169.1 (AcC=O, ^{t}BuC=O, NHC=O), 155.9 (NHC=O), 143.8, 143.7, 141.2, 136.4 ($C_{q,arom}$), 129.3, 128.6 (Ph-C_{arom}), 127.6 (Fmoc-C_{arom}), 127.0 (Ph-C_{arom}, Fmoc-C_{arom}), 125.1, 119.9 (Fmoc-C_{arom}), 82.7 ($\underline{C}(CH_3)_3$), 78.5 (C-1), 72.6 (C-5), 70.8, 68.1 (C-4, C-2), 67.2 (C-3), 67.0 (Fmoc-CH_2), 61.2 (C-6), 56.2 (CHNHAsn), 49.7 (CHNHFmoc), 47.1 (Fmoc-CH), 38.2 (Asn-CH_2), 37.9 (Phe-CH_2), 27.9 ($C(\underline{C}H_3)_3$), 20.7, 20.7, 20.5, 20.3 (CH_3).

$[\boldsymbol{\alpha}]_{\mathbf{D}}^{\mathbf{20}}$ = + 14.7 (c = 1, $CHCl_3$). **Mass** Anal. Calcd for $C_{46}H_{53}N_3O_{15}$ $[M+Na]^+$: m/z 910.33689; HRMS found $[M+Na]^+$: m/z 910.33750. **Anal**. Calcd for $C_{49}H_{60}N_2O_{22}$ C: 62.22%, H: 6.02%, N: 4.73%; found C: 62.13%, H: 6.24%, N: 4.58%.

***N*^{2}-[*N*^{2}-[(9*H*-Fluoren-9-ylmethoxy)carbonyl]phenylalaninyl]-*N*-(2,3,4,6-tetra-*O*-acetyl-β-D-mannopyranosyl)-L-asparagine-*tert*-butyl ester (4c)**

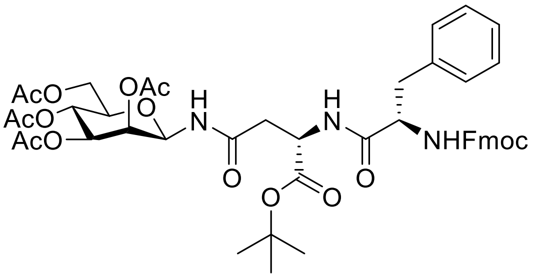

According to General Procedure D, **4c** was obtained from **3c** and Fmoc-L-Phe-OH as a colorless solid. Yield: 72% (Procedure D1)/80% (Procedure D2).

^{1}H NMR (400 MHz, $CDCl_3$): δ = 7.74 (d, J = 7.6 Hz, 2H, Fmoc-H_{arom}), 7.47–7.54, 7.34–7.43 (m, 4H, Fmoc-H_{arom}), 7.26–7.32 (m, 4H, Fmoc-$H_{arom,}$ Ph-H_{arom}), 7.18–7.24 (m, 3H, Ph-H_{arom}), 7.12 (d, J = 7.5 Hz, 1H, CHN$\underline{H}$Asn), 6.82 (d, J = 7.6 Hz, 1H, C-1-NH), 5.60 (d, J = 6.9 Hz, 1H, CHN$\underline{H}$Fmoc), 5.44 (dd, $J_{1,NH}$ = 9.2 Hz, $J_{1,2}$ = 0.7 Hz, 1H, H-1), 5.34 (dd, $J_{2,3}$ = 3.2 Hz, $J_{2,1}$ = 1.0 Hz, 1H, H-2), 5.17–5.25 (m, 1H, H-4), 5.09 (dd, $J_{3,4}$ = 10.1 Hz, $J_{3,2}$ = 3.3 Hz, 1H,

H-3), 4.46–4.59 (m, 2H, H-CHNHFmoc, CHNHAsn), 4.39 (dd, J = 10.5 Hz, J = 7.0 Hz, 2H, Fmoc-CH_2, H-6a), 4.20–4.30 (m, 1H, Fmoc-CH_2), 4.12–4.18 (m, 1H, Fmoc-CH), 4.03 (dd, $J_{6b,6a}$ = 12.4 Hz, $J_{6b,5}$ = 1.9 Hz, 1H, H-6b), 3.71 (ddd, $J_{5,4}$ = 9.7 Hz, $J_{5,6a}$ = 5.1 Hz, $J_{5,6b}$ = 1.7 Hz, 1H, H-5), 3.21 (dd, J = 13.0 Hz, J = 5.3 Hz, 1H, Asn-CH_2), 3.01 (dd, J = 12.6 Hz, J = 8.7 Hz, 1H, Asn-CH_2), 2.69–2.85 (m, 2H, Phe-CH_2), 2.19 2.04, 1.98, 1.97 (s, 12H, CH_3), 1.43 (s, 9H, $C(CH_3)_3$)

^{13}C NMR (101 MHz, $CDCl_3$): δ = 171.5, 170.9, 170.5, 169.9, 169.9, 169.6, 169.3, 169.0 (AcC=O, ^{t}BuC=O, NHC=O), 156.0 (NHC=O), 143.5, 143.3, 141.3 ($C_{q,arom}$), 129.2, 128.7 (Ph-C_{arom}), 127.7 (Fmoc-C_{arom}), 127.1 (Ph-C_{arom}), 125.1, 120.0 (Fmoc-C_{arom}), 83.2 ($\underline{C}(CH_3)_3$), 76.2 (C-1), 74.5 (C-5), 71.5 (C-3), 69.7 (C-2), 67.1 (Fmoc-CH_2), 65.2 (C-4), 65.1, 61.9 (C-6), 55.9 (CHNHAsn), 49.7 (CHNHFmoc), 47.1 (Fmoc-CH), 38.3, 38.2 (Phe-CH_2, Asn-CH_2), 27.8 ($C(\underline{C}H_3)_3$), 20.9, 20.7, 20.6, 20.5 (CH_3).

$[\alpha]_D^{20}$ = – 13.6 (c = 1, $CHCl_3$). **Mass** Anal. Calcd for $C_{46}H_{53}N_3O_{15}$ $[M+Na]^+$: m/z 910.33689; HRMS found $[M+Na]^+$: m/z 910.33802. **Anal**. Calcd for $C_{49}H_{60}N_2O_{22}$ C: 62.22%, H: 6.02%, N: 4.73%; found C: 62.02%, H: 6.21%, N: 4.67%.

N^2-[N^2-[(9*H*-Fluoren-9-ylmethoxy)carbonyl]phenylalaninyl]-*N*-(2,2',3,3',4,6,6'-hepta-*O*-β-D-cellobiosyl)-L-asparagine-*tert*-butyl ester (4d)

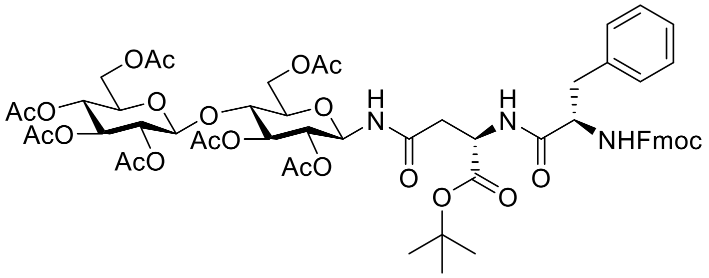

According to General Procedure D, **4d** was obtained from **3d** and Fmoc-L-Phe-OH as a colorless solid. Yield: 68% (Procedure D1)/80% (Procedure D2).

^{1}H NMR (400 MHz, $CDCl_3$): δ = 7.74 (d, *J* = 7.6 Hz, 2H, Fmoc-H_{arom}), 7.53 (dd, *J* = 17.2 Hz, *J* = 7.1 Hz, 2H, Fmoc-H_{arom}), 7.35–7.41 (m, 2H, Fmoc-H_{arom}), 7.25–7.31 (m, 4H, Fmoc-H_{arom}, Ph-H_{arom}), 7.19–7.25 (m, 3H, Ph-H_{arom}), 7.09 (d, *J* = 6.6 Hz, 1H, CHN$\underline{H}$Fmoc), 6.56 (d, *J* = 8.7 Hz, 1H, C-1-NH), 5.56 (d, *J* = 7.5 Hz, 1H, CHN$\underline{H}$Asn), 5.17–5.24 (m, 1H, H-3), 4.99–5.14 (m, 3H, H-3‘, H-4‘, H-1), 4.80–4.94 (m, 2H, H-2‘, H-2), 4.56–4.64 (m, 1H, C$\underline{H}$NHFmoc), 4.48–4.55 (m, 1H, C$\underline{H}$NHAsn), 4.42 (d, $J_{1‘,2‘}$ = 7.9 Hz, 1H, H-1‘), 4.34–4.39 (m, 1H, Fmoc-CH_2), 4.21–4.34 (m, 4H, H-6a‘, H-6a, H-6b, Fmoc-CH_2), 4.14–4.20 (m, 1H, Fmoc-CH), 3.90 (d, *J* = 12.2 Hz, 1H, H-6b‘), 3.54–3.67 (m, 3H, H-4, H-5, H5‘), 3.22 (dd, *J* = 13.7 Hz, *J* = 3.9 Hz, 1H, Asn-CH_2), 2.96 (dd, *J* = 13.3 Hz, *J* = 7.8 Hz, 1H, Asn-CH_2), 2.68–2.78 (m, 2H, Phe-CH_2), 2.07, 2.03, 2.00, 1.99, 1.97, 1.92 (s, 21H, CH_3), 1.43 (m, 9H, $C(CH_3)_3$).

^{13}C NMR (101 MHz, $CDCl_3$): δ = 171.1, 171.0, 170.8, 170.5, 170.4, 170.2, 169.4, 169.3, 169.0, 168.9 (AcC=O, ^{t}BuC=O, NHC=O), 156.0 (NHC=O), 143.8, 143.8, 141.2, 141.2, 136.4 ($C_{q,arom}$), 129.3 , 128.6 (Ph-C_{arom}), 127.7 (Fmoc-C_{arom}), 127.1 127.0 (Ph-C_{arom}, Fmoc-C_{arom}), 125.2, 119.9 (Fmoc-C_{arom}), 100.7 (C-1‘), 82.8 ($\underline{C}(CH_3)_3$), 78.0 (C-1), 76.5 (C-4), 74.9 (C-5), 72.8 (C-3‘), 72.2 (C-3), 72.0 (C-5‘), 71.5, 70.6 (C-2, C-2‘), 67.7 (C-4‘), 67.1 (Fmoc-CH_2), 61.6, 61.4 (C-6, C-6‘), 56.0 (CHNHAsn), 49.8 (CHNHFmoc), 47.1 (Fmoc-CH), 38.3 (Asn-CH_2), 38.1 (Phe-CH_2), 27.9 ($C(\underline{C}H_3)_3$), 20.8, 20.6, 20.6, 20.5, 20.5, 20.4 (CH_3).

$[\alpha]_D^{20}$ = 1.0 (c = 1, $CHCl_3$). **Mass** Anal. Calcd for $C_{58}H_{69}N_3O_{23}$ $[M+Na]^+$: m/z 1198.42141; HRMS found $[M+Na]^+$: m/z 1198.42140.

N^2-[N^2-[(9*H*-Fluoren-9-ylmethoxy)carbonyl]phenylalaninyl]-*N*-(2,2',3,3',4,6,6'-hepta-*O*-β-D-lactosyl)-L-asparagine-*tert*-butyl ester (4e)

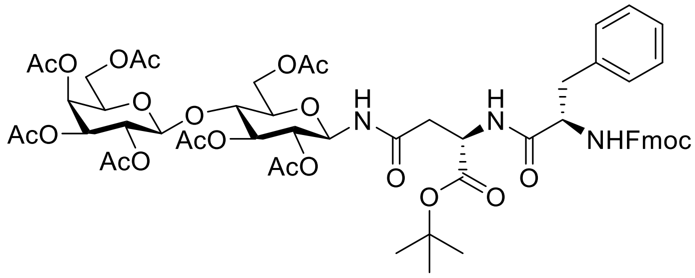

According to General Procedure D, **4e** was obtained from **3e** and Fmoc-L-Phe-OH as a colorless solid. Yield: 76% (Procedure D1)/79% (Procedure D2).

^{1}H NMR (400 MHz, $CDCl_3$): δ = 7.74 (d, *J* = 7.5 Hz, 2H, Fmoc-H_{arom}), 7.53 (dd, *J* = 15.9 Hz, *J* = 7.2 Hz 2H, Fmoc-H_{arom}), 7.35–7.41 (m, 2H, Fmoc-H_{arom}), 7.25–7.31 (m, 5H, Fmoc-H_{arom}, Ph-H_{arom}), 7.19–7.24 (m, 3H, Ph-H_{arom}), 7.10 (d, *J* = 6.7 Hz, 1H, CHN$\underline{H}$Fmoc), 6.57 (d, *J* = 8.8 Hz, 1H, C-1-NH), 5.56 (d, *J* = 7.5 Hz, 1H, CHN$\underline{H}$Asn), 5.31 (dd, $J_{4',3'}$ = 3.5 Hz, $J_{4',5'}$ = 0.6 Hz, 1H, H-4'), 5.19–5.26 (m, 1H, H-3), 5.04–5.11 (m, 2H, H-1, H-2'), 4.92 (dd, $J_{3',2'}$ = 10.4 Hz, $J_{3',4'}$ = 3.4 Hz, 1H, H-3'), 4.80–4.87 (m, 1H, H-2), 4.57–4.64 (m, 1H, C$\underline{H}$NHFmoc), 4.46–4.55 (m, 1H, C$\underline{H}$NHAsn), 4.39 (d, *J* = 7.9 Hz, 1H, H-1'), 4.21–4.38 (m, 4H, Fmoc-CH_2, H-6a, H-6b), 4.14 – 4.19 (m, 1H, Fmoc-CH), 4.00–4.05 (m, 2H, H-6a', H-6b'), 3.77–3.82 (m, 1H, H-5'), 3.63–3.69 (m, 1H, H-4), 3.58–3.63 (m, 1H, H-5), 3.22 (dd, *J* = 13.7 Hz, *J* = 4.5 Hz, 1H, Asn-CH_2), 2.96 (dd, *J* = 13.3 Hz, *J* = 8.4 Hz, 1H, Asn-CH_2), 2.67–2.80 (m, 2H, Phe-CH_2), 2.13, 2.06, 2.03, 2.02, 2.01, 1.95 (s, 21H, CH_3), 1.43 (s, 9H, C($\underline{C}H_3)_3$).

^{13}C NMR (101 MHz, $CDCl_3$): δ = 171.1, 171.0, 170.8, 170.5, 170.3, 170.1, 170.1, 169.4, 169.0, 168.9 (AcC=O, ^{t}BuC=O, NHC=O), 156.0 (NHC=O), 143.8, 141.2, 141.2, 136.4 ($C_{q,arom}$), 129.3, 128.6 (Ph-C_{arom}), 127.7 (Fmoc-C_{arom}), 127.0 (Fmoc-C_{arom}, Ph-C_{arom}), 125.2, 119.9 (Fmoc-C_{arom}), 100.9 (C-I), 82.7 ($\underline{C}(CH_3)_3$), 77.9 (C-1), 76.2 (C-4), 74.9 (C-5), 72.5 (C-3), 70.9

(C-3‘), 70.7, 70.7 (C-2, C-5‘), 69.0 (C-2‘), 67.1 (Fmoc-CH_2), 66.5 (C-4‘), 61.7 (C-6), 60.8 (C-6‘), 56.0 (CHNHAsn), 49.7 (CHNHFmoc), 47.0 (Fmoc-CH), 38.3 (Asn-CH_2), 38.0 (Phe-CH_2), 27.8 (C($\underline{C}H_3)_3$), 20.8, 20.6, 20.6, 20.6, 20.5 (CH_3).

$[\alpha]_D^{20}$ = + 3.2 (c = 1, $CHCl_3$). **Mass** Anal. Calcd for $C_{58}H_{69}N_3O_{23}$ $[M+Na]^+$: m/z 1198.42141; HRMS found $[M+Na]^+$: m/z 1198.42103. **Anal**. Calcd for $C_{58}H_{69}N_3O_{23}$ C: 59.23%, H: 5.91%, N: 3.57%; found C: 59.13%, H: 6.13%, N: 3.30%.

N^2-[N^2-[(9*H*-Fluoren-9-ylmethoxy)carbonyl]phenylalaninyl]-*N*-(2,2’,3,3’,4,6,6’-hepta-*O*-β-D-maltosyl)-L-asparagine-*tert*-butyl ester (4f)

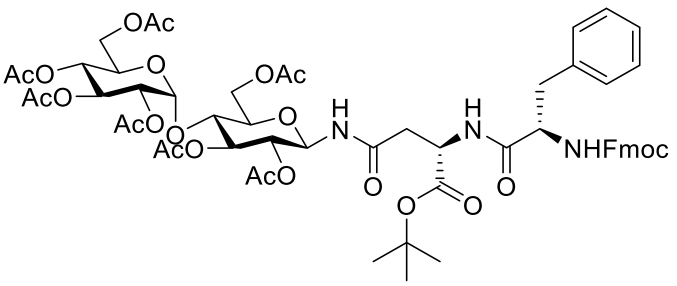

According to General Procedure D, **4f** was obtained from **3f** and Fmoc-L-Phe-OH as a colorless solid. Yield: 78% (Procedure D1)/67% (Procedure D2).

^{1}H NMR (300 MHz, $CDCl_3$): δ = 7.75 (d, *J* = 7.5 Hz, 2H, Fmoc-H_{arom}), 7.49–7.58 (m, 2H, Fmoc-H_{arom}), 7.39 (td, *J* = 7.2 Hz, *J* = 3.5 Hz, 2H, Fmoc-H_{arom}), 7.26–7.32 (m, 4H, Ph-H_{arom}, Fmoc-H_{arom}), 7.17–7.24 (m, 3H, Ph-H_{arom}), 7.11 (d, *J* = 7.4 Hz, 1H, CHN$\underline{H}$Fmoc), 6.52 (d, *J* = 9.1 Hz, 1H, C-1-NH), 5.53 (d, *J* = 8.9 Hz, 1H, CHN$\underline{H}$Asn), 5.25–5.39 (m, 3H, H-1‘, H-3‘, H-3), 5.08–5.17 (m, 1H, H-1), 5.00–5.08 (m, 1H, H-4‘), 4.84 (dd, $J_{2‘,3‘}$ = 10.5 Hz, $J_{2‘,1‘}$ = 4.0 Hz, 1H, H-2‘), 4.72–4.80 (m, 1H, H-2), 4.55–4.64 (m, 1H, C$\underline{H}$NHFmoc), 4.43 – 4.55 (m, 1H, C$\underline{H}$NHAsn), 4.28–4.43 (m, 3H, H-6a, Fmoc-CH_2), 4.14–4.28 (m, 3H, H-6b, H-6a‘, Fmoc-CH), 4.03 (dd, $J_{6b‘,6a‘}$ = 12.4 Hz, $J_{6b‘,5}$ = 2.1 Hz, 1H, H-6b‘), 3.84–3.94 (m, 2H, H-4,

H-5‘), 3.59–3.69 (m, 1H, H-5), 3.19 (dd, J = 13.8 Hz, J = 5.3 Hz, 1H, Asn-CH_2), 2.97 (dd, J = 13.9 Hz, J = 8.2 Hz, 1H, Asn-CH_2), 2.77 (dd, J = 15.6 Hz, J = 3.9 Hz, 2H, Phe-CH_2), 2.69 (dd, J = 16.0 Hz, J = 5.1 Hz, 2H, Phe-CH_2), 2.06, 2.05, 2.04, 2.02, 2.00, 1.99 , 1.95 (s, 21H, CH_3), 1.43 (s, 9H, C($\underline{C}H_3)_3$).

^{13}C NMR (75 MHz, $CDCl_3$): δ = 171.0, 170.9, 170.8, 170.6, 170.5, 170.4, 169.9, 169.8, 169.4, 169.0 (AcC=O, ^{t}BuC=O, NHC=O), 156.0 (NHC=O), 43.8, 143.8, 141.2, 136.3 ($C_{q,arom}$), 129.3, 128.6 (Ph-C_{arom}), 127.7, (Fmoc-C_{arom}), 127.0 (Ph-C_{arom}, Fmoc-C_{arom}), 125.1, 119.9 (Fmoc-C_{arom}), 95.5 (C-1‘), 82.8 ($\underline{C}(CH_3)_3$), 77.6 (C-1), 75.1 (C-3), 74.2 (C-5), 72.5 (C-5‘), 71.2 (C-2), 70.0 (C-2‘), 69.3 (C-3‘), 68.5 (C-4), 67.9 (C-4‘), 67.0 (Fmoc-CH_2), 62.4 (C-6), 61.4 (C-6‘), 56.1 (CHNHAsn), 56.0, 49.7 (CHNHFmoc), 47.1 (Fmoc-CH), 38.2 (Asn-CH_2), 38.0 (Phe-CH_2), 27.8 (C($\underline{C}H_3)_3$), 20.8, 20.6, 20.6 (CH_3).

$[\boldsymbol{\alpha}]_{\mathbf{D}}^{\mathbf{20}}$ = + 4.7 (c = 1, $CHCl_3$). **Mass** Anal. Calcd for $C_{58}H_{69}N_3O_{23}$ $[M+Na]^+$: m/z 1198.42141; HRMS found $[M+Na]^+$: m/z 1198.41999. **Anal**. Calcd for $C_{58}H_{69}N_3O_{23}$ C: 59.23%, H: 5.91%, N: 3.57%; found C: 59.28%, H: 6.26%, N: 3.20%.

N^2-[N^2-[(9*H*-Fluoren-9-ylmethoxy)carbonyl]tryptophyl]-*N*-(2,3,4,6-tetra-*O*-acetyl-β-D-glucopyranosyl)-L-asparagine-*tert*-butyl ester (5a)

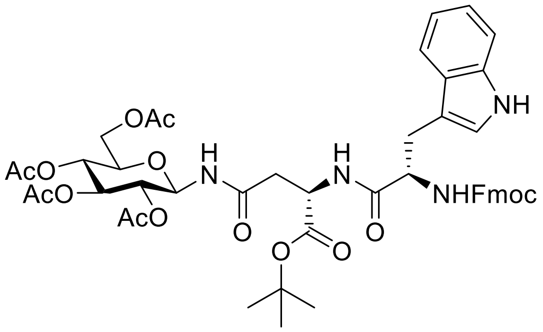

According to General Procedure D, **5a** was obtained from **3a** and Fmoc-L-Trp-OH as a colorless solid. Yield: 53% (Procedure D1)/66% (Procedure D2).

^{1}H NMR (400 MHz, $CDCl_3$): δ = 8.38 (br. s., 1H, Trp-NH), 7.74 (d, *J* = 7.6 Hz, 2H, Fmoc-H_{arom}), 7.65 (d, *J* = 7.8 Hz, 1H, Trp-H_{arom}), 7.50–7.58, 7.35–7.41 (m, 4H, H-Fmoc), 7.25–7.35 (m, 3H, Trp-H_{arom}, Fmoc-H_{arom}), 7.18–7.23, 7.10–7.15 (m, 3H, Trp-H_{arom}), 7.03 (d, *J* = 0.7 Hz, 1H, Trp-H_{arom}), 6.98 (d, *J* = 6.4 Hz, 1H, CHN<u>H</u>Asn/CHN<u>H</u>Fmoc), 6.39 (d, *J* = 6.1 Hz, 1H, C-1-NH), 5.46–5.55 (m, 1H, CHN<u>H</u>Asn/CHN<u>H</u>Fmoc), 5.20–5.29 (m, 1H, H-3), 5.07–5.14 (m, 1H, H-1), 4.97–5.04 (m, 1H, H-4), 4.86–4.94 (m, 1H, H-2), 4.51–4.61 (m, 2H, C<u>H</u>NHFmoc, C<u>H</u>NHAsn), 4.38–4.46 (m, 1H, Fmoc-CH_2), 4.31–4.37 (m, 1H, Fmoc-CH_2), 4.28 (dd, $J_{6a,6b}$ = 12.5 Hz, $J_{6a,5}$ = 4.8 Hz, 1H, H-6a), 4.15–4.22 (m, 1H, Fmoc-CH), 3.98–4.08 (m, 1H, H-6b), 3.65–3.75 (m, 1H, H-5), 3.39 (dd, *J* = 14.7 Hz, *J* = 5.7 Hz, 1H, Trp-CH_2), 3.15–3.25 (m, 1H, Trp-CH_2), 2.61–2.73 (m, 1H, Asn-CH_2), 2.42–2.55 (m, 1H, Asn-CH_2), 2.03, 2.02, 2.00, 1.99 (s, 12H, CH_3), 1.39 (s, 9H, $C(CH_3)_3$).

^{13}C NMR (101 MHz, $CDCl_3$): δ =171.3, 170.8, 170.7, 170.5, 169.9, 169.5, 169.0 (AcC=O, ^{t}BuC=O, NHC=O) , 156.0 (NHC=O), 143.9, 143.7, 141.2, 136.2 ($C_{q,arom}$), 127.7, 127.1, 125.2 (Fmoc-C_{arom}), 123.1, 122.3 (Trp-C_{arom}), 119.9 (Fmoc-C_{arom}), 119.7, 118.9, 111.4 (Trp-C_{arom}), 110.0 ($C_{q,arom}$), 82.5 (<u>C</u>$(CH_3)_3$), 78.0 (C-1), 73.7 (C-5), 72.7 (C-3), 70.4 (C-2), 68.1 (C-4), 67.1

(Fmoc-CH_2), 61.6 (C-6), 55.4 (CHNHFmoc), 49.4 (CHNHAsn), 47.1 (Fmoc-CH), 37.8 (Asn-CH_2), 27.9 (Trp-CH_2), 27.8 (C(<u>C</u>H_3)$_3$), 20.6, 20.6, 20.5 (CH_3).

$[\alpha]_D^{20}$ = + 13.1 (c = 1, $CHCl_3$). **Mass** Anal. Calcd for $C_{48}H_{54}N_4O_{15}$ $[M+Na]^+$: m/z 949.34799; HRMS found $[M+Na]^+$: m/z 949.34806. **Anal**. Calcd for $C_{48}H_{54}N_4O_{15}$ C: 62:19%, H: 5.87%, N: 6:04%; found C: 61.82%, H: 6.19%, N: 5.73%.

N^2-[N^2-[(9*H*-Fluoren-9-ylmethoxy)carbonyl]tryptophyl]-*N*-(2,3,4,6-tetra-*O*-acetyl-β-D-galactopyranosyl)-L-asparagine-*tert*-butyl ester (5b)

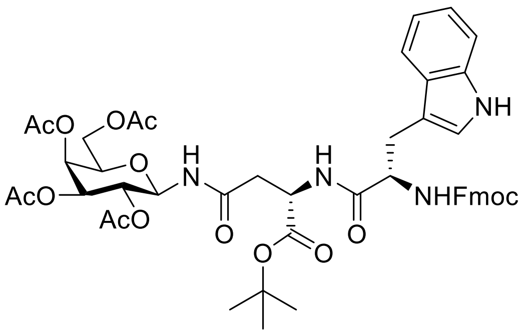

According to General Procedure D, **5b** was obtained from **3b** and Fmoc-L-Trp-OH as a white-yellow solid. Yield: 52% (Procedure D1)/75% (Procedure D2).

^{1}H NMR (300 MHz, $CDCl_3$): δ = 8.33 (br. s., 1H, Trp-NH), 7.74 (d, J = 7.5 Hz, 2H, Fmoc-H_{arom}), 7.67 (d, J = 7.6 Hz, 1H, Trp-H_{arom}), 7.47–7.58 (m, 2H, Fmoc-H_{arom}), 7.32–7.42 (m, 3H, Fmoc-H_{arom}, Trp-H_{arom}), 7.26–7.31 (m, 2H, Fmoc-H_{arom}), 7.16–7.23, 7.08–7.15 (m, 2H, Trp-H_{arom}), 7.06 (br. s., 1H, Trp-H_{arom}), 7.02 (d, J = 7.6 Hz, 1H, CHN<u>H</u>Asn), 6.44–6.56 (m, 1H, C-1-NH), 5.51 (d, J = 5.8 Hz, 1H, CHN<u>H</u>Fmoc), 5.35–5.43 (m, 1H, H-4), 5.02–5.17 (m, 3H, H-1, H-2, H-3), 4.48–4.66 (m, 2H, C<u>H</u>NHFmoc, C<u>H</u>NHAsn), 4.28–4.45 (m, 2H, Fmoc-CH_2), 4.13–4.22 (m, 2H, Fmoc-CH, H-6a), 4.01–4.13 (m, 1H, H-6b), 3.90–3.99 (ddd, 1H, H-5), 3.36

(dd, J = 14.9 Hz, J = 6.2 Hz, 1H, Trp-CH_2), 3.25 (dd, J = 14.1 Hz, J = 4.4 Hz, 1H, Trp-CH_2), 2.63–2.77 (m, 1H, Asn-CH_2), 2.48–2.62 (m, 1H, Asn-CH_2), 2.04, 2.03, 1.99, 1.97 (s, 12H, CH_3), 1.40 (s, 9H, $C(CH_3)_3$).

^{13}C NMR (75 MHz, $CDCl_3$): δ = 171.3, 171.0, 170.6, 170.4, 170.5, 170.0, 169.8, 169.1 (AcC=O, ^{t}BuC=O, NHC=O), 155.9 (NHC=O), 143.9, 143.7, 141.2, 136.2 ($C_{q,arom}$), 127.6 (Fmoc-C_{arom}), 127.5 ($C_{q,arom}$), 127.0, 125.2 (Fmoc-C_{arom}), 125.1, 123.1, 122.3(Trp-C_{arom}), 119.9 (Fmoc-C_{arom}), 119.7, 118.8, 111.4 (Trp-C_{arom}), 110.1 ($C_{q,arom}$), 82.5 ($\underline{C}(CH_3)_3$), 78.4 (C-1), 72.5 (C-5), 70.8 (C-3), 68.1, 67.9 (C-2), 67.2 (C-4), 67.0 (Fmoc-CH_2), 61.3 (C-6), 55.6 (CHNHFmoc), 49.4 (CHNHAsn), 47.1 (Fmoc-CH), 37.8 (Asn-CH_2), 28.0 (Trp-CH_2), 27.8 ($C(\underline{C}H_3)_3$), 20.7, 20.5, 20.4 (CH_3).

$[\alpha]_D^{20}$ = +17.1 (c = 1, $CHCl_3$). **Mass** Anal. Calcd for $C_{48}H_{54}N_4O_{15}$ $[M+Na]^+$: m/z 949.34799; HRMS found $[M+Na]^+$: m/z 949.34745. **Anal.** Calcd for $C_{48}H_{54}N_4O_{15}$ C: 62:19%, H: 5.87%, N: 6:04%; found C: 62.30%, H: 6.34%, N: 5.66%.

***N*²-[*N*²-[(9*H*-Fluoren-9-ylmethoxy)carbonyl]tryptophyl]-*N*-(2,3,4,6-tetra-*O*-acetyl-β-D-mannopyranosyl)-L-asparagine-*tert*-butyl ester (5c)**

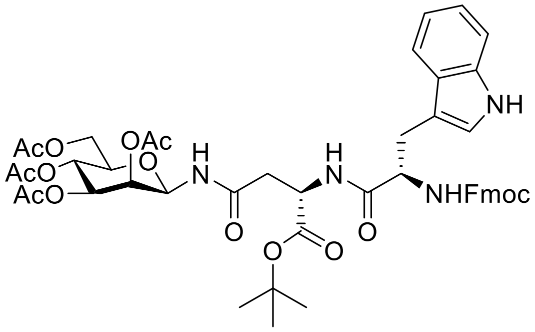

According to General Procedure D, **5c** was obtained from **3c** and Fmoc-L-Trp-OH as a white-yellow solid. Yield: 89% (Procedure D1)/62% (Procedure D2).

^{1}H NMR (400 MHz, $CDCl_3$): δ = 8.45 (br. s., 1H, Trp-NH), 7.74 (d, *J* = 7.6 Hz, 2H, Fmoc-H_{arom}), 7.63 (d, *J* = 7.6 Hz, 1H, Trp-H_{arom}), 7.53 (dd, *J* = 15.4 Hz, *J* = 7.3 Hz, 2H, Fmoc-H_{arom}), 7.36–7.41 (m, 2H, Fmoc-H_{arom}), 7.34 (d, *J* = 8.1 Hz, 1H, Trp-H_{arom}), 7.26–7.31 (m, 2H, Fmoc-H_{arom}), 7.14–7.20, 7.09–7.14 (m, 2H, H-Trp), 7.01–7.08 (m, 1H, CHN$\underline{\text{H}}$Asn, Trp-H_{arom}), 6.93 (d, *J* = 7.7 Hz, 1H, C-1-NH), 5.65 (d, *J* = 6.1 Hz, 1H, CHN$\underline{\text{H}}$Fmoc), 5.36–5.42 (m, 1H, H-1), 5.29–5.33 (m, 1H, H-2), 5.19–5.27 (m, 1H, H-4), 5.08 (dd, $J_{3,4}$ = 10.0 Hz, $J_{3,2}$ = 3.3 Hz, 1H, H-3), 4.55–4.66 (m, 1H, C$\underline{\text{H}}$NHFmoc), 4.37–4.49 (m, 2H, Fmoc-CH_2, C$\underline{\text{H}}$NHAsn), 4.34 (dd, *J* = 12.4 Hz, *J* = 5.3 Hz, 2H, Fmoc-CH_2, H-6a), 4.14–4.21 (m, 1H, Fmoc-CH), 4.06–4.11 (m, 1H, H-6b), 3.74 (ddd, *J* = 9.9 Hz, *J* = 5.1 Hz, *J* = 2.0 Hz, 1H, H-5), 3.37 (dd, *J* = 14.6 Hz, *J* = 4.7 Hz, 1H, Trp-CH_2), 3.21 (dd, *J* = 14.4 Hz, *J* = 3.9 Hz, 1H, Trp-CH_2), 2.57–2.73 (m, 2H, Asn-CH_2), 2.05, 2.03, 1.99, 1.98 (s, 12H, CH_3), 1.40 (s, 9H, $C(CH_3)_3$).

^{13}C NMR (101 MHz, $CDCl_3$): δ = 171.8, 170.7, 170.7, 170.0, 169.7, 169.4, 169.1 (AcC=O, ^{t}BuC=O, NHC=O), 156.1 (NHC=O), 143.8, 143.7, 141.2, 136.1 ($C_{q,arom}$), 127.7 (Fmoc-C_{arom}), 127.6 ($C_{q,arom}$), 127.1, 125.1 (Fmoc-C_{arom}), 123.3, 122.1 (Trp-C_{arom}), 119.9 (Fmoc-C_{arom}), 119.8, 118.7, 111.3 (Trp-C_{arom}), 109.8 ($C_{q,arom}$), 83.1 ($\underline{C}(CH_3)_3$), 77.2, 76.2 (C-1), 74.3 (C-5), 71.6 (C-2), 69.6 (C-3), 67.1 (Fmoc-CH_2), 65.2 (C-4), 62.1 (C-6), 55.5 (CHNHFmoc), 49.5 (CHNHAsn), 47.0 (Fmoc-CH), 38.3 (Asn-CH_2), 28.0 (Trp-CH_2), 27.8 ($C(\underline{C}H_3)_3$), 20.9, 20.7, 20.6, 20.6 (CH_3).

$[\alpha]_D^{20}$ = + 4.1 (c = 0.5, $CHCl_3$). **Mass** Anal. Calcd for $C_{48}H_{54}N_4O_{15}$ $[M+Na]^+$: m/z 949.34799; HRMS found $[M+Na]^+$: m/z 949.34594. **Anal.** Calcd for $C_{48}H_{54}N_4O_{15}$ C: 62:19%, H: 5.87%, N: 6:04%; found C: 62.17%, H: 6.11%, N: 5.89%.

N^2-[N^2-[(9*H*-Fluoren-9-ylmethoxy)carbonyl]tryptophyl]-*N*-(2,2',3,3',4,6,6'-hepta-*O*-β-D-cellobiosyl)-L-asparagine-*tert*-butyl ester (5d)

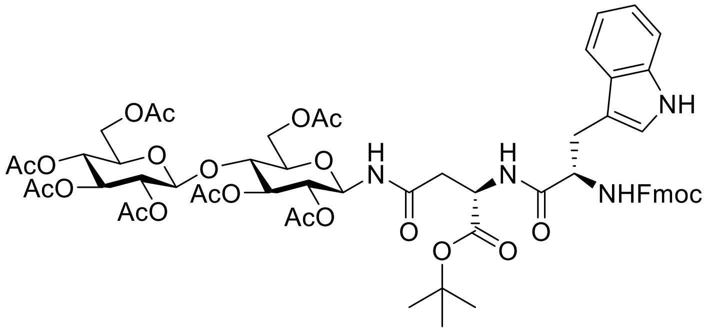

According to General Procedure D, **5d** was from **3d** and Fmoc-L-Trp-OH obtained as a white-yellow solid. Yield: 38% (Procedure D1)/59% (Procedure D2).

^{1}H NMR (400 MHz, $CDCl_3$): δ = 8.39 (br. s., 1H, Trp-NH), 7.74 (d, *J* = 7.5 Hz, 2H, Fmoc-H_{arom}), 7.65 (d, *J* = 7.5 Hz, 1H, Trp-H_{arom}), 7.49–7.57 (m, 2H, Fmoc-H_{arom}), 7.35–7.41 (m, 2H, Fmoc-H_{arom}), 7.26–7.35 (m, 3H, Trp-H_{arom}, Fmoc-H_{arom}), 7.15–7.22, 7.09–7.14 (m, 2H, Trp-H_{arom}), 7.07 (br. s., 1H, Trp-H_{arom}), 7.03 (d, *J* = 6.1 Hz, 1H, CHN<u>H</u>Asn), 6.46 (d, *J* = 6.9 Hz, 1H, C-1-NH), 5.57 (d, *J* = 5.0 Hz, 1H, CHN<u>H</u>Fmoc), 5.16–5.22 (m, 1H, H-3), 5.09–5.15 (m, 1H, H-3'), 5.01–5.08 (m, 2H, H-4', H-1), 4.90 (dd, *J* = 9.3 Hz, *J* = 8.2 Hz, 1H, H-2'), 4.79–4.87 (m, 1H, H-2), 4.57–4.63 (m, 1H, C<u>H</u>NHFmoc), 4.50–4.56 (m, 1H, C<u>H</u>NHAsn), 4.27–4.46 (m, 5H, Fmoc-CH_2, H-6a, H-6b, H-1'), 4.12–4.22 (m, 2H, Fmoc-CH, H-6a'), 3.90–3.99 (m, 1H, H-6b'), 3.56–3.68 (m, 3H, H-5', H-5, H-4), 3.36 (dd, *J* = 14.9 Hz, *J* = 6.7 Hz, 1H, Trp-CH_2), 3.17–3.30 (m, 1H, Trp-CH_2), 2.59–2.72 (m, 1H, Asn-CH_2), 2.44–2.56 (m, 1H, Asn-CH_2), 2.05, 2.01, 2.01, 2.00, 1.97, 1.96 (s, 21H, CH_3), 1.39 (s, 9H, $C(CH_3)_3$).

^{13}C NMR (101 MHz, $CDCl_3$): δ = 171.4, 171.3, 171.1, 170.9, 170.7, 170.5, 170.2, 170.0, 169.5, 169.3, 168.9 (AcC=O, ^{t}BuC=O, NHC=O), 156.0 (NHC=O), 143.7, 141.2, 141.2, 136.2 ($C_{q,arom}$), 127.7 (Fmoc-C_{arom}), 127.5 ($C_{q,arom}$), 127.1, 125.2 (Fmoc-C_{arom}), 124.2, 123.1, 122.2

(Trp-C_{arom}), 119.9 (Fmoc-C_{arom}), 119.7, 118.8 (Trp-C_{arom}), 111.4 ($C_{q,arom}$), 100.6 (C-1'), 82.6 ($\underline{C}(CH_3)_3$), 77.9 (C-1), 76.3 (C-5), 74.8 (C-5'), 72.8 (C-3), 72.2 (C-3'), 72.0 (C-4), 71.5 (C-2'), 70.6 (C-2), 67.7 (C-4'), 67.1 (Fmoc-CH_2), 61.6 (C-6), 61.5 (C-6'), 55.5 (CHNHFmoc), 49.5 (CHNHAsn), 47.1 (Fmoc-CH), 37.9 (Asn-CH_2), 28.0 (Trp-CH_2), 27.8 ($C(\underline{C}H_3)_3$), 20.8, 20.6, 20.5, 20.5, 20.4 (CH_3).

$[\alpha]_D^{20}$ = +1.1 (c = 1, $CHCl_3$). **Mass** Anal. Calcd for $C_{60}H_{70}N_4O_{23}$ $[M+Na]^+$: m/z 1237.43231; HRMS found $[M+Na]^+$: m/z 1237.43069. **Anal.** Calcd for $C_{60}H_{70}N_4O_{23}$ C: 59.30%, H: 5.81%, N: 4.61%; found C: 59.18%, H: 6.15%, N: 4.43%.

N^2-[N^2-[(9*H*-Fluoren-9-ylmethoxy)carbonyl]tryptophyl]-*N*-(2,2',3,3',4,6,6'-hepta-*O*-β-D-lactosyl)-L-asparagine-*tert*-butyl ester (5e)

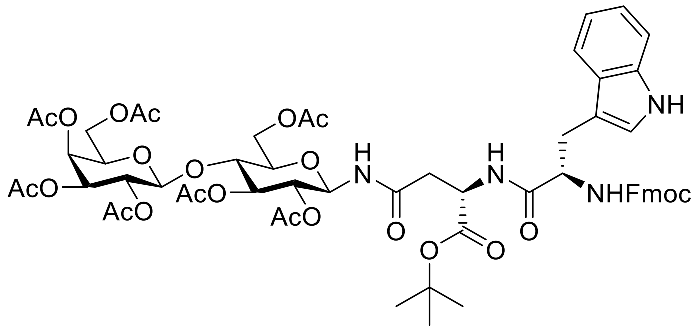

According to General Procedure D, **5e** was obtained from **3e** and Fmoc-L-Trp-OH as a white solid. Yield: 78% (Procedure D1)/66% (Procedure D2).

^{1}H NMR (400 MHz, $CDCl_3$): δ = 8.38 (br. s., 1H, Trp-NH), 7.74 (d, *J* = 7.6 Hz, 2H, Fmoc-H_{arom}), 7.65 (d, *J* = 7.3 Hz, 1H, Trp-H_{arom}), 7.49–7.58, 7.36–7.41 (m, 4H, Fmoc-H_{arom}), 7.33 (d, *J* = 8.2 Hz, 1H, Trp-H_{arom}), 7.26–7.30 (m, 2H, Fmoc-H_{arom}), 7.16–7.21, 7.09–7.14 (m, 2H, Trp-H_{arom}), 7.06 (br. s., 1H, Trp-H_{arom}), 7.02 (d, *J* = 6.0 Hz, 1H, CHN$\underline{H}$Asn), 6.42 (d, *J* = 6.6 Hz, 1H, C-1-NH), 5.56 (d, *J* = 5.4 Hz, 1H, CHN$\underline{H}$Fmoc), 5.32–5.34 (m, 1H, H-4'),

5.18–5.25 (m, 1H, H-3), 5.02–5.13 (m, 2H, H-1, H-2‘), 4.93 (dd, $J_{3‘,2‘}$ = 10.4 Hz, $J_{3‘,4‘}$ = 3.4 Hz, 1H, H-3‘), 4.79–4.87 (m, 1H, H-2), 4.57–4.63 (m, 1H, C<u>H</u>NHFmoc), 4.50–4.55 (m, 1H, C<u>H</u>NHAsn), 4.28–4.46 (m, 4H, H-6a, Fmoc-CH_2, H-1‘), 4.14–4.22 (m, 2H, Fmoc-CH, H-6b), 4.01–4.08 (m, 2H, H6a‘, H-6b‘), 3.79–3.86 (m, 1H, H-5‘), 3.65–3.71 (m, 1H, H-4), 3.59–3.65 (m, 1H, H-5), 3.36 (dd, J = 14.8 Hz, J = 6.2 Hz, 1H, Trp-CH), 3.18–3.28 (m, 1H, Trp-CH_2), 2.59–2.70 (m, 1H, Asn-CH_2), 2.43–2.55 (m, 1H, Asn-CH_2), 2.14, 2.04, 2.03, 2.02, 1.99, 1.96 (s, 21H, CH_3), 1.39 (s, 9H, $C(CH_3)_3$).

^{13}C NMR (101 MHz, $CDCl_3$): δ = 171.3, 171.0, 170.7, 170.3, 170.1, 170.1, 169.4, 169.0 (AcC=O, ^{t}BuC=O, NHC=O), 156.0 (NHC=O), 143.9, 143.7, 141.2, 136.2 ($C_{q,arom}$), 127.7 (Fmoc-C_{arom}), 127.5 ($C_{q,arom}$), 127.1, 125.2 (Fmoc-C_{arom}), 123.0, 122.2 (Trp-C_{arom}), 119.9 (Fmoc-C_{arom}), 119.7, 118.8, 111.4 (Trp-C_{arom}), 110.1 ($C_{q,arom}$), 100.9 (C-1‘), 82.6 (<u>C</u>(CH_3)$_3$), 77.9 (C-1), 76.0 (C-4), 74.8 (C-5), 72.5 (C-3), 70.9 (C-5‘), 70.7 (C-3‘, C-2), 69.0 (C-2‘), 67.1 (Fmoc-CH_2), 66.6 (C-6), 61.7 (C-6‘), 55.5 (CHNHFmoc), 49.5 (CHNHAsn), 47.1 (Fmoc-CH), 37.9 (Asn-CH_2), 28.0 (Trp-CH_2), 27.8 (C(<u>C</u>H_3)$_3$), 20.8, 20.7, 20.6, 20.6, 20.5 (CH_3).

$[\boldsymbol{\alpha}]_{\mathbf{D}}^{\mathbf{20}}$ = + 1.7 (c = 0.5, $CHCl_3$). **Mass** Anal. Calcd for $C_{60}H_{70}N_4O_{23}$ $[M+Na]^+$: m/z 1237.43231; HRMS found $[M+Na]^+$: m/z 1237.43024. **Anal.** Calcd for $C_{60}H_{70}N_4O_{23}$ C: 59.30%, H: 5.81%, N: 4.61%; found C: 59.03%, H: 5.96%, N: 4.60%.

N^2-[N^2-[(9*H*-Fluoren-9-ylmethoxy)carbonyl]tryptophyl]-*N*-(2,2’,3,3’,4,6,6’-hepta-*O*-β-D-maltosyl)-L-asparagine-*tert*-butyl ester (5f)

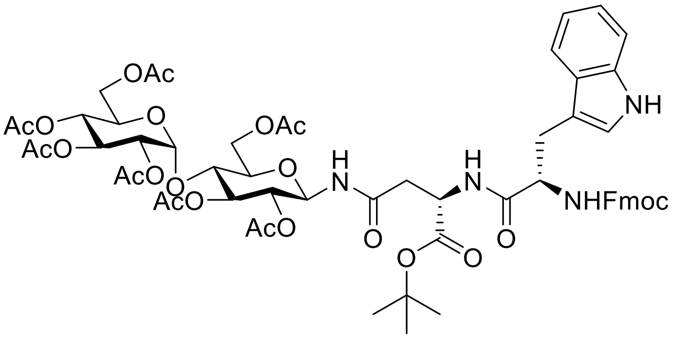

According to General Procedure D, **5f** was obtained from **3f** and Fmoc-L-Trp-OH as a white-yellow solid. Yield: 59% (Procedure D1)/73% (Procedure D2).

^{1}H NMR (400 MHz, $CDCl_3$): δ = 8.45 (br. s., 1H, Trp-NH), 7.75 (d, *J* = 7.5 Hz, 2H, Fmoc-H_{arom}), 7.65 (d, *J* = 7.5 Hz, 1H, Trp-H_{arom}), 7.50–7.58, 7.35–7.42 (m, 4H, Fmoc-H_{arom}), 7.25–7.35 (m, 3H, Fmoc-H_{arom}, Trp-H_{arom}), 7.16–7.22, 7.09–7.15 (m, 2H, Trp-H_{arom}), 7.05 (br. s., 1H, Trp-H_{arom}), 7.02 (d, *J* = 6.6 Hz, 1H, CHN<u>H</u>Asn), 6.36 (d, *J* = 7.8 Hz, 1H, C-1-NH), 5.53 (d, *J* = 6.2 Hz, 1H, CHN<u>H</u>Fmoc), 5.32–5.39 (m, 2H, H-1‘, H-3‘), 5.26–5.32 (m, 1H, H-3), 5.03–5.14 (m, 2H, H-1, H-4‘), 4.84 (dd, $J_{2‘,3‘}$ = 10.6 Hz, $J_{2‘,1‘}$ = 4.0 Hz, 1H, H-2‘), 4.71–4.79 (m, 1H, H-2), 4.50–4.62 (m, 2H, C<u>H</u>NHFmoc, C<u>H</u>NHAsn), 4.30–4.46 (m, 3H, H-6a, Fmoc-CH_2), 4.16–4.27 (m, 3H, H-6b, H-6a‘, Fmoc-CH), 4.06 (dd, $J_{6b‘,6a‘}$ = 12.6 Hz, $J_{6b‘,5‘}$ = 1.8 Hz, 1H, H-6b‘), 3.86–3.95 (m, 2H, H-4, H-5‘), 3.62–3.70 (m, 1H, H-5), 3.36 (dd, *J* = 14.9 Hz, *J* = 6.2 Hz, 1H, Trp-CH_2), 3.18 – 3.28 (m, 1H, Trp-CH_2), 2.59–2.71, 2.42–2.56 (m, 2H, Asn-CH_2), 2.07, 2.05, 2.04, 2.03, 2.02, 2.00, 1.99 (s, 21H, CH_3), 1.40 (s, 9H, $C(CH_3)_3$).

^{13}C NMR (101 MHz, $CDCl_3$): δ = 171.4, 170.8, 170.6, 169.9, 169.8, 169.4, 169.0 (AcC=O, ^{t}BuC=O, NHC=O), 156.0 (NHC=O), 143.9, 143.7, 141.3, 141.2, 136.2 ($C_{q,arom}$), 127.7 (Fmoc-C_{arom}), 127.6 ($C_{q,arom}$), 127.1 (Trp-C_{arom}), 125.2 (Fmoc-C_{arom}), 123.0, 122.2 (Trp-C_{arom}), 119.9 (Fmoc-C_{arom}), 119.7, 118.8, 111.4 (Trp-C_{arom}), 110.0 ($C_{q,arom}$), 95.5 (C-1‘), 82.6

(<u>C</u>(CH$_3$)$_3$), 77.6 (C-1), 75.1 (C-3), 74.2 (C-5), 72.5 (C-5‘), 71.2 (C-2), 69.9 (C-2‘), 69.3 (C-3‘), 68.5 (C-4), 68.0 (C-4‘), 67.1 (Fmoc-CH$_2$), 62.5 (C-6), 61.4 (C-6‘), 55.5 (CHNHAsn), 49.4 (CHNHFmoc), 47.1 (Fmoc-CH), 37.8 (Asn-CH$_2$), 27.9 (Trp-CH$_2$), 27.8 (C(<u>C</u>H$_3$)$_3$), 20.8, 20.8, 20.7, 20.6, 20.5 (CH$_3$).

$[\alpha]_D^{20}$ = +42.1 (c = 1, CHCl$_3$). **Mass** Anal. Calcd for $C_{60}H_{70}N_4O_{23}$ [M+Na]$^+$: m/z 1237.43231; HRMS found [M+Na]$^+$: m/z 1237.43272. **Anal.** Calcd for $C_{60}H_{70}N_4O_{23}$ C: 59.30%, H: 5.81%, N: 4.61%; found C: 59.11%, H: 6.00%, N: 4.58%.

***N*²-[*N*²[*N*²-[(9*H*-Fluoren-9-ylmethoxy)carbonyl]alaninyl]phenylalaninyl]-*N*-(2,3,4,6-tetra-*O*-acetyl-β-D-glucopyranosyl)-L-asparagine-*tert*-butyl ester (6a)**

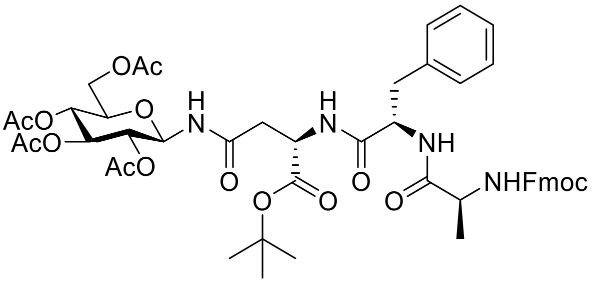

According to General Procedure D, **6a** was obtained from **4a** and Fmoc-L-Ala-OH as a colorless solid. Yield: 82% (Procedure D1)/75% (Procedure D2).

¹H NMR (400 MHz, CDCl$_3$): δ = 7.76 (d, *J* = 7.5 Hz, 2H, Fmoc-H$_{arom}$), 7.55–7.63, 7.34–7.45, 7.27–7.33 (m, 6H, Fmoc-H$_{arom}$), 7.21–7.23 (m, 2H, Ph-H$_{arom}$), 7.12–7.19 (m, 3H, Ph-H$_{arom}$), 6.99 (d, *J* = 7.7 Hz, 1H, CHN<u>H</u>Phe), 6.89 (d, *J* = 8.7 Hz, 1H, C-1-NH), 6.63 (d, *J* = 6.6 Hz, 1H, CHN<u>H</u>Asn), 5.50 (d, *J* = 6.4 Hz, 1H, CHN<u>H</u>Fmoc), 5.26 (dd, *J* = 16.6 Hz, *J* = 6.6 Hz, 1H, H-3), 5.13–5.18 (m, 1H, H-1), 4.94–5.03 (m, 2H, H-4, H-2), 4.58–4.68 (m, 2H, C<u>H</u>NHPhe, C<u>H</u>NHAsn), 4.28–4.42 (m, 3H, Fmoc-CH$_2$, H-6b), 4.15–4.24 (m, 2H, C<u>H</u>NHFmoc, Fmoc-CH), 3.98 (dd, *J* = 12.3 Hz, *J* = 1.8 Hz, 1H, H-6a), 3.70–3.76 (m, 1H, H-5), 3.22 (dd, *J* = 14.0 Hz, *J* = 5.6 Hz, 1H, Asn- CH$_2$), 2.97 (dd, *J* = 13.9 Hz, *J* = 8.1 Hz, 1H, Asn-CH$_2$),

2.66–2.76 (m., 2H, Phe-CH_2), 2.01–2.06 (m, 9H, CH_3), 1.94 (s, 3H, CH_3), 1.44 (s, 9H, $C(CH_3)_3$), 1.33 (d, J = 6.6 Hz, 3H, Ala-CH_3).

^{13}C NMR (101 MHz, $CDCl_3$): δ = 172.2, 171.1, 170.9, 170.7, 170.4, 169.9, 169.5, 168.9 (AcC=O, ^{t}BuC=O, NHC=O), 155.9 (NHC=O), 143.8, 141.3, 136.1 ($C_{q,arom}$), 129.2, 128.6 (Ph-C_{arom}), 127.7 (Ph-C_{arom}, Fmoc-C_{arom}), 127.1, 125.1, 120.0 (Fmoc-C_{arom}), 82.7 ($\underline{C}$(CH3)3), 78.0 (C-1), 73.8 (C-5), 72.9 (C-3), 70.3, 68.2 (C-4, C-2), 67.1 (Fmoc-CH_2), 61.6 (C-6), 54.5 (CHNHAsn),

50.4 (CHNHFmoc), 49.7 (CHNHPhe), 47.1 (Fmoc-CH), 38.1 (Phe-CH_2), 37.7 (Asn-CH_2), 27.9 ($C(\underline{C}H_3)_3$), 20.8, 20.7, 20.6, 20.5 (CH_3), 18.7 (Ala-CH_3).

$[\boldsymbol{\alpha}]_{\mathbf{D}}^{\mathbf{20}}$ = + 5.2 (c = 1, $CHCl_3$). **Mass** Anal. Calcd for $C_{49}H_{58}N_4O_{16}$ $[M+Na]^+$: m/z 981.37406; HRMS found $[M+Na]^+$: m/z 981.37274. **Anal.** Calcd for $C_{49}H_{58}N_4O_{16}$ C: 61.37%, H: 6.10%, N: 5.84%; found 61.31%, H: 6.34%, N: 5.46%.

***N*²-[*N*²[*N*²-[(9*H*-Fluoren-9-ylmethoxy)carbonyl]alaninyl]phenylalaninyl]-*N*-(2,3,4,6-tetra-*O*-acetyl-β-D-galactopyranosyl)-L-asparagine-*tert*-butyl ester (6b)**

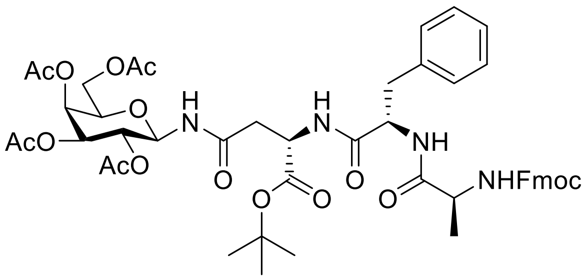

According to General Procedure D, **6b** was obtained from **4b** and Fmoc-L-Ala-OH as a colorless solid. Yield: 76% (Procedure D1)/72% (Procedure D2).

^{1}H NMR (300 MHz, $CDCl_3$): δ = 7.75 (d, J = 7.5 Hz, 2H, Fmoc-H_{arom}), 7.54–7.62, 7.35–7.43, 7.27–7.34 (m, 6H, Fmoc-H_{arom}), 7.12–7.24 (m, 5H, Ph-H_{arom}), 7.03 (d, J = 7.6 Hz, 1H,

CHNHAsn/CHNHPhe), 6.90 (d, *J* = 7.2 Hz, 1H, C-1-NH), 6.65 (d, *J* = 7.1 Hz, 1H, CHNHAsn/CHNHPhe), 5.51 (d, *J* = 6.0 Hz, 1H, CHNHFmoc), 5.36 (d, *J* = 3.0 Hz, 1H, H-3), 5.10–5.21 (m, 2H, H-4, H-1), 5.02–5.09 (m, 1H, H-2), 4.57–4.69 (m, 2H, CHNHAsn, CHNHPhe),

4.41 (dd, *J* = 10.3 Hz, *J* = 7.3 Hz, 1H, Fmoc-CH_2), 4.23–4.33 (m, 2H, Fmoc-CH_2, H-6a), 4.12–4.23 (m, 2H, CHNHFmoc, Fmoc-CH), 3.86–4.00 (m, 2H, H-5, H-6b), 3.26 (dd, *J* = 14.0 Hz, *J* = 5.1 Hz, 1H, Asn-CH_2), 2.98 (dd, *J* = 14.0 Hz, *J* = 8.7 Hz, 1H, Asn-CH_2), 2.69–2.77 (m, 2H, Phe-CH_2), 2.06, 2.05, 1.91 (s, 12H, CH_3), 1.45 (s, 9H, $C(CH_3)_3$), 1.34 (d, *J* = 6.6 Hz, 3H, Ala-CH_3).

^{13}C NMR (75 MHz, $CDCl_3$): δ = 172.2 , 171.0, 170.9, 170.7, 170.5, 170.0, 169.8, 169.0 (AcC=O, ^{t}BuC=O, NHC=O), 155.9 (NHC=O), 143.8, 143.8, 141.3, 136.3 ($C_{q,arom}$), 129.2, 128.6 (Ph-C_{arom}), 127.7 (Ph-C_{arom}, Fmoc-C_{arom}), 127.1, 125.1, 120.0 (Fmoc-C_{arom}), 82.7 ($C(CH_3)_3$), 78.4 (C-1), 72.6 (C-5), 71.0 (C-2), 67.9 (C-4), 67.2 (C-3), 67.0 (Fmoc-CH_2), 61.3 (C-6), 54.8 (CHNHAsn), 50.5 (CHNHFmoc), 49.8 (CHNHPhe), 47.1 (Fmoc-CH), 38.0 (Asn-CH_2), 37.7 (Phe-CH_2), 27.9 ($C(CH_3)_3$), 20.8, 20.5, 20.2 (CH_3), 18.8 (Ala-CH_3).

$[\boldsymbol{\alpha}]_{\mathbf{D}}^{\mathbf{20}}$ = +32.9 (c = 1, $CHCl_3$). **Mass** Anal. Calcd for $C_{49}H_{58}N_4O_{16}$ $[M+Na]^+$: m/z 981.37400; HRMS found $[M+Na]^+$: m/z 981.37402. **Anal.** Calcd for $C_{49}H_{58}N_4O_{16}$ C: 61.37%, H: 6.10%, N: 5.84%; found C: 61.27%, H: 6.22%, N: 5.78%.

***N*²-[*N*²[*N*²-[(9*H*-Fluoren-9-ylmethoxy)carbonyl]alaninyl]phenylalaninyl]-*N*-(2,3,4,6-tetra-*O*-acetyl-β-D-mannopyranosyl)-L-asparagine-*tert*-butyl ester (6c)**

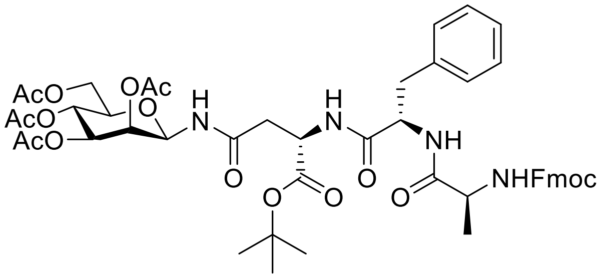

According to General Procedure D, **6c** was obtained from **4c** and Fmoc-L-Ala-OH as a colorless solid. Yield: 64% (Procedure D1)/60% (Procedure D2).

¹H NMR (600 MHz, $CDCl_3$): δ = 7.75 (d, *J* = 7.7 Hz, 2H, Fmoc-H_{arom}), 7.57 (d, *J* = 6.8 Hz, 2H, Fmoc-H_{arom}), 7.36–7.40 (m, 2H, Fmoc-H_{arom}), 7.27–7.35 (m, 4H, Fmoc-H_{arom}, CHN$\underline{H}$Phe, C-1-NH), 7.17–7.22, 7.10–7.14 (m, 5H, Ph-H_{arom}), 7.01 (d, *J* = 6.1 Hz, 1H, CHN$\underline{H}$Asn), 5.46–5.51 (m, 1H, H-1), 5.40 (d, *J* = 6.4 Hz, 1H, CHN$\underline{H}$Fmoc), 5.34–5.37 (m, 1H, H-2), 5.21–5.26 (m, 1H, H-4), 5.14 (dd, $J_{3,4}$ = 10.1 Hz, $J_{3,2}$ = 3.3 Hz, 1H, H-3), 4.73–4.79 (m, 1H, C$\underline{H}$NHAsn), 4.50–4.55 (m, 1H, C$\underline{H}$NHPhe), 4.35–4.44 (m, 2H, H-6a, Fmoc-CH_2), 4.26–4.33 (m, 1H, Fmoc-CH_2), 4.18–4.24 (m, 1H, C$\underline{H}$NHFmoc), 4.14–4.18 (m, 1H, Fmoc-CH), 4.06–4.10 (m, 1H, H-6b), 3.78 (ddd, $J_{5,4}$ = 10.1 Hz, $J_{5,6}$ = 5.1 Hz, $J_{5,6}$ = 2.0 Hz, 1H, H-5), 3.21 (dd, *J* = 14.1 Hz, *J* = 5.0 Hz, 1H, Asn-CH_2), 2.97 (dd, *J* = 14.0 Hz, *J* = 8.9 Hz, 1H, Asn-CH_2), 2.74 (dd, *J* = 15.6 Hz, *J* = 2.6 Hz, 1H, Phe-CH_2), 2.64–2.78 (m, 2H, Phe-CH_2), 2.13, 2.02, 1.97, 1.93 (s, 12H, CH_3), 1.42 (s, 9H, $C(CH_3)_3$), 1.28 (d, *J* = 5.9 Hz, 3H, Ala-CH_3).

¹³C NMR (151 MHz, $CDCl_3$): δ = 172.4, 171.1, 170.9, 170.5, 169.9, 169.6, 169.5, 169.1 (AcC=O, ^{t}BuC=O, NHC=O), 155.8 (NHC=O) , 143.7, 143.7, 141.2, 136.2, 130.6 ($C_{q,arom}$), 129.2, 128.5 (Ph-C_{arom}), 127.7, 127.1 (Fmoc-C_{arom}), 126.9 (Ph-C_{arom}), 125.0, 119.9 (Fmoc-C_{arom}), 82.8 ($\underline{C}(CH_3)_3$), 76.4 (C-1), 74.5 (C-5), 71.5 (C-3), 69.7 (C-2), 67.0 (Fmoc-CH_2), 65.2 (C-4), 61.8 (C-6), 54.1 (CHNHAsn), 50.4 (CHNHFmoc), 49.4 (CHNHPhe), 47.0

(Fmoc-CH), 37.9 (Asn-CH_2), 37.7 (Phe-CH_2), 27.8 (C($\underline{C}H_3)_3$), 20.8, 20.6, 20.6, 20.5 (CH_3), 18.4 (Ala-CH_3).

$[\alpha]_D^{20}$ = – 17.4 (c = 1, $CHCl_3$). **Mass** Anal. Calcd for $C_{49}H_{58}N_4O_{16}$ $[M+Na]^+$: m/z 981.37400; HRMS found $[M+Na]^+$: m/z 981.37404. **Anal.** Calcd for $C_{49}H_{58}N_4O_{16}$ C: 61.37%, H: 6.10%, N: 5.84%; found C: 61.57%, H: 6.49%, N: 5.47%.

***N*^{2}-[*N*^{2}[*N*^{2}-[(9*H*-Fluoren-9-ylmethoxy)carbonyl]alaninyl]phenylalaninyl]-*N*-(2,2',3,3', 4,6,6'-hepta-*O*-acetyl-β-D-cellobiosyl)-L-asparagine-*tert*-butyl ester (6d)**

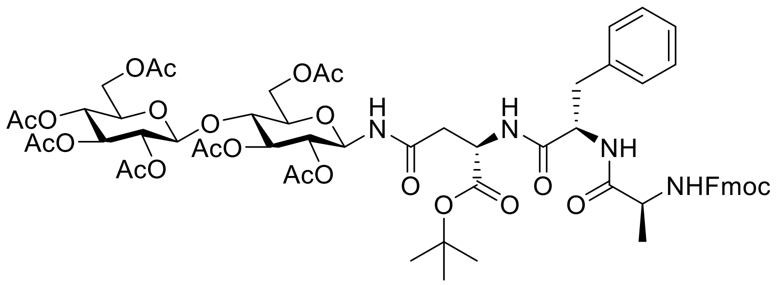

According to General Procedure D, **6d** was obtained from **4d** and Fmoc-L-Ala-OH as a colorless solid. Yield: 75% (Procedure D1)/59% (Procedure D2).

^{1}H NMR (700 MHz, $CDCl_3$): δ = 7.74 (d, *J* = 7.5 Hz, 2H, Fmoc-H_{arom}), 7.55–7.60 (m, 2H, Fmoc-H_{arom}), 7.37 (td, *J* = 7.4 Hz, *J* = 3.1 Hz, 2H, Fmoc-H_{arom}), 7.26–7.30 (m, 2H, Fmoc-H_{arom}), 7.18–7.22, 7.15–7.18 (m, 4H, Ph-H_{arom}), 7.10–7.15 (m, 2H, Ph-H_{arom}, CHN$\underline{H}$Phe), 6.98 (d, *J* = 8.8 Hz, 1H, C-1-NH), 6.93 (d, *J* = 6.2 Hz, 1H, CHN$\underline{H}$Asn), 5.66 (d, *J* = 7.1 Hz, 1H, CHN$\underline{H}$Fmoc), 5.17 – 5.21 (m, 1H, H-3), 5.07–5.12 (m, 2H, H-3‘, H-1), 5.01–5.05 (m, 1H, H-4‘), 4.81–4.93 (m, 2H, H-2‘, H-2), 4.66–4.71 (m, 1H, C$\underline{H}$NHAsn), 4.54–4.59 (m, 1H, C$\underline{H}$NHPhe), 4.43 (d, *J* = 8.0 Hz, 1H, H-1‘), 4.35–4.40 (m, 1H, Fmoc-CH_2), 4.32–4.34 (m, 1H, H-6a), 4.30 (dd, $J_{6a',6b}$ = 12.5 Hz, $J_{6a',5}$ = 4.3 Hz, 1H, H-6a‘), 4.25–4.28 (m, 1H, Fmoc-CH_2), 4.23 (dd, *J* = 12.0 Hz, *J* = 5.4 Hz, 2H, H-6b, C$\underline{H}$NHFmoc), 4.15–4.18 (m, 1H, Fmoc-CH), 3.91–

3.95 (m, 1H, H-6b‘), 3.63–3.67 (m, 1H, H-4), 3.60–3.63 (m, 1H, H-5), 3.55–3.59 (m, 1H, H-5‘), 3.18 (dd, *J* = 14.0 Hz, *J* = 5.4 Hz, 1H, Asn-CH_2), 2.93 (dd, *J* = 13.9 Hz, *J* = 8.3 Hz, 1H, Asn-CH_2), 2.66–2.73 (m, 2H, Phe-CH_2), 2.06, 2.01, 2.00, 1.99, 1.97, 1.95, 1.92 (s, 21H, CH_3), 1.42 (m, 9H, $C(CH_3)_3$), 1.31 (d, *J* = 6.5 Hz, 3H, Ala-CH_3).

^{13}C NMR (176 MHz, $CDCl_3$): δ = 172.3, 170.8, 170.7, 170.6, 170.5, 170.4, 170.2, 169.5, 169.2, 168.9, 168.9 (AcC=O, ^{t}BuC=O, NHC=O), 155.8 (NHC=O), 143.7, 143.7, 141.2, 136.2 ($C_{q,arom}$), 129.2, 128.5 (Ph-C_{arom}), 127.7, 127.7, 127.0 (Fmoc-C_{arom}), 126.9 (Ph-C_{arom}), 125.1, 119.9 (Fmoc-C_{arom}), 100.6 (C-1‘), 82.6 ($\underline{C}(CH_3)_3$), 77.8 (C-1), 76.4 (C-4), 74.8 (C-5), 72.8 (C-3‘), 72.4 (C-3), 71.8 (C-5‘), 71.4 (C-2‘), 70.5 (C-2), 67.6 (C-4‘), 67.0 (Fmoc-CH_2), 61.7 (C-6), 61.4 (C-6‘), 54.4 (CHNHAsn), 50.3 (CHNHFmoc), 49.7 (CHNHPhe), 47.0 (Fmoc-CH), 38.0 (Phe-CH_2), 37.8 (Asn-CH_2), 27.8 ($C(\underline{C}H_3)_3$), 20.8, 20.6, 20.5, 20.5, 20.4, 20.4, 20.4 (CH_3), 18.7 (Ala-CH_3).

$[\alpha]_D^{20}$ = – 2.0 (c = 0.5, $CHCl_3$). **Mass** Anal. Calcd for $C_{61}H_{74}N_4O_{24}$ $[M+Na]^+$: m/z 1269.45852; HRMS found $[M+Na]^+$: m/z 1269.45754. **Anal.** Calcd for $C_{61}H_{74}N_4O_{24}$ C: 58.68%, H: 5.98%, N: 4.49%; found C: 58.68%, H: 6.22%, N: 4.23%.

N^2-[N^2[N^2-[(9*H*-Fluoren-9-ylmethoxy)carbonyl]alaninyl]phenylalaninyl]-*N*-(2,2',3,3',4,6,6'-hepta-*O*-acetyl-β-D-lactosyl)-L-asparagine-*tert*-butyl ester (6e)

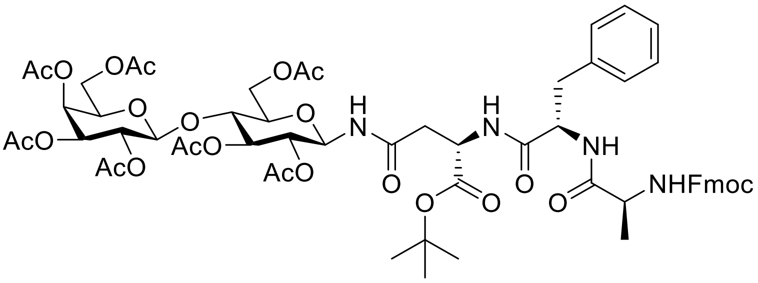

According to General Procedure D, **6e** was obtained from **4e** and Fmoc-L-Ala-OH as a colorless solid. Yield: 50% (Procedure D1)/70% (Procedure D2).

^{1}H NMR (400 MHz, $CDCl_3$): δ = 7.75 (d, *J* = 7.6 Hz, 2H, Fmoc-H_{arom}), 7.55–7.61, 7.35–7.41, 7.25–7.32 (m, 6H, Fmoc-H_{arom}), 7.12–7.24 (m, 5H, Ph-H_{arom}), 7.10 (d, *J* = 7.6 Hz, 1H, CHNHPhe), 6.95 (d, *J* = 8.9 Hz, 1H, C-1-NH), 6.87 (d, *J* = 6.6 Hz, 1H, CHNHAsn), 5.61 (d, *J* = 7.0 Hz, 1H, CHNHFmoc), 5.30 (dd, $J_{4',3'}$ = 3.4 Hz, $J_{4',5'}$ = 0.7 Hz, 1H, H-4'), 5.15–5.24 (m, 1H, H-3), 5.04–5.13 (m, 2H, H-1,H-2'), 4.85–4.94 (m, 2H, H-2, H-3'), 4.65–4.72 (m, 1H, CHNHAsn), 4.54–4.60 (m, 1H, CHNHPhe), 4.41 (d, $J_{1',2'}$ = 7.9 Hz, 1H, H-1'), 4.32–4.39 (m, 1H, Fmoc-CH_2), 4.13–4.31 (m, 5H, H-6a, H-6b, Fmoc-CH_2, Fmoc-CH, CHNHFmoc), 3.97–4.07 (m, 2H, H-6a', H-6b'), 3.76–3.82 (m, 1H, H-5'), 3.60–3.71 (m, 2H, H-4, H-5), 3.20 (dd, *J* = 13.9 Hz, *J* = 5.4 Hz, 1H, Asn-CH_2), 2.94 (dd, *J* = 13.9 Hz, *J* = 8.4 Hz, 1H, Asn-CH_2), 2.64–2.75 (m, 2H, Phe-CH_2), 2.12, 2.07, 2.01, 2.01, 2.00, 1.96, 1.94 (s, 21H, CH_3), 1.43 (s, 9H, $C(CH_3)_3$), 1.32 (d, *J* = 6.5 Hz, 3H, Ala-CH_3).

^{13}C NMR (101 MHz, $CDCl_3$): δ = 172.3, 170.9, 170.8, 170.6, 170.5, 170.2, 170.1, 170.0, 169.4, 168.9, 168.9 (AcC=O, ^{t}BuC=O, NHC=O), 155.8 (NHC=O), 143.8, 143.7, 141.2, 136.2 ($C_{q,arom}$), 129.2, 128.5 (Ph-C_{arom}), 127.7 (Ph-C_{arom}), 127.0 (Fmoc-C_{arom}), 126.9 (Ph-C_{arom}), 125.1, 120.0 (Fmoc-C_{arom}), 100.9 (C-1'), 82.6 ($C(CH_3)_3$), 77.8 (C-1), 76.2 (C-4), 74.8 (C-5), 72.7 (C-3), 70.9 (C-3'/C-5'), 70.6 (C-2), 70.6 (C-3'/C-5'), 69.0 (C-2'), 67.0 (Fmoc-CH_2), 66.5

(C-5‘), 61.8 (C-6), 60.7 (C-6‘), 54.4 (CHNHAsn), 50.4 (CHNHFmoc), 49.8 (CHNHPhe), 47.0 (Fmoc-CH), 38.0 (Phe-CH_2), 37.8 (Asn-CH_2), 27.8 (C(<u>C</u>H_3)$_3$), 20.9, 20.7, 20.6, 20.6, 20.5, 20.4 (CH_3), 18.7 (Ala-CH_3).

$[\alpha]_D^{20}$ = + 0.5 (c = 0.5, $CHCl_3$). **Mass** Anal. Calcd for $C_{61}H_{74}N_4O_{24}$ $[M+Na]^+$: m/z 1269.45852; HRMS found $[M+Na]^+$: m/z 1269.45731. **Anal.** Calcd for $C_{61}H_{74}N_4O_{24}$ C: 58.68%, H: 5.98%, N: 4.49%; found C: 58.62%, H: 6.07%, N: 4.40%.

N²**-[*N*²[*N*²-[(9*H*-Fluoren-9-ylmethoxy)carbonyl]alaninyl]phenylalaninyl]-*N*-(2,2',3,3', 4,6,6'-hepta-*O*-acetyl-β-D-maltosyl)-L-asparagine-*tert*-butyl ester (6f)**

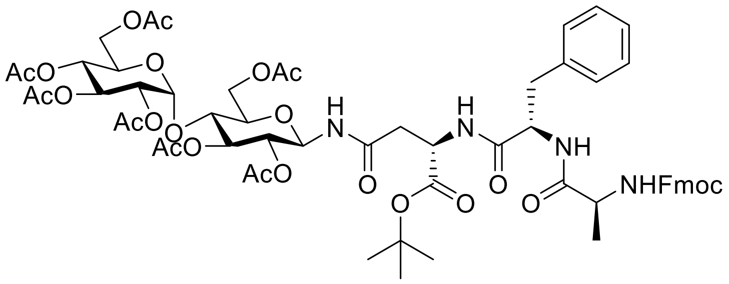

According to General Procedure D, **6f** was obtained from **4f** and Fmoc-L-Ala-OH as a white-yellow solid. Yield: 50% (Procedure D1)/78% (Procedure D2).

¹H NMR (400 MHz, $CDCl_3$): δ = 7.76 (d, *J* = 7.5 Hz, 2H, Fmoc-H_{arom}), 7.54–7.63, 7.36–7.43, 7.27–7.33 (m, 6H, Fmoc-H_{arom}), 7.12–7.24 (m, 6H, Ph-H_{arom}), 7.04 (d, *J* = 7.5 Hz, 1H, CHN<u>H</u>Phe), 6.80 (d, *J* = 9.3 Hz, 1H, C-1-NH), 6.76 (d, *J* = 7.1 Hz, 1H, CHN<u>H</u>Asn), 5.56 (d, *J* = 6.8 Hz, 1H, CHN<u>H</u>Fmoc), 5.31–5.39 (m, 3H, H-1‘, H-3‘), 5.23–5.31 (m, 1H, H-3), 5.09–5.17 (m, 1H, H-1), 5.00–5.08 (m, 1H, H-4‘), 4.77–4.87 (m, 2H, H-2, H-2‘), 4.63–4.71 (m, 1H, C<u>H</u>NHAsn), 4.54–4.61 (m, 1H, C<u>H</u>NHPhe), 4.33–4.43 (m, 2H, Fmoc-CH_2, H-6a), 4.24–4.32 (m, 2H, Fmoc-CH_2, H-6b), 4.14–4.24 (m, 4H, H-6a‘, C<u>H</u>NHFmoc, Fmoc-CH), 4.01–4.08 (m, 1H, H-6b‘), 3.85–3.94 (m, 2H, H-4, H-5‘), 3.61–3.70 (m, 1H, H-5), 3.21 (dd, *J* = 14.1 Hz, *J* =

5.4 Hz, 1H, Asn-CH_2), 2.94 (dd, *J* = 13.9 Hz, *J* = 8.4 Hz, 1H, Asn-CH_2), 2.63–2.77 (m, 2H, Phe-CH_2), 1.91–2.11 (s, 21H, CH_3), 1.44 (s, 9H, $C(CH_3)_3$), 1.33 (d, *J* = 6.4 Hz, 3H, Ala-CH_3).

^{13}C NMR (101 MHz, $CDCl_3$): δ = 172.3, 170.9, 170.8, 170.6, 170.5, 170.5, 170.4, 169.8, 169.4, 168.9 (AcC=O, ^{t}BuC=O, NHC=O), 155.9 (NHC=O), 143.8, 141.2, 136.2 ($C_{q,arom}$), 129.2, 128.5 (Ph-C_{arom}), 127.7 (Fmoc-C_{arom}), 127.0 (Ph-C_{arom}, Fmoc-C_{arom}), 125.1, 120.0 (Fmoc-C_{arom}), 95.5 (C-1‘), 82.7 ($\underline{C}(CH_3)_3$), 77.6 (C-1), 75.3 (C-3), 74.1 (C-5), 72.4 (C-5‘), 71.0 (C-2), 69.9 (C-2‘), 69.3 (C-3‘), 68.5 (C-4), 67.9 (C-4‘), 67.0 (Fmoc-CH_2), 62.4 (C-6), 61.3 (C-6‘), 54.4 (CHNHAsn), 50.4 (CHNHFmoc), 49.7 (CHNHPhe), 47.1 (Fmoc-CH), 38.0 (Phe-CH_2), 37.7 (Asn-CH_2), 27.8 ($C(\underline{C}H_3)_3$), 20.9, 20.8, 20.6, 20.6 (CH_3), 18.7 (Ala-CH_3).

$[\alpha]_D^{20}$ = + 38.0 (c = 1, $CHCl_3$). **Mass** Anal. Calcd for $C_{61}H_{74}N_4O_{24}$ $[M+Na]^+$: m/z 1269.45852, HRMS found $[M+Na]^+$: m/z 1269.45675. **Anal.** Calcd for $C_{61}H_{74}N_4O_{24}$ C: 58.68%, H: 5.98%, N: 4.49%; found C: 58.37%, H: 6.10%, N: 4.20%.

***N*²-[*N*²[*N*²-[(9*H*-Fluoren-9-ylmethoxy)carbonyl]alaninyl]tryptophyl]-*N*-(2,3,4,6-tetra-*O*-acetyl-β-D-glucopyranosyl)-L-asparagine-*tert*-butyl ester (7a)**

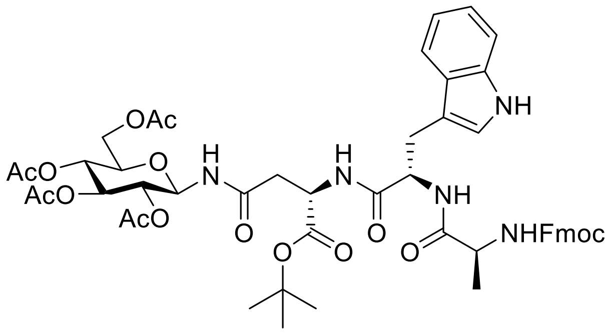

According to General Procedure D, **7a** was obtained from **5a** and Fmoc-L-Ala-OH as a white-yellow solid. Yield: 60% (Procedure D1)/68% (Procedure D2).

^{1}H NMR (400 MHz, $CDCl_3$): δ = 8.27 (br. s., 1H, Trp-NH), 7.76 (d, *J* = 7.6 Hz, 2H, Fmoc-H_{arom}), 7.54–7.63 (m, 3H, Trp-H_{arom}, Fmoc-H_{arom}), 7.36–7.43, 7.27–7.33 (m, 4H, Fmoc-H_{arom}), 7.23 (s, 1H, Trp-H_{arom}), 7.09–7.16, 7.01–7.09 (m, 3H, Trp-H_{arom}), 6.99 (d, *J* = 7.7 Hz, 1H, CHN<u>H</u>Asn), 6.79 (d, *J* = 6.5 Hz, 1H, CHN<u>H</u>Trp), 6.72 (d, *J* = 9.2 Hz, 1H, C-1-NH), 5.47 (d, *J* = 7.1 Hz, 1H, CHN<u>H</u>Fmoc), 5.18–5.26 (m, 1H, H-3), 5.08–5.15 (m, 1H, H-1), 4.97–5.03 (m, 1H, H-4), 4.90–4.96 (m, 1H, H-2), 4.72 (q, *J* = 6.1 Hz, 1H, C<u>H</u>NHTrp), 4.49–4.56 (m, 1H, C<u>H</u>NHAsn), 4.32–4.40 (m, 1H, Fmoc-CH_2), 4.24–4.31 (m, 2H, H-6a, Fmoc-CH_2), 4.14–4.23 (m, 2H, Fmoc-CH, C<u>H</u>NHFmoc), 4.01 (dd, $J_{6b,6a}$ = 12.3 Hz, $J_{6b,5}$ = 1.8 Hz, 1H, H-6b), 3.64–3.74 (m, 1H, H-5), 3.28 (dd, *J* = 14.8 Hz, *J* = 6.1 Hz, 1H, Trp-CH_2), 3.25 (dd, *J* = 14.7 Hz, *J* = 5.6 Hz, 1H, Trp-CH_2), 2.63 (dd, *J* = 15.9 Hz, *J* = 4.0 Hz, 1H, Asn-CH_2), 2.48 (dd, *J* = 15.9 Hz, *J* = 4.8 Hz, 1H, Asn-CH_2), 2.02, 2.00, 1.95 (s, 12H, CH_3), 1.42 (s, 9H, $C(CH_3)_3$), 1.32 (d, *J* = 6.6 Hz, 3H, Ala-CH_3).

^{13}C NMR (101 MHz, $CDCl_3$): δ = 172.3, 170.8, 170.8, 170.7, 170.6, 169.9, 169.5, 169.0 (AcC=O, ^{t}BuC=O, NHC=O), 155.9 (NHC=O), 143.8, 141.2, 136.1($C_{q,arom}$), 127.8 (Fmoc-C_{arom}), 127.5 ($C_{q,arom}$), 127.1, 125.2 (Fmoc-C_{arom}), 123.2, 122.2 (Trp-C_{arom}), 120.0 (Fmoc-C_{arom}), 119.6, 118.7, 111.3 (Trp-C_{arom}), 109.8 ($C_{q,arom}$), 82.5 (<u>C</u>$(CH_3)_3$), 78.0 (C-1), 73.7 (C-5), 72.8 (C-3), 70.4 (C-2), 68.2 (C-4), 67.0 (Fmoc-CH_2), 61.6 (C-6), 53.8 (CHNHTrp), 50.5 (CHNHFmoc), 49.5 (CHNHAsn), 47.1 (Fmoc-CH), 37.7 (Asn-CH_2), 27.8 (C(<u>C</u>H_3)$_3$), 27.6 (Trp-CH_2), 20.7, 20.6, 20.6, 20.5 (CH_3), 18.6 (Ala-CH_3).

$[\boldsymbol{\alpha}]_{\mathbf{D}}^{\mathbf{20}}$ = +5.7 (c = 1, $CHCl_3$). **Mass** Anal. Calcd for $C_{51}H_{59}N_5O_{16}$ $[M+Na]^+$: m/z 1020.38490; HRMS found $[M+Na]^+$: m/z 1020.38502. **Anal.** Calcd for $C_{51}H_{59}N_5O_{16}$ C: 61.38%, H: 5.96%, N: 7.02%; found C: 61.19%, H: 6.85%, N: 6.85%.

N^2-[N^2[N^2-[(9*H*-Fluoren-9-ylmethoxy)carbonyl]alaninyl]tryptophyl]-*N*-(2,3,4,6-tetra-*O*-acetyl-β-D-galactopyranosyl)-L-asparagine-*tert*-butyl ester (7b)

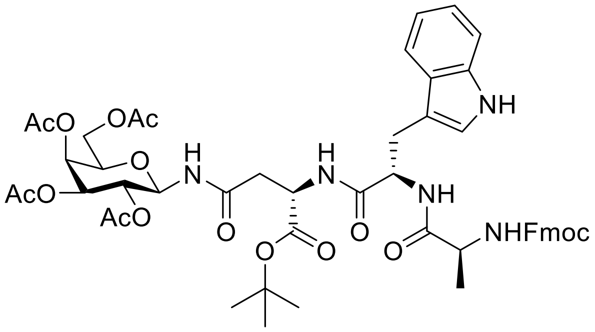

According to General Procedure D, **7b** was obtained from **5b** and Fmoc-L-Ala-OH as a yellowish solid. Yield: 76% (Procedure D1)/79% (Procedure D2).

^{1}H NMR (300 MHz, $CDCl_3$): δ = 8.23 (br. s., 1H, Trp-NH), 7.76 (d, *J* = 7.5 Hz, 2H, Fmoc-H_{arom}), 7.64 (d, *J* = 7.7 Hz, 1H, Trp-H_{arom}), 7.53–7.61 (m, 2H, Fmoc-H_{arom}), 7.40 (td, *J* = 7.1 Hz, *J* = 2.4 Hz, 2H, Fmoc-H_{arom}), 7.26–7.34 (m, 3H, Trp-H_{arom}, Fmoc-H_{arom}), 7.15 (dd, *J* = 7.0 Hz, *J* = 1.0 Hz, 1H, Trp-H_{arom}), 7.10 (dd, *J* = 6.3 Hz, *J* = 1.0 Hz, 1H, Trp-H_{arom}), 7.03–7.07 (m, 1H, Trp-H_{arom}), 7.01 (d, *J* = 7.6 Hz, 1H, CHN<u>H</u>Asn), 6.73 (m, 2H, CHN<u>H</u>Trp, C-1-NH), 5.43 (d, *J* = 6.9 Hz, 1H, CHN<u>H</u>Fmoc), 5.34–5.38 (m, 1H, H-4), 5.07–5.15 (m, 2H, H-1, H-2), 5.05 ($J_{3,2}$ = 10.2 Hz, $J_{3,4}$ = 3.0 Hz, 1H, H-3), 4.71 (q, *J* = 6.7 Hz, 1H, C<u>H</u>NHTrp), 4.53–4.61 (m, 1H, C<u>H</u>NHAsn), 4.31–4.39 (m, 1H, Fmoc-CH_2), 4.13–4.30 (m, 4H, H-6a, C<u>H</u>NHFmoc, Fmoc-CH, Fmoc-CH_2), 4.01 (dd, $J_{6b,6a}$ = 11.4 Hz, $J_{6b,5}$ = 5.8 Hz, H-6b), 3.84–3.92 (m, 1H, H-5), 3.24–3.33 (m, 2H, Trp-CH_2), 2.67 (dd, *J* = 15.8 Hz, *J* = 3.9 Hz, 1H, Asn-CH_2), 2.55 (dd, *J* = 15.7 Hz, *J* = 4.7 Hz, 1H, Asn-CH_2), 2.04, 2.01, 1.93, 1.92 (s, 12H, CH_3), 1.43 (s, 9H, $C(CH_3)_3$), 1.34 (d, *J* = 7.0 Hz, 3H, Ala-CH_3).

^{13}C NMR (75 MHz, $CDCl_3$): δ = 172.1, 171.0, 170.7, 170.6, 170.5, 170.0, 169.8, 169.1 (AcC=O, ^{t}BuC=O, NHC=O), 154.4 (NHC=O), 143.8, 141.3, 136.1 ($C_{q,arom}$), 127.8 (Fmoc-C_{arom}), 127.5 ($C_{q,arom}$), 127.1 (Trp-C_{arom}, Fmoc-C_{arom}), 125.2 (Fmoc-C_{arom}), 123.2, 122.3

(Trp-C_{arom}), 120.0 (Fmoc-C_{arom}), 119.6, 118.7, 111.4 (Trp-C_{arom}), 110.0 ($C_{q,arom}$), 82.5 ($\underline{C}(CH_3)_3$), 78.4 (C-1), 72.5 (C-5), 70.9 (C-3), 68.0 (C-2), 67.2 (C-4), 67.0 (Fmoc-CH_2), 61.4 (C-6), 53.8 (CHNHTrp), 50.6 (CHNHFmoc), 49.5 (CHNHAsn), 47.1 (Fmoc-CH), 37.8 (Asn-CH_2), 27.9 ($C(\underline{C}H_3)_3$), 27.6 (Trp-CH_2), 20.7, 20.5, 20.3 (CH_3), 18.7 (Ala-CH_3).

$[\boldsymbol{\alpha}]_{\mathbf{D}}^{\mathbf{20}}$ = +3.3 (c = 1, $CHCl_3$). **Mass** Anal. Calcd for $C_{51}H_{59}N_5O_{16}$ $[M+Na]^+$: m/z 1020.38490; HRMS found $[M+Na]^+$: m/z 1020.38478. **Anal.** Calcd for $C_{51}H_{59}N_5O_{16}$ C: 61.38%, H: 5.96%, N: 7.02%; found C: 61.72%, H: 6.39%, N: 7.09%.

N^2-[N^2[N^2-[(9*H*-Fluoren-9-ylmethoxy)carbonyl]alaninyl]tryptophyl]-*N*-(2,3,4,6-tetra-*O*-acetyl-β-D-mannopyranosyl)-L-asparagine-*tert*-butyl ester (7c)

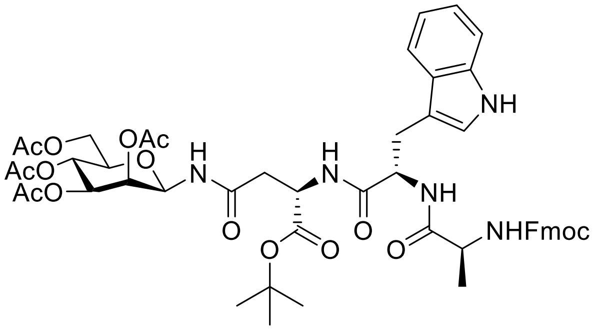

According to General Procedure D, **7c** was obtained from **5c** and Fmoc-L-Ala-OH as a yellowish solid. Yield: 76% (Procedure D1)/58% (Procedure D2).

^{1}H NMR (400 MHz, $CDCl_3$): δ = 8.31 (br. s., 1H, Trp-NH), 7.76 (d, *J* = 7.6 Hz, 2H, Fmoc-H_{arom}), 7.54–7.62 (m, 3H, Trp-H_{arom}, Fmoc-H_{arom}), 7.36–7.43 (m, 2H, Fmoc-H_{arom}), 7.28–7.34 (m, 2H, Fmoc-H_{arom}), 7.22–7.27, 7.08–7.13, 7.05–7.08 (m, 3H, Trp-H_{arom}), 7.01–7.05 (m, 2H, CHN$\underline{H}$Asn, Trp-H_{arom}), 6.82–6.90 (m, 2H, CHN$\underline{H}$Trp, C-1-NH), 5.32–5.39 (m, 2H, H-1, CHN$\underline{H}$Fmoc), 5.31 (dd, $J_{2,3}$ = 3.1 Hz, $J_{2,1}$ = 0.8 Hz, 1H, H-2), 5.18–5.25 (m, 1H, H-4), 5.06 (dd, $J_{3,4}$ = 10.1 Hz, $J_{3,2}$ = 3.2 Hz, 1H, H-3), 4.76 (q, *J* = 6.7 Hz, 1H, C$\underline{H}$NHTrp), 4.39–4.45 (m, 1H, C$\underline{H}$NHAsn), 4.34–4.39 (m, 1H, Fmoc-CH_2),

4.19–4.32 (m, 3H, H-6a, C<u>H</u>NHFmoc, Fmoc-CH_2), 4.14–4.19 (m, 1H, Fmoc-CH), 4.04–4.09 (m, 1H, H-6b), 3.71 (ddd, $J_{5,4}$ = 9.7 Hz, $J_{5,6}$ = 4.8 Hz, $J_{5,6}$ = 1.8 Hz, 1H, H-5), 3.34 (dd, J = 14.5 Hz, J = 5.1 Hz, 1H, Trp-CH_2), 3.22 (dd, J = 14.8 Hz, J = 5.5 Hz, 1H, Trp-CH_2), 2.53–2.66 (m, 2H, Asn-CH_2), 2.04, 2.03, 1.99, 1.98 (s, 12H, CH_3), 1.41 (s, 9H, $C(CH_3)_3$), 1.32 (d, J = 6.8 Hz, 3H, Ala-CH_3).

^{13}C NMR (101 MHz, $CDCl_3$): δ = 172.2, 171.3, 170.8, 170.7, 170.0, 169.7, 169.4, 169.1 (AcC=O, ^{t}BuC=O, NHC=O), 155.9 (NHC=O), 143.8, 143.7, 141.3, 136.1 ($C_{q,arom}$), 127.8 (Fmoc-C_{arom}), 127.5 ($C_{q,arom}$), 127.1 (Fmoc-C_{arom}), 125.1 (Trp-C_{arom}), 125.1 (Fmoc-C_{arom}), 123.3, 122.1 (Trp-C_{arom}), 120.0 (Fmoc-C_{arom}), 119.6, 118.7, 111.3 (Trp-C_{arom}), 109.8 ($C_{q,arom}$), 82.9 (<u>C</u>$(CH_3)_3$), 76.2 (C-1), 74.2 (C-5), 71.6 (C-3), 69.7 (C-2), 67.0 (Fmoc-CH_2), 65.2 (C-4), 62.1 (C-6), 53.6 (CHNHTrp), 50.6 (CHNHFmoc), 49.5 (CHNHAsn), 47.0 (Fmoc-CH), 38.0 (Asn-CH_2), 27.8 (C(<u>C</u>$H_3)_3$), 27.6 (Trp-CH_2), 20.9, 20.7, 20.6 (CH_3), 18.5 (Ala-CH_3).

$[\alpha]_D^{20}$ = – 13.6 (c = 1, $CHCl_3$). **Mass** Anal. Calcd for $C_{51}H_{59}N_5O_{16}$ $[M+Na]^+$: m/z 1020.38490; HRMS found $[M+Na]^+$: m/z 1020.38353.

N^2-[N^2[N^2-[(9*H*-Fluoren-9-ylmethoxy)carbonyl]alaninyl]tryptophyl]-*N*-(2,2’,3,3’,4,6,6’-hepta-*O*-acetyl-β-D-maltosyl)-L-asparagine-*tert*-butyl ester (7d)

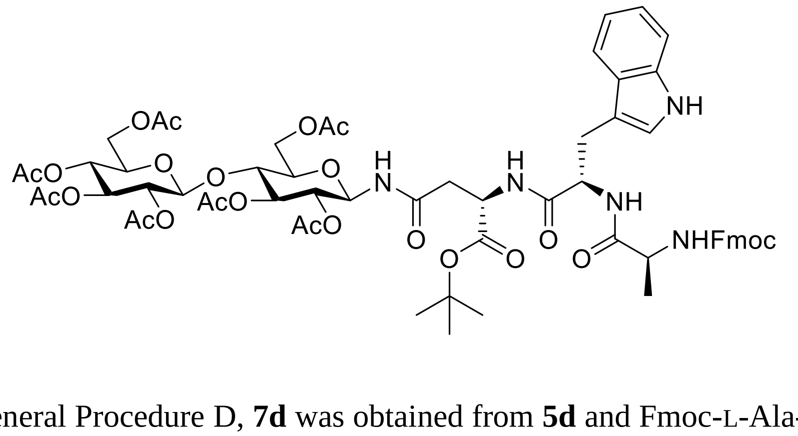

According to General Procedure D, **7d** was obtained from **5d** and Fmoc-L-Ala-OH as a white-yellow solid. Yield: 60% (Procedure D1)/68% (Procedure D2).

^{1}H NMR (300 MHz, $CDCl_3$): δ = 8.31 (s, 1H, Trp-NH), 7.76 (d, *J* = 7.4 Hz, 2H, Fmoc-H_{arom}), 7.51–7.65 (m, 3H, Trp-H_{arom}, Fmoc-H_{arom}), 7.35–7.44, 7.26–7.34 (m, 4H, Fmoc-H_{arom}), 7.22 (br. s., 1H, Trp-H_{arom}), 7.08 – 7.15 (m, 1H, Trp-H_{arom}), 7.00–7.08 (m, 3H, CHN$\underline{H}$Asn, Trp-H_{arom}), 6.81 (d, *J* = 6.9 Hz, 1H, CHN$\underline{H}$Trp), 6.74 (d, *J* = 9.1 Hz, 1H, C-1-NH), 5.50 (d, *J* = 7.3 Hz, 1H, CHN$\underline{H}$Fmoc), 5.13–5.22 (m, 1H, H-3), 4.98–5.12 (m, 3H, H-1, H-3‘, H-4‘), 4.82–4.93 (m, 2H, H-2, H-2‘), 4.69–4.78 (m, 1H, C$\underline{H}$NHTrp), 4.47–4.55 (m, 1H, C$\underline{H}$NHAsn), 4.43 (d, $J_{1‘,2‘}$ = 8.0 Hz, 1H, H-1‘), 4.31–4.40 (m, 3H, Fmoc-CH_2, H-6a, H-6b), 4.24–4.31 (m, 1H, Fmoc-CH_2), 4.12–4.23 (m, 3H, C$\underline{H}$NHFmoc, Fmoc-CH, H-6a‘), 3.95 (dd, $J_{6b‘,6a‘}$ = 12.3 Hz, $J_{6b‘,5‘}$ = 1.7 Hz, 1H, H-6b‘), 3.61–3.70 (m, 1H, H-4), 3.52–3.60 (m, 2H, H-5, H-5‘), 3.26 (d, *J* = 5.7 Hz, 2H, Trp-CH_2), 2.59 (dd, *J* = 15.7 Hz, *J* = 3.9 Hz, 1H, Asn-CH_2), 2.51 (dd, *J* = 15.6 Hz, *J* = 4.9 Hz, 1H, Asn-CH_2), 2.04, 2.00, 1.98, 1.97, 1.95 (s, 21H, CH_3), 1.42 (s, 9H, $C(CH_3)_3$), 1.33 (d, *J* = 6.8 Hz, 3H, Ala-CH_3).

^{13}C NMR (75 MHz, $CDCl_3$): δ = 172.3, 170.9, 170.8, 170.8, 170.5, 170.4, 170.2, 169.5, 169.3, 168.9 (AcC=O, ^{t}BuC=O, NHC=O), 155.9 (NHC=O), 143.8, 143.8, 141.2, 136.1 ($C_{q,arom}$), 127.8 (Fmoc-C_{arom}), 127.5 ($C_{q,arom}$), 127.1, 125.1 (Fmoc-C_{arom}), 123.1, 122.1 (Trp-C_{arom}), 120.0 (Fmoc-C_{arom}), 119.5, 118.7, 111.3 (Trp-C_{arom}), 109.9 ($C_{q,arom}$), 100.6 (C-1‘), 82.6 ($\underline{C}(CH_3)_3$), 77.9 (C-1), 76.3 (C-5), 74.8 (C-5‘), 72.8 (C-3‘), 72.4 (C-3), 71.9 (C-4), 71.5 (C-2‘), 70.6 (C-2), 67.7 (C-4‘), 67.0 (Fmoc-CH_2), 61.7 (C-6), 61.5 (C-6‘), 53.8 (CHNHTrp), 50.6 (CHNHFmoc), 49.6 (CHNHAsn), 47.1 (Fmoc-CH), 37.8 (Asn-CH_2), 27.8 ($C(\underline{C}H_3)_3$), 27.6 (Trp-CH_2), 20.8, 20.6, 20.5, 20.4 (CH_3), 18.7 (Ala-CH_3).

$[\alpha]_D^{20}$ = –1.3 (c = 0.5, $CHCl_3$). **Mass** Anal. Calcd for $C_{63}H_{75}N_5O_{24}$ $[M+Na]^+$: m/z 1308.46942; HRMS found $[M+Na]^+$: m/z 1308.46697. **Anal.** Calcd for $C_{63}H_{75}N_5O_{24}$ C: 58.83 %, H: 5.88%, N: 5.44%; found C: 59.02%, H: 6.18%, N: 5.33%.

***N*²-[*N*²[*N*²-[(9*H*-Fluoren-9-ylmethoxy)carbonyl]alaninyl]tryptophyl]-*N*-(2,2',3,3',4,6,6'-hepta-*O*-acetyl-β-D-maltosyl)-L-asparagine-*tert*-butyl ester (7e)**

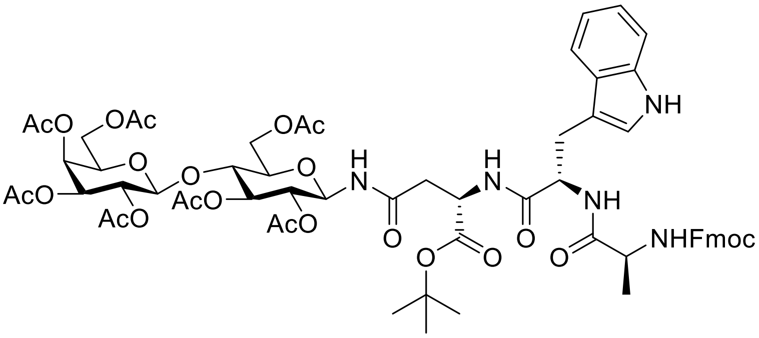

According to General Procedure D, **7e** was obtained from **5e** and Fmoc-L-Ala-OH as a colorless solid. Yield: 75% (Procedure D1)/65% (Procedure D2).

^{1}H NMR (400 MHz, $CDCl_3$): δ = 8.22 (br. s., 1H, Trp-NH), 7.77 (d, *J* = 7.6 Hz, 2H, Fmoc-H_{arom}), 7.55–7.64 (m, 3H, Trp-H_{arom}, Fmoc-H_{arom}), 7.36–7.44 (m, 2H, Trp-H_{arom}), 7.27–7.35 (m, 2H, Fmoc-H_{arom}), 7.23 (br. s., 1H, Trp-H_{arom}), 7.10–7.16, 7.03–7.09 (m, 3H, Trp-H_{arom}), 6.99 (d, *J* = 7.3 Hz, 1H, CHNHAsn), 6.71 (d, *J* = 6.6 Hz, 1H, CHNHTrp), 6.64 (d, *J* = 9.0 Hz, 1H, C-1-NH), 5.44 (d, *J* = 7.0 Hz, 1H, CHNHFmoc), 5.30–5.33 (m, 1H, H-4'), 5.16–5.23 (m, 1H, H-3), 5.02–5.12 (m, 2H, H-1, H-2'), 4.92 (dd, $J_{3',2'}$ = 10.4 Hz, $J_{3',4'}$ = 3.4 Hz, 1H, H-3'), 4.82–4.89 (m, 1H, H-2), 4.70–4.77 (m, 1H, CHNHTrp), 4.49–4.55 (m, 1H, CHNHAsn), 4.33–4.43 (m, 3H, Fmoc-CH_2, H-6a, H-1'), 4.14–4.31 (m, 4H, Fmoc-CH, CHNHFmoc, Fmoc-CH_2, H-6b), 3.99–4.09 (m, 1H, H-6a', H-6b'), 3.77–3.83 (m, 1H, H-5'), 3.64–3.71 (m, 1H, H-4), 3.56–3.63 (m, 1H, H-5), 3.21–3.33 (m, 2H, Trp-CH_2), 2.60 (dd, *J* = 15.6 Hz, *J* = 3.9 Hz, 1H, Asn-CH_2), 2.48 (dd, *J* = 15.4 Hz, *J* = 4.5 Hz, 1H, Asn-CH_2), 2.14, 2.05, 2.03, 2.02, 1.99, 1.96 (s, 21H, CH_3), 1.42 (s, 9H, $C(CH_3)_3$), 1.34 (d, *J* = 6.7 Hz, 3H, Ala-CH_3).

^{13}C NMR (101 MHz, $CDCl_3$): δ = 172.2, 170.9, 170.9, 170.5, 170.3, 170.1, 170.1, 169.4, 168.9 (AcC=O, ^{t}BuC=O, NHC=O), 155.9 (NHC=O), 143.8, 143.8, 141.2, 136.1 ($C_{q,arom}$), 127.8

(Fmoc-C$_{arom}$), 127.5 (C$_{q,arom}$), 127.1, 125.1 (Fmoc-C$_{arom}$), 123.1, 122.2 (Trp-C$_{arom}$), 120.0 (Fmoc-C$_{arom}$), 119.6, 118.8, 111.3 (Trp-C$_{arom}$), 110.0 (C$_{q,arom}$), 100.9 (C-1‘), 82.6 (C(CH$_3$)$_3$), 77.8 (C-1), 76.1 (C-4), 74.8 (C-5), 72.6 (C-3), 70.9 (C-3‘), 70.7, 70.6 (C-2, C-5‘), 69.0 (C-2‘), 67.0 (Fmoc-CH$_2$), 66.6 (C-4‘), 61.8 (C-6), 60.7 (C-6‘), 53.7 (CHNHTrp), 50.6 (CHNHFmoc), 49.6 (CHNHAsn), 47.1 (Fmoc-CH), 37.8 (Asn-CH$_2$), 27.8 (C(CH$_3$)$_3$), 27.6 (Trp-CH$_2$), 20.9, 20.7, 20.7, 20.6, 20.5 (CH$_3$), 18.6 (Ala-CH$_3$).

$[\alpha]_D^{20}$ = + 0.3 (c = 0.5, CHCl$_3$). **Mass** Anal. Calcd for $C_{63}H_{75}N_5O_{24}$ $[M+Na]^+$: m/z 1308.46942; HRMS found $[M+Na]^+$: m/z 1308.46825. **Anal.** Calcd for $C_{63}H_{75}N_5O_{24}$ C: 58.83%, H: 5.88%, N: 5.44%; found C: 58.56%, H: 5.97%, N: 5.47%.

N^2-[N^2[N^2-[(9*H*-Fluoren-9-ylmethoxy)carbonyl]alaninyl]tryptophyl]-*N*-(2,2’,3,3’,4,6,6’-hepta-*O*-acetyl-β-D-maltosyl)-L-asparagine-*tert*-butyl ester (7f)

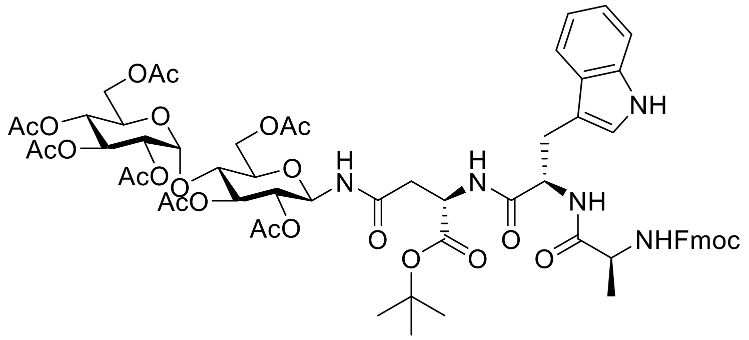

According to General Procedure D, **7f** was obtained from **5f** and Fmoc-L-Ala-OH as a white-yellow solid. Yield: 58% (Procedure D1)/50% (Procedure D2).

^{1}H NMR (300 MHz, CDCl$_3$): δ = 8.42 (br. s., 1H, Trp-NH), 7.78 (d, *J* = 7.4 Hz, 2H, Fmoc-H$_{arom}$), 7.52–7.63 (m, 3H, Trp-H$_{arom}$, Fmoc-H$_{arom}$), 7.35–7.45, 7.26–7.34 (m, 2H, Fmoc-H$_{arom}$), 7.22 (s, 1H, Trp-H$_{arom}$), 7.08–7.15 (m, 1H, Trp-H$_{arom}$), 7.00–7.08 (m, 3H, CHNHAsn, Trp-H$_{arom}$), 6.86 (d, *J* = 6.8 Hz, 1H, CHNHTrp), 6.70 (d, *J* = 9.3 Hz, 1H, C-1-NH), 5.50 (d, *J* = 7.2 Hz, 1H, CHNHFmoc), 5.32–5.40 (m, 2H, H-1‘, H-3‘), 5.18–5.27 (m, 1H, H-3),

5.00–5.13 (m, 2H, H-1, H-4‘), 4.84 (dd, $J_{2',3'}$ = 10.6 Hz, $J_{2',1'}$ = 4.0 Hz, 1H, H-2‘), 4.69–4.80 (m, 2H, H-2, CHNHTrp), 4.46–4.55 (m, 1H, CHNHAsn), 4.31–4.42 (m, 2H, Fmoc-CH_2, H-6a), 4.12–4.30 (m, 5H, H-6a‘, H-6b, Fmoc-CH_2, Fmoc-CH, CHNHFmoc), 4.05 (dd, $J_{6b',6a'}$ = 12.3 Hz, $J_{6b',5}$ = 1.7 Hz, H-6b‘), 3.81–3.93 (m, 2H, H-4,H-5‘), 3.48–3.59 (m, 1H, H-5), 3.19–3.34 (m, 2H, Trp-CH_2), 2.60 (dd, J = 15.9 Hz, J = 3.9 Hz, 1H, Asn-CH_2), 2.51 (dd, J = 15.8 Hz, J = 4.8 Hz, 1H, Asn-CH_2), 2.06, 2.05, 2.04, 2.02, 2.00, 1.98, 1.95 (s, 21H, CH_3), 1.42 (s, 9H, $C(CH_3)_3$), 1.32 (d, J = 6.7 Hz, 3H, Ala-CH_3).

^{13}C NMR (75 MHz, $CDCl_3$): δ = 172.3, 170.9, 170.8, 170.7, 170.6, 170.4, 169.9, 169.8, 169.4, 169.0 (AcC=O, ^{t}BuC=O, NHC=O), 155.9 (NHC=O), 143.8, 141.3, 136.6 ($C_{q,arom}$), 127.8 (Fmoc-C_{arom}), 127.5 ($C_{q,arom}$), 127.1, 125.2 (Fmoc-C_{arom}), 123.1, 122.1 (Trp-C_{arom}), 120.0 (Fmoc-C_{arom}), 119.5, 118.6, 111.4 (Trp-C_{arom}), 109.8 ($C_{q,arom}$), 95.5 (C-1‘), 82.5 ($C(CH_3)_3$), 77.5 (C-1), 75.3 (C-3), 74.0 (C-5), 72.4 (C-5‘), 71.1 (C-2), 69.9 (C-2‘), 69.3 (C-3‘), 68.4 (C-4), 67.9 (C-4‘), 67.0 (Fmoc-CH_2), 62.4 (C-6), 61.3 (C-6‘), 53.8 (CHNHTrp), 50.6 (CHNHFmoc), 49.6 (CHNHAsn), 47.1 (Fmoc-CH), 37.7 (Asn-CH_2), 27.8 ($C(CH_3)_3$), 27.5 (Trp-CH_2), 20.8, 20.6, 20.6 (CH_3), 18.6 (Ala-CH_3).

$[\alpha]_D^{20}$ = + 43.1 (c = 1, $CHCl_3$). **Mass** Anal. Calcd for $C_{63}H_{75}N_5O_{24}$ $[M+Na]^+$: m/z 1308.46942; HRMS found $[M+Na]^+$: m/z 1308.46811. **Anal.** Calcd for $C_{63}H_{75}N_5O_{24}$ C: 58.83%, H: 5.88%, N: 5.44%; found C: 59.02%, H: 6.14%, N: 5.37%.

***N*²-[*N*²[*N*²-[(9*H*-Fluoren-9-ylmethoxy)carbonyl]alaninyl]tryptophyl]-*N*-(2,3,4,6-tetra-*O*-acetyl-β-D-glucopyranosyl)-L-asparagine (8a)**

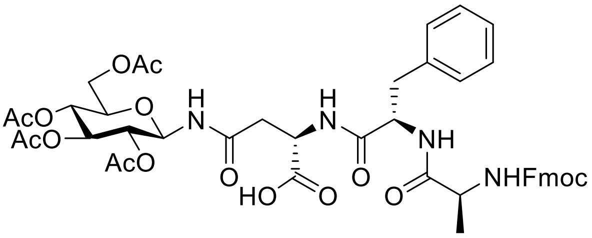

According to General Procedure E, **8a** was obtained from **6a** as a colorless solid. Yield: 99%.

^{1}H-NMR (400 MHz, $CDCl_3$): δ = 7.77, 7.55 (d, *J* = 7.5 Hz, 4H, Fmoc-H_{arom}), 7.38–7.45 (m, 2H, Fmoc-H_{arom}), 7.32 (m, 3H, Fmoc-H_{arom}, C-1-NH), 7.21–7.23 (m, 2H, Ph-H_{arom}), 7.10–7.17 (m, 3H, Ph-H_{arom}), 6.97 (d, J = 7.8 Hz, 1H, CHN<u>H</u>Gln), 6.49 (d, *J* = 6.6 Hz, 1H, CHN<u>H</u>Phe), 5.20–5.27 (m, 3H, H-3, H-1), 5.05–5.12 (m, 3H, H-4), 4.97 (s, 1H, H-2), 4.57 (q, *J* = 6.6 Hz, 1H, H-C<u>H</u>NHPhe), 4.36–4.44 (m, 2H, C<u>H</u>NHGln, Fmoc-CH_2), 4.19–4.28 (m, 2H, Fmoc-CH_2, H-6b), 4.10–4.16 (m, 2H, C<u>H</u>NHFmoc, Fmoc-CH), 4.04–4.09 (m, 1H, H-6a), 3.74–3.81 (m, 1H, H-5), 3.13 (d, *J* = 6.1 Hz, 2H, Phe-CH_2), 2.19–2.30 (m, 1H, Gln-$C^{\gamma}H_2$), 2.09–2.18 (m, 2H, Gln-$C^{\gamma}H_2$, Gln-$C^{\beta}H_2$), 2.06, 2.01, 1.97, 1.94 (s, 12H, CH_3), 1.74–1.90 (m, 1H, Gln-$C^{\beta}H_2$), 1.38 (s, 9H, $C(CH_3)_3$), 1.32 (d, *J* = 7.1 Hz, 3H, Ala-CH_3).

^{13}C-NMR (101 MHz, $CDCl_3$): δ = 173.1, 172.4, 171.5 (NHCO), 170.8 (^{t}BuC=O), 170.7, 170.3, 170.0, 169.5 (AcOC=O), 156.5 (NHCO), 143.5, 141.3, 136.0 ($C_{q,arom}$), 129.1, 128.9 (Ph-C_{arom}), 127.9 (Fmoc-C_{arom}), 127.2 (Ph-C_{arom}), 127.1, 124.5,120.1 (Fmoc-C_{arom}), 80.8 (C(CH3)3), 77.9 (C-1), 73.7 (C-5), 73.4 (C-3), 70.8 (C-2), 68.2 (C-4), 67.1 (Fmoc-CH_2), 61.9 (C-6), 54.5 (CHNHPhe), 52.9(CHNHGln), 51.3 (Fmoc-CH), 47.0 (CHNHFmoc), 36.6 (Phe-CH_2), 31.7 (Gln-$C^{\gamma}H_2$), 28.0 (C(<u>C</u>H3)3), 26.5 (Gln-$C^{\beta}H_2$), 20.8, 20.7, 20.6 (CH_3), 17.7 (Ala-CH_3).

$[\alpha]_D^{20}$ = – 5.5 (c = 1, MeOH). **Mass** Anal. Calcd for $C_{45}H_{50}N_4O_{16}$ $[M+Na]^+$: m/z 925.31140; HRMS found $[M+Na]^+$: m/z 925.30982.

N^2-[N^2[N^2-[(9*H*-Fluoren-9-ylmethoxy)carbonyl]alaninyl]tryptophyl]-*N*-(2,3,4,6-tetra-*O*-acetyl-β-D-galactopyranosyl)-L-asparagine (8b)

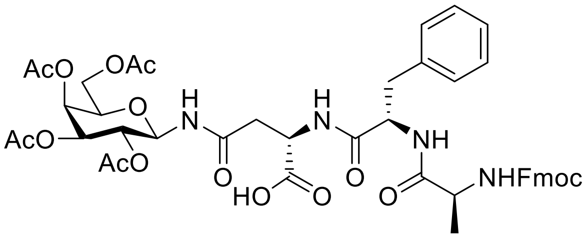

According to General Procedure E, **8b** was obtained from **6b** as a colorless solid. Yield: 99%.

^{1}H NMR (300 MHz, $CDCl_3$): δ = 7.75 (d, *J* = 7.5 Hz, 2H, Fmoc-H_{arom}), 7.52–7.61 (m, 3H, Fmoc-H_{arom}, CHN<u>H</u>Phe), 7.34–7.43 (m, 2H, Fmoc-H_{arom}), 7.29 (dd, *J* = 7.3 Hz, *J* = 2.1 Hz, 2H, Fmoc-H_{arom}), 7.08–7 .23 (m, 7H, Ph-H_{arom}, C-1-NH, CHN<u>H</u>Asn), 6.37 (br. s., 1H, COOH), 5.76 (d, *J* = 6.3 Hz, 1H, CHN<u>H</u>Fmoc), 5.40–5.45 (m, 1H, H-4), 5.22–5.35 (m, 1H, H1), 5.08–5.18 (m, 2H, H-2, H-3), 4.73–4.83 (m, 1H, C<u>H</u>NHAsn), 4.65–4.73 (m, 1H, C<u>H</u>NHPhe), 4.23–4.45 (m, 3H, C<u>H</u>NHFmoc, Fmoc-CH_2), 4.11–4.22 (m, 2H, Fmoc-CH, H-6a), 3.95–4.11 (m, 2H, H-5, H-6b), 3.19 (dd, *J* = 14.3 Hz, *J* = 5.8 Hz, 1H, Asn-CH_2), 2.98 (dd, *J* = 13.8 Hz, *J* = 8.3 Hz, 1H, Asn-CH_2), 2.75–2.88 (m, 2H, Phe-CH_2), 2.00, 2.00, 1.94 (s, 12H, CH_3), 1.31 (d, *J* = 5.8 Hz, 3H, Ala-CH_3).

^{13}C NMR (75 MHz, $CDCl_3$): δ = 173.0, 171.7, 171.5, 171.2, 171.2, 170.3, 170.1 (AcC=O, COOH, NHC=O), 156.2 (NHC=O), 143.7, 141.3, 137.0, 135.9 ($C_{q,arom}$), 129.2, 128.6 (Ph-C_{arom}), 127.8 (Fmoc-C_{arom}), 127.1 (Ph-C_{arom}, Fmoc-C_{arom}), 125.1 (Fmoc-C_{arom}), 120.0 (Fmoc-C_{arom}), 78.2 (C-1), 72.1 (C-5), 71.0 (C-2, C-3), 68.4 (C-2, C-3), 67.3 (C-4), 67.3 (Fmoc-CH_2), 61.3 (C-6), 54.6 (CHNHAsn), 49.9 (CHNHFmoc), 49.2 (CHNHPhe), 47.0 (Fmoc-CH), 37.7 (Asn-CH_2), 37.3 (Phe-CH_2), 20.8, 20.6, 20.4 (CH_3), 18.5 (Ala-CH_3).

$[\alpha]_D^{20}$ = + 6.5 (c = 1, $CHCl_3$). **Mass** Anal. Calcd for $C_{45}H_{50}N_4O_{16}$ $[M+Na]^+$: m/z 925.31140; HRMS found $[M+Na]^+$: m/z 925.31087.

***N*[2]-[*N*[2][*N*[2]-[(9*H*-Fluoren-9-ylmethoxy)carbonyl]alaninyl]tryptophyl]-*N*-(2,3,4,6-tetra-*O*-acetyl-β-D-mannopyranosyl)-L-asparagine (8c)**

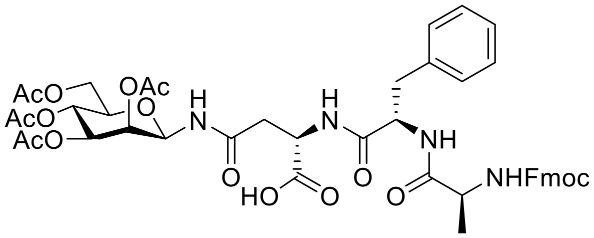

According to General Procedure E, **8c** was obtained from **6c** as a colorless solid. Yield: 99%.

^{1}H NMR (300 MHz, $CDCl_3$): δ = 7.74 (d, J = 7.5 Hz, 2H, Fmoc-H_{arom}), 7.66 (d, J = 5.1 Hz, 1H, CHNHPhe), 7.55 (d, J = 7.0 Hz, 2H, Fmoc-H_{arom}), 7.33–7.42 (m, 3H, CHNHAsn, Fmoc-H_{arom}), 7.26–7.32 (m, 2H, Fmoc-H_{arom}), 7.05–7.20 (m, 6H, Ph-H_{arom}, C-1-NH), 5.66 (d, J = 6.0 Hz, 1H, CHNHFmoc), 5.50–5.58 (m, 1H, H-1), 5.34–5.40 (m, 1H, H-2), 5.14–5.26 (m, 3H, H-3, H-4), 5.06 (br. s., 1H, COOH), 4.72–4.83 (m, 1H, CHNHAsn), 4.61–4.71 (m, 1H, CHNHPhe), 4.20–4.38 (m, 4H, H-6a, CHNHFmoc, Fmoc-CH_2), 4.10–4.19 (m, 1H, Fmoc-CH), 4.03 (dd, $J_{6b,6a}$ = 12.5 Hz, $J_{6b,5}$ = 1.7 Hz, 1H, H-6b), 3.72–3.82 (m, 1H, H-5), 3.16 (dd, J = 13.9 Hz, J = 4.7 Hz, 1H, Asn-CH_2), 2.90–3.02 (m, 1H, Asn-CH_2), 2.75–2.87 (m, 2H, Phe-CH_2), 2.11, 2.00, 1.98, 1.95 (s, 12H, CH_3), 1.21–1.30 (m, 3H, Ala-CH_3).

^{13}C NMR (75 MHz, $CDCl_3$): δ = 172.3, 172.0, 171.6, 171.0, 170.9, 170.7, 170.3, 169.7 (AcC=O, COOH, NHC=O), 156.2 (NHC=O), 143.7, 141.2, 136.0 ($C_{q,arom}$), 129.2, 128.5 (Ph-C_{arom}), 127.8, 127.1 (Fmoc-C_{arom}), 127.0 (Ph-C_{arom}), 125.1 (Fmoc-C_{arom}), 120.0 (Fmoc-C_{arom}), 76.3 (C-1), 74.0 (C-5), 71.6 (C-3), 69.7 (C-2), 67.2 (Fmoc-CH_2), 65.4 (C-4), 62.2 (C-6), 54.4 (CHNHAsn), 50.5 (CHNHFmoc), 49.1 (CHNHPhe), 47.0 (Fmoc-CH), 38.2 (Asn-CH_2), 37.7 (Phe-CH_2), 20.8, 20.7, 20.6 (CH_3), 18.3 (Ala-CH_3).

$[\alpha]_D^{20}$ = – 3.9 (c = 0.5, $CHCl_3$). **Mass** Anal. Calcd for $C_{45}H_{50}N_4O_{16}$ $[M+Na]^+$: m/z 925.31140; HRMS found $[M+Na]^+$: m/z 925.31140.

N^2-[N^2[N^2-[(9*H*-Fluoren-9-ylmethoxy)carbonyl]alaninyl]tryptophyl]-*N*-(2,2’,3,3’,4,6,6’-hepta-*O*-acetyl-β-D-cellobiosyl)-L-asparagine (8d)

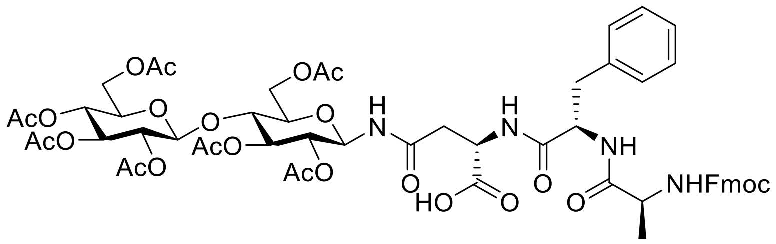

According to General Procedure E, **8d** was obtained from **6d** as a colorless solid. Yield: 99%.

^{1}H NMR (400 MHz, $CDCl_3$): δ = 7.74 (d, *J* = 7.5 Hz, 2H, Fmoc-H_{arom}), 7.57 (m, 3H, CHNHPhe, Fmoc-H_{arom}), 7.33–7.41 (m, 3H, C-1-NH, Fmoc-H_{arom}), 7.26–7.32 (m, 3H, CHNHAsn, Fmoc-H_{arom}), 7.07–7.22 (m, 5H, Ph-H_{arom}), 5.75 – 5.86 (m, 1H, CHNHFmoc), 5.17–5.28 (m, 2H, H-1, H-3), 5.08–5.15 (m, 1H, H-3‘), 5.00–5.08 (m, 1H, H-4‘), 4.81–4.94 (m, 2H, H-2, H-2‘), 4.72–4.79 (m, 1H, CHNHAsn), 4.64–4.71 (m, 1H, CHNHPhe), 4.45 (d, $J_{1‘,2‘}$ = 7.7 Hz, 1H, H-1‘), 4.21–4.42 (m, 5H, H-6a, H-6b, Fmoc-CH_2 CHNHFmoc), 4.10–4.20 (m, 2H, Fmoc-CH, H-6a‘), 3.90–3.99 (m, 1H, H-6b‘), 3.64–3.73 (m, 2H, H-4, H-5), 3.56–3.63 (m, 1H, H-5‘), 3.15 (dd, *J* = 13.8 Hz, *J* = 4.7 Hz, 1H, Asn-CH_2), 2.97 (d, *J* = 13.2 Hz, *J* = 8.0 Hz, 1H, Asn-CH_2), 2.69 – 2.85 (m, 2H, Phe-CH_2), 2.03, 2.02, 1.99, 1.98, 1.97, 1.95 (s, 21H, CH_3), 1.29 (d, *J* = 6.5 Hz, 3H, Ala-CH_3).

^{13}C NMR (101 MHz, $CDCl_3$): δ = 173.3, 171.8, 171.5, 171.1, 170.8, 170.6, 170.3, 169.9, 169.4, 169.3 (AcC=O, COOH, NHC=O), 156.2 (NHC=O), 143.7, 141.2, 135.9 ($C_{q,arom}$), 129.2, 128.6 (Ph-C_{arom}), 127.8 (Fmoc-C_{arom}), 127.1 (Fmoc-C_{arom}, Ph-C_{arom}), 125.1, 120.0 (Fmoc-C_{arom}), 100.7 (C-1‘), 77.7 (C-1), 76.3 (C-4), 74.7 (C-5), 72.8 (C-3‘), 72.5 (C-3), 71.9 (C-5‘), 71.5 (C-2‘), 70.6 (C-2), 67.7 (C-4‘), 67.3 (Fmoc-CH_2), 61.7 (C-6), 61.5 (C-6‘), 54.5 (CHNHAsn), 50.4 (CHNHFmoc), 49.2 (CHNHPhe), 47.0 (Fmoc-CH), 37.6 (Asn-CH_2), 37.4 (Phe-CH_2), 20.8, 20.6, 20.5 (CH_3), 18.4 (Ala-CH_3).

[α]$^{20}_{D}$ = – 0.9 (c = 0.5, CH_2Cl_2). **Mass** Anal. Calcd for $C_{57}H_{66}N_4O_{24}$ [M+Na]$^+$: m/z 1213.39592; HRMS found [M+Na]$^+$: m/z 1213.39566.

***N*²-[*N*²[*N*²-[(9*H*-Fluoren-9-ylmethoxy)carbonyl]alaninyl]tryptophyl]-*N*-(2,2',3,3',4,6,6'-hepta-*O*-acetyl-β-D-lactosyl)-L-asparagine (8e)**

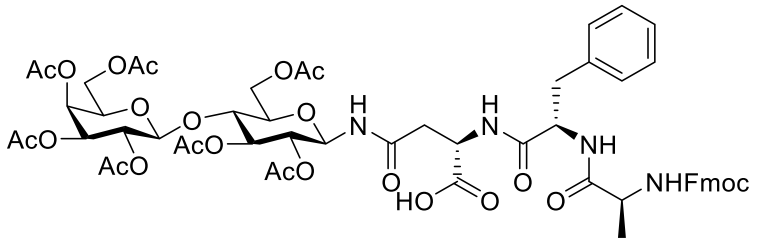

According to General Procedure E, **8e** was obtained from **6e** as a colorless solid. Yield: 99%.

¹H NMR (400 MHz, $CDCl_3$): δ = 7.74 (d, *J* = 7.5 Hz, 2H, Fmoc-H$_{arom}$), 7.51–7.64 (m, 3H, Fmoc-H$_{arom}$, CHNHPhe), 7.34–7.42 (m, 3H, Fmoc-H$_{arom}$, C-1-NH), 7.26–7.32 (m, 3H, 'Fmoc-H$_{arom}$, CHNHAsn), 7.08–7.22 (m, 5H, Ph-H$_{arom}$), 5.77–5.90 (m, 1H, CHNHFmoc), 5.67 (br. s., 1H, COOH), 5.30–5.34 (m, 1H, H-4'), 5.17–5.30 (m, 2H, H-1, H-3), 5.03–5.10 (m, 1H, H-2'), 4.93 (dd, $J_{3',2'}$ = 10.6 Hz, $J_{3',4}$ = 2.1 Hz, 1H, H-3'), 4.84–4.90 (m, 1H, H-2), 4.72–4.80 (m, 1H, CHNHAsn), 4.63–4.71 (m, 1H, CHNHPhe), 4.43 (d, $J_{1',2'}$ = 7.6 Hz, 1H, H-1'), 4.22–4.40 (m, 4H, H-6a, CHNHFmoc, Fmoc-CH_2), 4.10–4.20 (m, 2H, H-6b, Fmoc-CH), 3.97–4.09 (m, 2H, H-6a', H-6b'), 3.78–3.76 (m, 1H, H-5'), 3.65–3.76 (m, 2H, H-4, H-5), 3.16 (dd, *J* = 11.0 Hz, *J* = 2.5 Hz, 1H, Asn-CH_2), 2.90–3.02 (m, 1H, Asn-CH_2), 2.67–2.78 (m, 2H, Phe-CH_2), 2.12, 2.01, 1.98, 1.95 (s, 21H, CH_3), 1.29 (br. s., 3H, Ala-CH_3).

¹³C NMR (101 MHz, $CDCl_3$): δ = 173.2, 171.8, 171.5, 171.1, 170.8, 170.4, 170.2, 170.1, 169.8, 169.3 (AcC=O, COOH, NHC=O), 156.2 (NHC=O), 143.7, 141.2, 135.9 (C$_{q,arom}$), 129.2, 128.5 (Ph-C$_{arom}$), 127.8 (Fmoc-C$_{arom}$), 127.1 (Ph-C$_{arom}$, Fmoc-C$_{arom}$), 125.1, 120.0 (Fmoc-C$_{arom}$), 100.9 (C-1'), 77.7 (C-1), 76.0 (C-5), 74.7 (C-4), 72.8 (C-3), 70.9 (C-3'), 70.7 (C-2), 70.5

(C-5‘), 69.0 (C-2‘), 67.2 (Fmoc-CH_2), 66.5 (C-4‘), 61.8 (C-6), 60.6 (C-6‘), 54.5 (CHNHAsn), 50.4 (CHNHFmoc), 49.2 (CHNHPhe), 47.0 (Fmoc-CH), 37.6 (Asn-CH_2), 37.4 (Phe-CH_2), 20.8, 20.7, 20.6, 20.5 (CH_3), 18.5 (Ala-CH_3).

$[\alpha]_D^{20}$ = + 3.5 (c = 1, $CHCl_3$). **Mass** Anal. Calcd for $C_{57}H_{66}N_4O_{24}$ $[M+Na]^+$: m/z 1213.39592; HRMS found $[M+Na]^+$: m/z 1213.39566.

N[2]-[*N*[2][*N*[2]-[(9*H*-Fluoren-9-ylmethoxy)carbonyl]alaninyl]tryptophyl]-*N*-(2,2’,3,3’,4,6,6’-hepta-*O*-acetyl-β-D-maltosyl)-L-asparagine (8f)

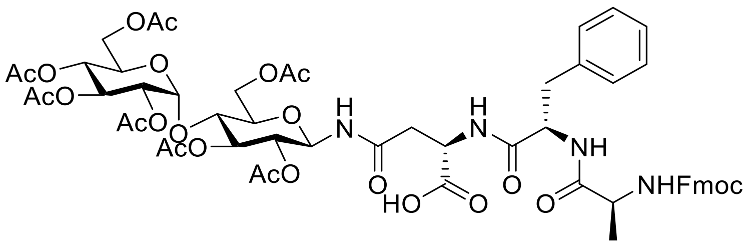

According to General Procedure E, **8f** was obtained from **6f** as a colorless solid. Yield: 99%.

^{1}H NMR (400 MHz, $CDCl_3$): δ = 7.75 (d, *J* = 7.3 Hz, 2H, Fmoc-H_{arom}), 7.53–7.63 (m, 2H, Fmoc-H_{arom}), 7.50 (d, *J* = 6.6 Hz, 1H, CHN<u>H</u>Asn/CHN<u>H</u>Phe), 7.35–7.43 (m, 2H, Fmoc-H_{arom}), 7.26–7.33 (m, 2H, Fmoc-H_{arom}), 7.09–7.23 (m, 7H, CHN<u>H</u>Asn/CHN<u>H</u>Phe, C-1-NH, Ph-H_{arom}), 5.76 (br. s., 2H, COOH, CHN<u>H</u>Fmoc), 5.29–5.39 (m, 4H, H-3, H-3‘, H-1‘), 5.17–5.27 (m, 1H, H-1), 5.01–5.10 (m, 1H, H-4‘), 4.78–4.89 (m, 2H, H-2, H-2‘), 4.65–4.77 (m, 2H, C<u>H</u>NHPhe, C<u>H</u>NHAsn), 4.33–4.44 (m, 2H, Fmoc-CH_2, H-6a), 4.13–4.33 (m, 5H, C<u>H</u>NHFmoc, Fmoc-CH, Fmoc-CH_2, H-6b, H-6a‘), 4.00–4.09 (m, 1H, H-6b‘), 3.86–3.96 (m, 2H, H-4, H-5‘), 3.65–3.76 (m, 1H, H-5), 3.16 (dd, *J* = 13.9 Hz, *J* = 5.4 Hz, 1H, Asn-CH_2), 2.94–3.00 (m, 1H, Asn-CH_2), 2.68–2.83 (m, 2H, Phe-CH_2), 2.07, 2.05, 2.02, 2.01, 1.99, 1.97, 1.95 (s, 21H, CH_3), 1.30 (d, *J* = 5.6 Hz, 3H, Ala-CH_3).

^{13}**C NMR** (101 MHz, $CDCl_3$): d = 173.3, 171.6, 171.5, 171.0, 170.9, 170.6, 170.1, 170.0, 169.5 (AcC=O, COOH, NHC=O), 155.8 (NHC=O), 143.7, 141.2, 135.8 ($C_{q,arom}$), 129.2 (Ph-C_{arom}), 128.6 (Ph-C_{arom}), 127.8 (Fmoc-C_{arom}), 127.1 (Fmoc-$C_{arom,}$ Phe-C_{arom}), 125.1, 120.0 (Fmoc-C_{arom}), 95.3 (C-1‘), 77.5 (C-1), 75.3 (C-3), 74.1 (C-5), 72.5 (C-5‘), 71.0 (C-2), 70.0 (C-2‘), 69.3 (C-3‘), 68.5 (C-4), 67.9 (C-4‘), 67.3 (Fmoc-CH_2), 62.5 (C-6), 61.4 (C-6‘), 54.6 (CHNHAsn), 50.5 (CHNHFmoc), 49.3 (CHNHPhe), 47.0 (Fmoc-CH), 37.5 (Asn-CH_2, Phe-CH_2), 20.8, 20.8, 20.7, 20.6 (CH_3), 18.4 (Ala-CH_3).

$[\alpha]_D^{20}$ = +29.7 (c = 0.5, $CHCl_3$). **Mass** Anal. Calcd for $C_{57}H_{66}N_4O_{24}$ $[M+Na]^+$: m/z 1213.39592; HRMS found $[M+Na]^+$: m/z 1213.39827.

***N*²-[*N*²[*N*²-[(9*H*-Fluoren-9-ylmethoxy)carbonyl]alaninyl]tryptophyl]-*N*-(2,3,4,6-tetra-*O*-acetyl-β-D-glucopyranosyl)-L-asparagine (9a)**

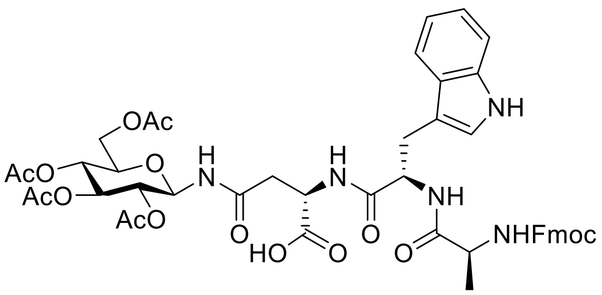

According to General Procedure E, **9a** was obtained from **7a** as a red solid. Yield: 99%.

^{1}**H NMR** (300 MHz, $CDCl_3$): δ = 8.49 (br. s., 1H, Trp-NH), 7.75 (d, J = 7.4 Hz, 2H, Fmoc-H_{arom}), 7.49–7.62 (m, 3H, Trp-H_{arom}, Fmoc-H_{arom}), 7.34–7.44 (m, 3H, Fmoc-H_{arom}, CHN<u>H</u>Asn), 7.26–7.32 (m, 3H, Fmoc-H_{arom}, CHN<u>H</u>Trp), 7.21 (d, J = 7.7 Hz, 1H, Trp-H_{arom}), 6.97–7.14 (m, 4H, Trp-H_{arom}, C-1-NH), 5.77 (d, J = 6.7 Hz, 1H, CHN<u>H</u>Fmoc), 5.22–5.31 (m,1H, H-3), 5.11–5.21 (m, 1H, H-1), 4.97–5.06 (m, 1H, H-4), 4.84–4.95 (m, 1H, H-2), 4.71–4.81 (m, 1H, C<u>H</u>NHTrp), 4.55–4.64 (m, 1H, C<u>H</u>NHAsn), 4.09–4.41 (m, 6H, Fmoc-CH, C<u>H</u>NHFmoc, Fmoc-CH_2, H-6a), 4.02 (dd, $J_{6b,6a}$ = 11.6 Hz, $J_{6b,5}$ = 0.6 Hz, 1H, H-6b),

3.65–3.75 (m, 1H, H-5), 3.14–3.31 (m, 2H, Trp-CH_2), 2.60 (dd, J = 15.2 Hz, J = 2.8 Hz, 1H, Asn-CH_2), 2.43–2.54 (m, 1H, Asn-CH_2), 2.00, 1.97, 1.96, 1.95 (s, 12H, CH_3), 1.26 (d, J = 9.1 Hz, 3H, Ala-CH_3).

^{13}C NMR (75 MHz, $CDCl_3$): δ = 173.5 (NHC=O), 172.2, 172.0, 171.5, 171.1, 171.0, 170.2, 169.7 (AcC=O, COOH, NHC=O), 156.3 (NHC=O), 143.7, 141.2, 136.1 ($C_{q,arom}$), 127.8 (Fmoc-C_{arom}), 127.4 ($C_{q,arom}$), 127.1, 125.1 (Fmoc-C_{arom}), 123.7, 122.1 (Trp-C_{arom}), 120.0 (Fmoc-C_{arom}), 119.5, 118.5, 111.5 (Trp-C_{arom}), 109.2 ($C_{q,arom}$), 77.9 (C-1), 73.6 (C-5), 72.8 (C-3), 70.5 (C-2), 68.1 (C-4), 67.4 (Fmoc-CH_2), 67.3, 61.7 (C-6), 54.1 (CHNHTrp), 50.6 (CHNHFmoc), 48.9 (CHNHAsn), 46.9 (Fmoc-CH), 36.9 (Asn-CH_2), 27.5 (Trp-CH_2), 20.6, 20.5 (CH_3), 18.2 (Ala-CH_3).

$[\alpha]_D^{20}$ = + 4.5 (c = 0.5, $CHCl_3$). **Mass** Anal. Calcd for $C_{47}H_{51}N_5O_{16}$ $[M+Na]^+$: m/z 964.32230; HRMS found $[M+Na]^+$: m/z 964.32188.

N^2-[N^2[N^2-[(9*H*-Fluoren-9-ylmethoxy)carbonyl]alaninyl]tryptophyl]-*N*-(2,3,4,6-tetra-*O*-acetyl-β-D-galactopyranosyl)-L-asparagine (9b)

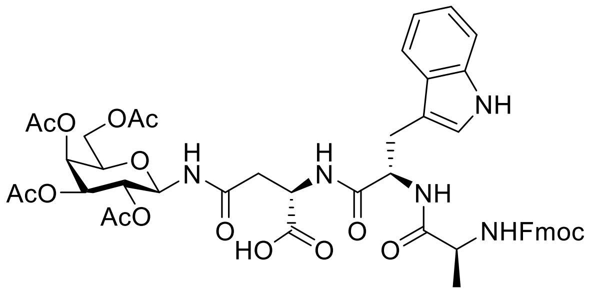

According to General Procedure E, **9b** was obtained from **7b** as a red solid. Yield: 99%.

^{1}H NMR (300 MHz, $CDCl_3$): δ = 8.50 (br. s., 1H, Trp-NH), 7.75 (d, J = 7.4 Hz, 2H, Fmoc-H_{arom}), 7.50–7.60 (m, 3H, Trp-H_{arom}, Fmoc-H_{arom}), 7.33–7.43 (m, CHN<u>H</u>Asn, CHN<u>H</u>Trp, Fmoc-H_{arom}), 7.29 (d, J = 6.8 Hz, 2H, H-Fmoc), 7.21 (br. s., 1H, Trp-H_{arom}), 6.93–7.13 (m, 5H,

Trp-H_{arom}, C-1-NH), 5.66 (d, *J* = 6.6 Hz, 1H, CHNHFmoc), 5.34–5.41 (s, 1H, H-4), 5.04–5.23 (m, 3H, H-1, H-2, H-3), 4.72–4.83 (m, 1H, CHNHTrp), 4.56–4.66 (m, 1H, CHNHAsn), 4.21–4.40 (m, 4H, CHNHFmoc, Fmoc-CH_2), 4.10–4.20 (m, 1H, Fmoc-CH), 3.89–4.09 (m, 3H, H-5, H-6a, H-6b), 3.68 (br.s., 1H, COOH), 3.16–3.30 (m, 2H, Trp-CH_2), 2.59–2.72 (m, 1H, Asn-CH_2), 2.45–2.58 (m, 1H, Asn-CH_2), 2.03, 1.96, 1.95, 1.94 (s, 12H, CH_3), 1.25–1.32 (m, 3H, Ala-CH_3).

^{13}C NMR (75 MHz, $CDCl_3$): δ = 173.1, 172.1, 171.8, 171.3, 170.9, 170.1 (AcC=O, COOH, NHC=O), 156.2 (NHC=O), 143.8, 143.7, 141.2, 136.1 ($C_{q,arom}$), 127.8 (Fmoc-C_{arom}), 127.4 ($C_{q,arom}$), 127.1, 125.1 (Fmoc-C_{arom}), 124.5, 122.0 (Trp-C_{arom}), 120.0 (Fmoc-C_{arom}), 119.5, 118.5, 111.5 (Trp-C_{arom}), 109.3 ($C_{q,arom}$), 78.2 (C-1), 72.2 (C-5), 70.9 (C-3), 68.3 (C-2), 67.3 (C-4, Fmoc-CH_2), 61.4 (C-6), 61.2, 54.1 (CHNHTrp), 50.5 (CHNHFmoc), 48.9 (CHNHAsn), 47.0 (Fmoc-CH), 37.0 (Asn-CH_2), 27.7 (Trp-CH_2), 20.6, 20.5, 20.4 (CH_3), 18.4 (Ala-CH_3).

$[\alpha]_D^{20}$ = + 7.7 (c = 0.5, $CHCl_3$). **Mass** Anal. Calcd for $C_{47}H_{51}N_5O_{16}$ $[M+Na]^+$: m/z 964.32230; HRMS found $[M+Na]^+$: m/z 964.32289.

N^2-[N^2[N^2-[(9*H*-Fluoren-9-ylmethoxy)carbonyl]alaninyl]tryptophyl]-*N*-(2,3,4,6-tetra-*O*-acetyl-β-D-mannopyranosyl)-L-asparagine (9c)

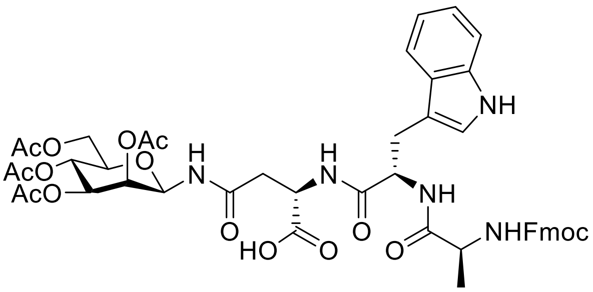

According to General Procedure E, **9c** was obtained from **7c** as a red solid. Yield: 99%.

^{1}H NMR (400 MHz, $CDCl_3$): δ = 8.61 (br. s., 1H, Trp-NH), 7.68–7.76 (m, 3H, Fmoc-H_{arom}, CHNHAsn/CHNHTrp/C-1-NH), 7.49–7.61 (m, 3H, Trp-H_{arom}, Fmoc-H_{arom}), 7.32–7.40 (m, 3H, Fmoc-H_{arom}, CHNHAsn/CHNHTrp/C-1-NH), 7.26–7.31 (m, 2H, Fmoc-H_{arom}), 7.18–7.23 (m, 2H, CHNHAsn/CHNHTrp/C-1-NH, Trp-H_{arom}), 6.91–7.08 (m, 3H, Trp-H_{arom}), 5.63–6.19 (m, 2H, CHNHFmoc, COOH), 5.39–5.51 (m, 1H, H-1), 5.29–5.38 (m, 1H, H-2), 5.08–5.24 (m, 2H, H-3, H-4), 4.71–4.86 (m, 1H, CHNHTrp), 4.50–4.66 (m, 1H, CHNHAsn), 4.09–4.42 (m, 5H, CHNHFmoc, Fmoc-CH_2, Fmoc-CH, H-6a), 3.95–4.08 (m, 1H, H-6b), 3.63–3.77 (m, 1H, H-5), 3.09–3.30 (m, 2H, Trp-CH_2), 2.42–2.81 (m, 2H, Asn-CH_2), 2.11, 1.99, 1.95 (s, 12H, CH_3), 0.86–0.91 (m, 3H, Ala-CH_3).

^{13}C NMR (101 MHz, $CDCl_3$): δ = 173.1, 171.9, 171.0, 170.4, 169.7 (AcC=O, COOH, NHC=O), 156.3 (NHC=O), 143.7, 141.2, 136.1 ($C_{q,arom}$), 127.8 (Fmoc-C_{arom}), 127.4 ($C_{q,arom}$), 127.1, 125.1 (Fmoc-C_{arom}), 123.7, 121.9 (Trp-C_{arom}), 120.0 (Fmoc-C_{arom}), 119.4, 118.5, 111.4 (Trp-C_{arom}), 109.3 ($C_{q,arom}$), 76.3 (C-1), 73.8 (C-5), 71.6 (C-3), 69.6 (C-2), 67.2 (Fmoc-CH_2), 65.3 (C-4), 62.2 (C-6), 54.0 (CHNHTrp), 50.5 (CHNHFmoc), 49.0 (CHNHAsn), 46.9 (Fmoc-CH), 37.1 (Asn-CH_2), 26.7 (Trp-CH_2), 20.8, 20.6, 20.5 (CH_3), 14.1 (Ala-CH_3).

$[\alpha]_D^{20}$ = – 1.5 (c = 0.5, $CHCl_3$). **Mass** Anal. Calcd for $C_{47}H_{51}N_5O_{16}$ $[M+Na]^+$: m/z 964.32230; HRMS found $[M+Na]^+$: m/z 964.32143.

N^2-[N^2[N^2-[(9*H*-Fluoren-9-ylmethoxy)carbonyl]alaninyl]tryptophyl]-*N*-(2,2',3,3',4,6,6'-hepta-*O*-acetyl-β-D-cellobiosyl)-L-asparagine (9d)

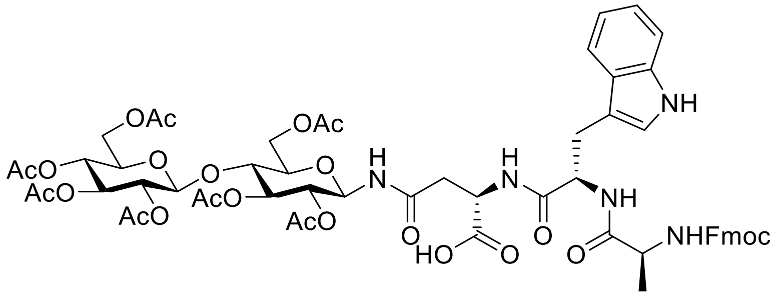

According to General Procedure E, **9d** was obtained from **7d** as a red solid. Yield: 99%.

^{1}H NMR (400 MHz, $CDCl_3$): δ = 8.58 (br. s., 1H, Trp-NH), 7.75 (d, *J* = 7.5 Hz, 2H, Fmoc-H_{arom}), 7.50–7.61 (m, 3H, Fmoc-H_{arom}, Trp-H_{arom}), 7.34–7.47 (m, 3H, C<u>H</u>NHAsn, Fmoc-H_{arom}), 7.28 (m, 3H, CHN<u>H</u>Trp, Fmoc-H_{arom}), 7.18–7.24 (m, 1H, Trp-H_{arom}), 6.96–7.13 (m, 4H, C-1-NH, Trp-H_{arom}), 5.73–5.84 (m, 1H, CHN<u>H</u>Fmoc), 5.58 (br. s., 1H, COOH), 5.18– 5.26 (m, 1H, H-3), 5.08–5.17 (m, 2H, H-1, H-3'), 4.99–5.07 (m, 1H, H-4'), 4.80–4.93 (m, 2H, H-2, H-2'), 4.72–4.80 (m, 1H, C<u>H</u>NHTrp), 4.53–4.63 (m, 1H, C<u>H</u>NHAsn), 4.37–4.48 (m, 2H, H-1', H-6a), 4.21–4.36 (m, 4H, H-6b, C<u>H</u>NHFmoc, Fmoc-CH_2), 4.12–4.20 (m, 1H, Fmoc-CH), 4.01–4.10 (m, 1H, H-6a'), 3.90–3.99 (m, 1H, H-6b'), 3.53–3.72 (m, 3H, H-4, H-5, H-5'), 3.14–3.29 (m, 2H, Trp-CH_2), 2.57–2.70 (m, 1H, Asn-CH_2), 2.41–2.51 (m, 1H, Asn-CH_2), 2.03, 1.99, 1.98, 1.97, 1.94 (s, 21H, CH_3), 1.25–1.31 (m, 3H, Ala-CH_3).

^{13}C NMR (101 MHz, $CDCl_3$): δ = 174.2, 173.3, 171.9, 171.5, 171.2, 170.7, 170.6, 170.3, 169.9, 169.4, 169.3 (AcC=O, COOH, NHC=O), 156.3 (NHC=O), 143.7, 141.2, 136.1 ($C_{q,arom}$), 127.8 (Fmoc-C_{arom}), 127.4 ($C_{q,arom}$), 127.1, 125.1 (Fmoc-C_{arom}), 123.7, 122.0 (Trp-C_{arom}), 120.0 (Fmoc-C_{arom}), 119.5, 118.5, 111.5 (Trp-C_{arom}), 109.3 ($C_{q,arom}$), 100.7 (C-1'), 77.8 (C-1), 76.2 (C-5), 74.6 (C-5'), 72.8 (C-3'), 72.5 (C-3), 71.8 (C-4), 71.5 (C-2'), 70.6 (C-2), 67.7 (C-4'), 67.3 (Fmoc-CH_2), 61.5 (C-6', C-6), 54.1 (CHNHTrp), 50.5 (CHNHAsn), 49.0 (CHNHFmoc), 46.9 (Fmoc-CH), 37.0 (Asn-CH_2), 29.7 (Trp-CH_2), 20.7, 20.6, 20.5 (CH_3), 18.4 (Ala-CH_3).

$[\alpha]_D^{20}$ = – 0.2 (c = 0.5, $CHCl_3$). **Mass** Anal. Calcd for $C_{59}H_{67}N_5O_{24}$ [M+Na]$^+$: m/z 1252.40682; HRMS found [M+Na]$^+$: m/z 1252.40764 .

***N*²-[*N*²[*N*²-[(9*H*-Fluoren-9-ylmethoxy)carbonyl]alaninyl]tryptophyl]-*N*-(2,2',3,3',4,6,6'-hepta-*O*-acetyl-β-D-lactosyl)-L-asparagine (9e)**

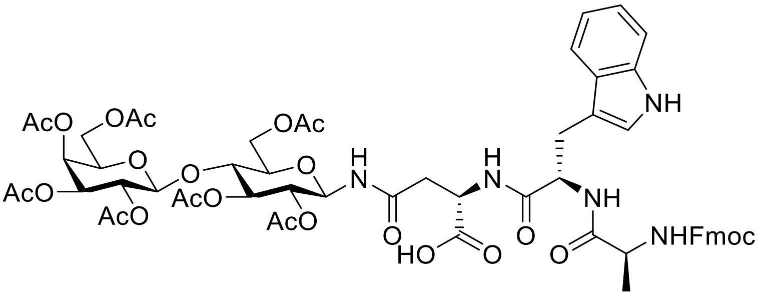

According to General Procedure E, **9e** was obtained from **7e** as a red solid. Yield: 99%.

¹H NMR (400 MHz, $CDCl_3$): δ = 8.51 (br. s., 1H, Trp-NH), 7.75 (d, *J* = 7.6 Hz, 2H, Fmoc-H_{arom}), 7.51–7.60 (m, 3H, Trp-$H_{arom,}$ Fmoc-H_{arom}), 7.39 (m, 3H, CHNHAsn, Fmoc-H_{arom}), 7.29 (d, *J* = 5.4 Hz, 3H, CHNHTrp, Fmoc-H_{arom}), 7.19–7.24 (m, 1H, Trp-H_{arom}), 6.98–7.12 (m, 4H, Trp-H_{arom}, C-1-NH), 5.66–5.93 (m, 2H, COOH, CHNHFmoc), 5.30–5.34 (d, *J* = 3.3 Hz, 1H, H-4‘), 5.19–5.26 (m, 1H, H-3), 5.04–5.15 (m, 2H, H-1, H-2‘), 4.93 (dd, $J_{3',4'}$ = 10.4 Hz, $J_{3',2'}$ = 3.4 Hz, 1H, H-3‘), 4.80–4.87 (m, 1H, H-2), 4.72–4.79 (m, 1H, CHNHTrp), 4.45–4.62 (m, 1H, CHNHAsn), 4.30–4.45 (m, 3H, H-1‘, H-6a, Fmoc-CH_2), 4.23–4.30 (m, 2H, Fmoc-CH_2, CHNHFmoc), 4.12–4.19 (m, 1H, Fmoc-CH), 3.98–4.12 (m, 3H, H-6b, H-6a‘, H-6b‘), 3.78–3.85 (m, 1H, H-5‘), 3.66–3.74 (m, 1H, H-4), 3.58–3.65 (m, 1H, H-5), 3.23 (m, 2H, Trp-CH_2), 2.59–2.69, 2.40–2.49 (m, 1H, Asn-CH_2), 2.12, 2.02, 2.00, 1.99, 1.96 (s, 21H, CH_3), 1.95 (br. s., 2H, H-CH3), 1.28 (d, *J* = 6.4 Hz, 3H, Ala-CH_3).

¹³C NMR (101 MHz, $CDCl_3$): δ = 173.5, 172.0, 171.9, 171.5, 171.2, 170.9, 170.5, 170.2, 170.2, 169.9, 169.3 (AcC=O, COOH, NHC=O), 156.3 (NHC=O), 143.7, 141.2, 136.1 ($C_{q,arom}$), 127.8 (Fmoc-C_{arom}), 127.4 ($C_{q,arom}$), 127.1, 125.1 (Fmoc-C_{arom}), 123.6, 122.1 (Trp-C_{arom}), 120.1

(Fmoc-C_{arom}), 119.5, 118.5, 111.5 (Trp-C_{arom}), 109.2 ($C_{q,arom}$), 100.9 (C-1‘), 77.7 (C-1), 75.8 (C-4), 74.7 (C-5), 72.6 (C-3), 70.9, 70.7, 70.6 (C-3‘, C-5‘, C-2), 69.0 (C-2‘), 67.3 (Fmoc-CH_2), 66.5 (C-4‘), 61.8 (C-6), 60.6 (C-6‘), 54.1 (CHNHTrp), 50.5 (CHNHFmoc), 49.0 (CHNHAsn), 46.9 (Fmoc-CH), 37.0 (Asn-CH_2), 27.5 (Trp-CH_2), 20.7, 20.7, 20.6, 20.5 (CH_3), 18.2 (Ala-CH_3).

$[\boldsymbol{\alpha}]_{\mathbf{D}}^{\mathbf{20}}$ = + 1.5 (c = 0.25, $CHCl_3$). **Mass** Anal. Calcd for $C_{59}H_{67}N_5O_{24}$ $[M+Na]^+$: m/z 1252.40682; HRMS found $[M+Na]^+$: m/z 1252.40520.

***N*²-[*N*²[*N*²-[(9*H*-Fluoren-9-ylmethoxy)carbonyl]alaninyl]tryptophyl]-*N*-(2,2’,3,3’,4,6,6’-hepta-*O*-acetyl-β-D-maltosyl)-L-asparagine (9f)**

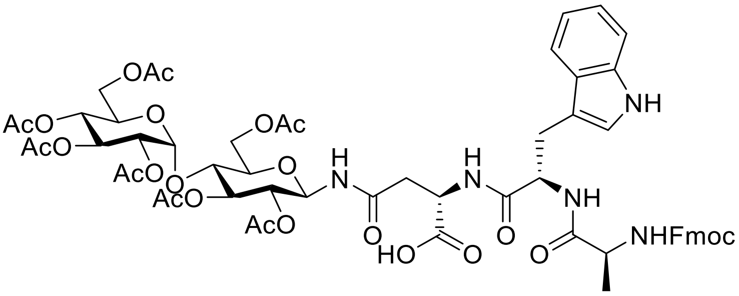

According to General Procedure E, **9f** was obtained from **7f** as a red solid. Yield: 99%.

¹H NMR (400 MHz, $CDCl_3$): δ = 8.56 (br. s., 1H, Trp-NH), 7.76 (d, *J* = 7.5 Hz, 2H, Fmoc-H_{arom}), 7.56 (d, *J* = 7.3 Hz, 3H, Trp-H_{arom}, Fmoc-H_{arom}), 7.32–7.43 (m, 3H, CHN<u>H</u>Asn, Fmoc-H_{arom}), 7.26–7.30 (m, 2H, Fmoc-H_{arom}), 7.22 (br. s., 2H, Trp-$H_{arom,}$ CHN<u>H</u>Trp), 7.07–7.13, 7.00–7.06 (m, 3H, Trp-H_{arom}), 6.90–6.98 (m, 1H, C-1-NH), 5.66–5.75 (m, 1H, CHN<u>H</u>Fmoc), 5.33–5.40 (m, 2H, H-1‘, H-3‘), 5.24–5.31 (m, 1H, H-3), 5.10–5.16 (m, 1H, H-1), 5.02–5.09 (m, 1H, H-4‘), 4.84 (dd, $J_{2‘,3‘}$ = 10.6 Hz, $J_{2‘,1‘}$ = 4.0 Hz, 3H, H-2‘), 4.68–4.80 (m, 2H, H-2, C<u>H</u>NHTrp), 4.60 (m, 1H, C<u>H</u>NHAsn), 4.30–4.43 (m, 2H, H-6a, Fmoc-CH_2), 4.24–4.30 (m, 2H, Fmoc-CH_2, C<u>H</u>NHFmoc), 4.21 (dd, $J_{6a‘,6b‘}$ = 12.5 Hz, $J_{6a‘,5‘}$ = 3.2 Hz, 2H,

H-6a‘), 4.13–4.18 (m, 1H, Fmoc-CH), 4.10 (dd, $J_{6b,6a}$ = 12.4 Hz, $J_{6b,5}$ = 3.3 Hz, 1H, H-6b), 4.00–4.07 (m, 1H, H-6b‘), 3.84–3.93 (m, 2H, H-4, H-5‘), 3.51–3.64 (br. s., 1H, H-5), 3.16–3.33 (m, 2H, Trp-CH_2), 2.56–2.68 (m, 1H, Asn-CH_2), 2.35–2.46 (m, 1H, Asn-CH_2), 2.07, 2.03, 2.02, 2.01, 2.00, 1.97, 1.94 (s, 21H, CH_3), 1.27–1.33 (m, 3H, Ala-CH_3).

^{13}C NMR (101 MHz, $CDCl_3$): δ = 173.3, 171.9, 171.7, 171.4, 170.9, 170.7, 170.6, 170.1, 169.5 (AcC=O, COOH, NHC=O), 156.3 (NHC=O), 143.7, 141.2, 136.1 ($C_{q,arom}$), 127.9 (Fmoc-C_{arom}), 127.4 ($C_{q,arom}$), 127.1, 125.1 (Fmoc-C_{arom}), 123.6, 122.1 (Trp-C_{arom}), 120.1 (Fmoc-C_{arom}), 119.6, 118.5, 111.5 (Trp-C_{arom}), 109.2 ($C_{q,arom}$), 95.6 (C-1‘), 77.5 (C-1), 75.2 (C-3), 74.0 (C-5), 72.3 (C-5‘), 71.1 (C-2), 70.0 (C-2‘), 69.3 (C-3‘), 68.5 (C-4), 67.9 (C-4‘), 67.3 (Fmoc-CH_2), 62.5 (C-6), 61.3 (C-6‘), 54.1 (CHNHTrp), 50.5 (CHNHFmoc), 48.9 (CHNHAsn), 47.0 (Fmoc-CH), 37.0 (Asn-CH_2), 27.6 (Trp-CH_2), 20.9, 20.8, 20.7, 20.6, 20.6, 20.5 (CH_3), 18.3 (Ala-CH_3).

$[\alpha]_D^{20}$ = +22.3 (c = 1, $CHCl_3$). **Mass** Anal. Calcd for $C_{59}H_{67}N_5O_{24}$ $[M+Na]^+$: m/z 1252.40682; HRMS found $[M+Na]^+$: m/z 1252.40616.

N^2**-[*N*^{2}[*N*^{2}Alaninyl]phenylalaninyl]-*N*-(β-D-glucopyranosyl)-L-asparagine (10a)**

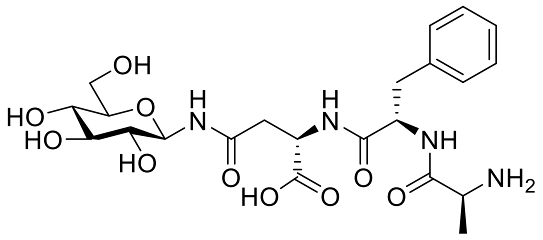

According to General Procedure F, **10a** was obtained from **8a** as a colorless solid. Yield: 98%.

^{1}H NMR (300 MHz, D_2O): δ = 7.25–7.39 (m, 5H, Ph-H_{arom}), 4.91 (d, $J_{1,2}$ = 9.1 Hz, 1H, H-1), 4.64 (dd, *J* = 9.4 Hz, *J* = 5.7 Hz, 1H, C<u>H</u>NHAsn), 4.41–4.48 (m, 1H, C<u>H</u>NHPhe), 3.96 (q, *J* = 7.2 Hz 1H, C<u>H</u>NH_2), 3.84 (dd, $J_{6a,6b}$ = 12.5 Hz, $J_{6a,5}$ = 2.1 Hz, 1H, H-6a), 3.68 (dd, $J_{6b,6a}$ = 12.4 Hz, $J_{6b,5}$ = 5.2 Hz, 1H, H-6b), 3.45–3.55 (m, 2H, H-3, H-5), 3.32–3.42 (m, 2H,

H-2, H-4), 3.21 (dd, J = 14.2 Hz, J = 5.8 Hz, 1H, Asn-CH_2), 2.99 (dd, J = 14.1 Hz, J = 9.5 Hz, 1H, Asn-CH_2), 2.74–2.82 (m, 2H, Phe-CH_2), 1.48 (d, J = 7.2 Hz, 3H, Ala-CH_3).

^{13}C NMR (101 MHz, $CDCl_3$): δ = 176.4, 175.6, 173.9, 172.0 (NHC=O, COOH), 136.6 ($C_{q,arom}$), 129.2, 128.7, 127.1 (Ph-C_{arom}), 79.2 (C-1), 77.5 (C-5), 76.4 (C-3), 71.8, 69.2 (C-2, C-4), 60.5 (C-6), 54.8 (CHNHAsn), 51.5 (CHNHPhe), 49.5 ($CHNH_2$), 38.0 (Phe-CH_2), 36.8 (Asn-CH_2), 18.4 (Ala-CH_3).

$[\alpha]_D^{20}$ = – 0.5 (c = 0.1, H_2O). **Mass** Anal. Calcd for $C_{22}H_{32}N_4O_{10}$ $[M+H]^+$: m/z 513.21912; HRMS found $[M+H]^+$: m/z 513.21934.

***N*²-[*N*²[*N*²Alaninyl]phenylalaninyl]-*N*-(β-D-galactopyranosyl)-L-asparagine (10b)**

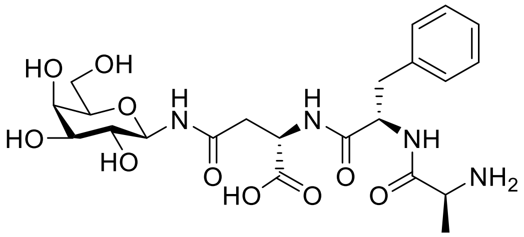

According to General Procedure F, **10b** was obtained from **8b** as a colorless solid. Yield: 96%.

^{1}H NMR (400 MHz, D_2O): δ = 7.23–7.40 (m, 5H, H-Ph), 4.86 (d, $J_{1,2}$ = 8.9 Hz, 1H, H-1), 4.66 (dd, J = 9.3 Hz, J = 6.0 Hz, 1H, C$\underline{H}$NHAsn), 4.37–4.46 (m, 1H, C$\underline{H}$NHPhe), 3.89–3.99 (m, 1H, H-3, C$\underline{H}NH_2$), 3.63–3.78 (m, 4H, H-4, H-5, H-6a, H-6b), 3.56–3.63 (m, 1H, H-2), 3.22 (dd, J = 13.6 Hz, J = 5.0 Hz, 1H, Asn-CH_2), 2.98 (dd, J = 13.6 Hz, J = 9.8 Hz, 1H, Asn-CH_2), 2.73–2.82 (m, 2H, Phe-CH_2), 1.46 (dd, J = 7.0 Hz, 3H, Ala-CH_3).

^{13}C NMR (101 MHz, D_2O): δ = 176.4, 173.9, 171.7, 170.6 (NHC=O, COOH), 136.6 ($C_{q,arom}$), 129.2, 128.7, 127.1 (Ph-C_{arom}), 79.7 (C-1), 76.7 (C-5), 73.3, 69.3 (C-2, C-4), 68.6 (C-3), 61.0

(C-6), 55.2 (CHNHAsn), 51.5 (CHNHPhe), 48.9 ($CHNH_2$), 37.9 (Phe-CH_2), 36.6 (Asn-CH_2), 16.5 (Ala-CH_3).

$[\alpha]_D^{20}$ = + 3.5 (c = 0.1, H_2O). **Mass** Anal. Calcd for $C_{22}H_{32}N_4O_{10}$ $[M+H]^+$: m/z 513.21912; HRMS found $[M+H]^+$: m/z 513.21292.

*N*²-[*N*²[*N*²Alaninyl]phenylalaninyl]-*N*-(β-D-mannopyranosyl)-L-asparagine (10c)

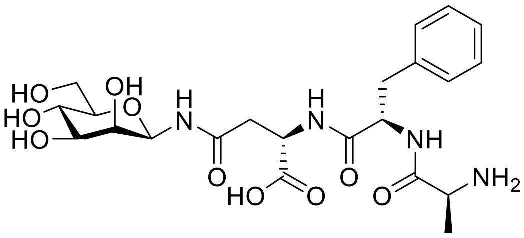

According to General Procedure F, **10c** was obtained from **8c** as a colorless solid. Yield: 66%.

¹H NMR (300 MHz, D_2O): δ = 7.32–7.40 (m, 2H, Ph-H_{arom}), 7.25–7.32 (m, 3H, Ph-H_{arom}), 5.16 (d, $J_{1,2}$ = 1.0 Hz, 1H, H-1), 4.65 (dd, J = 9.5 Hz, J = 5.5 Hz, 1H, C<u>H</u>NHAsn), 4.38–4.45 (m, 1H, C<u>H</u>NHPhe), 3.92–3.99 (m, 1H, C<u>H</u>NH_2), 3.90 (dd, $J_{2,3}$ = 3.3 Hz, $J_{2,1}$ = 1.0 Hz, 1H, H-2), 3.86 (dd, $J_{6a,6b}$ = 12.5 Hz, $J_{6a,5}$ = 2.3 Hz, 1H, H-6a), 3.67–3.73 (m, 1H, H-6b), 3.63–3.67 (m, 1H, H-3), 3.52–3.61 (m, 1H, H-4), 3.38–3.46 (m, 1H, H-5), 3.22 (dd, J = 14.2 Hz, J = 5.7 Hz, Asn-CH_2), 2.99 (dd, J = 14.0 Hz, J = 9.5 Hz, 1H, Asn-CH_2), 2.78 (dd, J = 6.3 Hz, J = 3.1 Hz, 2H, Phe-CH_2), 1.46 (d, J = 7.2 Hz, 3H, Ala-CH_3).

¹³C NMR (75 MHz, D_2O): δ = 176.6, 176.3, 173.2, 171.7 (NHC=O, COOH), 136.6 ($C_{q,arom}$), 129.1, 128.8, 127.1 (Ph-C_{arom}), 77.7 (C-1), 77.6 (C-5), 73.3 (C-3), 70.1 (C-2), 66.4 (C-4), 60.9 (C-6), 55.1 (CHNHAsn), 51.6 (CHNHPhe), 48.9 ($CHNH_2$), 37.8 (Phe-CH_2), 36.6 (Asn-CH_2), 16.5 (Ala-CH_3).

$[\alpha]_D^{20}$ = – 7.5 (c = 0.1, H_2O). **Mass** Anal. Calcd for $C_{22}H_{32}N_4O_{10}$ $[M+H]^+$: m/z 513.21912; HRMS found $[M+H]^+$: m/z 513.21974.

***N*²-[*N*²[*N*²Alaninyl]phenylalaninyl]-*N*-(β-D-cellobiosyl)-L-asparagine (10d)**

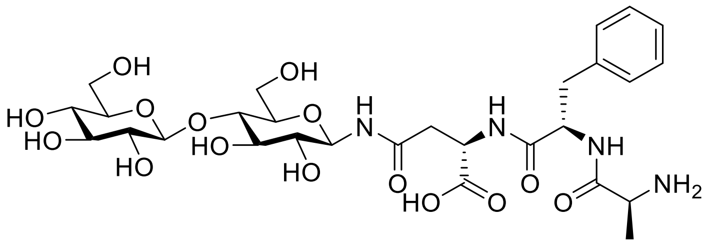

According to General Procedure F, **10d** was obtained from **8d** as a colorless solid. Yield: 61%.

^{1}H NMR (600 MHz, D_2O): δ = 7.33–7.38 (m, 2H, Ph-H_{arom}), 7.26–7.32(m, 3H, Ph-H_{arom}), 4.94 (dd, $J_{1,2}$ = 9.2 Hz, 1H, H-1), 4.65 (dd, *J* = 9.4 Hz, *J* = 5.7 Hz, 1H, C<u>H</u>NHAsn), 4.49 (d, $J_{1',2'}$ = 7.9 Hz, 1H, H-1‘), 4.39–4.45 (m, 1H, C<u>H</u>NHPhe), 3.95 (q, *J* = 6.9 Hz, 1H, C<u>H</u>NH_2), 3.86–3.92 (m, 2H, H-6a, H-6a‘), 3.78 ($J_{6b,6a}$ = 12.5 Hz, $J_{6b,5}$ = 3.7 Hz, 1H, H-6b), 3.71 ($J_{6b',6a'}$ = 12.3 Hz, $J_{6b',5'}$ = 5.7 Hz, 1H, H-6b‘), 3.60–3.68 (m, 3H, H-3, H-4, H-5), 3.44–3.50 (m, 2H, H-3‘, H-5‘), 3.37–3.44 (m, 2H, H-2, H-4‘), 3.27–3.32 (m, 1H, H-2‘), 3.22 (dd, *J* = 13.9 Hz, *J* = 5.3 Hz, 1H, Asn-CH_2), 2.99 (dd, *J* = 13.9 Hz, *J* = 9.2 Hz, 1H, Asn-CH_2), 2.73–2.82 (m, 2H, Phe-CH_2), 1.47 (d, *J* = 7.0 Hz, 3H, Ala-CH_3).

^{13}C NMR (151 MHz, D_2O): δ = 176.4, 173.9, 171.8, 170.7 (NHC=O, COOH), 136.5 ($C_{q,arom}$), 129.2, 128.8, 127.2 (Ph-C_{arom}), 102.5 (C-1‘), 79.1 (C-1), 78.1 (C-5), 76.3 (C-3), 76.0, 75.5 (C-3‘, C-5’), 74.9 (C-4), 73.1 (C-2’), 71.6 (C-2), 69.4 (C-4’), 60.6 (C-6’), 59.9 (C-6), 55.2 (CHNHAsn), 51.6 (CHNHPhe), 48.9 ($CHNH_2$), 38.1 (Phe-CH_2), 36.7 (Asn-CH_2), 16.5 (Ala-CH_3).

$[\alpha]_D^{20}$ = – 1.6 (c = 0.1, H_2O). **Mass** Anal. Calcd for $C_{28}H_{42}N_4O_{15}$ $[M+H]^+$: m/z 675.27194; HRMS found $[M+H]^+$: m/z 675.27215.

N^2-[N^2[N^2Alaninyl]phenylalaninyl]-*N*-(β-D-lactosyl)-L-asparagine (10e)

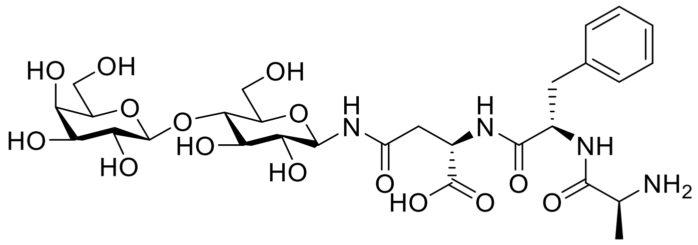

According to General Procedure F, **10e** was obtained from **8e** as a colorless solid. Yield: 80%.

^{1}H NMR (600 MHz, D_2O): δ = 7.33–7.39 (m, 2H, Ph-H_{arom}), 7.26–7.32 (m, 3H, Ph-H_{arom}), 4.90–4.99 (m, 1H, H-1), 4.61–4.69 (m, 1H, CHNHAsn), 4.39–4.47 (m, 2H, CHNHPhe, H-1‘), 3.85–3.93 (m, 3H, CHNH$_2$, H-4‘, H-6a), 3.72–3.81 (m, 2H, H-6b, H-6a‘), 3.60–3.72 (m, 6H, H-3, H-3‘, H-4, H-5, H-5‘, H-6b‘), 3.50–3.56 (m, 1H, H-2‘), 3.39–3.45 (m, 1H, H-2), 3.22 (dd, *J* = 13.7 Hz, *J* = 4.4 Hz, 1H, Asn-CH_2), 2.99 (dd, *J* = 12.5 Hz, *J* = 9.9 Hz, 1H, Asn-CH_2), 2.72–2.83 (m, 2H, Phe-CH_2), 1.42 (d, *J* = 6.2 Hz, 3H, Ala-CH_3).

^{13}C NMR (151 MHz, D_2O): δ = 178.9, 176.4, 174.4, 174.3 (NHC=O, COOH), 139.1 ($C_{q,arom}$), 131.7, 131.3, 129.7 (Ph-C_{arom}), 105.4 (C-1‘), 81.6 (C-1), 80.3 (C-5), 78.9, 77.9, 77.5, 75.0 (C-3, C-3‘, C-4, C-5‘), 74.1 (C-2), 73.5 (C-2‘), 71.1 (C-4‘), 63.6 (C-6‘), 62.4 (C-6), 57.7 (CHNHAsn), 54.1 (CHNHPhe), 51.3 (CHNH$_2$), 40.5 (Phe-CH_2), 39.3 (Asn-CH_2), 19.5 (Ala-CH_3).

$[\alpha]_D^{20}$ = – 0.5 (c = 0.1, H_2O). **Mass** Anal. Calcd for $C_{28}H_{42}N_4O_{15}$ $[M+H]^+$: m/z 675.27194; HRMS found $[M+H]^+$: m/z 675.27231.

N^2-[N^2[N^2Alaninyl]phenylalaninyl]-*N*-(β-D-maltosyl)-L-asparagine (10f)

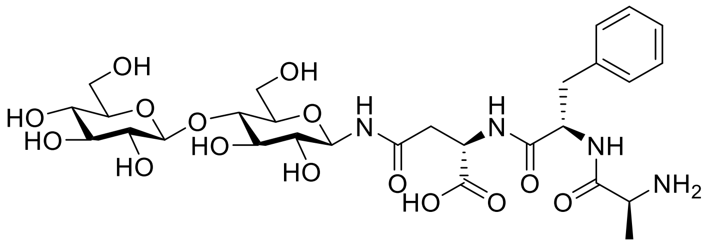

According to General Procedure F, **10f** was obtained from **8f** as a colorless solid. Yield: 99%.

^{1}H NMR (600 MHz, D_2O): δ = 7.34–7.38, 7.26–7.31 (m, 5H, Phe-H_{arom}), 5.39 (d, $J_{1',2'}$ = 3.9 Hz, 1H, H-1‘), 4.93 (d, $J_{1,2}$ = 9.2 Hz, 1H, H-1), 4.65 (dd, *J* = 9.5 Hz, *J* = 5.5 Hz, 1H, C<u>H</u>NHAsn), 4.40–4.44 (m, 1H, C<u>H</u>NHPhe), 3.82–3.87 (m, 4H, H-6a, C<u>H</u>NH_2), 3.78–3.82 (m, 2H, H-3, H-6b), 3.71–3.75 (m, 2H, H-6a‘, H-6b‘), 3.67–3.71 (m, 2H, H-5‘), 3.60 – 3.67 (m, 3H, H-4, H-5, H-3‘), 3.55 (dd, $J_{2',3'}$ = 9.9 Hz, $J_{2',1'}$ = 3.9 Hz, 1H, H-2‘), 3.37–3.42 (m, 2H, H-2, H-4‘), 3.22 (dd, *J* = 14.1 Hz, *J* = 5.5 Hz, 1H, Asn-CH_2), 2.99 (dd, *J* = 14.1 Hz, *J* = 9.7 Hz, 1H, Asn-CH_2), 2.80 (dd, *J* = 12.0 Hz, *J* = 1.7 Hz, 1H, Phe-CH_2), 2.76 (dd, *J* = 12.1 Hz, *J* = 2.9 Hz, 1H, Phe-CH_2), 1.40 (d, *J* = 7.2 Hz, 3H, Ala-CH_3).

^{13}C NMR (151 MHz, D_2O): δ = 176.4, 173.9, 172.2, 171.8 (NHC=O, COOH), 136.6 ($C_{q,arom}$), 129.2, 128.7, 127.1 (Phe-C_{arom}), 99.5 (C-1‘), 79.1 (C-1), 76.9 (C-3), 76.2, 76.1 (C-5, C-3‘/C-4), 72.8, 72.7 (C-5‘, C-3‘/C-4), 71.7 (C-2‘, C-2), 69.3 (C-4), 60.6 (C-6), 60.4 (C-6‘), 55.0 (CHNHAsn), 51.6 (CHNHPhe), 49.1 ($CHNH_2$), 37.9 (Phe-CH_2), 36.7 (Asn-CH_2), 17.1 (Ala-CH_3).

$[\alpha]_D^{20}$ = + 4.0 (c = 0.25, H_2O). **Mass** Anal. Calcd for $C_{28}H_{42}N_4O_{15}$ $[M+H]^+$: m/z 675.27194; HRMS found $[M+H]^+$: m/z 675.27076.

N^2-[N^2[N^2Alaninyl]tryptophyl]-*N*-(β-D-glucopyranosyl)-L-asparagine (11a)

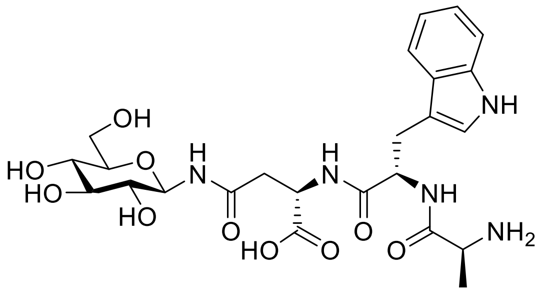

According to General Procedure F, **11a** was obtained from **9a** as a yellow solid. Yield: 92%.

^{1}H NMR (700 MHz, D_2O): δ = 7.64 (d, *J* = 8.0 Hz, 1H, Trp-H_{arom}), 7.46 (d, *J* = 8.1 Hz, 1H, Trp-H_{arom}), 7.23 (s, 1H, Trp-H_{arom}), 7.19–7.22, 7.11–7.15 (m, 2H, Trp-H_{arom}), 4.88 (d, $J_{1,2}$ = 9.0 Hz, 1H, H-1), 4.69 (dd, *J* = 8.2 Hz, *J* = 6.2 Hz, 1H, C<u>H</u>NHTrp), 4.31–4.35 (m, 1H, C<u>H</u>NHAsn), 3.94 (q, *J* = 7.1 Hz, 1H, C<u>H</u>NH_2), 3.84 (dd, $J_{6b,6a}$ = 12.3 Hz, $J_{6b,5}$ = 1.7 Hz, 1H, H-6b), 3.67 (dd, $J_{6a,6b}$ = 12.5 Hz, $J_{6a,5}$ = 5.6 Hz, 1H, H-6a), 3.46–3.51 (m, 1H, H-3, H-5), 3.33–3.39 (m, 1H, H-2, H-4), 3.31 (dd, *J* = 15.1 Hz, *J* = 6.0 Hz, 1H, Trp-CH_2), 3.23 (dd, *J* = 14.8 Hz, *J* = 8.4 Hz, 1H, Trp-CH_2), 2.68 (dd, *J* = 15.9 Hz, *J* = 6.7 Hz, 1H, Asn-CH_2), 2.54 (dd, *J* = 15.9 Hz, *J* = 4.7 Hz, 1H, Asn-CH_2), 1.46 (d, *J* = 7.1 Hz, 3H, Ala-CH_3).

^{13}C NMR (176 MHz, D_2O): δ = 176.2, 173.8, 172.0, 170.5 (NHC=O, COOH), 136.0, 126.8 ($C_{q,arom}$), 124.4, 121.9, 119.3, 118.3, 112.0 (Trp-C_{arom}), 108.9 ($C_{q,arom}$), 79.2 (C-1), 77.5 (C-5), 76.4 (C-3), 71.8, 69.3, 60.6 (C-6), 54.6 (CHNHTrp), 51.3 (CHNHAsn), 48.9 ($CHNH_2$), 37.7 (Asn-CH_2), 26.7 (Trp-CH_2), 16.5 (Ala-CH_3).

$[\boldsymbol{\alpha}]_{\mathbf{D}}^{\mathbf{20}}$ = – 2.6 (c = 0.5, H_2O). **Mass** Anal. Calcd for $C_{24}H_{33}N_5O_{10}$ $[M+H]^+$: m/z 552.23002; HRMS found $[M+H]^+$: m/z 552.23000.

N^2-[N^2[N^2Alaninyl]tryptophyl]-*N*-(β-D-galactopyranosyl)-L-asparagine (11b)

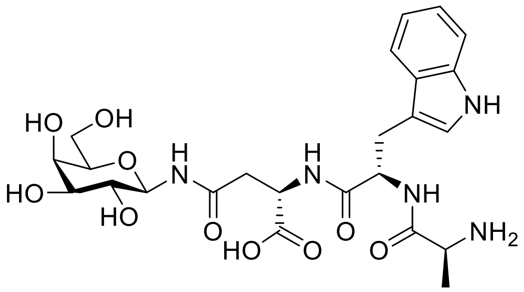

According to General Procedure F, **11b** was obtained from **9b** as a yellow solid. Yield: 99%.

^{1}H NMR (300 MHz, D_2O): δ = 7.65 (d, *J* = 7.8 Hz, 1H, Trp-H_{arom}), 7.48 (d, *J* = 8.1 Hz, 1H, Trp-H_{arom}), 7.24 (s, 1H, Trp-H_{arom}), 7.21 (dd, *J* = 8.2 Hz, *J* = 1.0 Hz, 1H, Trp-H_{arom}), 7.11–7.17 (m, 1H, Trp-H_{arom}), 4.84 (d, $J_{1,2}$ = 8.7 Hz, 1H, H-1), 4.66–4.73 (m, 1H, C<u>H</u>NHTrp), 4.31–4.37 (m, 1H, C<u>H</u>NHAsn), 3.89–3.99 (m, 2H, H-4, C<u>H</u>NH_2), 3.64–3.75 (m, 4H, H-3, H-5, H-6a, H-6b), 3.55–3.63 (m, 1H, H-2), 3.33 (dd, *J* = 15.2 Hz, *J* = 6.5 Hz, 1H, Trp-CH_2), 3.23 (dd, *J* = 15.0 Hz, *J* = 8.3 Hz, 1H, Trp-CH_2), 2.71 (dd, *J* = 15.9 Hz, *J* = 6.4 Hz, 1H, Asn-CH_2), 2.55 (dd, *J* = 15.8 Hz, *J* = 4.9 Hz, 1H, Asn-CH_2), 1.46 (d, *J* = 7.2 Hz, 3H, Ala-CH_3).

^{13}C NMR (75 MHz, D_2O): δ = 176.3, 173.9, 172.0, 170.6 (NHC=O, COOH), 136.1, 128.6, ($C_{q,arom}$), 124.5, 121.9, 119.3, 118.4, 112.0 (Trp-C_{arom}), 108.9 ($C_{q,arom}$), 79.6 (C-1), 76.7 (C-5), 73.3 (C-3), 69.4 (C-2), 68.7 (C-4), 61.0 (C-6), 54.7 (CHNHTrp), 52.5, 51.4 (CHNHAsn), 48.9 ($CHNH_2$), 37.7 (Asn-CH_2), 26.7 (Trp-CH_2), 16.5 (Ala-CH_3).

$[\alpha]_D^{20}$ = – 3.2 (c = 0.1, H_2O). **Mass** Anal. Calcd for $C_{24}H_{33}N_5O_{10}$ $[M+H]^+$: m/z 552.23002; HRMS found $[M+H]^+$: m/z 552.23046.

N^2-[N^2[N^2Alaninyl]tryptophyl]-*N*-(β-D-mannopyranosyl)-L-asparagine (11c)

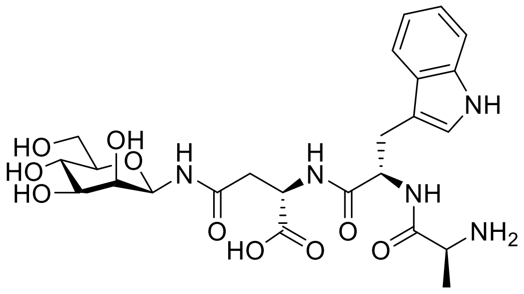

According to General Procedure F, **11c** was obtained from **9c** as a yellow solid. Yield: **77**%.

^{1}H NMR (400 MHz, D_2O): δ = 7.63 (d, *J* = 8.0 Hz, 1H, Trp-H_{arom}), 7.45 (d, *J* = 8.2 Hz, 1H, Trp-H_{arom}), 7.16–7.24 (m, 2H, Trp-H_{arom}), 7.08–7.15 (m, 1H, Trp-H_{arom}), 5.07 (d, $J_{1,2}$ = 0.9 Hz, 1H, H-1), 4.68 (dd, *J* = 8.2 Hz, *J* = 6.0 Hz, 1H, C<u>H</u>NHTrp), 4.32 (dd, *J* = 6.2 Hz, *J* = 4.9 Hz, 1H, C<u>H</u>NHAsn), 3.82 (s, 3H, H-2, H-6a C<u>H</u>NH_2), 3.66 (dd, $J_{6b,6a}$ = 12.2 Hz, $J_{6b,5}$ = 6.1 Hz, 1H, H-6b), 3.61 (dd, $J_{3,4}$ = 9.5 Hz, $J_{3,2}$ = 3.2 Hz, 1H, H-3), 3.50–3.56 (m, 1H, H-4), 3.39 (ddd, $J_{5,4}$ = 9.3 Hz, $J_{5,6b}$ = 6.0 Hz, $J_{5,6a}$ = 2.1 Hz, 1H, H-5), 3.30 (dd, *J* = 14.8 Hz, *J* = 5.8 Hz, 1H, Trp-CH_2), 3.21 (dd, *J* = 14.8 Hz, *J* = 8.2 Hz, 1H, Trp-CH_2), 2.69 (dd, *J* = 15.8 Hz, *J* = 6.6 Hz, 1H, Asn-CH_2), 2.54 (dd, *J* = 15.8 Hz, *J* = 4.8 Hz, 1H, Asn-CH_2), 1.40 (d, *J* = 7.1 Hz, 3H, Ala-CH_3).

^{13}C NMR (101 MHz, D_2O): δ = 176.3, 173.1, 172.0, 171.3 (NHC=O, COOH), 136.0, 126.8 ($C_{q,arom}$), 124.4, 121.9, 119.3, 118.3, 111.9 (Trp-C_{arom}), 108.9 ($C_{q,arom}$), 77.7 (C-1), 77.6 (C-5), 73.2 (C-3), 70.1 (C-2), 66.4 (C-4), 60.9 (C-6), 54.5 (CHNHTrp), 51.4 (CHNHAsn), 49.0 ($CHNH_2$), 37.6 (Asn-CH_2), 26.7 (Trp-CH_2), 16.7 (Ala-CH_3).

$[\alpha]_D^{20}$ = – 2.0 (c = 0.5, H_2O/acetone 1:1). **Mass** Anal. Calcd for $C_{24}H_{33}N_5O_{10}$ $[M+H]^+$: m/z 552.23002; HRMS found $[M+H]^+$: m/z 552.23026.

***N*[2]-[*N*[2][*N*[2]Alaninyl]tryptophyl]-*N*-(β-D-cellobiosyl)-L-asparagine (11d)**

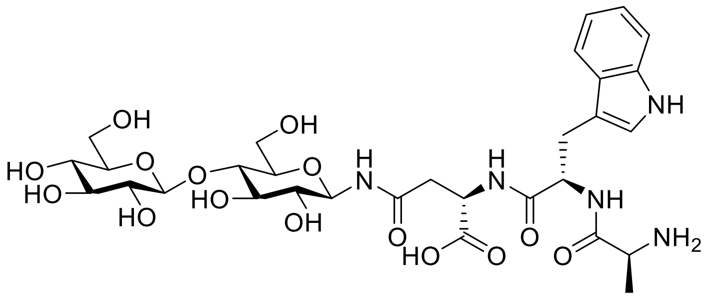

According to General Procedure F, **11d** was obtained from **9d** as a yellow solid. Yield: 49%.

^{1}H NMR (700 MHz, D_2O): δ = 7.62–7.67, 7.44–7.49 (m, 2H, Trp-H_{arom}), 7.23 (s, 1H, Trp-H_{arom}), 7.18–7.22, 7.10–7.16 (m, 2H, Trp-H_{arom}), 4.90 (d, $J_{1,2}$ = 9.3 Hz, 1H, H-1), 4.69 (dd, J = 8.2 Hz, J = 5.8 Hz, 1H, C<u>H</u>NHTrp), 4.46 (d, $J_{1',2'}$ = 8.0 Hz, 1H, H-1‘), 4.31–4.35 (m, 1H, C<u>H</u>NHAsn), 3.93 (q, J = 7.0 Hz, 1H, C<u>H</u>NH_2), 3.86–3.90 (m, 1H, H-6a, H-6a‘), 3.75 (dd, $J_{6b,6a}$ =12.3 Hz, $J_{6b',5'}$ = 4.1 Hz, 1H, H-6b), 3.69 (dd, $J_{6b',6a'}$ =12.5 Hz, $J_{6b',5'}$ = 6.0 Hz, 1H, H-6b‘), 3.60–3.63 (m, 1H, H-3, H-4, H-5), 3.43–3.46 (m, 1H, H-3‘, h-%‘), 3.36–3.40 (m, 1H, H-2‘, H-4‘), 3.31 (dd, J =15.1 Hz, J = 6.0 Hz, 1H, Trp-CH_2), 3.25–3.29 (m, 1H, H-2), 3.23 (dd, J = 14.8 Hz, J = 8.4 Hz, 1H, Trp-CH_2), 2.68 (dd, J =15.7 Hz, J = 6.7 Hz, 1H, Asn-CH_2), 2.57 (dd, J =15.7 Hz, J = 4.7 Hz, 1H, Asn-CH_2), 1.45 (d, J = 7.3 Hz, 3H, Ala-CH_3).

^{13}C NMR (176 MHz, D_2O): δ = 173.8, 172.0 (NHC=O), 135.9, 126.8 ($C_{q,arom}$), 124.4, 121.9, 119.3, 118.3, 111.8 (Trp-C_{arom}), 108.9 ($C_{q,arom}$), 102.5 (C-1‘), 79.0 (C-1), 78.1 (C-3), 76.3 (C-5), 76.0 (C-5‘), 75.4 (C-3‘), 74.9 (C-4), 73.1 (C-2‘), 71.6 (C-2), 69.4 (C-4‘), 60.5 (C-6‘), 59.9 (C-6), 54.6 (CHNHTrp), 51.4 (CHNHAsn), 48.9 ($CHNH_2$), 37.7 (Asn-CH_2), 26.7 (Trp-CH_2), 16.5 (Ala-CH_3).

$[\alpha]_D^{20}$ = – 1.2 (c = 0.1, H_2O). **Mass** Anal. Calcd for $C_{30}H_{43}N_5O_{15}$ $[M+H]^+$: m/z 714.28284; HRMS found $[M+H]^+$: m/z 714.28300.

N^2-[N^2[N^2Alaninyl]tryptophyl]-*N*-(β-D-lactosyl)-L-asparagine (11e)

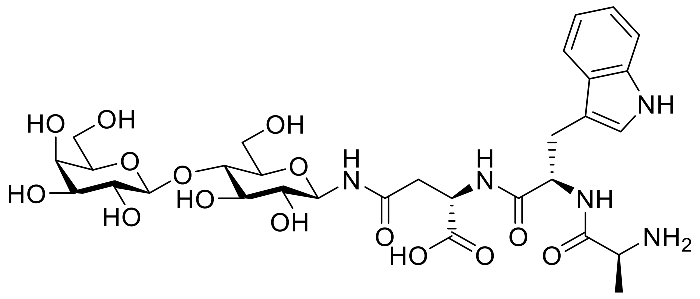

According to General Procedure F, **11e** was obtained from **9e** as a yellow solid. Yield: 99%.

^{1}H NMR (300 MHz, D_2O): δ = 7.63–7.70, 7.44–7.51 (m, 2H, Trp-H_{arom}), 7.24 (br. s., 1H, Trp-H_{arom}), 7.21 (dd, *J* = 8.3 Hz, *J* = 1.1 Hz, 1H, Trp-H_{arom}), 7.11–7.18 (m, 1H, Trp-H_{arom}), 4.92 (d, *J* = 9.2 Hz, 1H, H-1), 4.67–4.72 (m, 1H, C<u>H</u>NHTrp), 4.39–4.45 (d, $J_{1‘,2‘}$ = 7.6 Hz, 1H, H-1‘), 4.36 (dd, *J* = 6.1 Hz, *J* = 5.0 Hz, 1H, C<u>H</u>NHAsn), 3.82–3.95 (m, 3H, H-4‘, H-6a, C<u>H</u>NH_2), 3.69–3.81 (m, 4H, H-4, H-6b, H-6a‘, H-6b‘), 3.58–3.68 (m, 4H, H-5‘, H-5, H-3‘, H-3), 3.52 (dd, , $J_{2‘,3‘}$ = 9.9 Hz, $J_{2‘,1‘}$ = 7.7 Hz, 1H, H-2‘), 3.38–3.45 (m, 1H, H-2), 3.33 (dd, *J* =14.8 Hz, *J* = 5.5 Hz, 1H, Trp-CH_2), 3.23 (dd, *J* =14.9 Hz, *J* = 8.4 Hz, 1H, Trp-CH_2), 2.70 (dd, *J* =15.7 Hz, *J* = 6.7 Hz, 1H, Asn-CH_2), 2.59 (dd, *J* =15.7 Hz, *J* = 5.1 Hz, 1H, Asn-CH_2), 1.42 (d, *J* = 7.2 Hz, 3H, Ala-CH_3).

^{13}C NMR (75 MHz, D_2O): δ = 173.8, 172.1 (NHC=O, COOH), 136.1, 124.4 ($C_{q,arom}$), 121.9, 119.3, 118.4, 112.0, 109.0 (Trp-C_{arom}), 102.9 (C-1‘), 79.1 (C-1), 77.9 (C-5), 76.3, 75.3, 75.0, 72.5 (C-5‘, C-3, C-3‘, C-4), 71.5 (C-2‘), 70.9 (C-2), 68.5 (C-4‘), 61.0 (C-6‘), 60.0 (C-6), 54.6 (CHNHTrp), 51.4 (CHNHAsn), 49.1 ($CHNH_2$), 37.8 (Asn-CH_2), 26.8 (Trp-CH_2), 16.9 (Ala-CH_3).

$[\alpha]_D^{20}$ = + 2.3 (c = 0.25, H_2O). **Mass** Anal. Calcd for $C_{30}H_{43}N_5O_{15}$ $[M+H]^+$: m/z 714.28284; HRMS found $[M+H]^+$: m/z 714.28146.

***N*²-[*N*²[*N*²Alaninyl]tryptophyl]-*N*-(β-D-maltosyl)-L-asparagine (11f)**

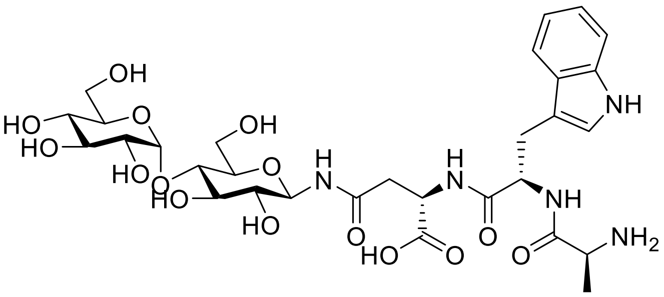

According to General Procedure F, **11f** was obtained from **9f** as a light yellow solid. Yield: 87%.

^{1}H NMR (400 MHz, D_2O): δ = 7.60–7.66, 7.43–7.48 (m, 2H, Trp-H_{arom}), 7.22 (br. s., 1H, Trp-H_{arom}), 7.17–7.21, 7.09–7.15 (m, 2H, Trp-H_{arom}), 5.33–5.38 (d, $J_{1‘,2‘}$ = 7.6 Hz, 1H, H-1‘), 4.88 (d, $J_{1,2}$ = 9.3 Hz, 1H, H-1), 4.68 (dd, *J* = 8.2 Hz, *J* = 6.0 Hz, 1H, C<u>H</u>NHTrp), 4.32 (dd, *J* = 6.1 Hz, *J* = 5.0 Hz, 1H, C<u>H</u>NHAsn), 3.94 (q, *J* = 7.1 Hz, 1H, C<u>H</u>NH_2), 3.73–3.86 (m, 3H, H-6a, H-6b, H-3), 3.58–3.73 (m, 6H, H-5, H-5‘, H-4, H-3‘, H-6a‘, H-6b‘), 3.52 (dd, $J_{2’,3’}$ = 9.9 Hz, $J_{2’,1’}$ = 3.9 Hz, 1H, H-2‘), 3.33–3.39 (m, 2H, H-2, H-4‘), 3.30 (dd, *J* =14.8 Hz, *J* = 5.9 Hz, 1H, Trp-CH_2), 3.22 (dd, *J* =14.9 Hz, *J* = 8.3 Hz, 1H, Trp-$CH_2$2.67 (dd, *J* =15.8 Hz, *J* = 6.7 Hz, 1H, Asn-CH_2), 2.53 (dd, *J* =15.8 Hz, *J* = 4.9 Hz, 1H, Asn-CH_2), 1.45 (d, *J* = 7.2 Hz, 3H, Ala-CH_3).

^{13}C NMR (101 MHz, D_2O): δ = 173.8, 172.0, 170.5 (NHC=O, COOH), 136.0, 126.8 ($C_{q,arom}$), 124.4, 121.9, 119.3, 118.3, 112.0 (Trp-C_{arom}), 108.9 ($C_{q,arom}$), 99.5 (C-1‘), 79.0 (C-1), 76.8 (C-3), 76.2, 76.1 (C-5, C-3‘/C-4), 72.8, 72.6 (C-5‘, C-3‘/C-4), 71.6 (C-2, C-2‘), 69.2 (C-4‘), 60.6 (C-6), 60.4 (C-6‘), 54.6 (CHNHAsn), 51.2 (CHNHTrp), 48.9 ($CHNH_2$), 37.7 (Asn-CH_2), 26.7 (Trp-CH_2), 16.4 (Ala-CH_3).

$[\alpha]_D^{20}$ = + 3.5 (c = 0.1, H_2O). **Mass** Anal. Calcd for $C_{30}H_{43}N_5O_{15}$ $[M+H]^+$: m/z 714.28284; HRMS found $[M+H]^+$: m/z 714.28206.

References

1. Esteves, A. P.; Rodrigues, L. M.; Silva, M. E.; Gupta, S.; Oliveira-Campos, A. M. F.; Machalicky, O.; Mendonça, A. J. *Tetrahedron* **2005**, *61*, 8625-8632.

2. Sirion, U.; Kim, H. J.; Lee, J. H.; Seo, J. W.; Lee, B. S.; Lee, S. J.; Oh, S. J.; Chi, D. Y. *Tetrahedron Lett.* **2007**, *48*, 3953-3957.

3. Salunke, S. B.; Babu, N. S.; Chen, C. T. *Chem. Commun. (Cambridge, U. K.)* **2011**, *47*, 10440-10442.

4. Berthold, H. J.; Franke, S.; Thiem, J.; Schotten, T. *J. Org. Chem.* **2010**, *75*, 3859-3862.

5. Porwanski, S.; Marsura, A. *Eur. J. Org. Chem.* **2009**, *2009*, 2047-2050.

6. Pető, C.; Batta, G.; Györgydeák, Z.; Sztaricskai, F. *Liebigs Ann. Chem.* **1991**, *1991*, 505-507.

7. Varamini, P.; Mansfeld, F. M.; Blanchfield, J. T.; Wyse, B. D.; Smith, M. T.; Toth, I. *J. Med. Chem.* **2012**, *55*, 5859-5867.

8. Park, J.; Rader, L. H.; Thomas, G. B.; Danoff, E. J.; English, D. S.; DeShong, P. *Soft Matter* **2008**, *4*.

9. Maier, M. A.; Yannopoulos, C. G.; Mohamed, N.; Roland, A.; Fritz, H.; Mohan, V.; Just, G.; Manoharan, M. *Bioconjugate Chem.* **2003**, *14*, 18-29.

10. Zheng, X.; Morgan, J.; Pandey, S. K.; Chen, Y.; Tracy, E.; Baumann, H.; Missert, J. R.; Batt, C.; Jackson, J.; Bellnier, D. A.; Henderson, B. W.; Pandey, R. K. *J. Med. Chem.* **2009**, *52*, 4306-4318.

11. Choudhury, A. K.; Kitaoka, M.; Hayashi, K. *Eur. J. Org. Chem.* **2003**, *2003*, 2462-2470.

12. Sansone, F.; Chierici, E.; Casnati, A.; Ungaro, R. *Org. Biomol. Chem.* **2003**, *1*, 1802-1809.

13. van Ameijde, J.; Albada, H. B.; Liskamp, R. M. J. *J. Chem. Soc., Perkin Trans. 1* **2002**, 1042-1049.

14. Moyle, P. M.; Olive, C.; Ho, M. F.; Pandey, M.; Dyer, J.; Suhrbier, A.; Fujita, Y.; Toth, I. *J. Med. Chem.* **2007**, *50*, 4721-4727.

15. Györgydeák, Z.; Paulsen, H. *Justus Liebigs Annalen der Chemie* **1977**, *1977*, 1987-1991.
