# Supplementary material for: Synthesis of new asparagine-based glycopeptides for future scanning tunneling microscopy investigations
Source: Beilstein J Org Chem. 2020 Apr 30;16:888–94. doi: 10.3762/bjoc.16.80 (PMC7214877; doi:10.3762/bjoc.16.80)
Supplement: File 2 — NMR spectra of the compounds 1a–f to 11a–f. [file Beilstein_J_Org_Chem-16-888-s002.pdf]

BEILSTEIN JOURNAL OF ORGANIC CHEMISTRY

Supporting Information

for

Synthesis of new asparagine-based glycopeptides for future scanning tunneling microscopy investigations

Laura Sršan and Thomas Ziegler

Beilstein J. Org. Chem. **2020,** *16,* 888–894. doi:10.3762/bjoc.16.80

NMR spectra of the compounds 1a–f to 11a–f

License and Terms: This is a supporting information file under the terms of the Creative Commons Attribution License (http://creativecommons.org/licenses/by/4.0). Please note that the reuse, redistribution and reproduction in particular requires that the authors and source are credited.
The license is subject to the *Beilstein Journal of Organic Chemistry* terms and conditions: (https://www.beilstein-journals.org/bjoc)

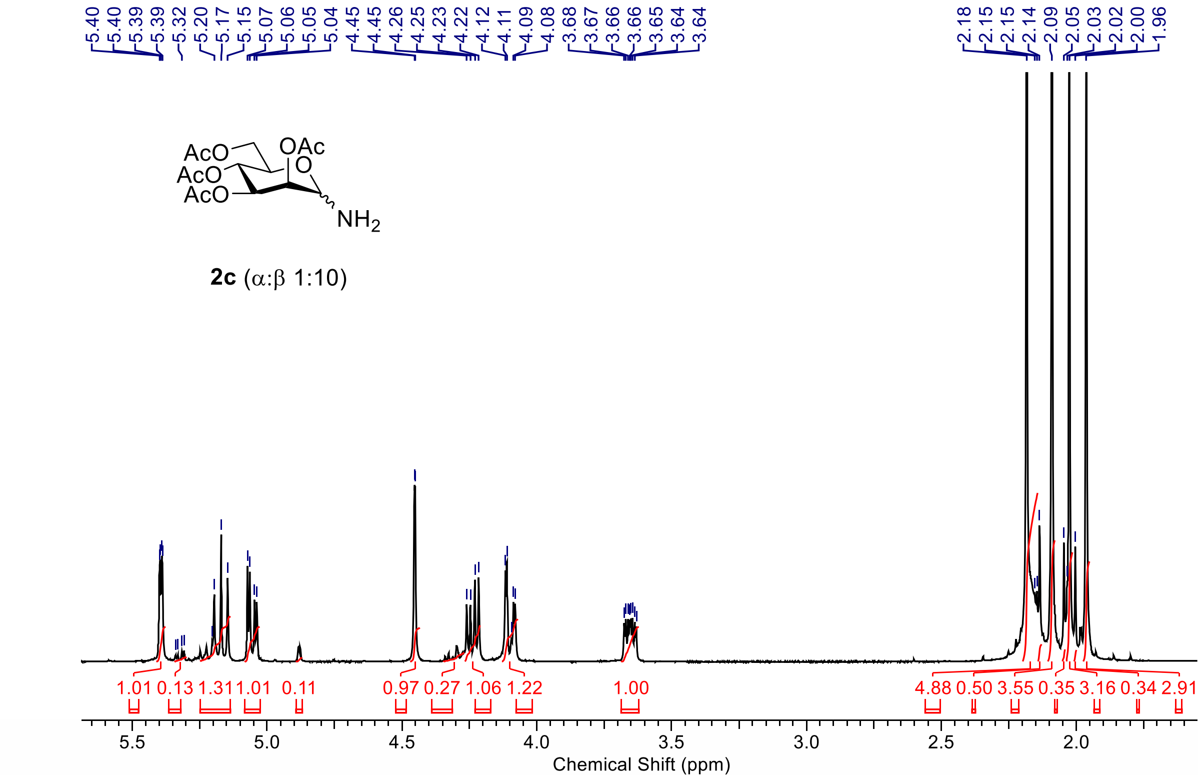
5.40 5.40 5.39 5.39 5.32 5.20 5.17 5.15 5.07 5.06 5.05 5.04 4.45 4.26 4.25 4.23 4.22 4.12 4.11 4.09 4.08 3.68 3.67 3.66 3.66 3.65 3.64 3.64
2.18 2.15 2.15 2.14 2.09 2.05 2.03 2.02 2.00 1.96
AcO
AcO
AcO
OAc
O
NH2
2c (α:β 1:10)
1.01 0.13 1.31 1.01 0.11 0.97 0.27 1.06 1.22 1.00 4.88 0.50 3.55 0.35 3.16 0.34 2.91
5.5 5.0 4.5 4.0 3.5 3.0 2.5 2.0
Chemical Shift (ppm)

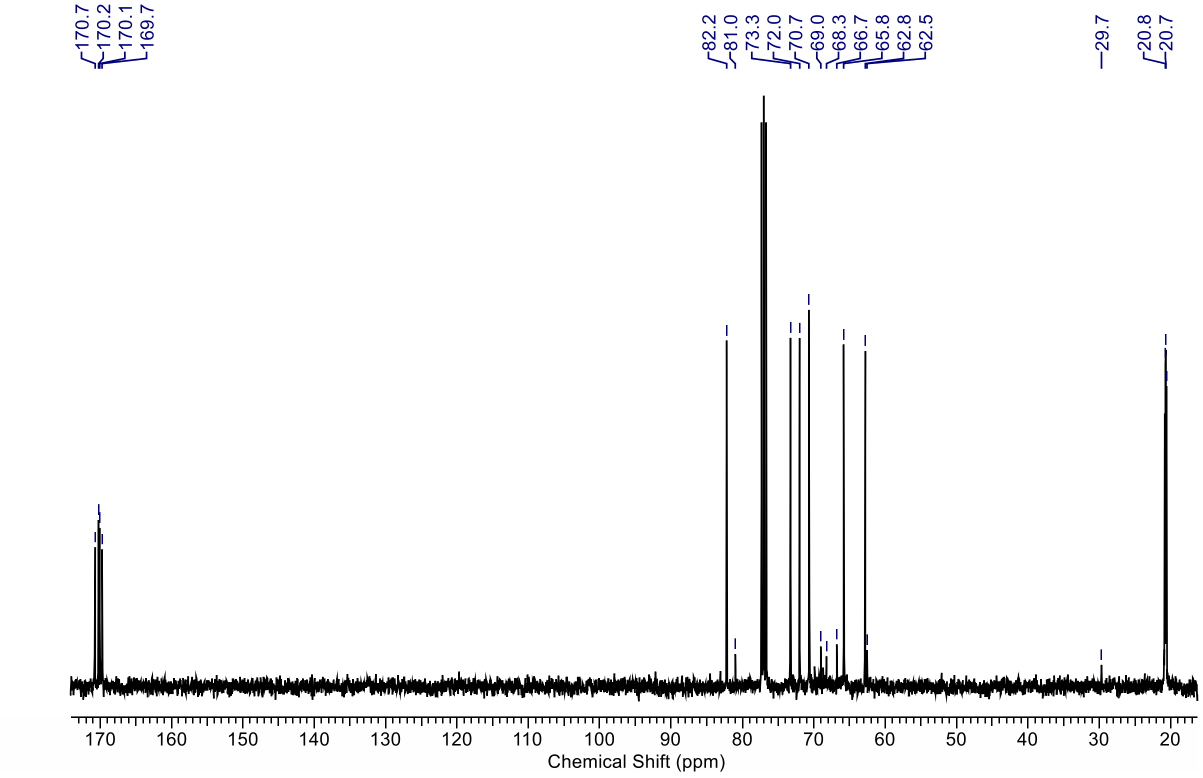
170.7 170.2 170.1 169.7
82.2 81.0 73.3 72.0 70.7 69.0 68.3 66.7 65.8 62.8 62.5
29.7
20.8 20.7
170 160 150 140 130 120 110 100 90 80 70 60 50 40 30 20
Chemical Shift (ppm)

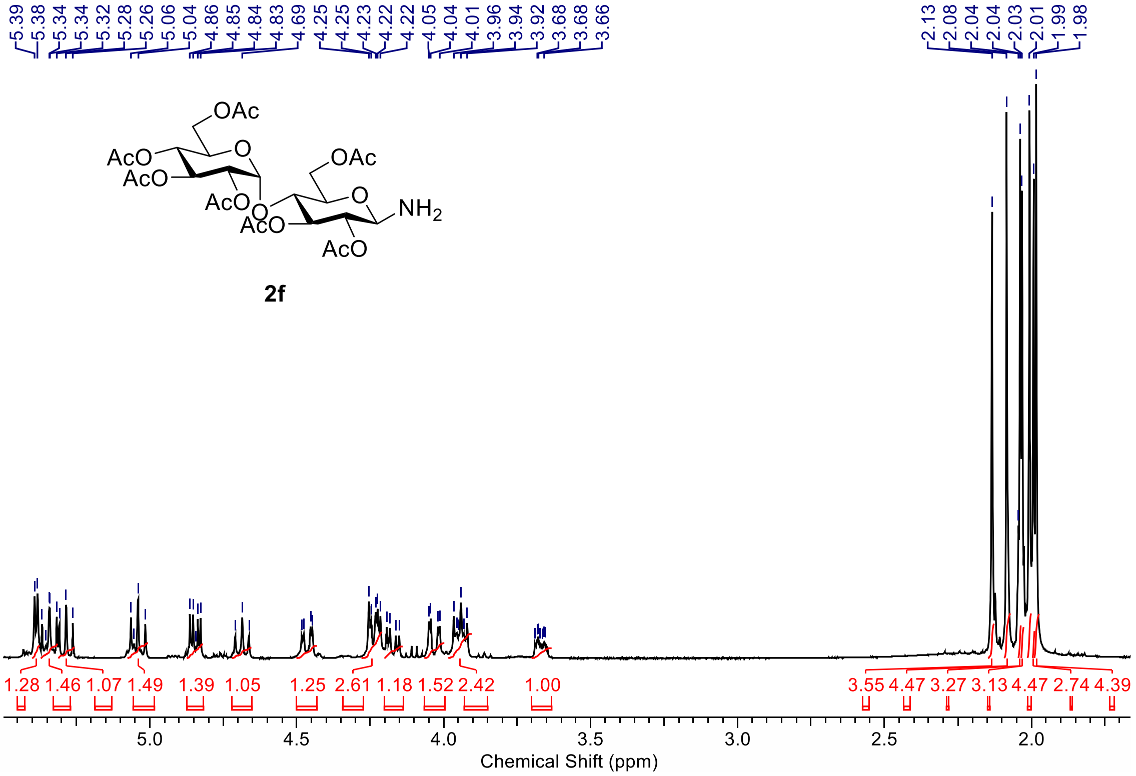
5.39 5.38 5.34 5.34 5.32 5.28 5.26 5.06 5.04 4.86 4.85 4.84 4.83 4.69 4.25 4.25 4.23 4.22 4.22 4.05 4.04 4.01 3.96 3.94 3.92 3.68 3.68 3.66
2.13 2.08 2.04 2.04 2.03 2.01 1.99 1.98
OAc
AcO
AcO
AcO
O
OAc
NH2
AcO
AcO
2f
1.28 1.46 1.07 1.49 1.39 1.05 1.25 2.61 1.18 1.52 2.42 1.00
3.55 4.47 3.27 3.13 4.47 2.74 4.39
5.0 4.5 4.0 3.5 3.0 2.5 2.0
Chemical Shift (ppm)

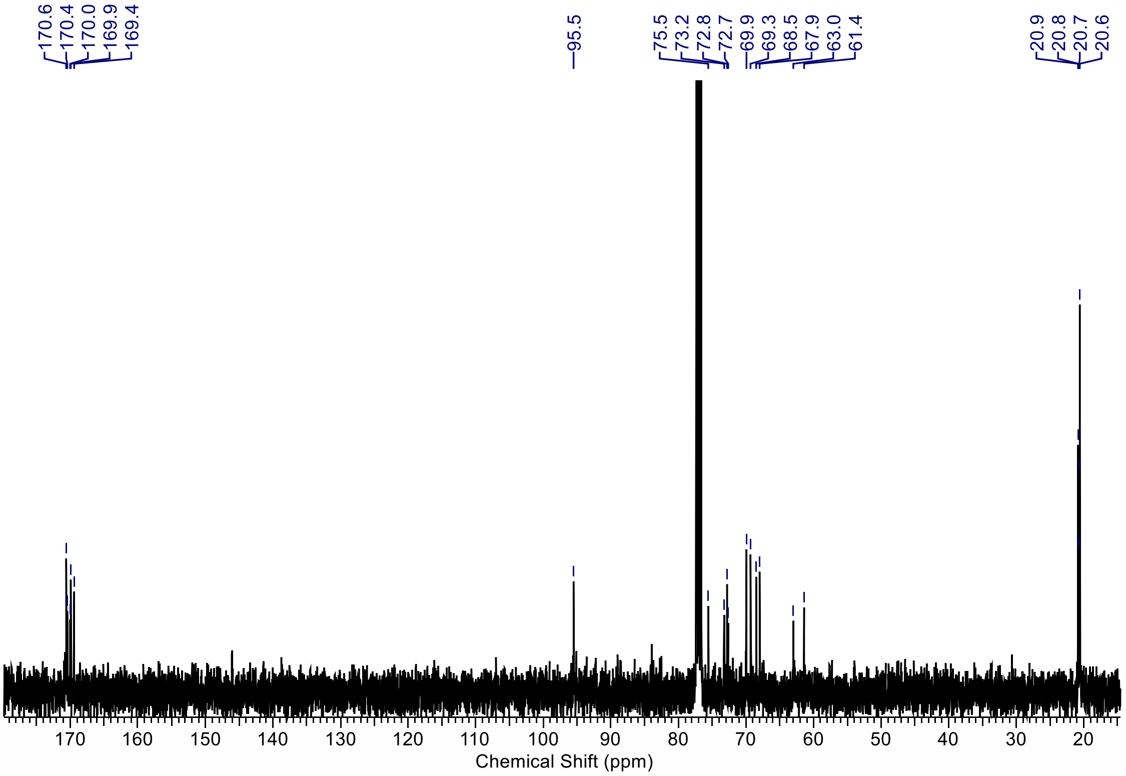
170.6 170.4 170.0 169.9 169.4
95.5
75.5 73.2 72.8 72.7 69.9 69.3 68.5 67.9 63.0 61.4
20.9 20.8 20.7 20.6
170 160 150 140 130 120 110 100 90 80 70 60 50 40 30 20
Chemical Shift (ppm)

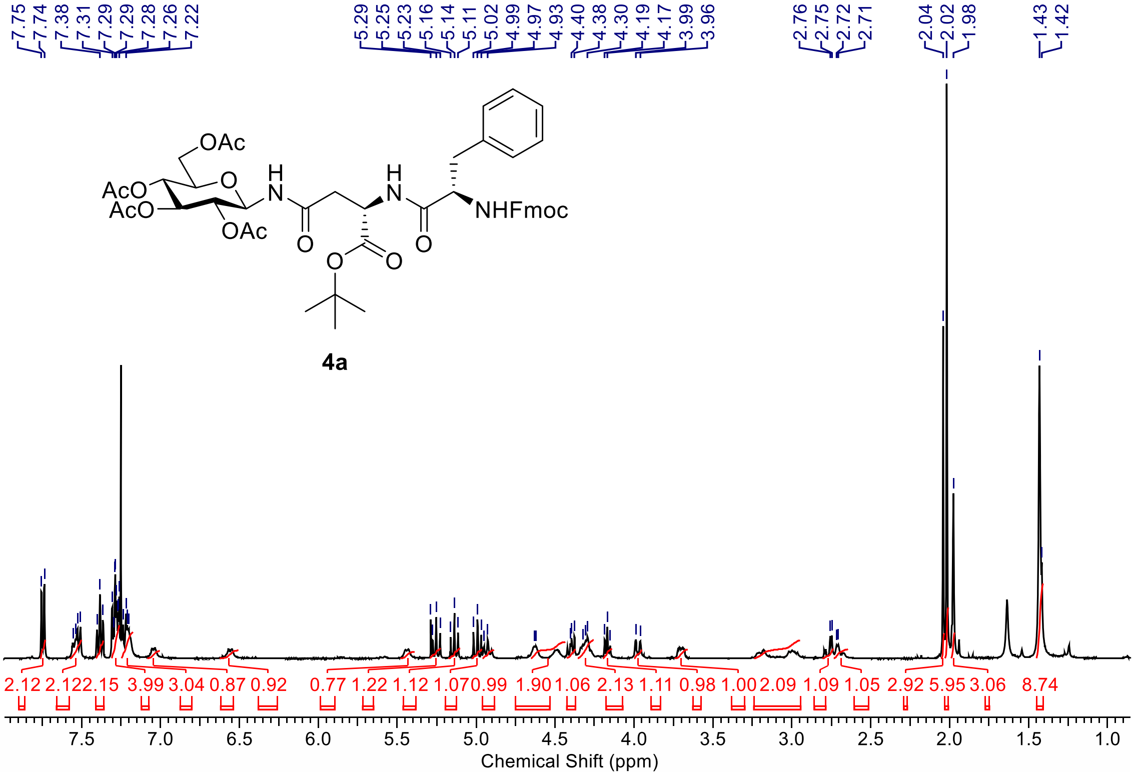
7.75 7.74 7.38 7.31 7.29 7.29 7.28 7.26 7.22
5.29 5.25 5.23 5.16 5.14 5.11 5.02 4.99 4.97 4.93 4.40 4.38 4.30 4.19 4.17 3.99 3.96
2.76 2.75 2.72 2.71
2.04 2.02 1.98
1.43 1.42
OAc
AcO
AcO
O
H
N
OAc
O
H
N
NHFmoc
O
O
O
4a
2.12 2.12 2.15 3.99 3.04 0.87 0.92 0.77 1.22 1.12 1.07 0.99 1.90 1.06 2.13 1.11 0.98 1.00 2.09 1.09 1.05 2.92 5.95 3.06 8.74
7.5 7.0 6.5 6.0 5.5 5.0 4.5 4.0 3.5 3.0 2.5 2.0 1.5 1.0
Chemical Shift (ppm)

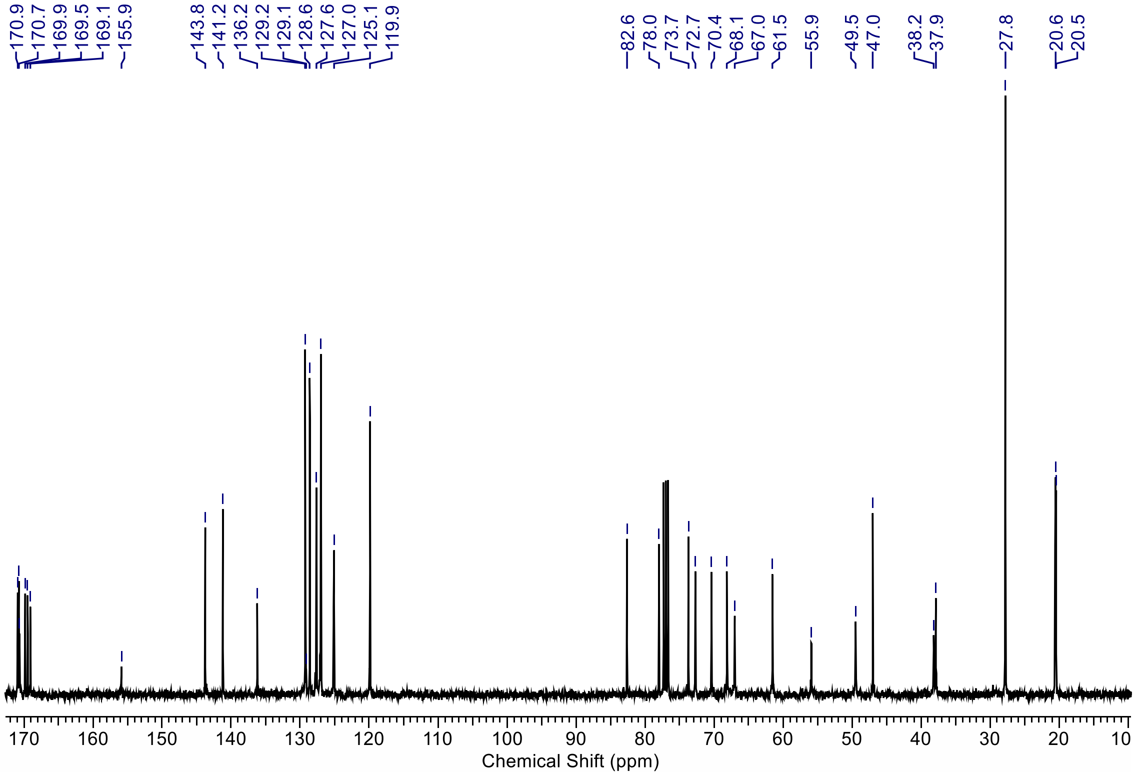
170.9 170.7 169.9 169.5 169.1 155.9
143.8 141.2 136.2 129.2 129.1 128.6 127.6 127.0 125.1 119.9
82.6 78.0 73.7 72.7 70.4 68.1 67.0 61.5 55.9 49.5 47.0 38.2 37.9 27.8 20.6 20.5
170 160 150 140 130 120 110 100 90 80 70 60 50 40 30 20 10
Chemical Shift (ppm)

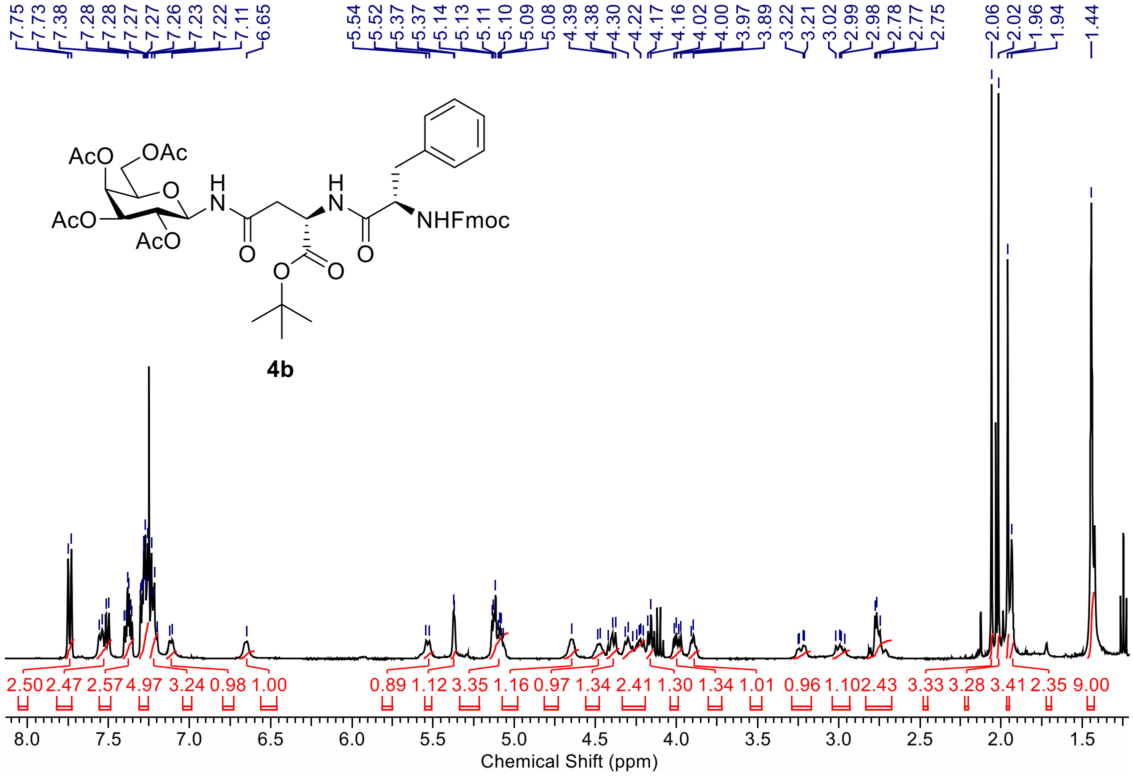
7.75 7.73 7.38 7.28 7.28 7.27 7.27 7.26 7.23 7.22 7.11 6.65
5.54 5.52 5.37 5.37 5.14 5.13 5.11 5.10 5.09 5.08 4.39 4.38 4.30 4.22 4.17 4.16 4.02 4.00 3.97 3.89 3.22 3.21 3.02 2.99 2.98 2.78 2.77 2.75
2.06 2.02 1.96 1.94 1.44
AcO
OAc
AcO
AcO
O
H
N
O
H
N
O
O
O
NHFmoc
4b
2.50 2.47 2.57 4.97 3.24 0.98 1.00 0.89 1.12 3.35 1.16 0.97 1.34 2.41 1.30 1.34 1.01 0.96 1.10 2.43 3.33 3.28 3.41 2.35 9.00
8.0 7.5 7.0 6.5 6.0 5.5 5.0 4.5 4.0 3.5 3.0 2.5 2.0 1.5
Chemical Shift (ppm)

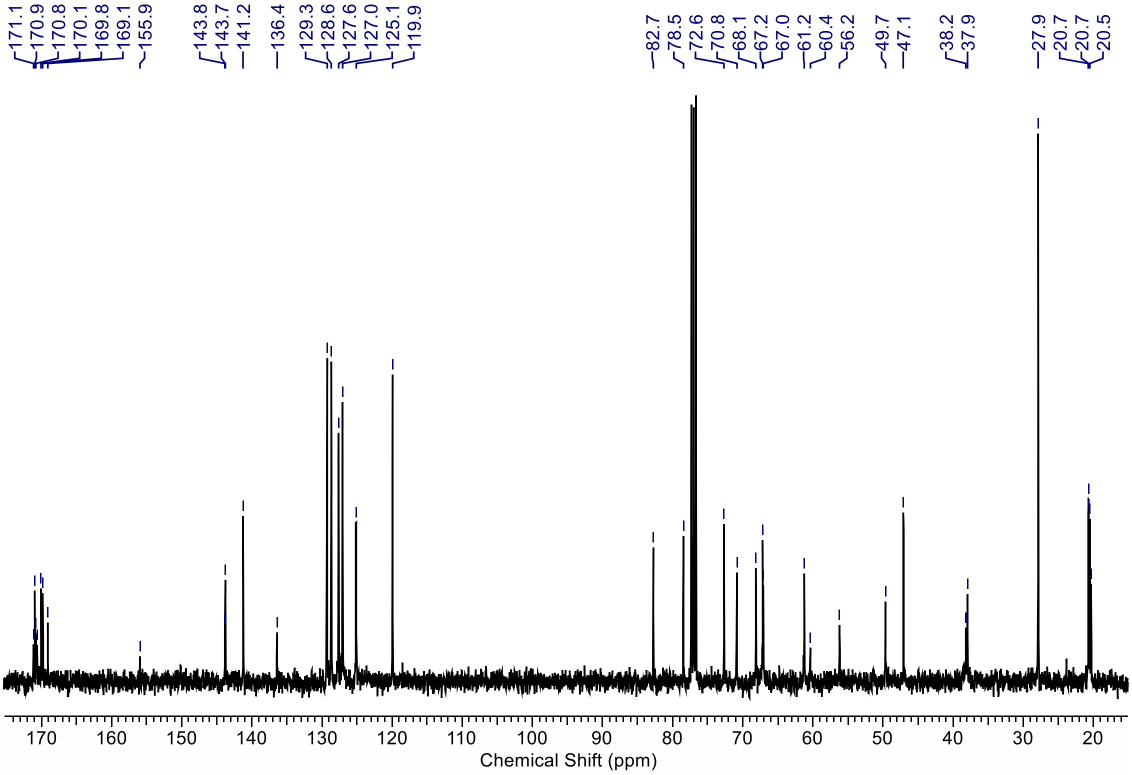
171.1 170.9 170.8 170.1 169.8 169.1 155.9 143.8 143.7 141.2 136.4 129.3 128.6 127.6 127.0 125.1 119.9
82.7 78.5 72.6 70.8 68.1 67.2 67.0 61.2 60.4 56.2 49.7 47.1 38.2 37.9 27.9 20.7 20.7 20.5
170 160 150 140 130 120 110 100 90 80 70 60 50 40 30 20
Chemical Shift (ppm)

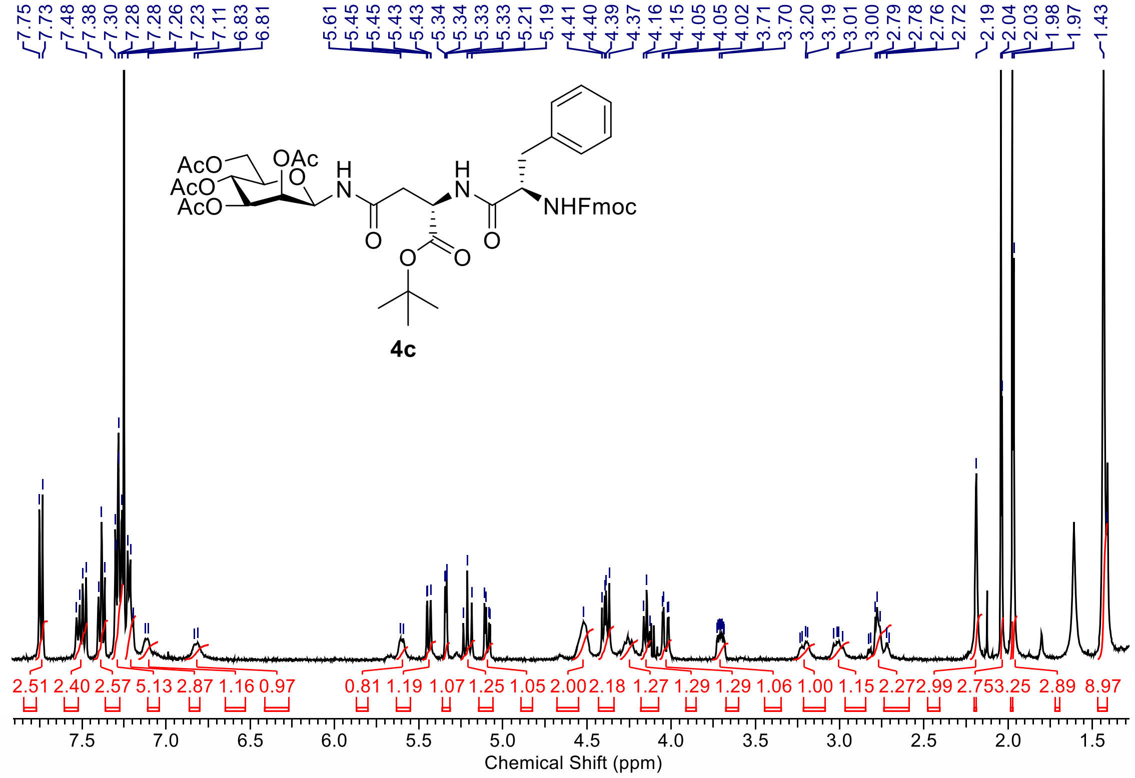
AcO
OAc
AcO
AcO
O
H
N
H
N
NHFmoc
O
O
O
O
4c
2.51 2.40 2.57 5.13 2.87 1.16 0.97 0.81 1.19 1.07 1.25 1.05 2.00 2.18 1.27 1.29 1.29 1.06 1.00 1.15 2.27 2.99 2.75 3.25 2.89 8.97
Chemical Shift (ppm)

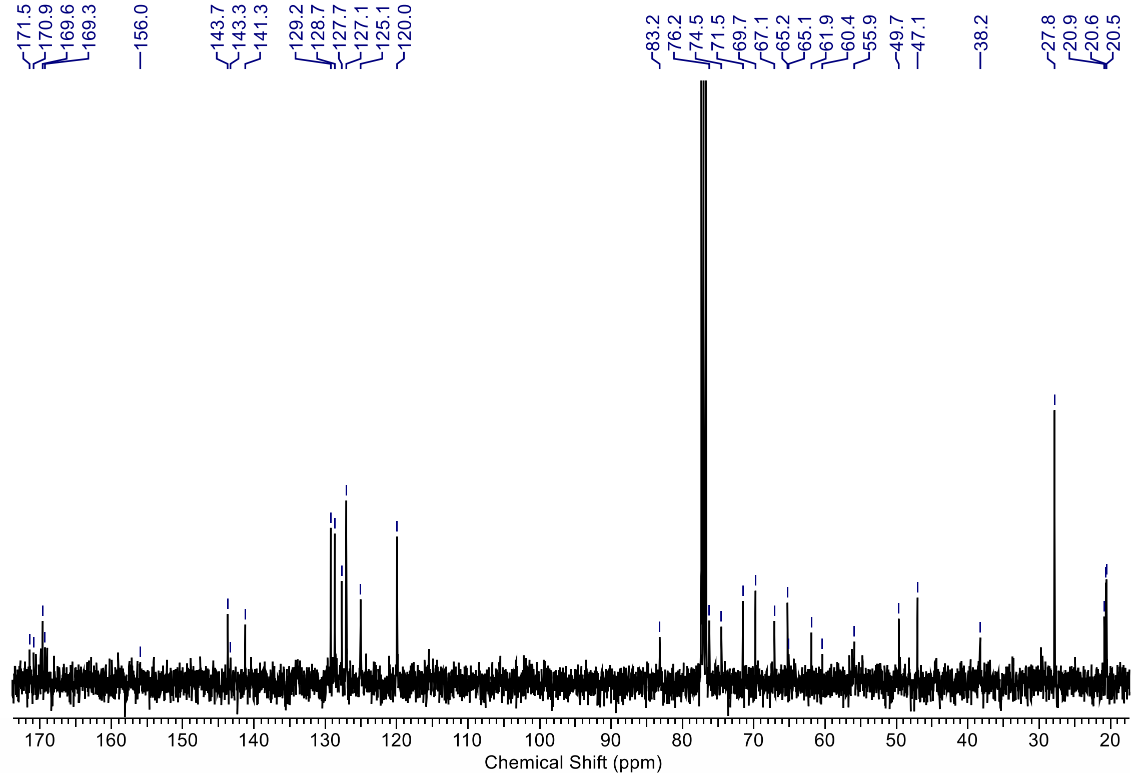
171.5
170.9
169.6
169.3
156.0
143.7
143.3
141.3
129.2
128.7
127.7
127.1
125.1
120.0
83.2
76.2
74.5
71.5
69.7
67.1
65.2
65.1
61.9
60.4
55.9
49.7
47.1
38.2
27.8
20.9
20.6
20.5
Chemical Shift (ppm)

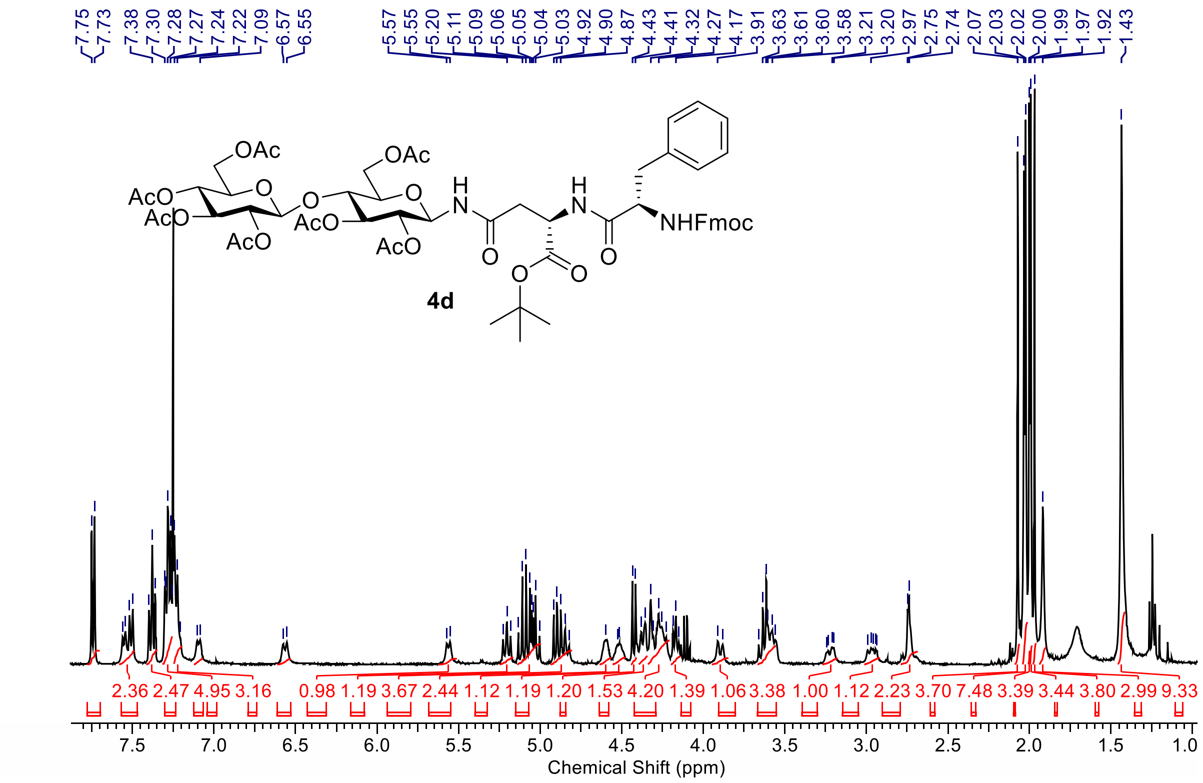
7.75
7.73
7.38
7.30
7.28
7.27
7.24
7.22
7.09
6.57
6.55
5.57
5.55
5.20
5.11
5.09
5.06
5.05
5.04
5.03
4.92
4.90
4.87
4.43
4.41
4.32
4.27
4.17
3.91
3.63
3.61
3.60
3.58
3.21
3.20
2.97
2.75
2.74
2.07
2.03
2.02
2.00
1.99
1.97
1.92
1.43
OAc
AcO
AcO
O
O
AcO
AcO
AcO
OAc
O
H
N
O
H
N
O
NHFmoc
O
O
4d
2.36
2.47
4.95
3.16
0.98
1.19
3.67
2.44
1.12
1.19
1.20
1.53
4.20
1.39
1.06
3.38
1.00
1.12
2.23
3.70
7.48
3.39
3.44
3.80
2.99
9.33
Chemical Shift (ppm)

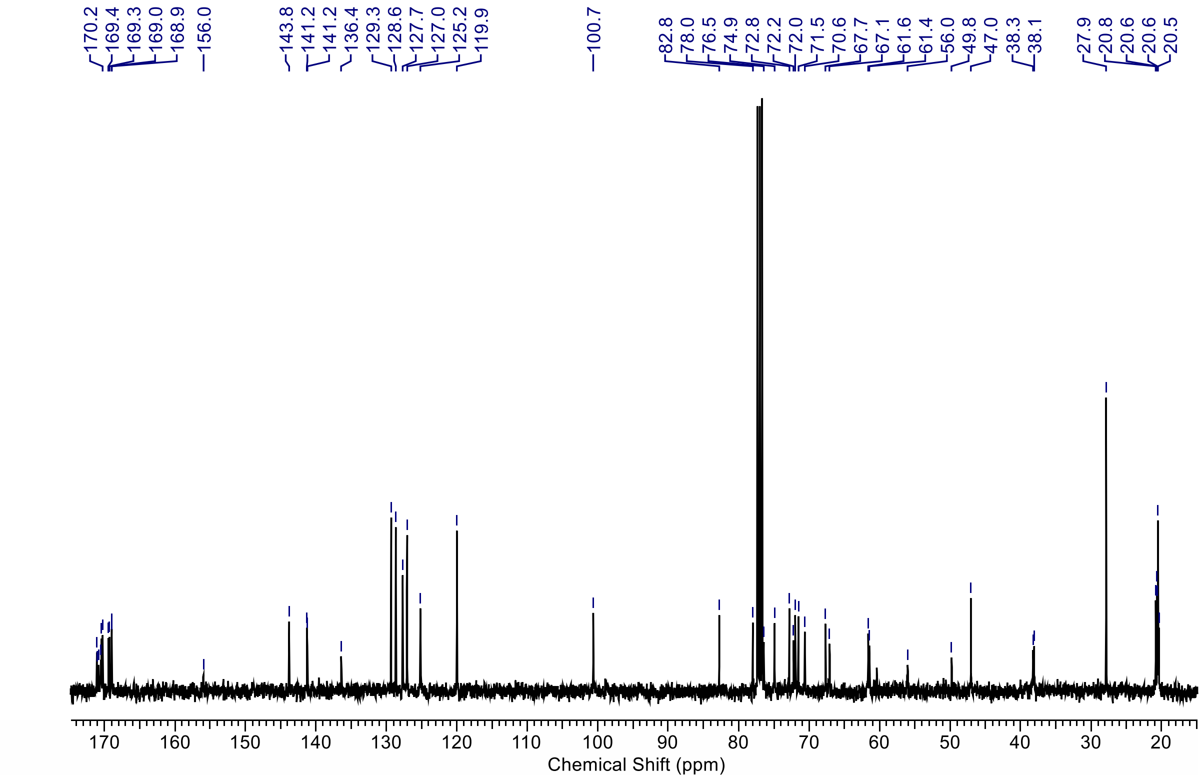
170.2
169.4
169.3
169.0
168.9
156.0
143.8
141.2
141.2
136.4
129.3
128.6
127.7
127.0
125.2
119.9
100.7
82.8
78.0
76.5
74.9
72.8
72.2
72.0
71.5
70.6
67.7
67.1
61.6
61.4
56.0
49.8
47.0
38.3
38.1
27.9
20.8
20.6
20.6
20.5
Chemical Shift (ppm)

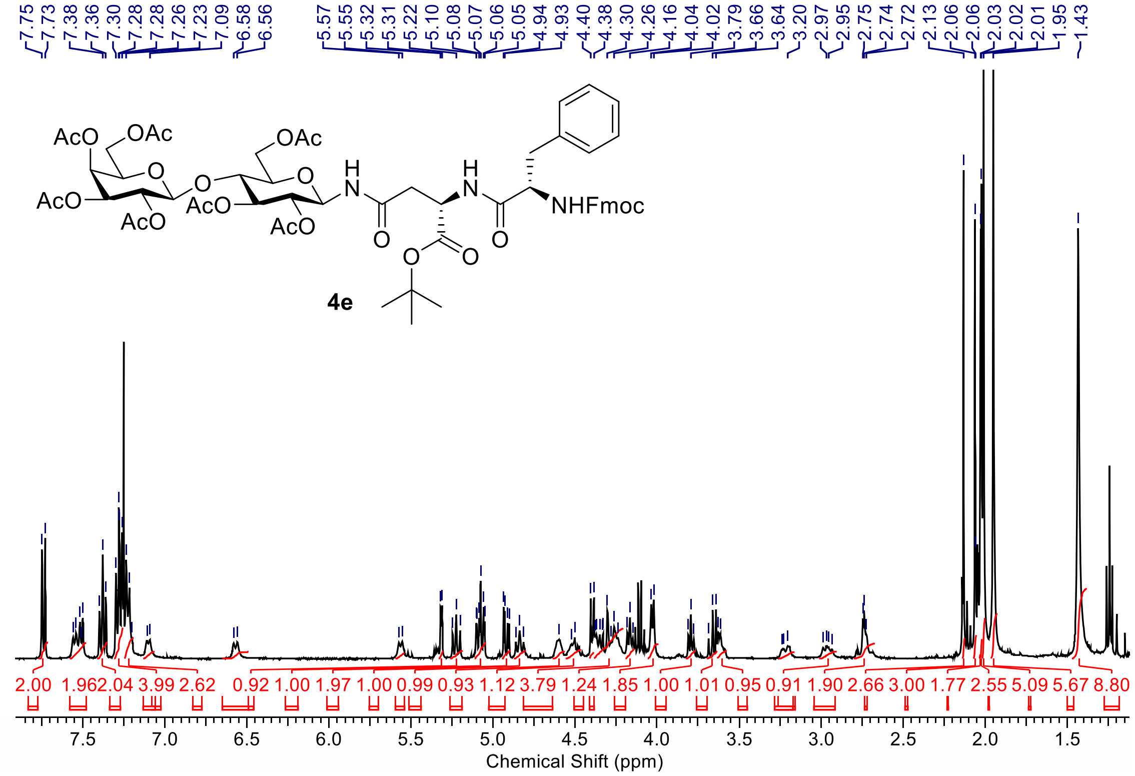

7.75 7.73 7.38 7.36 7.30 7.28 7.28 7.26 7.23 7.09 6.58 6.56 5.57 5.55 5.32 5.31 5.22 5.10 5.08 5.07 5.06 5.05 4.94 4.93 4.40 4.38 4.30 4.26 4.16 4.04 4.02 3.79 3.66 3.64 3.20 2.97 2.95 2.75 2.74 2.72 2.13 2.06 2.06 2.03 2.02 2.01 1.95 1.43
AcO
OAc
AcO
AcO
O
O
AcO
OAc
O
AcO
H
N
O
H
N
NHFmoc
O
O
O
4e
2.00 1.96 2.04 3.99 2.62 0.92 1.00 1.97 1.00 0.99 0.93 1.12 3.79 1.24 1.85 1.00 1.01 0.95 0.91 1.90 2.66 3.00 1.77 2.55 5.09 5.67 8.80
7.5 7.0 6.5 6.0 5.5 5.0 4.5 4.0 3.5 3.0 2.5 2.0 1.5
Chemical Shift (ppm)

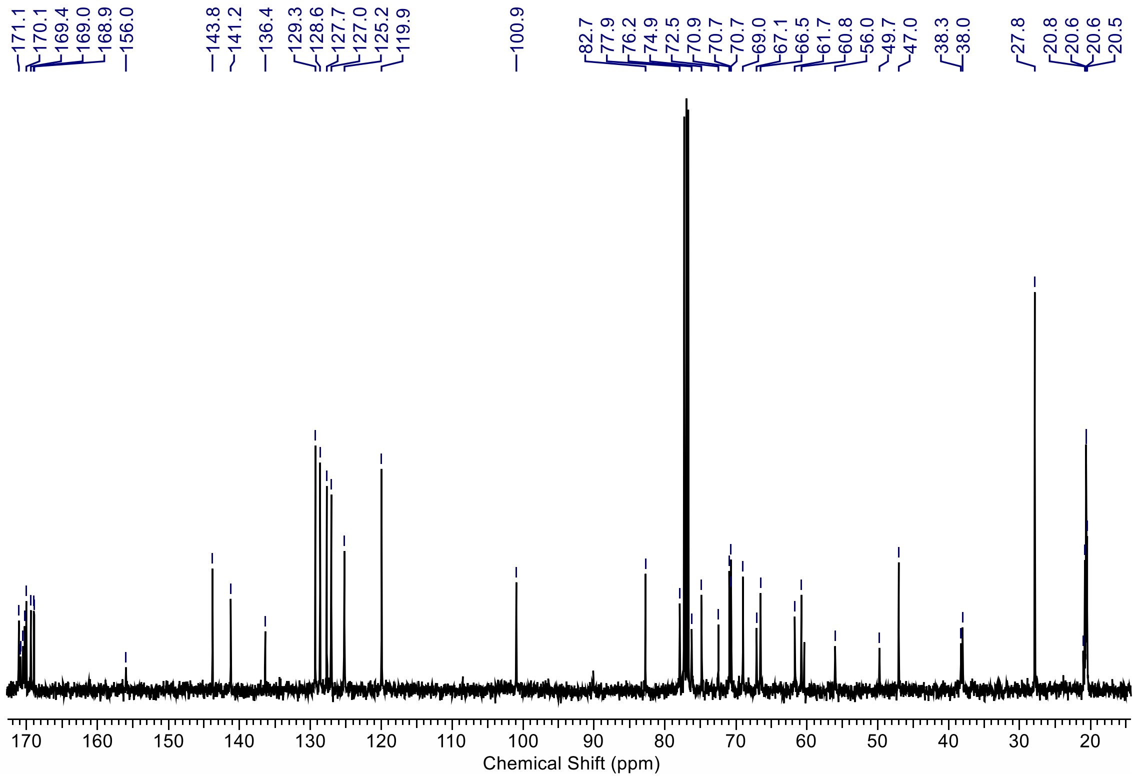

171.1 170.1 169.4 169.0 168.9 156.0 143.8 141.2 136.4 129.3 128.6 127.7 127.0 125.2 119.9 100.9 82.7 77.9 76.2 74.9 72.5 70.9 70.7 70.7 69.0 67.1 66.5 61.7 60.8 56.0 49.7 47.0 38.3 38.0 27.8 20.8 20.6 20.6 20.5
170 160 150 140 130 120 110 100 90 80 70 60 50 40 30 20
Chemical Shift (ppm)

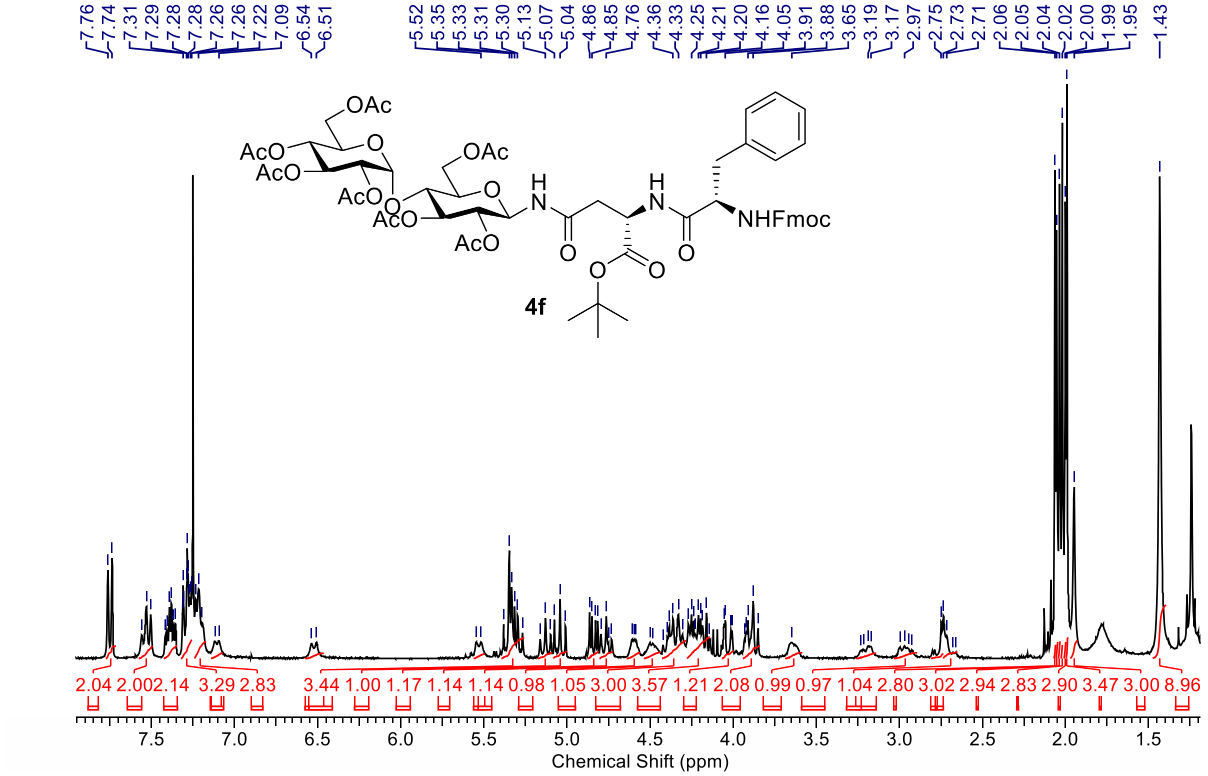
4f
Chemical Shift (ppm)

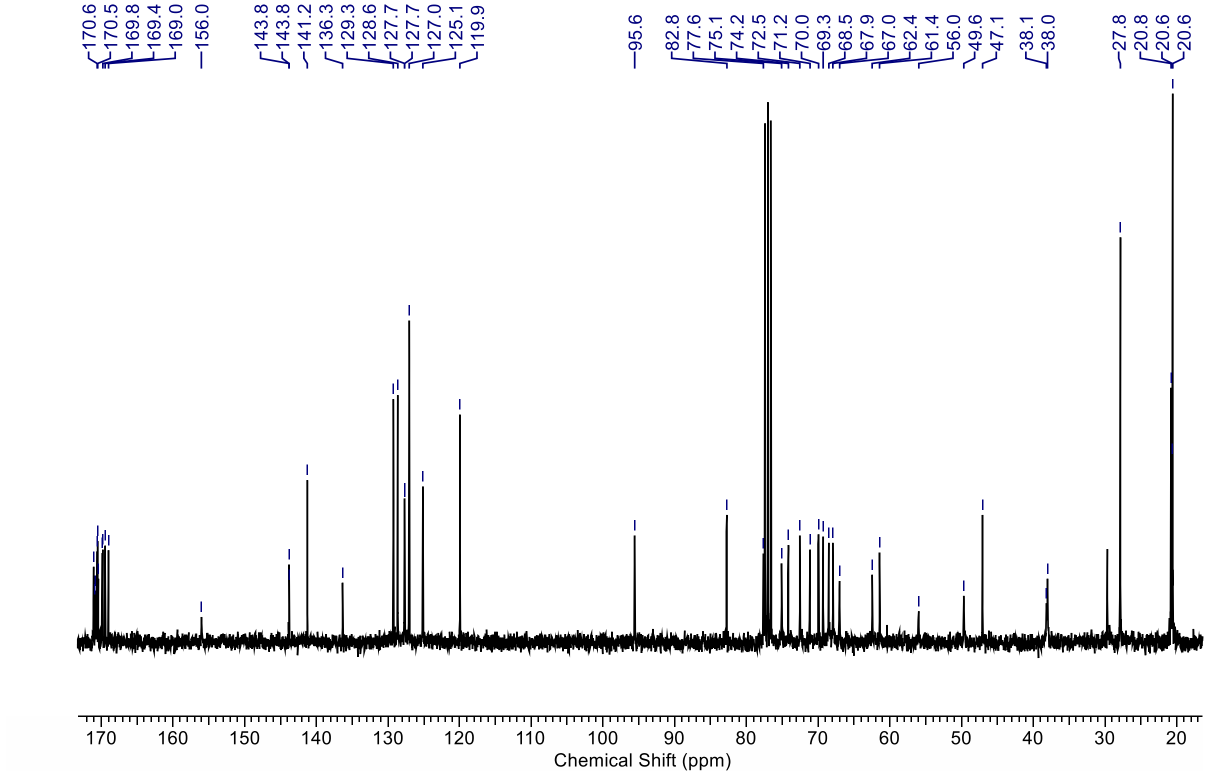
Chemical Shift (ppm)

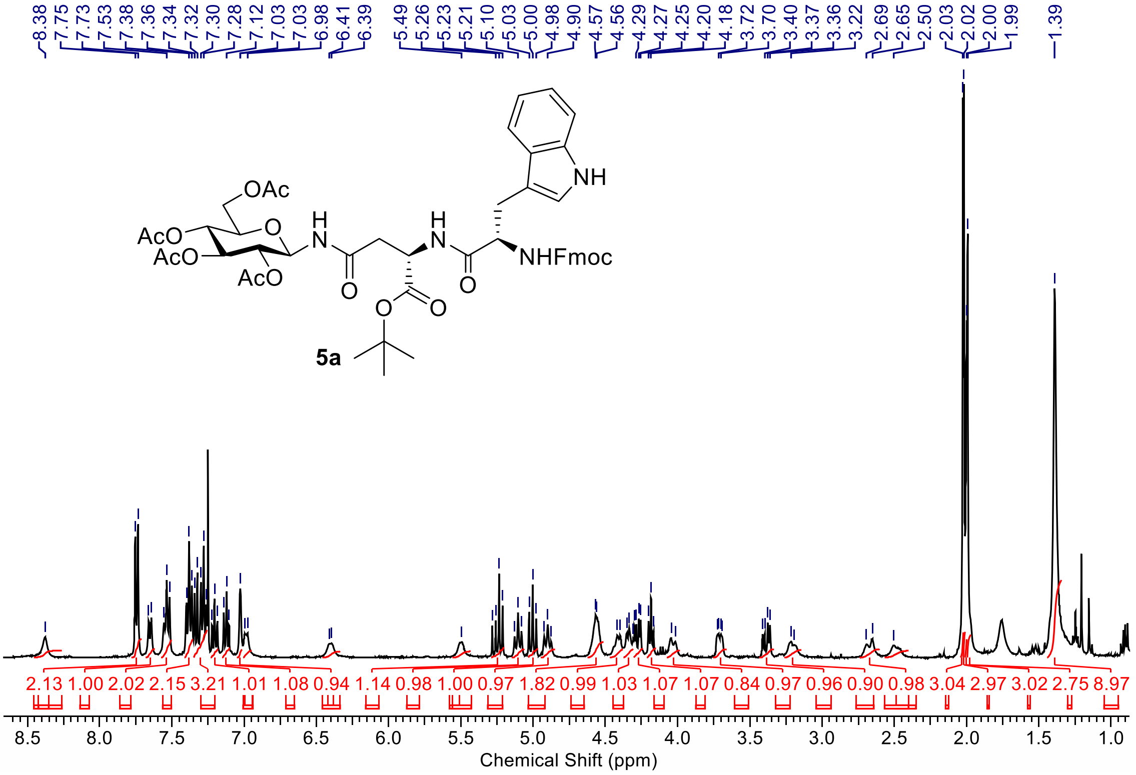
8.38 7.75 7.73 7.53 7.38 7.36 7.34 7.32 7.30 7.28 7.12 7.03 7.03 6.98 6.41 6.39 5.49 5.26 5.23 5.21 5.10 5.03 5.00 4.98 4.90 4.57 4.56 4.29 4.27 4.25 4.20 4.18 3.72 3.70 3.40 3.37 3.36 3.22 2.69 2.65 2.50 2.03 2.02 2.00 1.99 1.39
OAc
AcO
AcO
AcO
O
H
N
H
N
O
O
O
O
NH
NHFmoc
5a
2.13 1.00 2.02 2.15 3.21 1.01 1.08 0.94 1.14 0.98 1.00 0.97 1.82 0.99 1.03 1.07 1.07 0.84 0.97 0.96 0.90 0.98 3.04 2.97 3.02 2.75 8.97
8.5 8.0 7.5 7.0 6.5 6.0 5.5 5.0 4.5 4.0 3.5 3.0 2.5 2.0 1.5 1.0
Chemical Shift (ppm)

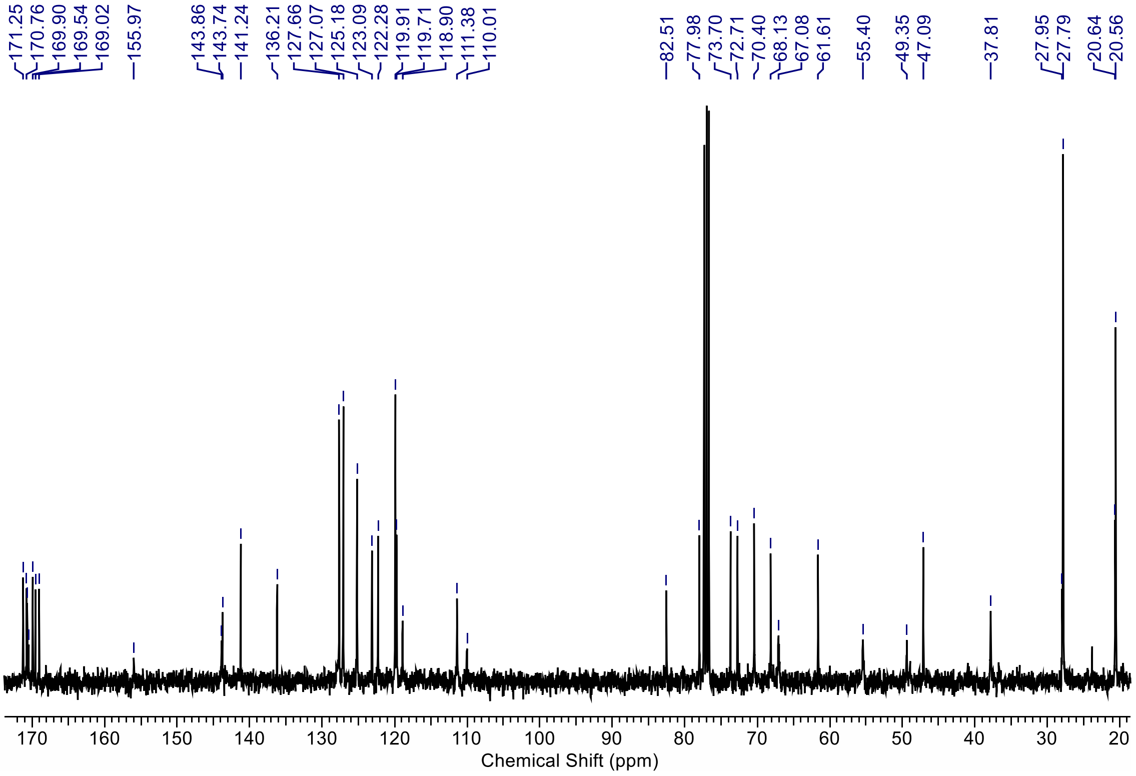
171.25 170.76 169.90 169.54 169.02 155.97 143.86 143.74 141.24 136.21 127.66 127.07 125.18 123.09 122.28 119.91 119.71 118.90 111.38 110.01 82.51 77.98 73.70 72.71 70.40 68.13 67.08 61.61 55.40 49.35 47.09 37.81 27.95 27.79 20.64 20.56
170 160 150 140 130 120 110 100 90 80 70 60 50 40 30 20
Chemical Shift (ppm)

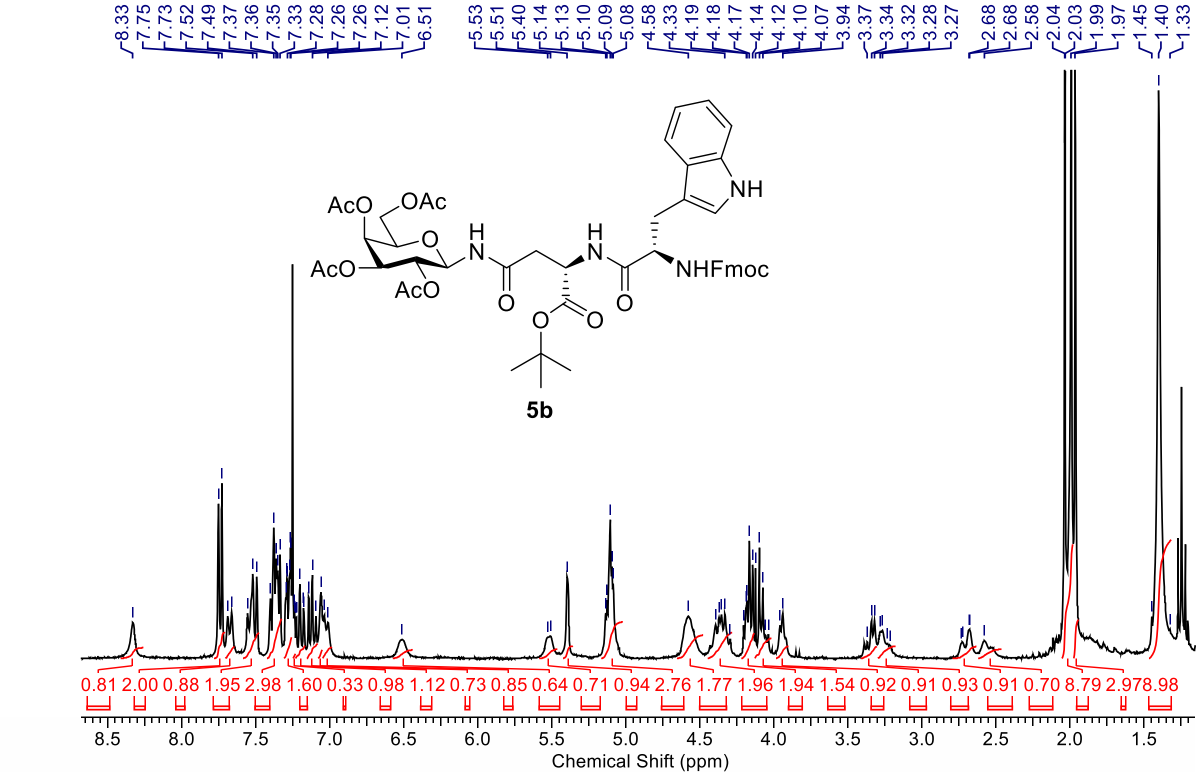
8.33 7.75 7.73 7.52 7.49 7.37 7.36 7.35 7.33 7.28 7.26 7.26 7.12 7.01 6.51
5.53 5.51 5.40 5.14 5.13 5.10 5.09 5.08 4.58 4.33 4.19 4.18 4.17 4.14 4.12 4.10 4.07 3.94
3.37 3.34 3.32 3.28 3.27 2.68 2.68 2.58 2.04 2.03 1.99 1.97 1.45 1.40 1.33
AcO
OAc
O
AcO
AcO
H
N
O
H
N
O
O
O
NH
NHFmoc
5b
0.81 2.00 0.88 1.95 2.98 1.60 0.33 0.98 1.12 0.73 0.85 0.64 0.71 0.94 2.76 1.77 1.96 1.94 1.54 0.92 0.91 0.93 0.91 0.70 8.79 2.97 8.98
8.5 8.0 7.5 7.0 6.5 6.0 5.5 5.0 4.5 4.0 3.5 3.0 2.5 2.0 1.5
Chemical Shift (ppm)

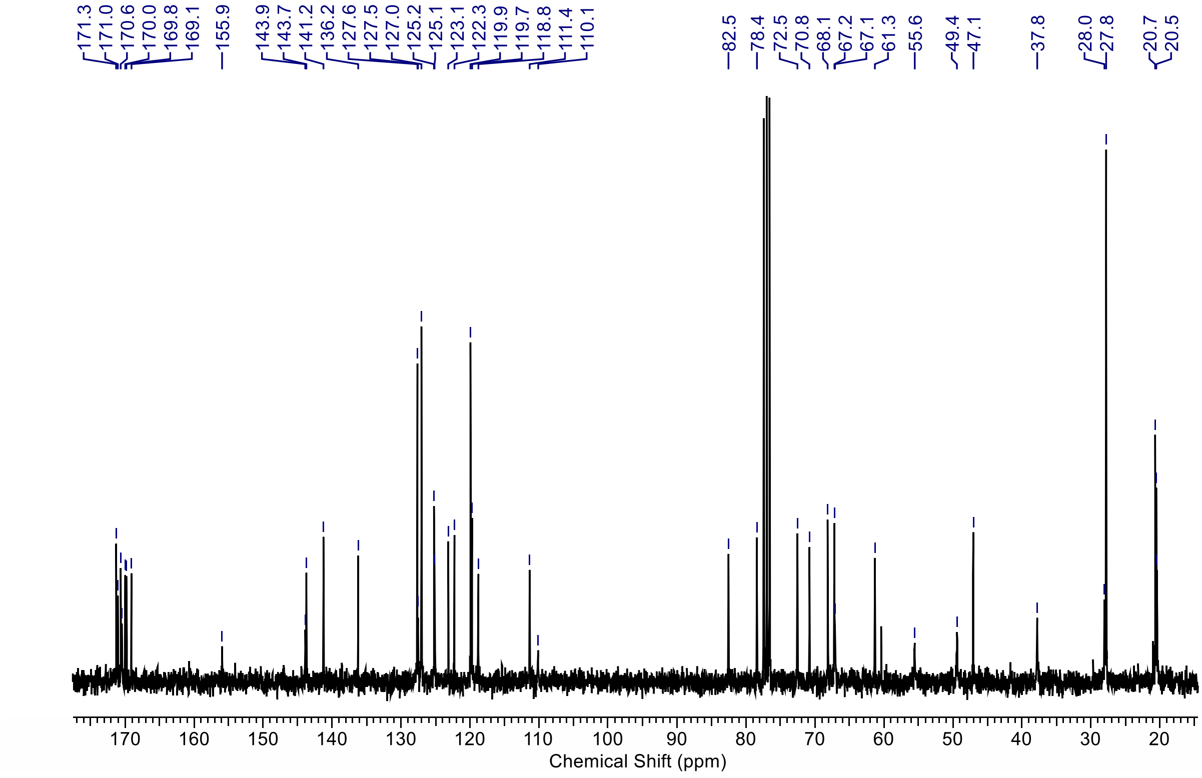
171.3 171.0 170.6 170.0 169.8 169.1 155.9 143.9 143.7 141.2 136.2 127.6 127.5 127.0 125.2 125.1 123.1 122.3 119.9 119.7 118.8 111.4 110.1
82.5 78.4 72.5 70.8 68.1 67.2 67.1 61.3 55.6 49.4 47.1 37.8 28.0 27.8 20.7 20.5
170 160 150 140 130 120 110 100 90 80 70 60 50 40 30 20
Chemical Shift (ppm)

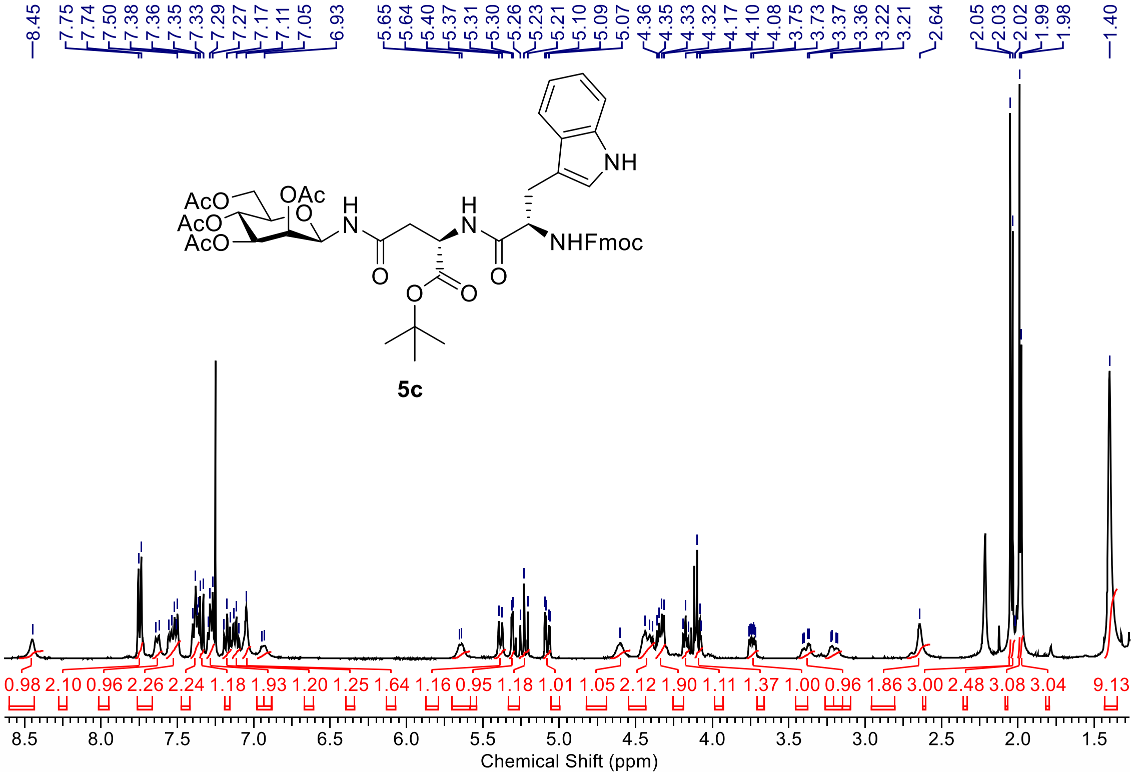

8.45 7.75 7.74 7.50 7.38 7.36 7.35 7.33 7.29 7.27 7.17 7.11 7.05 6.93 5.65 5.64 5.40 5.37 5.31 5.30 5.26 5.23 5.21 5.10 5.09 5.07 4.36 4.35 4.33 4.32 4.17 4.10 4.08 3.75 3.73 3.37 3.36 3.22 3.21 2.64 2.05 2.03 2.02 1.99 1.98 1.40
AcO
AcO
AcO
OAc
O
H
N
H
N
NH
NHFmoc
O
O
O
O
5c
0.98 2.10 0.96 2.26 2.24 1.18 1.93 1.20 1.25 1.64 1.16 0.95 1.18 1.01 1.05 2.12 1.90 1.11 1.37 1.00 0.96 1.86 3.00 2.48 3.08 3.04 9.13
8.5 8.0 7.5 7.0 6.5 6.0 5.5 5.0 4.5 4.0 3.5 3.0 2.5 2.0 1.5
Chemical Shift (ppm)

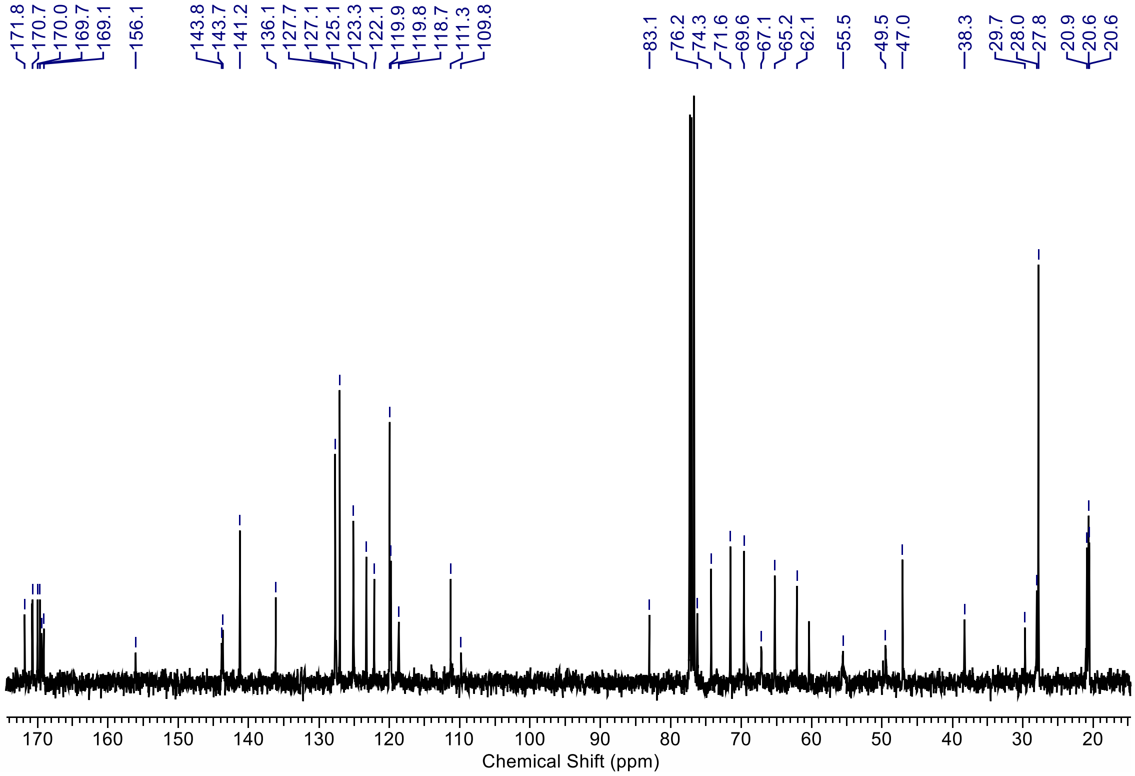

171.8 170.7 170.0 169.7 169.1 156.1 143.8 143.7 141.2 136.1 127.7 127.1 125.1 123.3 122.1 119.9 119.8 118.7 111.3 109.8 83.1 76.2 74.3 71.6 69.6 67.1 65.2 62.1 55.5 49.5 47.0 38.3 29.7 28.0 27.8 20.9 20.6 20.6
170 160 150 140 130 120 110 100 90 80 70 60 50 40 30 20
Chemical Shift (ppm)

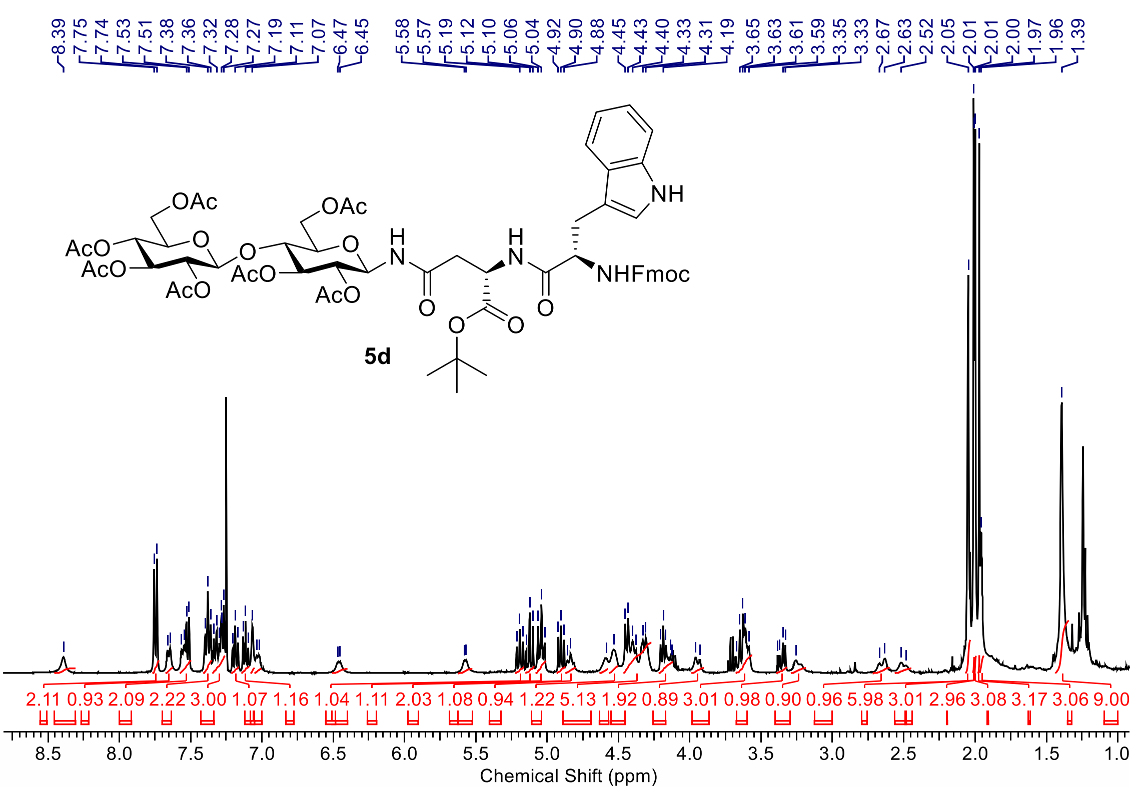
8.39
7.75
7.74
7.53
7.51
7.38
7.36
7.32
7.28
7.27
7.19
7.11
7.07
6.47
6.45
5.58
5.57
5.19
5.12
5.10
5.06
5.04
4.92
4.90
4.88
4.45
4.43
4.40
4.33
4.31
4.19
3.65
3.63
3.61
3.59
3.35
3.33
2.67
2.63
2.52
2.05
2.01
2.01
2.00
1.97
1.96
1.39
OAc
OAc
AcO
AcO
AcO
AcO
AcO
O
NH
NHFmoc
5d
2.11 0.93 2.09 2.22 3.00 1.07 1.16 1.04 1.11 2.03 1.08 0.94 1.22 5.13 1.92 0.89 3.01 0.98 0.90 0.96 5.98 3.01 2.96 3.08 3.17 3.06 9.00
8.5 8.0 7.5 7.0 6.5 6.0 5.5 5.0 4.5 4.0 3.5 3.0 2.5 2.0 1.5 1.0
Chemical Shift (ppm)

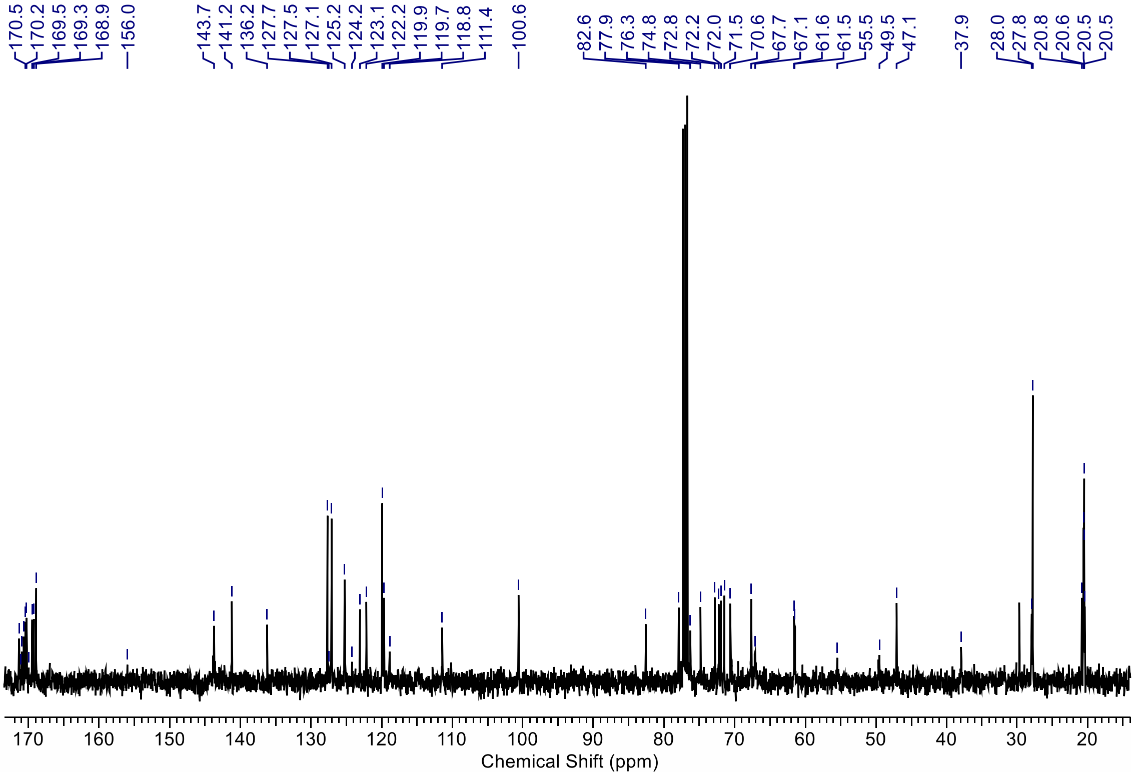
170.5
170.2
169.5
169.3
168.9
156.0
143.7
141.2
136.2
127.7
127.5
127.1
125.2
124.2
123.1
122.2
119.9
119.7
118.8
111.4
100.6
82.6
77.9
76.3
74.8
72.8
72.2
72.0
71.5
70.6
67.7
67.1
61.6
61.5
55.5
49.5
47.1
37.9
28.0
27.8
20.8
20.6
20.5
20.5
170 160 150 140 130 120 110 100 90 80 70 60 50 40 30 20
Chemical Shift (ppm)

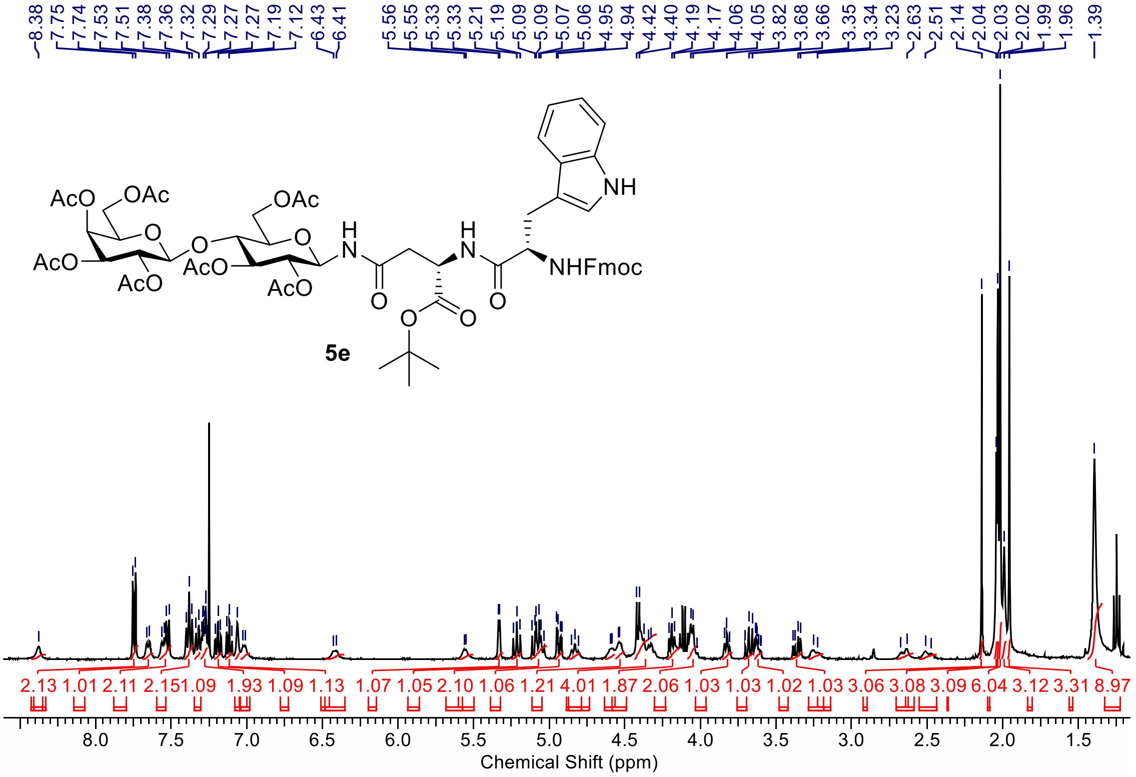
8.38 7.75 7.74 7.53 7.51 7.38 7.36 7.32 7.29 7.27 7.27 7.19 7.12 6.43 6.41 5.56 5.55 5.33 5.33 5.21 5.19 5.09 5.09 5.07 5.06 4.95 4.94 4.42 4.40 4.19 4.17 4.06 4.05 3.82 3.68 3.66 3.35 3.34 3.23 2.63 2.51 2.14 2.04 2.03 2.02 1.99 1.96 1.39
AcO
OAc
OAc
O
AcO
AcO
AcO
AcO
H
N
H
N
NH
NHFmoc
O
O
O
O
5e
2.13 1.01 2.11 2.15 1.09 1.93 1.09 1.13 1.07 1.05 2.10 1.06 1.21 4.01 1.87 2.06 1.03 1.03 1.02 1.03 3.06 3.08 3.09 6.04 3.12 3.31 8.97
8.0 7.5 7.0 6.5 6.0 5.5 5.0 4.5 4.0 3.5 3.0 2.5 2.0 1.5
Chemical Shift (ppm)

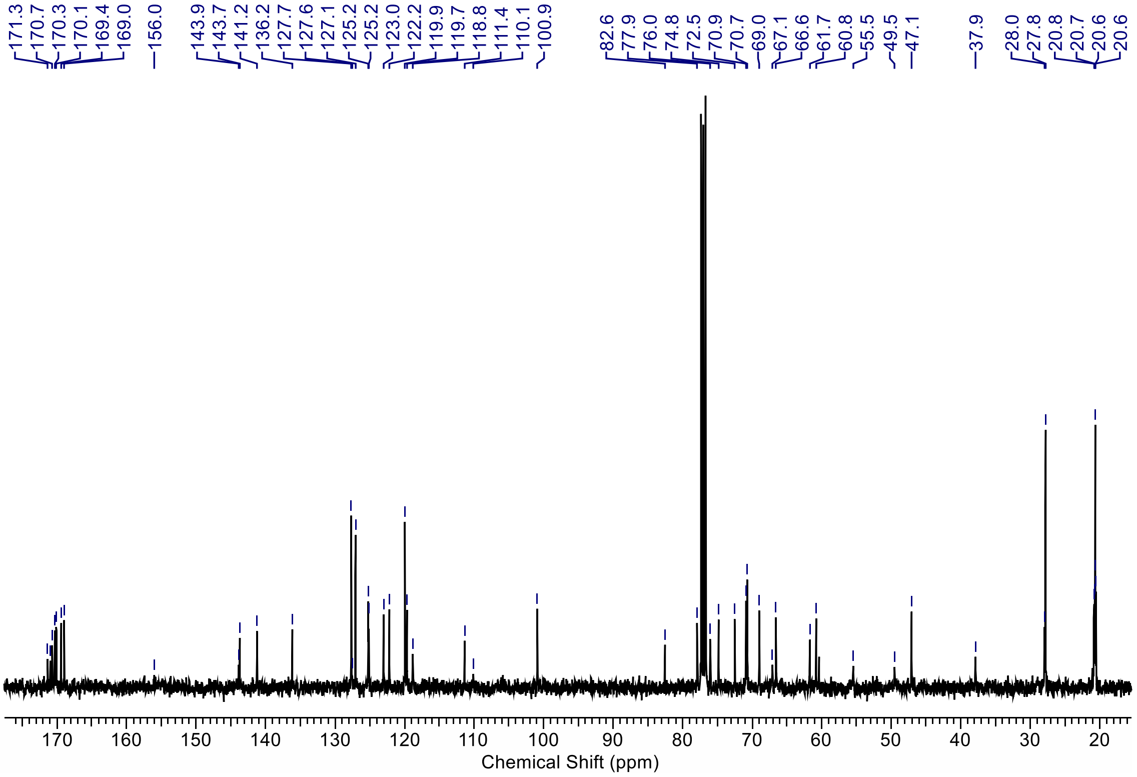
171.3 170.7 170.3 170.1 169.4 169.0 156.0 143.9 143.7 141.2 136.2 127.7 127.6 127.1 125.2 125.2 123.0 122.2 119.9 119.7 118.8 111.4 110.1 100.9 82.6 77.9 76.0 74.8 72.5 70.9 70.7 69.0 67.1 66.6 61.7 60.8 55.5 49.5 47.1 37.9 28.0 27.8 20.8 20.7 20.6 20.6
170 160 150 140 130 120 110 100 90 80 70 60 50 40 30 20
Chemical Shift (ppm)

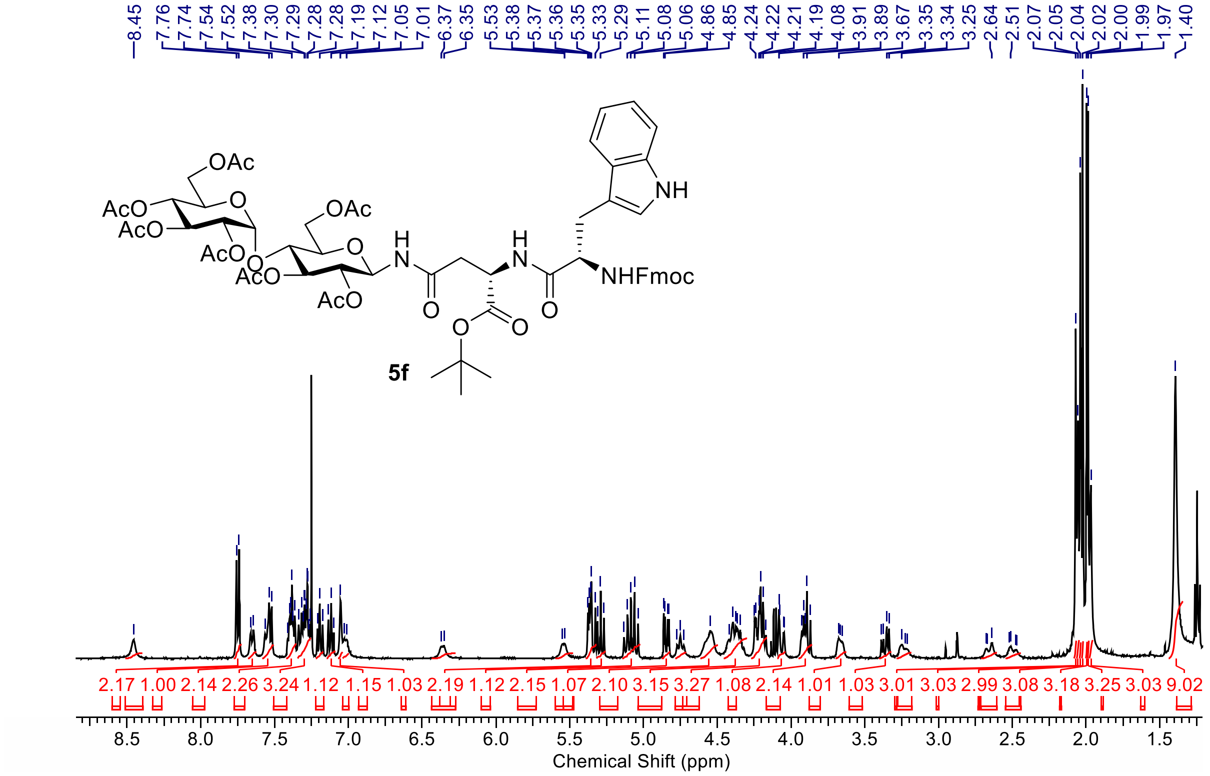
5f
NHFmoc
Chemical Shift (ppm)

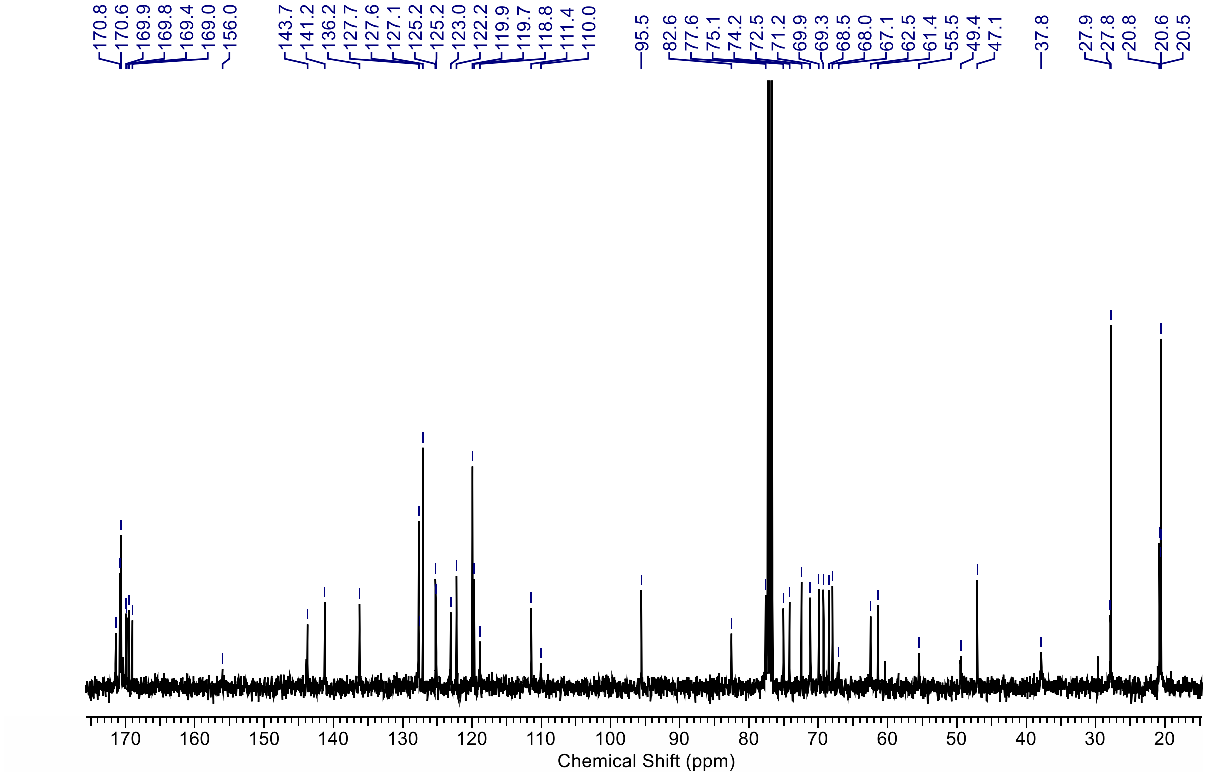
Chemical Shift (ppm)

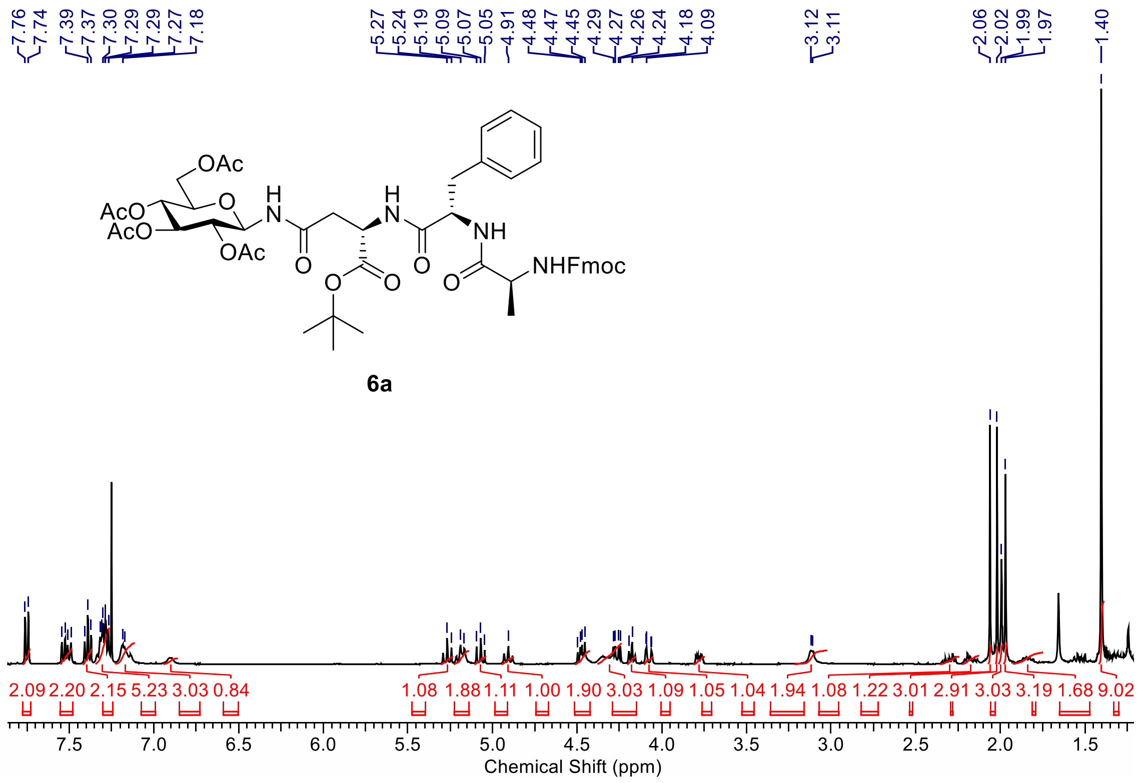
7.76
7.74
7.39
7.37
7.30
7.29
7.29
7.27
7.18
5.27
5.24
5.19
5.09
5.07
5.05
4.91
4.48
4.47
4.45
4.29
4.27
4.26
4.24
4.18
4.09
3.12
3.11
2.06
2.02
1.99
1.97
1.40
OAc
AcO
AcO
O
H
N
OAc
O
H
N
O
NH
O
O
O
NHFmoc
6a
2.09 2.20 2.15 5.23 3.03 0.84
1.08 1.88 1.11 1.00 1.90 3.03 1.09 1.05 1.04 1.94 1.08 1.22 3.01 2.91 3.03 3.19 1.68 9.02
7.5
7.0
6.5
6.0
5.5
5.0
4.5
4.0
3.5
3.0
2.5
2.0
1.5
Chemical Shift (ppm)

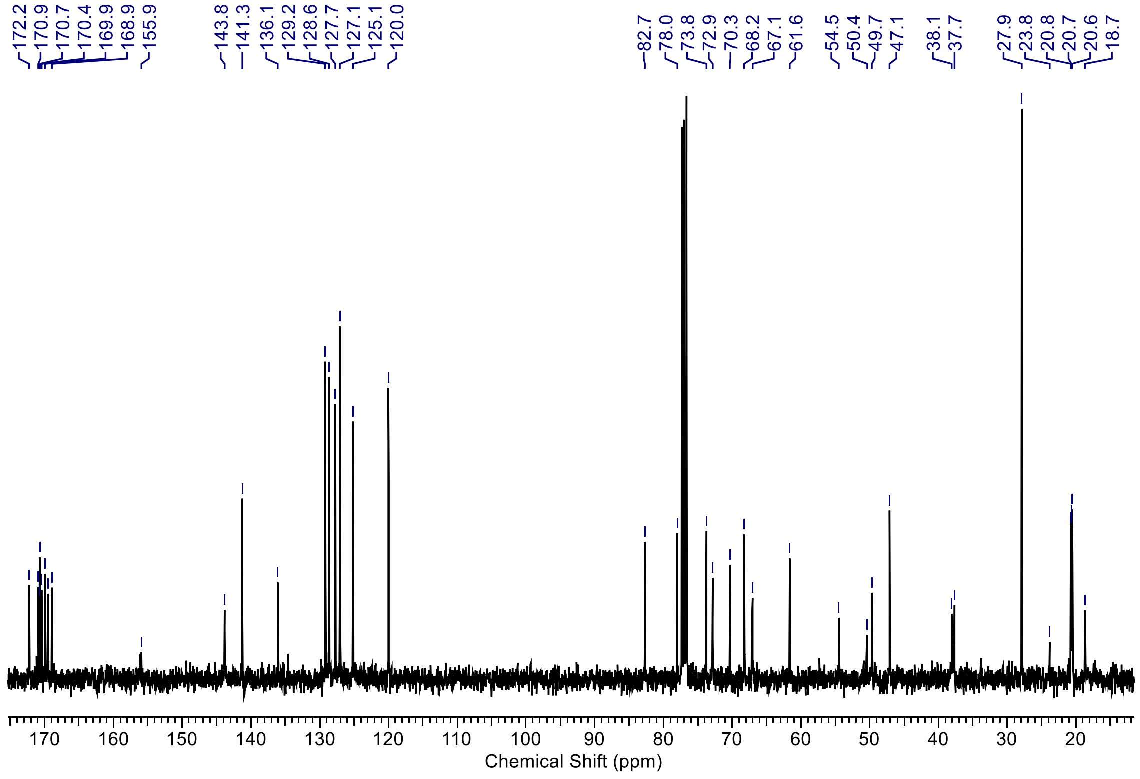
172.2
170.9
170.7
170.4
169.9
168.9
155.9
143.8
141.3
136.1
129.2
128.6
127.7
127.1
125.1
120.0
82.7
78.0
73.8
72.9
70.3
68.2
67.1
61.6
54.5
50.4
49.7
47.1
38.1
37.7
27.9
23.8
20.8
20.7
20.6
18.7
170
160
150
140
130
120
110
100
90
80
70
60
50
40
30
20
Chemical Shift (ppm)

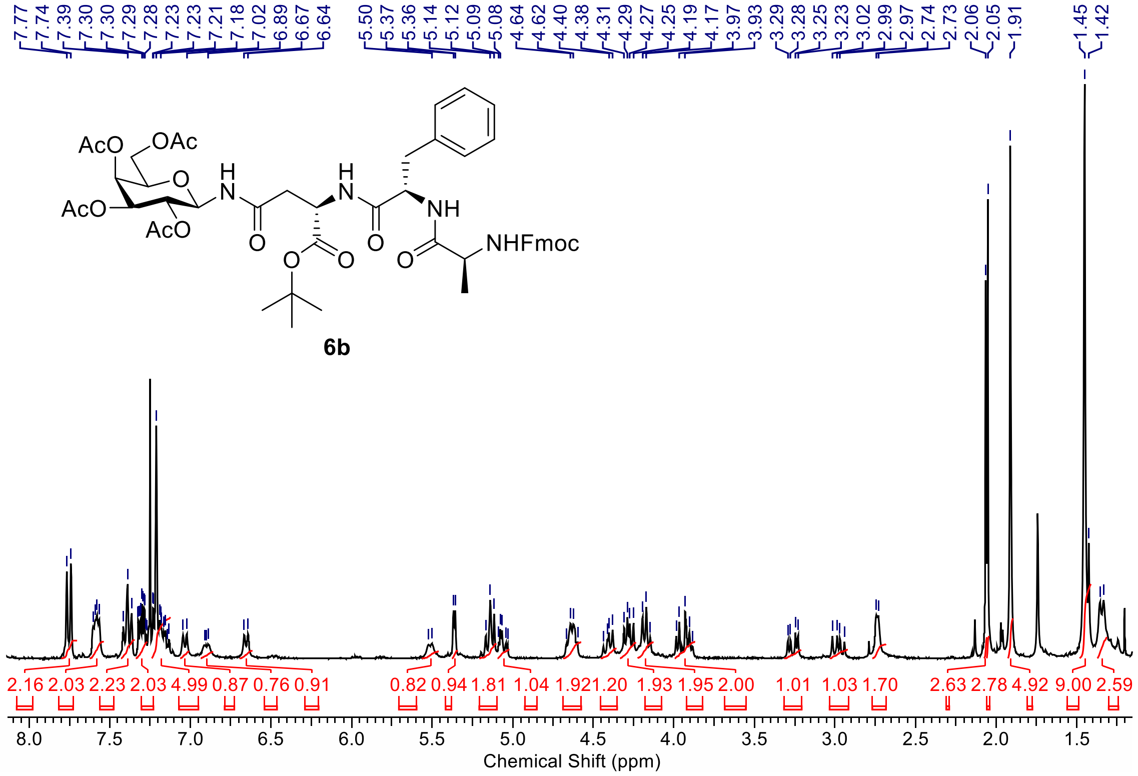
7.77 7.74 7.39 7.30 7.30 7.29 7.28 7.23 7.23 7.21 7.18 7.02 6.89 6.67 6.64
5.50 5.37 5.36 5.14 5.12 5.09 5.08 4.64 4.62 4.40 4.38 4.31 4.29 4.27 4.25 4.19 4.17 3.97 3.93 3.29 3.28 3.25 3.23 3.02 2.99 2.97 2.74 2.73 2.06 2.05 1.91
1.45 1.42
AcO
OAc
O
H
N
AcO
AcO
O
H
N
O
O
O
NH
NHFmoc
O
O
6b
2.16 2.03 2.23 2.03 4.99 0.87 0.76 0.91
0.82 0.94 1.81 1.04 1.92 1.20 1.93 1.95 2.00
1.01 1.03 1.70
2.63 2.78 4.92 9.00 2.59
8.0 7.5 7.0 6.5 6.0 5.5 5.0 4.5 4.0 3.5 3.0 2.5 2.0 1.5
Chemical Shift (ppm)

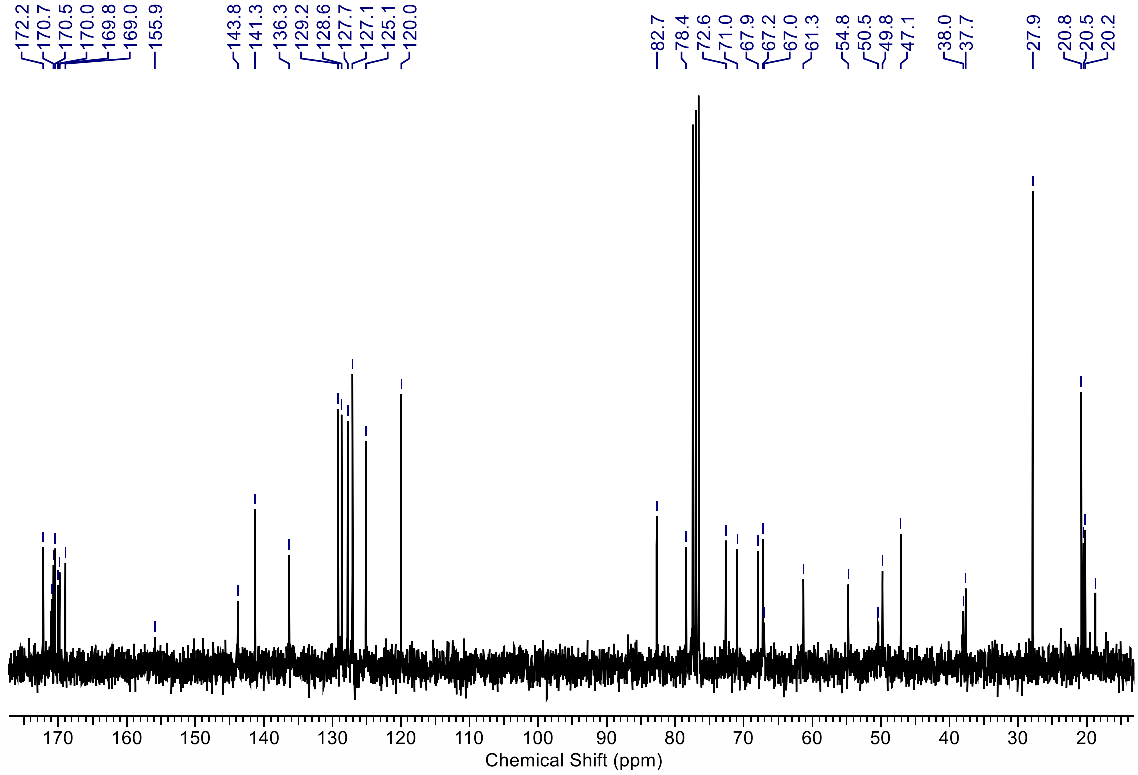
172.2 170.7 170.5 170.0 169.8 169.0 155.9
143.8 141.3 136.3 129.2 128.6 127.7 127.1 125.1 120.0
82.7 78.4 72.6 71.0 67.9 67.2 67.0 61.3 54.8 50.5 49.8 47.1
38.0 37.7 27.9 20.8 20.5 20.2
170 160 150 140 130 120 110 100 90 80 70 60 50 40 30 20
Chemical Shift (ppm)

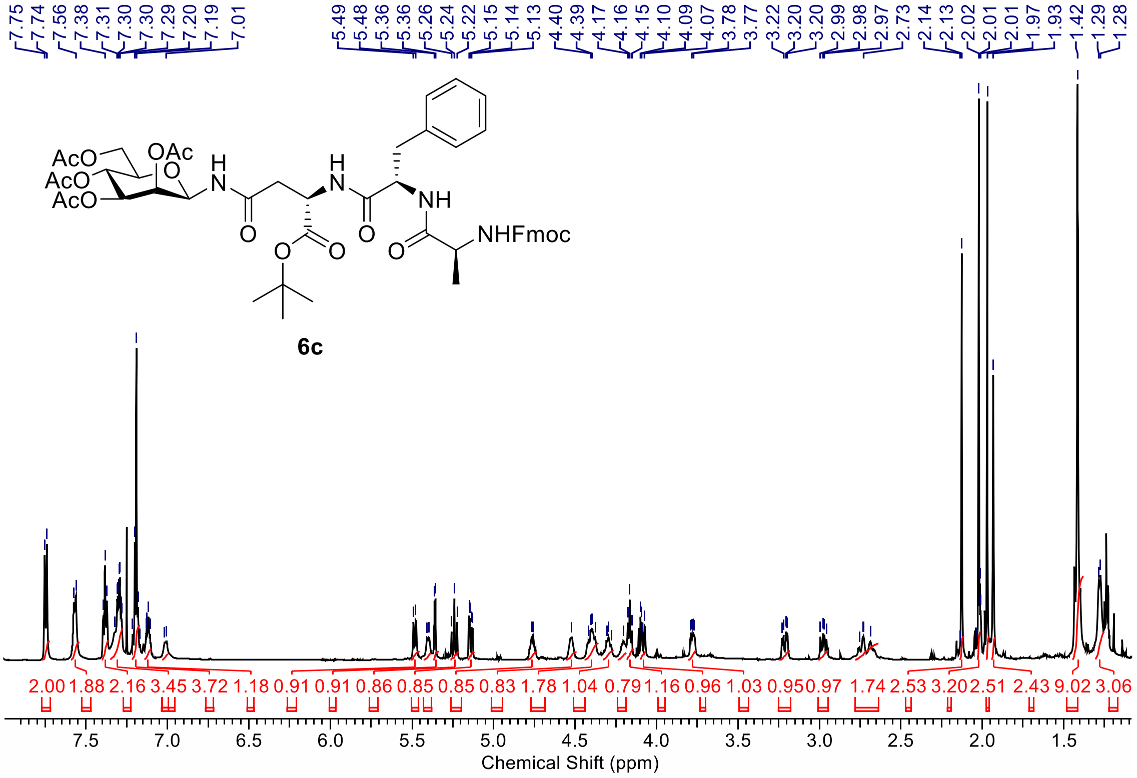
7.75 7.74 7.56 7.38 7.31 7.30 7.30 7.29 7.20 7.19 7.01
5.49 5.48 5.36 5.36 5.26 5.24 5.22 5.15 5.14 5.13 4.40 4.39 4.17 4.16 4.15 4.10 4.09 4.07 3.78 3.77 3.22 3.20 3.20 2.99 2.98 2.97 2.73 2.14 2.13 2.02 2.01 2.01 1.97 1.93 1.42 1.29 1.28
AcO
AcO
AcO
OAc
O
H
N
H
N
NH
NHFmoc
O
O
O
O
O
6c
2.00 1.88 2.16 3.45 3.72 1.18 0.91 0.91 0.86 0.85 0.85 0.83 1.78 1.04 0.79 1.16 0.96 1.03 0.95 0.97 1.74 2.53 3.20 2.51 2.43 9.02 3.06
7.5 7.0 6.5 6.0 5.5 5.0 4.5 4.0 3.5 3.0 2.5 2.0 1.5
Chemical Shift (ppm)

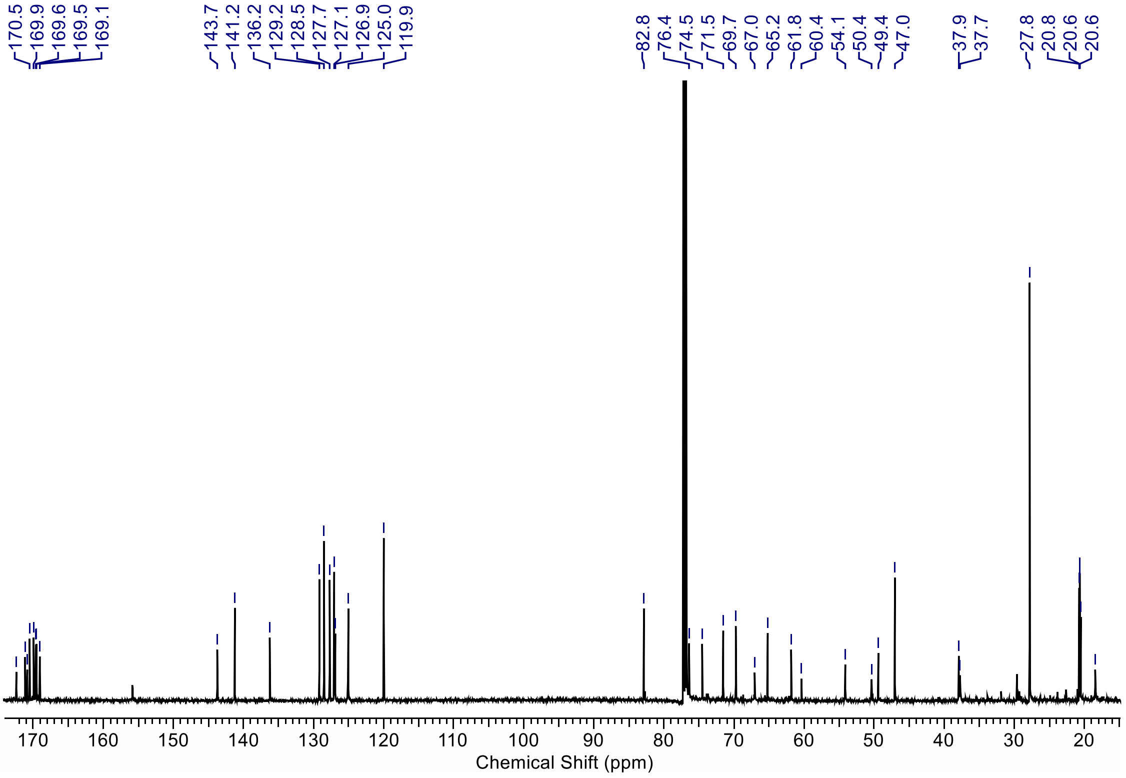
170.5 169.9 169.6 169.5 169.1
143.7 141.2 136.2 129.2 128.5 127.7 127.1 126.9 125.0 119.9
82.8 76.4 74.5 71.5 69.7 67.0 65.2 61.8 60.4 54.1 50.4 49.4 47.0
37.9 37.7
27.8 20.8 20.6 20.6
170 160 150 140 130 120 110 100 90 80 70 60 50 40 30 20
Chemical Shift (ppm)

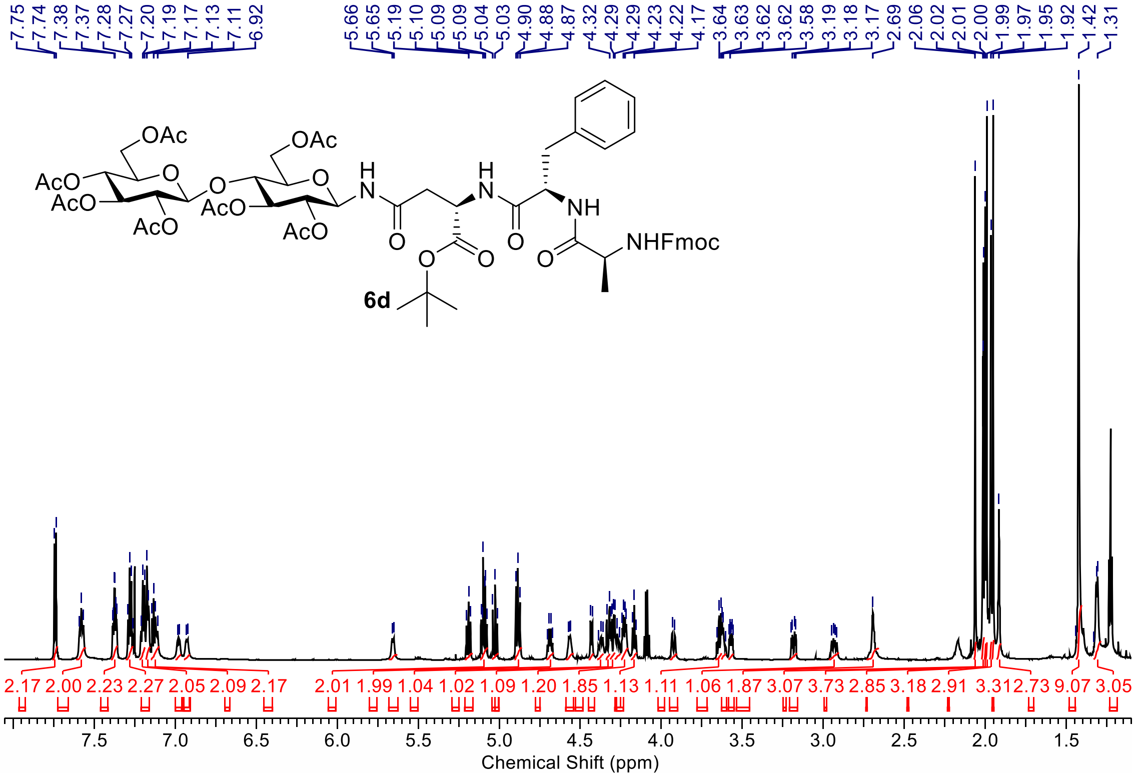
7.75 7.74 7.38 7.37 7.28 7.27 7.20 7.19 7.17 7.13 7.11 6.92
5.66 5.65 5.19 5.10 5.09 5.09 5.04 5.03 4.90 4.88 4.87 4.32 4.29 4.29 4.23 4.22 4.17 3.64 3.63 3.62 3.62 3.58 3.19 3.18 3.17 2.69 2.06 2.02 2.01 2.00 1.99 1.97 1.95 1.92 1.42 1.31
OAc
AcO
AcO
O
AcO
OAc
AcO
AcO
H
N
O
NH
NHFmoc
6d
2.17 2.00 2.23 2.27 2.05 2.09 2.17 2.01 1.99 1.04 1.02 1.09 1.20 1.85 1.13 1.11 1.06 1.87 3.07 3.73 2.85 3.18 2.91 3.31 2.73 9.07 3.05
7.5 7.0 6.5 6.0 5.5 5.0 4.5 4.0 3.5 3.0 2.5 2.0 1.5
Chemical Shift (ppm)

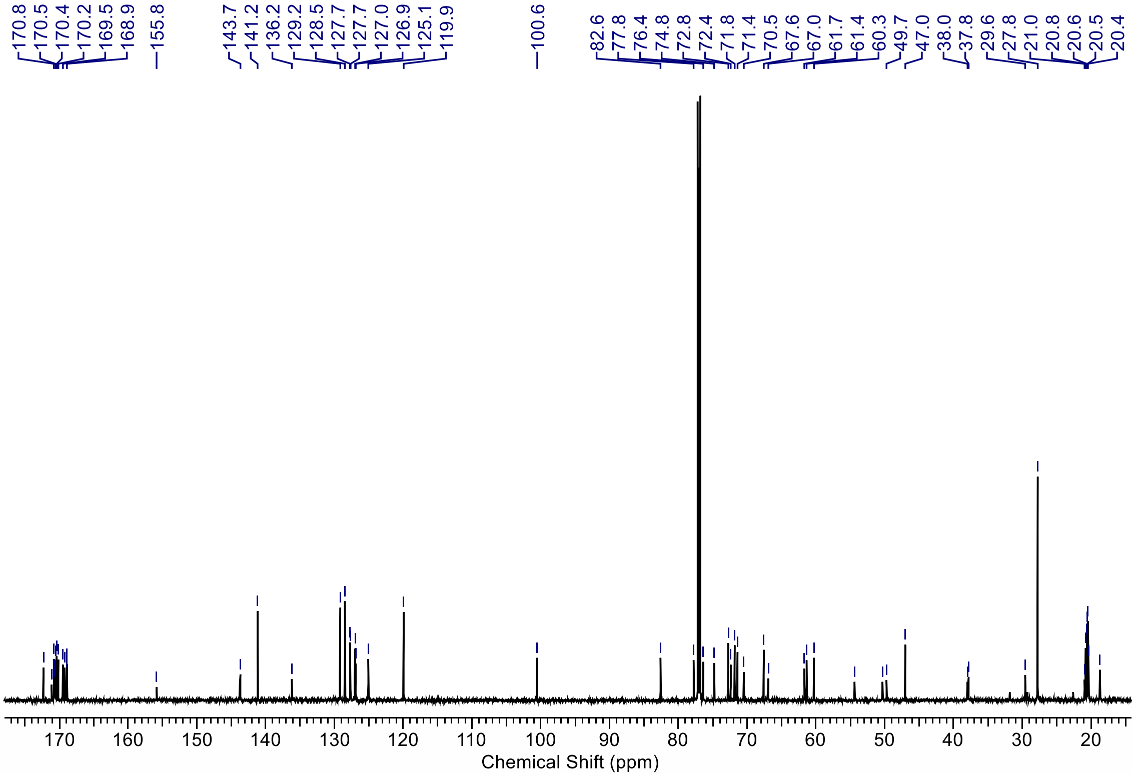
170.8 170.5 170.4 170.2 169.5 168.9 155.8 143.7 141.2 136.2 129.2 128.5 127.7 127.7 127.0 126.9 125.1 119.9 100.6 82.6 77.8 76.4 74.8 72.8 72.4 71.8 71.4 70.5 67.6 67.0 61.7 61.4 60.3 49.7 47.0 38.0 37.8 29.6 27.8 21.0 20.8 20.6 20.5 20.4
170 160 150 140 130 120 110 100 90 80 70 60 50 40 30 20
Chemical Shift (ppm)

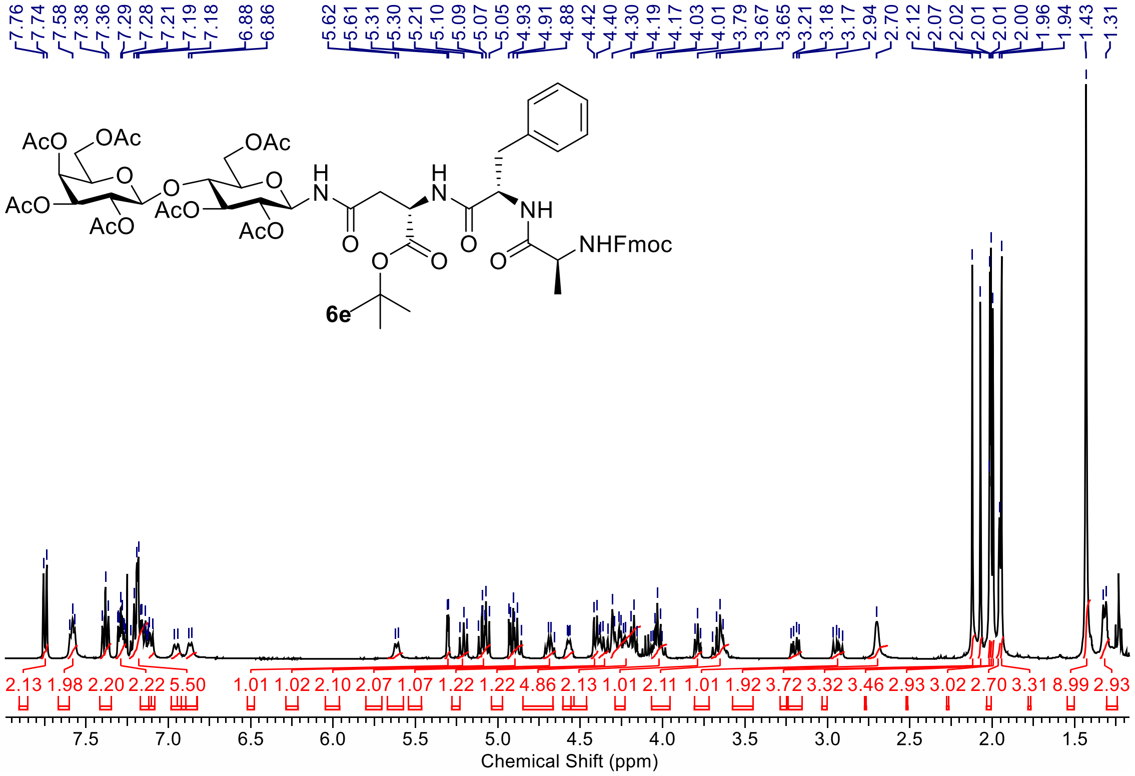
AcO
OAc
AcO
AcO
O
OAc
AcO
AcO
H
N
O
NH
NHFmoc
6e
Chemical Shift (ppm)

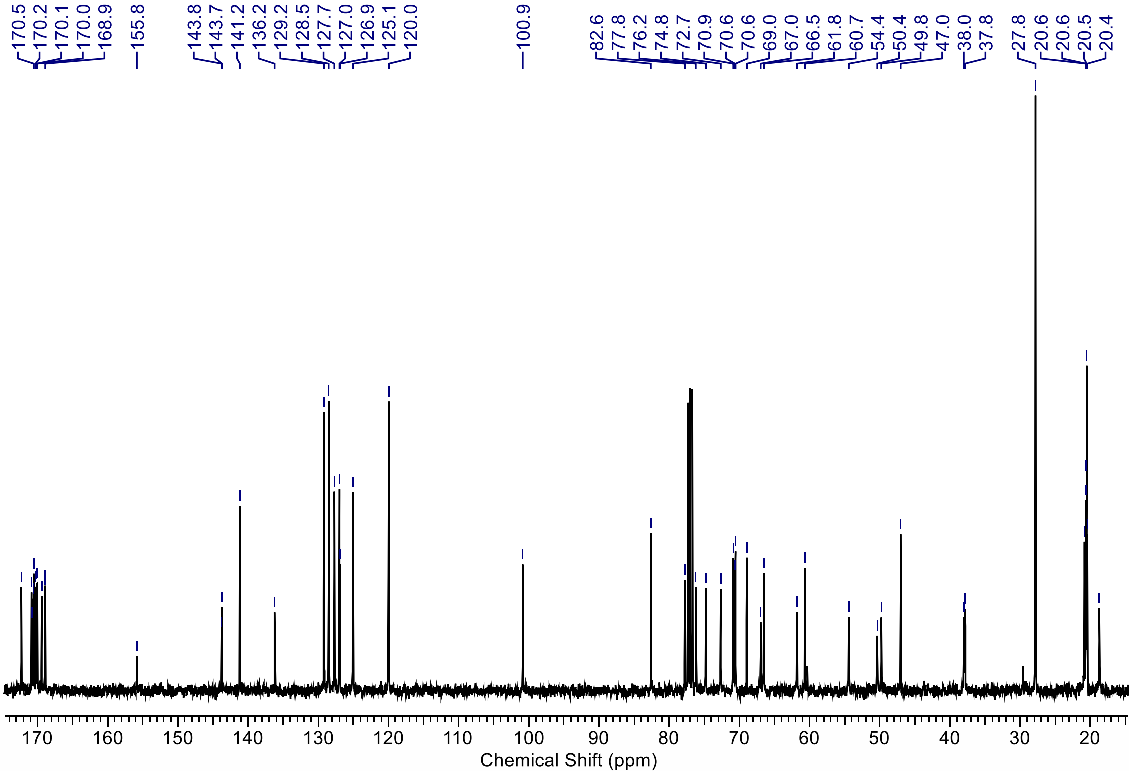
Chemical Shift (ppm)

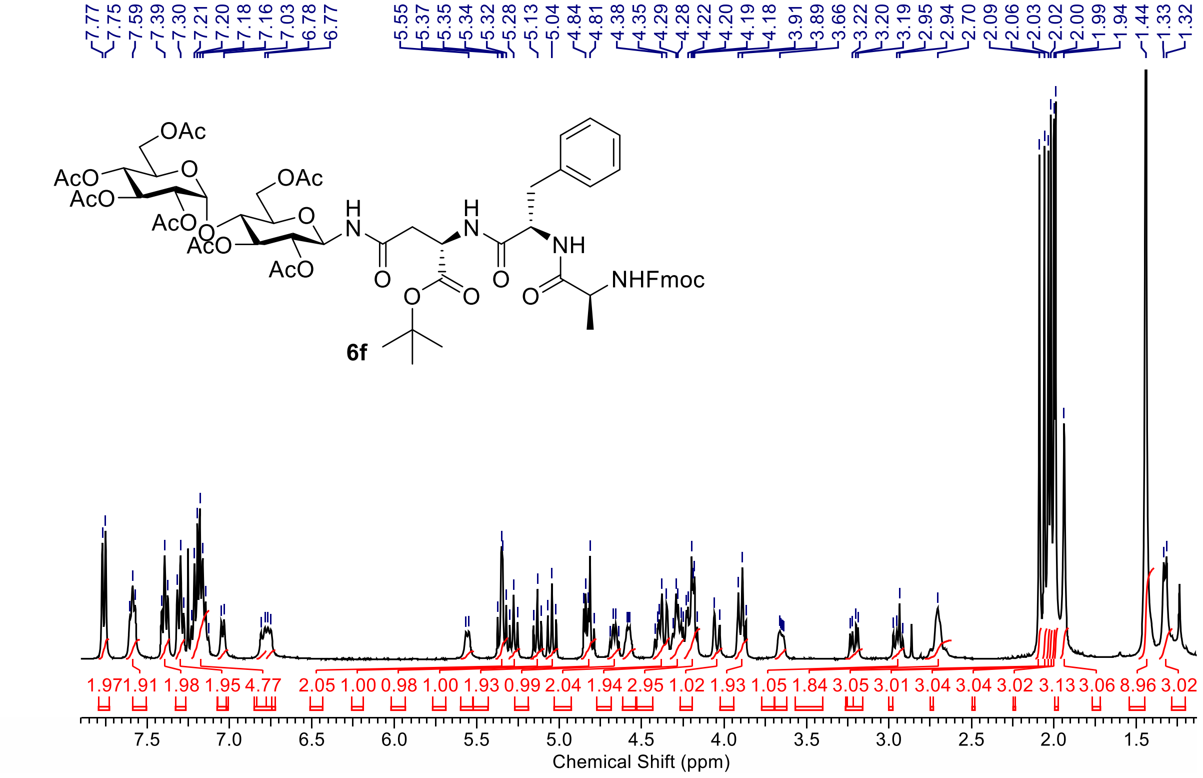
6f
NHFmoc
Chemical Shift (ppm)

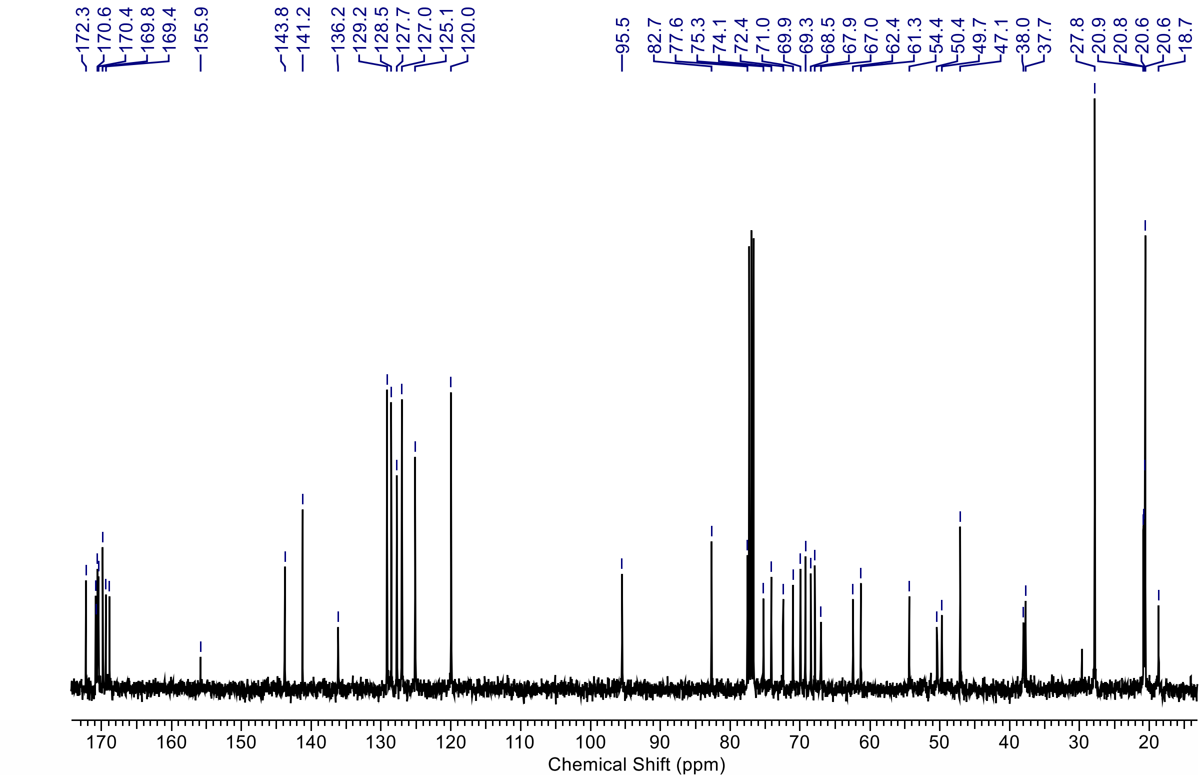
Chemical Shift (ppm)

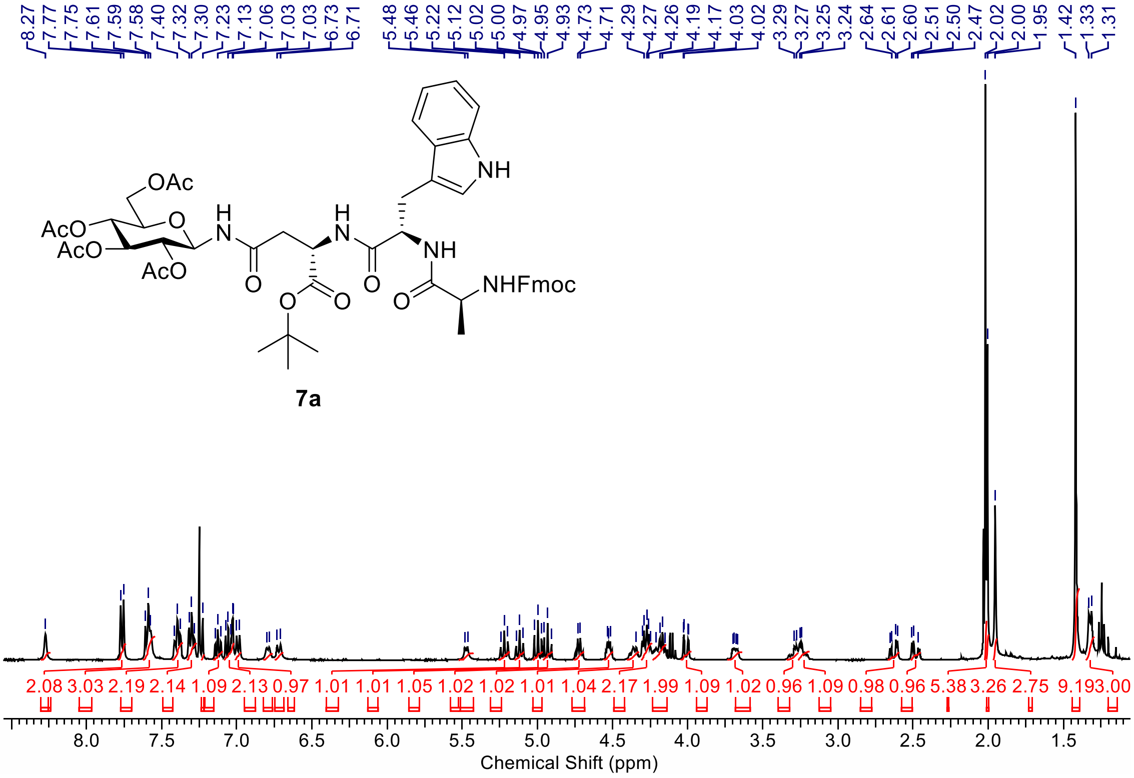

8.27 7.77 7.75 7.61 7.59 7.58 7.40 7.32 7.30 7.23 7.13 7.06 7.03 7.03 6.73 6.71 5.48 5.46 5.22 5.12 5.02 5.00 4.97 4.95 4.93 4.73 4.71 4.29 4.27 4.26 4.19 4.17 4.03 4.02 3.29 3.27 3.25 3.24 2.64 2.61 2.60 2.51 2.50 2.47 2.02 2.00 1.95 1.42 1.33 1.31
OAc
AcO
AcO
AcO
O
H
N
O
O
O
H
N
O
NH
NH
O
NHFmoc
7a
2.08 3.03 2.19 2.14 1.09 2.13 0.97 1.01 1.01 1.05 1.02 1.02 1.01 1.04 2.17 1.99 1.09 1.02 0.96 1.09 0.98 0.96 5.38 3.26 2.75 9.19 3.00
8.0 7.5 7.0 6.5 6.0 5.5 5.0 4.5 4.0 3.5 3.0 2.5 2.0 1.5
Chemical Shift (ppm)

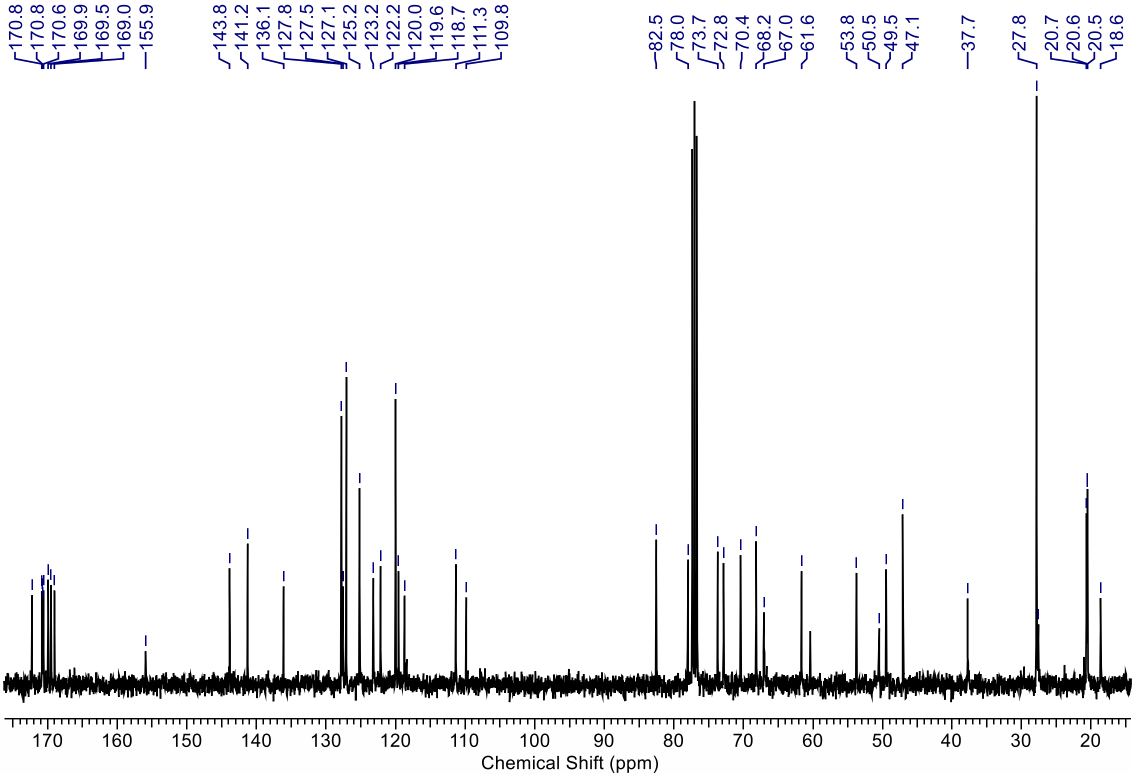

170.8 170.8 170.6 169.9 169.5 169.0 155.9 143.8 141.2 136.1 127.8 127.5 127.1 125.2 123.2 122.2 120.0 119.6 118.7 111.3 109.8 82.5 78.0 73.7 72.8 70.4 68.2 67.0 61.6 53.8 50.5 49.5 47.1 37.7 27.8 20.7 20.6 20.5 18.6
170 160 150 140 130 120 110 100 90 80 70 60 50 40 30 20
Chemical Shift (ppm)

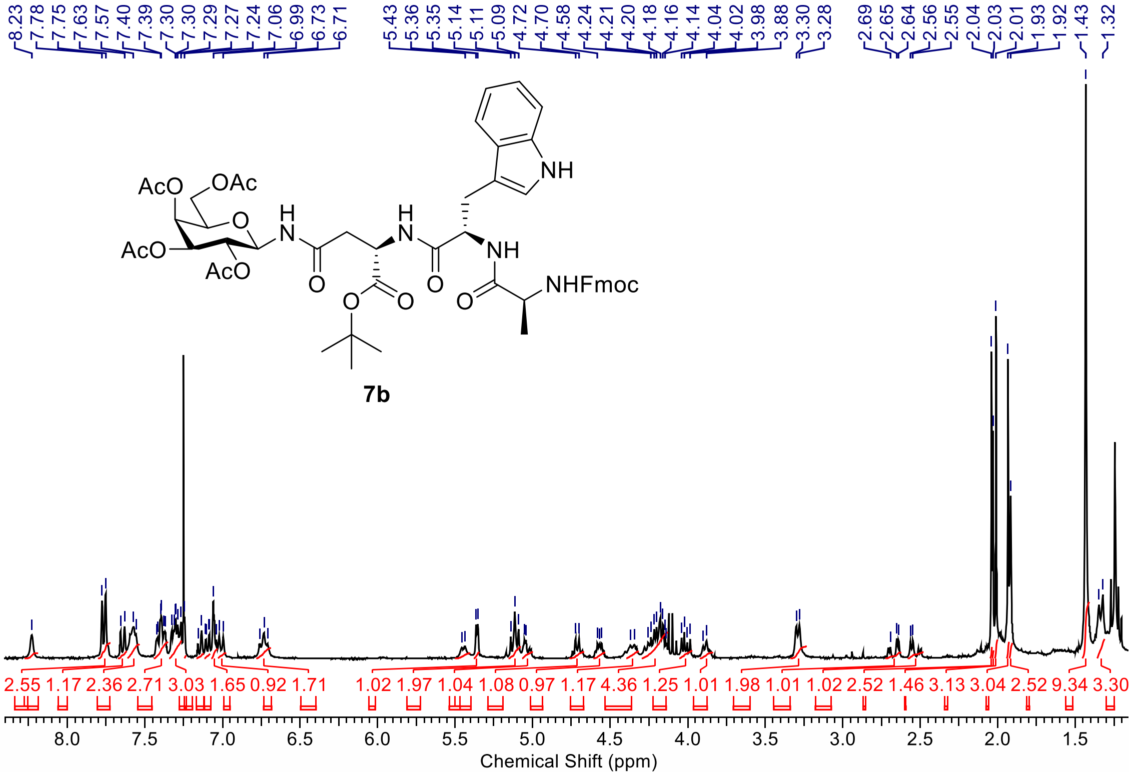
8.23
7.78
7.75
7.63
7.57
7.40
7.39
7.30
7.30
7.29
7.27
7.24
7.06
6.99
6.73
6.71
5.43
5.36
5.35
5.14
5.11
5.09
4.72
4.70
4.58
4.24
4.21
4.20
4.18
4.16
4.14
4.04
4.02
3.98
3.88
3.30
3.28
2.69
2.65
2.64
2.56
2.55
2.04
2.03
2.01
1.93
1.92
1.43
1.32
AcO
OAc
AcO
AcO
NH
NHFmoc
7b
2.55 1.17 2.36 2.71 3.03 1.65 0.92 1.71 1.02 1.97 1.04 1.08 0.97 1.17 4.36 1.25 1.01 1.98 1.01 1.02 2.52 1.46 3.13 3.04 2.52 9.34 3.30
Chemical Shift (ppm)

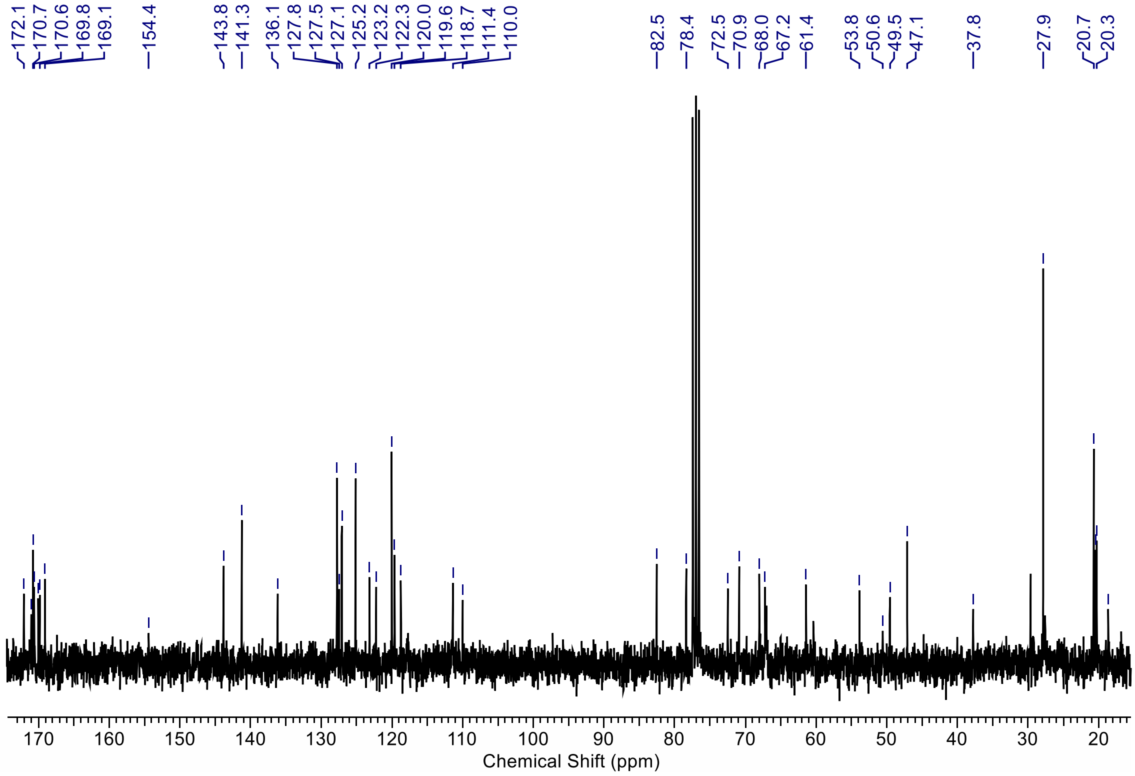
172.1
170.7
170.6
169.8
169.1
154.4
143.8
141.3
136.1
127.8
127.5
127.1
125.2
123.2
122.3
120.0
119.6
118.7
111.4
110.0
82.5
78.4
72.5
70.9
68.0
67.2
61.4
53.8
50.6
49.5
47.1
37.8
27.9
20.7
20.3
Chemical Shift (ppm)

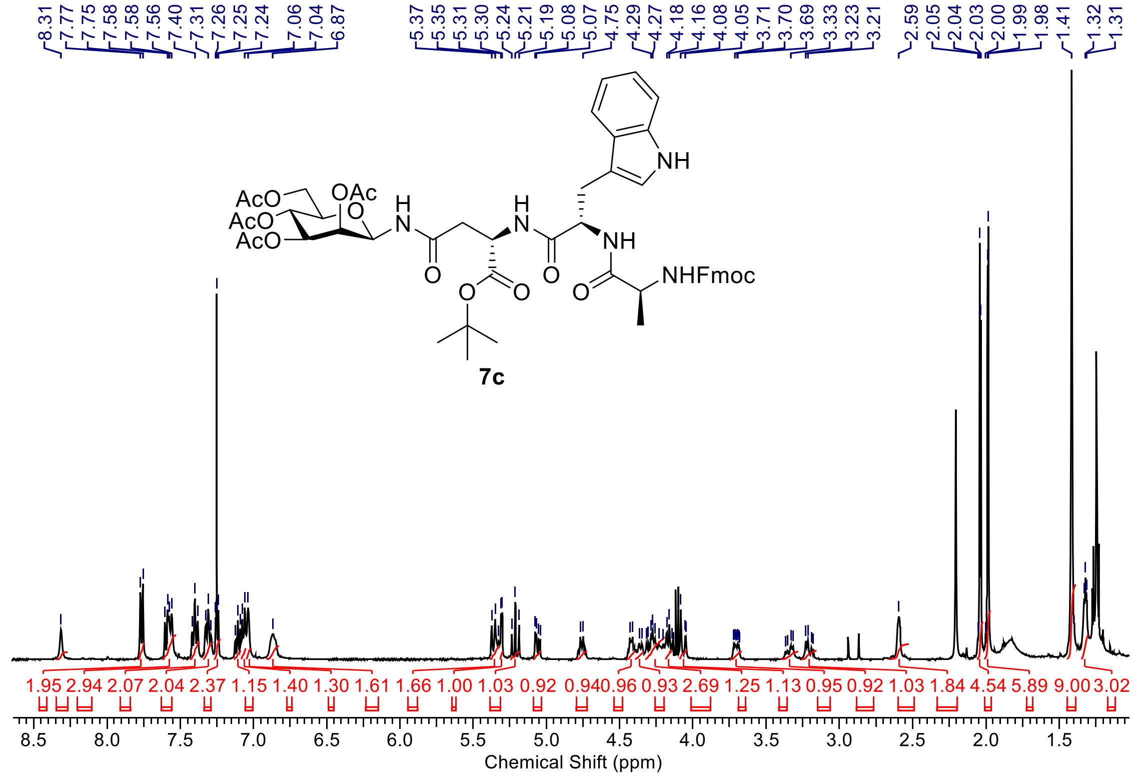
8.31 7.77 7.75 7.58 7.58 7.56 7.40 7.31 7.26 7.25 7.24 7.06 7.04 6.87
5.37 5.35 5.31 5.30 5.24 5.21 5.19 5.08 5.07 4.75 4.29 4.27 4.18 4.16 4.08 4.05 3.71 3.70 3.69 3.33 3.23 3.21
2.59 2.05 2.04 2.03 2.00 1.99 1.98 1.41 1.32 1.31
AcO
AcO
AcO
OAc
O
H
N
NH
NHFmoc
7c
1.95 2.94 2.07 2.04 2.37 1.15 1.40 1.30 1.61 1.66 1.00 1.03 0.92 0.94 0.96 0.93 2.69 1.25 1.13 0.95 0.92 1.03 1.84 4.54 5.89 9.00 3.02
Chemical Shift (ppm)

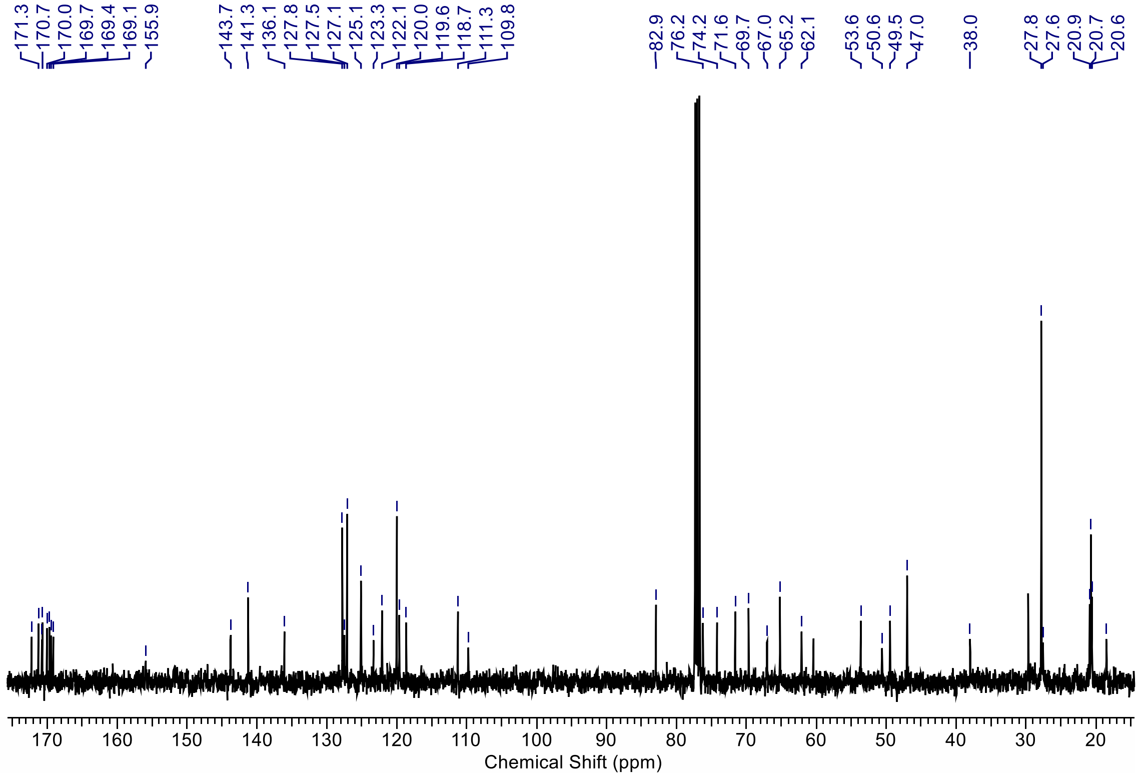
171.3 170.7 170.0 169.7 169.4 169.1 155.9
143.7 141.3 136.1 127.8 127.5 127.1 125.1 123.3 122.1 120.0 119.6 118.7 111.3 109.8
82.9 76.2 74.2 71.6 69.7 67.0 65.2 62.1
53.6 50.6 49.5 47.0
38.0
27.8 27.6 20.9 20.7 20.6
Chemical Shift (ppm)

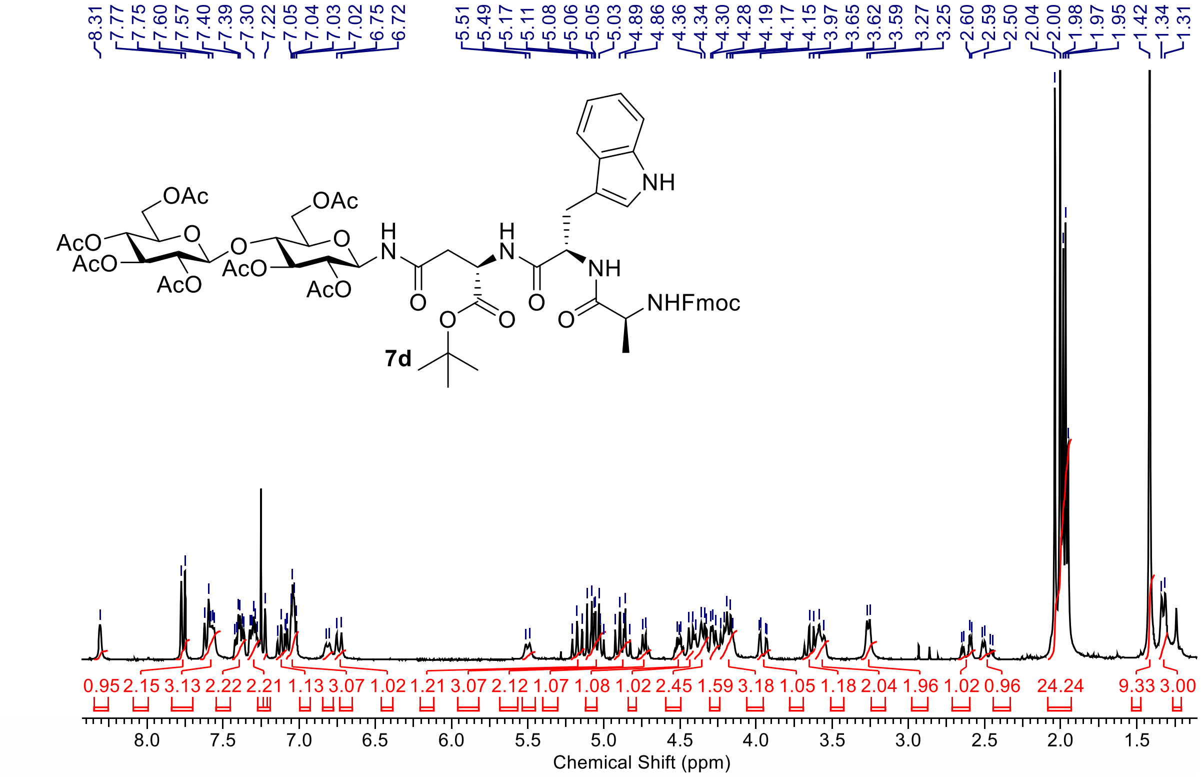

8.31 7.77 7.75 7.60 7.57 7.40 7.39 7.30 7.22 7.05 7.04 7.03 7.02 6.75 6.72
5.51 5.49 5.17 5.11 5.08 5.06 5.05 5.03 4.89 4.86 4.36 4.34 4.30 4.28 4.19 4.17 4.15 3.97 3.65 3.62 3.59 3.27 3.25 2.60 2.59 2.50 2.04 2.00 1.98 1.97 1.95 1.42 1.34 1.31
AcO
AcO
OAc
AcO
O
O
OAc
AcO
AcO
H
N
NH
NH
NHFmoc
7d
0.95 2.15 3.13 2.22 2.21 1.13 3.07 1.02 1.21 3.07 2.12 1.07 1.08 1.02 2.45 1.59 3.18 1.05 1.18 2.04 1.96 1.02 0.96 24.24 9.33 3.00
Chemical Shift (ppm)

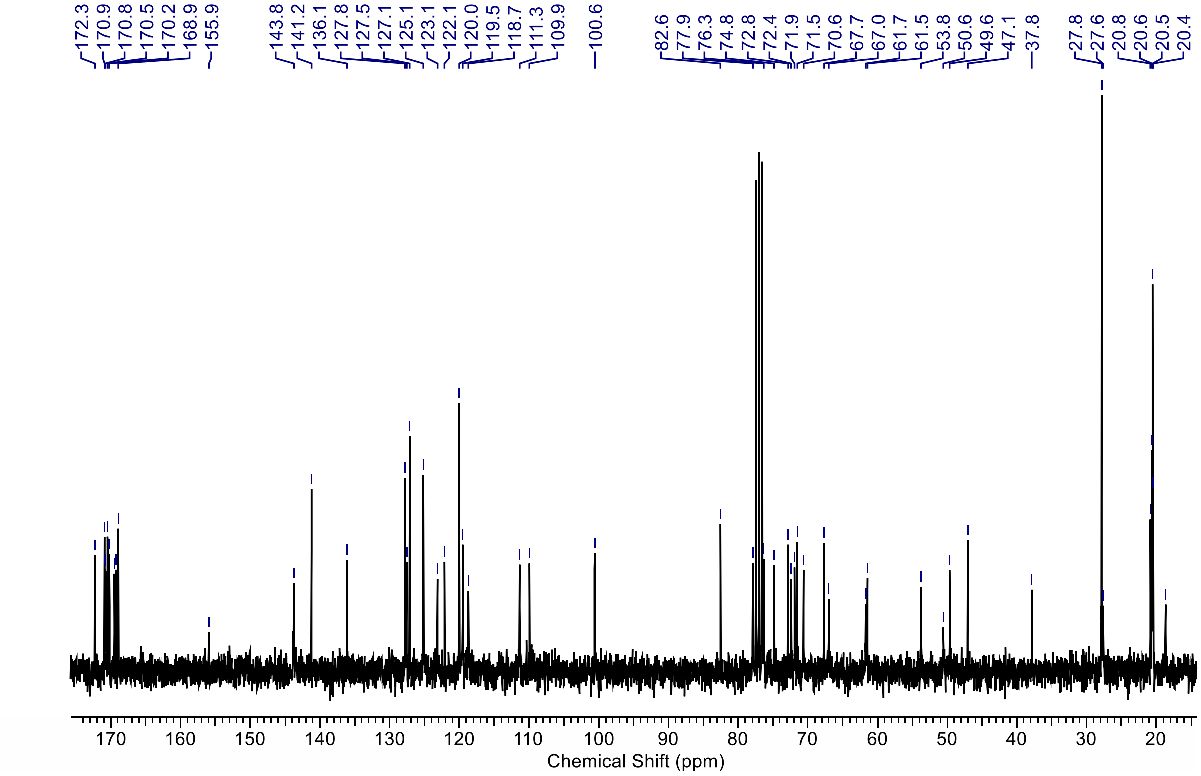

172.3 170.9 170.8 170.5 170.2 168.9 155.9 143.8 141.2 136.1 127.8 127.5 127.1 125.1 123.1 122.1 120.0 119.5 118.7 111.3 109.9 100.6 82.6 77.9 76.3 74.8 72.8 72.4 71.9 71.5 70.6 67.7 67.0 61.7 61.5 53.8 50.6 49.6 47.1 37.8 27.8 27.6 20.8 20.6 20.5 20.4
Chemical Shift (ppm)

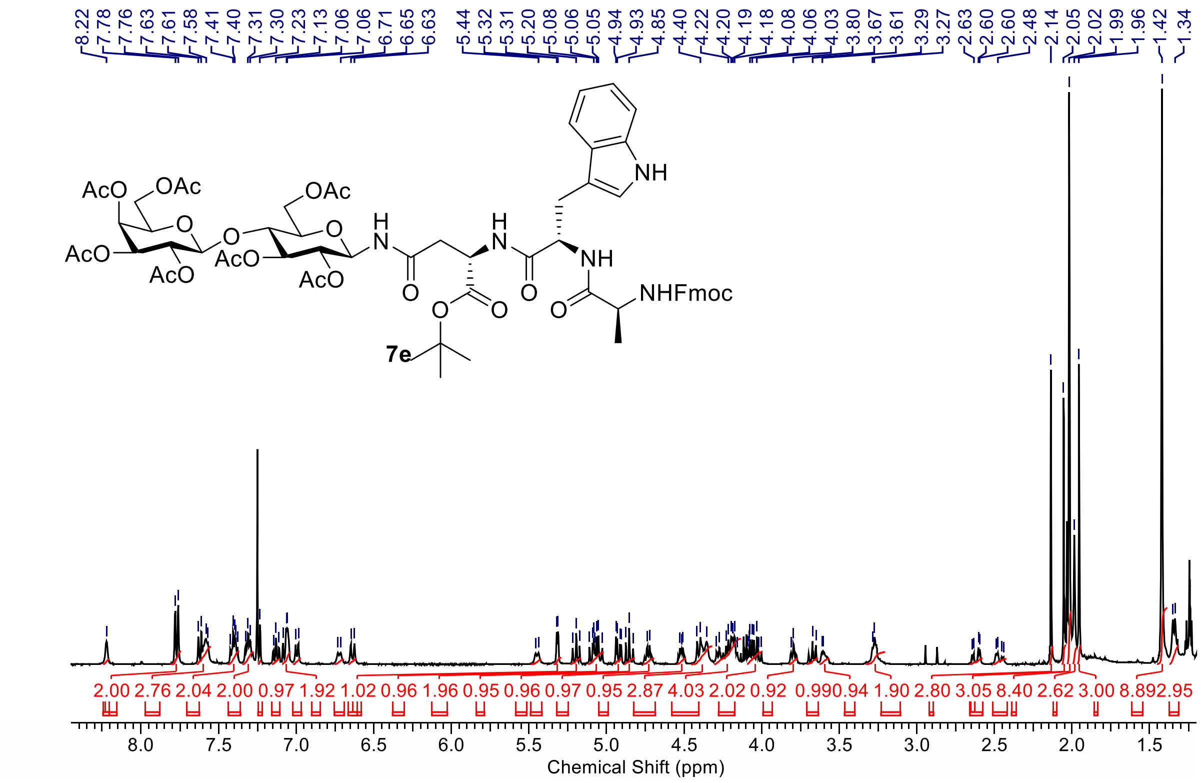

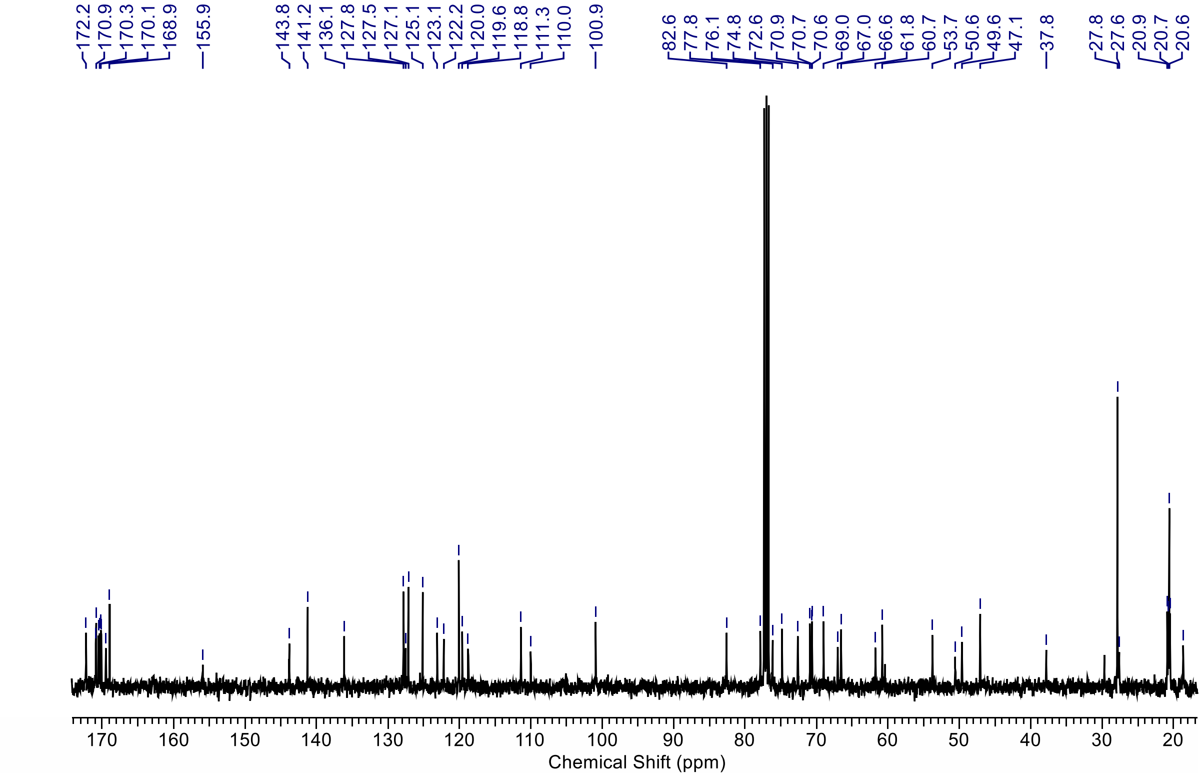

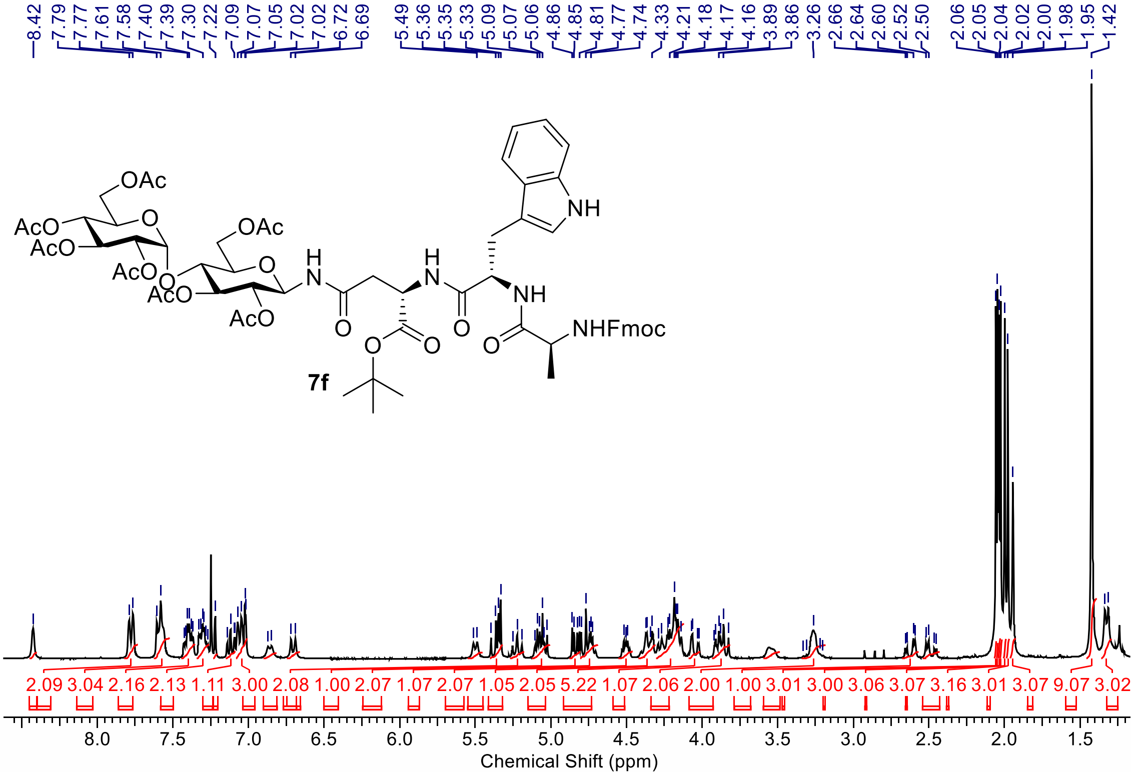

8.42 7.79 7.77 7.61 7.58 7.40 7.39 7.30 7.22 7.09 7.07 7.05 7.02 7.02 6.72 6.69 5.49 5.36 5.35 5.33 5.09 5.07 5.06 4.86 4.85 4.81 4.77 4.74 4.33 4.21 4.18 4.17 4.16 3.89 3.86 3.26 2.66 2.64 2.60 2.52 2.50 2.06 2.05 2.04 2.02 2.00 1.98 1.95 1.42
OAc
AcO
AcO
AcO
O
AcO
AcO
OAc
O
H
N
O
H
N
O
NH
NH
NHFmoc
O
O
7f
2.09 3.04 2.16 2.13 1.11 3.00 2.08 1.00 2.07 1.07 2.07 1.05 2.05 5.22 1.07 2.06 2.00 1.00 3.01 3.00 3.06 3.07 3.16 3.01 3.07 9.07 3.02
8.0 7.5 7.0 6.5 6.0 5.5 5.0 4.5 4.0 3.5 3.0 2.5 2.0 1.5
Chemical Shift (ppm)

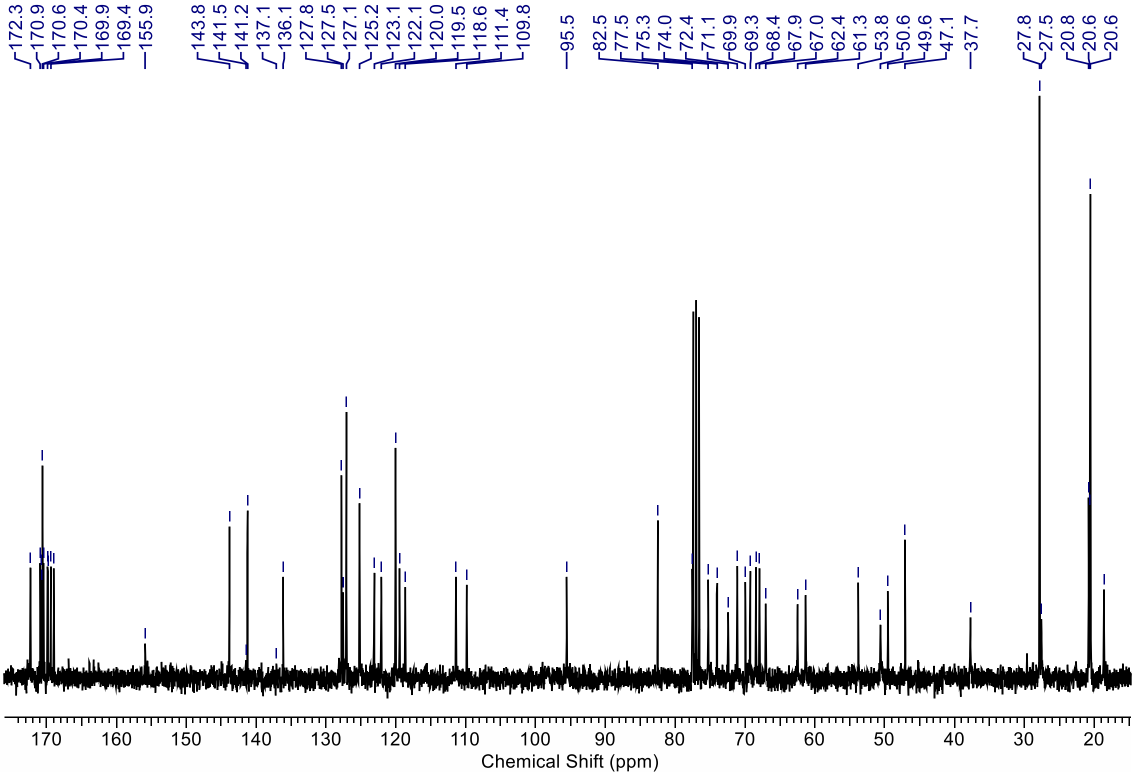

172.3 170.9 170.6 170.4 169.9 169.4 155.9 143.8 141.5 141.2 137.1 136.1 127.8 127.5 127.1 125.2 123.1 122.1 120.0 119.5 118.6 111.4 109.8 95.5 82.5 77.5 75.3 74.0 72.4 71.1 69.9 69.3 68.4 67.9 67.0 62.4 61.3 53.8 50.6 49.6 47.1 37.7 27.8 27.5 20.8 20.6 20.6
170 160 150 140 130 120 110 100 90 80 70 60 50 40 30 20
Chemical Shift (ppm)

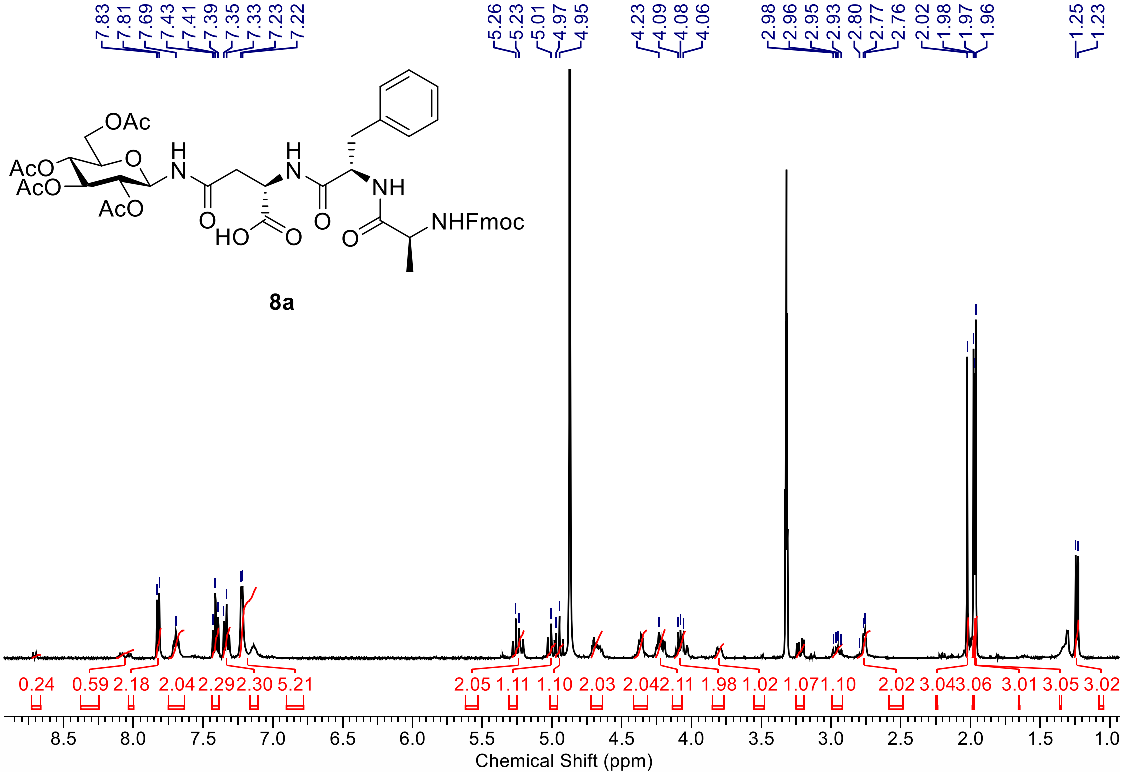

7.83 7.81 7.69 7.43 7.41 7.39 7.35 7.33 7.23 7.22
5.26 5.23 5.01 4.97 4.95
4.23 4.09 4.08 4.06
2.98 2.96 2.95 2.93 2.80 2.77 2.76 2.02 1.98 1.97 1.96
1.25 1.23
OAc
AcO
AcO
AcO
O
H
N
H
N
NH
NHFmoc
HO
O
8a
0.24 0.59 2.18 2.04 2.29 2.30 5.21
2.05 1.11 1.10 2.03 2.04 2.11 1.98 1.02 1.07 1.10 2.02 3.04 3.06 3.01 3.05 3.02
8.5 8.0 7.5 7.0 6.5 6.0 5.5 5.0 4.5 4.0 3.5 3.0 2.5 2.0 1.5 1.0
Chemical Shift (ppm)

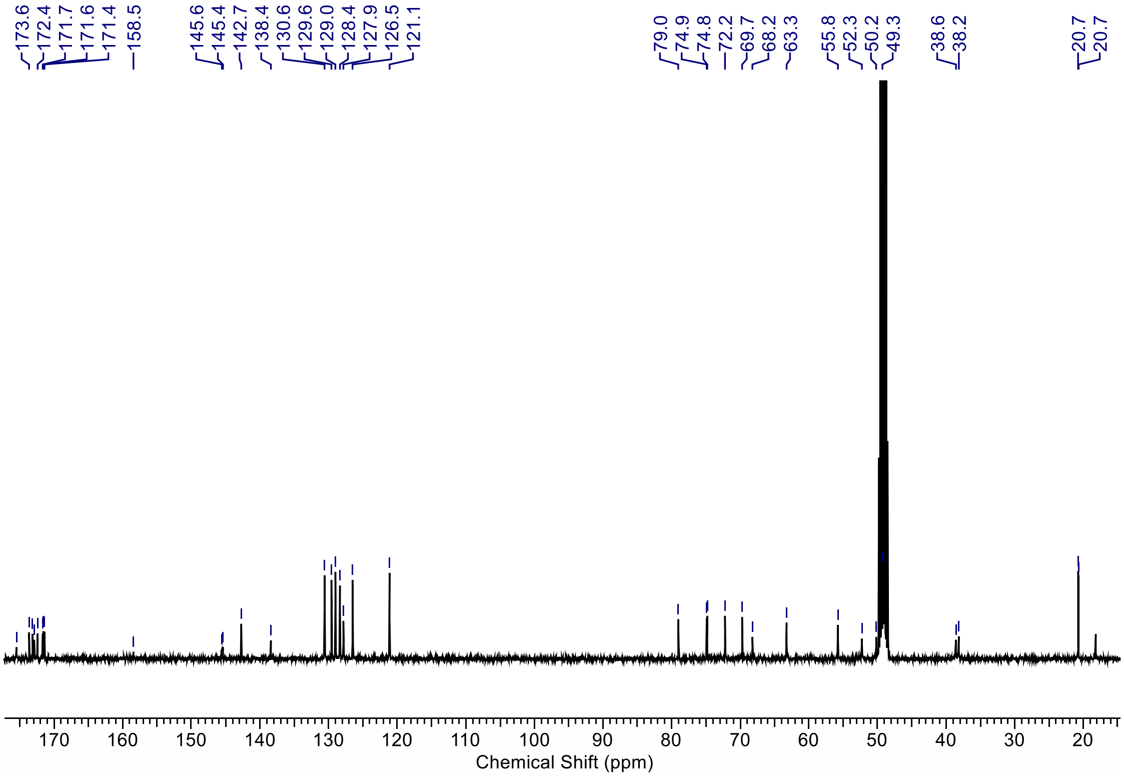

173.6 172.4 171.7 171.6 171.4 158.5
145.6 145.4 142.7 138.4 130.6 129.6 129.0 128.4 127.9 126.5 121.1
79.0 74.9 74.8 72.2 69.7 68.2 63.3
55.8 52.3 50.2 49.3 38.6 38.2
20.7 20.7
170 160 150 140 130 120 110 100 90 80 70 60 50 40 30 20
Chemical Shift (ppm)

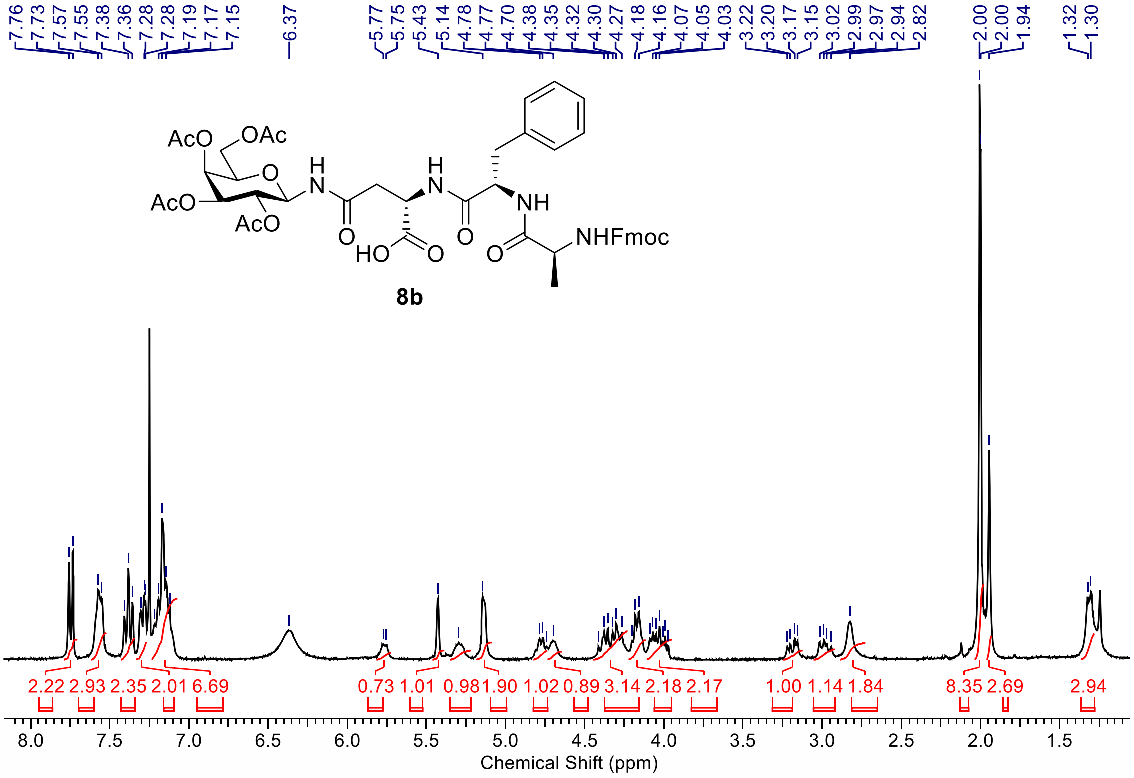
7.76
7.73
7.57
7.55
7.38
7.36
7.28
7.28
7.19
7.17
7.15
6.37
5.77
5.75
5.43
5.14
4.78
4.77
4.70
4.38
4.35
4.32
4.30
4.27
4.18
4.16
4.07
4.05
4.03
3.22
3.20
3.17
3.15
3.02
2.99
2.97
2.94
2.82
2.00
2.00
1.94
1.32
1.30
AcO
OAc
O
H
N
H
N
NH
AcO
AcO
O
HO
O
O
O
NHFmoc
8b
2.22 2.93 2.35 2.01 6.69
0.73 1.01 0.98 1.90 1.02 0.89 3.14 2.18 2.17
1.00 1.14 1.84
8.35 2.69
2.94
8.0 7.5 7.0 6.5 6.0 5.5 5.0 4.5 4.0 3.5 3.0 2.5 2.0 1.5
Chemical Shift (ppm)

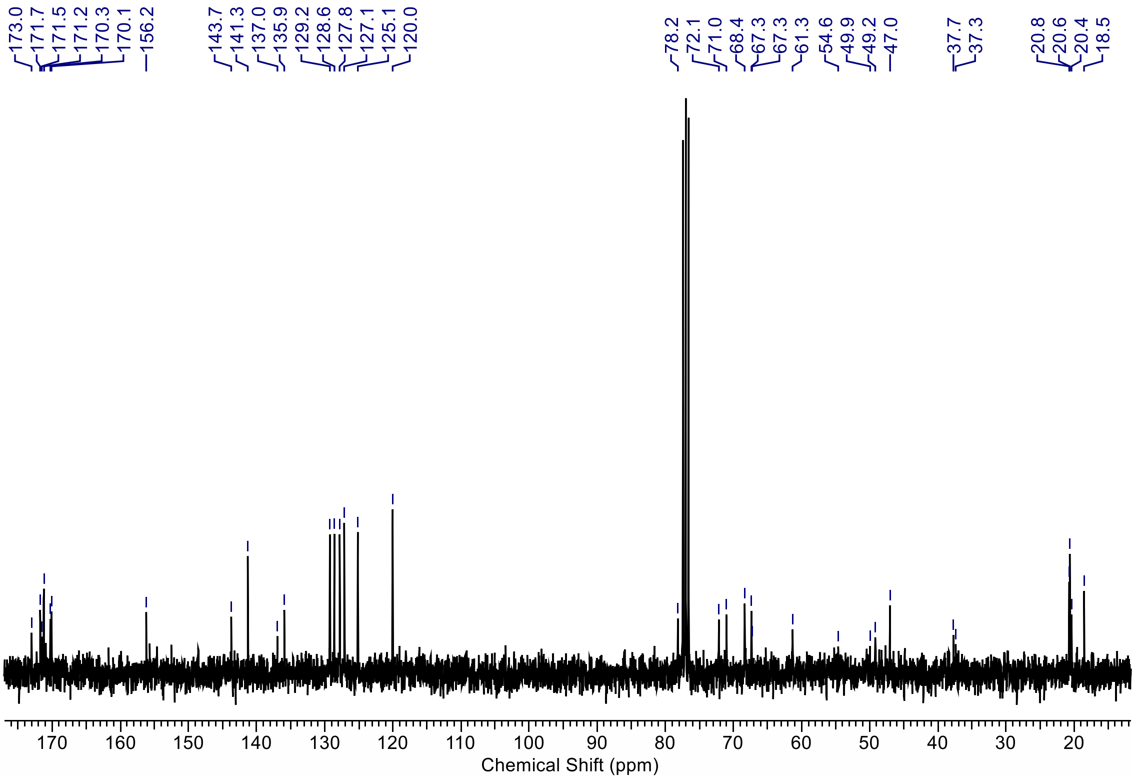
173.0
171.7
171.5
171.2
170.3
170.1
156.2
143.7
141.3
137.0
135.9
129.2
128.6
127.8
127.1
125.1
120.0
78.2
72.1
71.0
68.4
67.3
67.3
61.3
54.6
49.9
49.2
47.0
37.7
37.3
20.8
20.6
20.4
18.5
170 160 150 140 130 120 110 100 90 80 70 60 50 40 30 20
Chemical Shift (ppm)

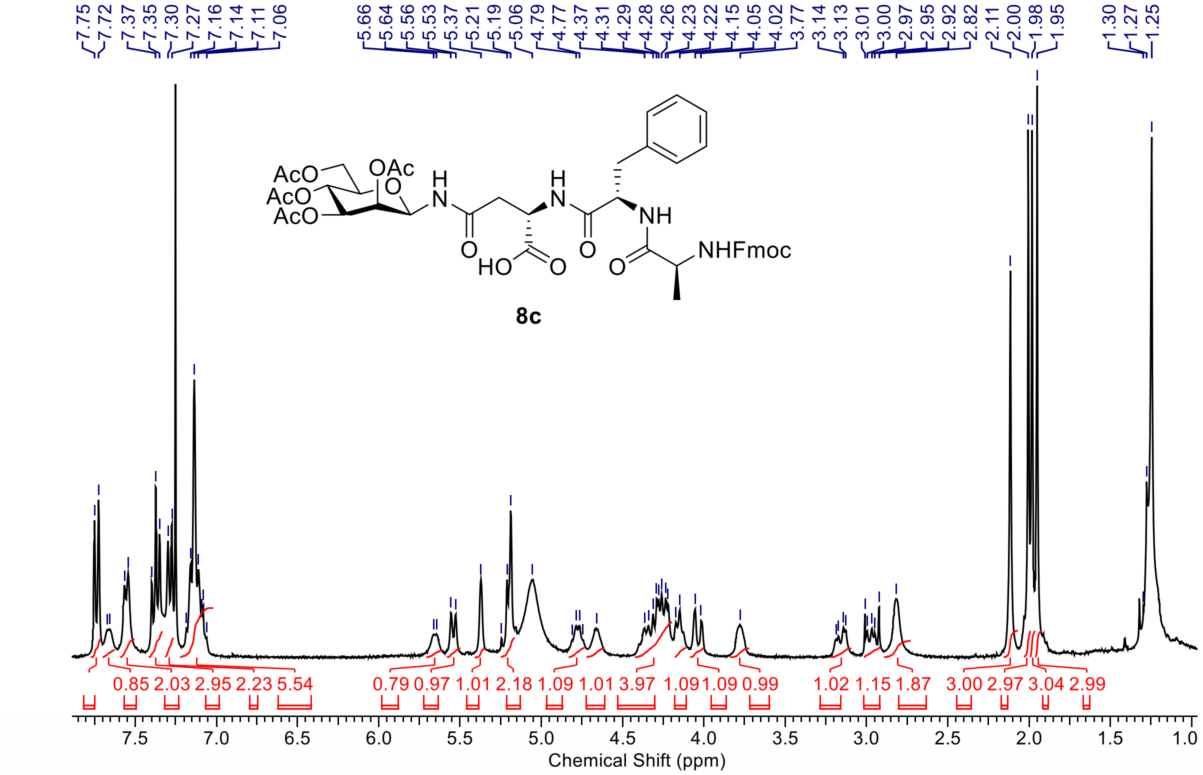
7.75 7.72 7.37 7.35 7.30 7.27 7.16 7.14 7.11 7.06
5.66 5.64 5.56 5.53 5.37 5.21 5.19 5.06 4.79 4.77 4.37 4.31 4.29 4.28 4.26 4.23 4.22 4.15 4.05 4.02 3.77 3.14 3.13 3.01 3.00 2.97 2.95 2.92 2.82 2.11 2.00 1.98 1.95
1.30 1.27 1.25
AcO
AcO
AcO
OAc
O
H
N
H
N
NH
NHFmoc
O
HO
O
O
O
8c
0.85 2.03 2.95 2.23 5.54
0.79 0.97 1.01 2.18 1.09 1.01 3.97 1.09 1.09 0.99
1.02 1.15 1.87 3.00 2.97 3.04 2.99
7.5 7.0 6.5 6.0 5.5 5.0 4.5 4.0 3.5 3.0 2.5 2.0 1.5 1.0
Chemical Shift (ppm)

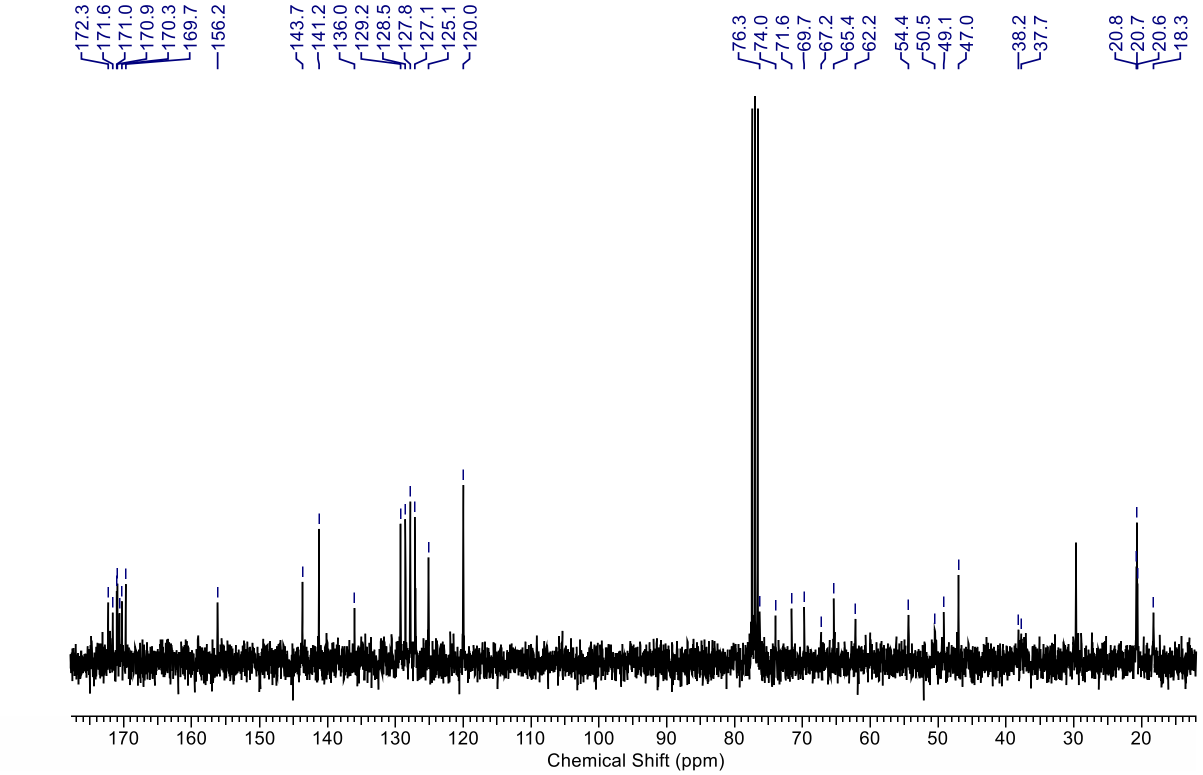
172.3 171.6 171.0 170.9 170.3 169.7 156.2
143.7 141.2 136.0 129.2 128.5 127.8 127.1 125.1 120.0
76.3 74.0 71.6 69.7 67.2 65.4 62.2
54.4 50.5 49.1 47.0
38.2 37.7
20.8 20.7 20.6 18.3
170 160 150 140 130 120 110 100 90 80 70 60 50 40 30 20
Chemical Shift (ppm)

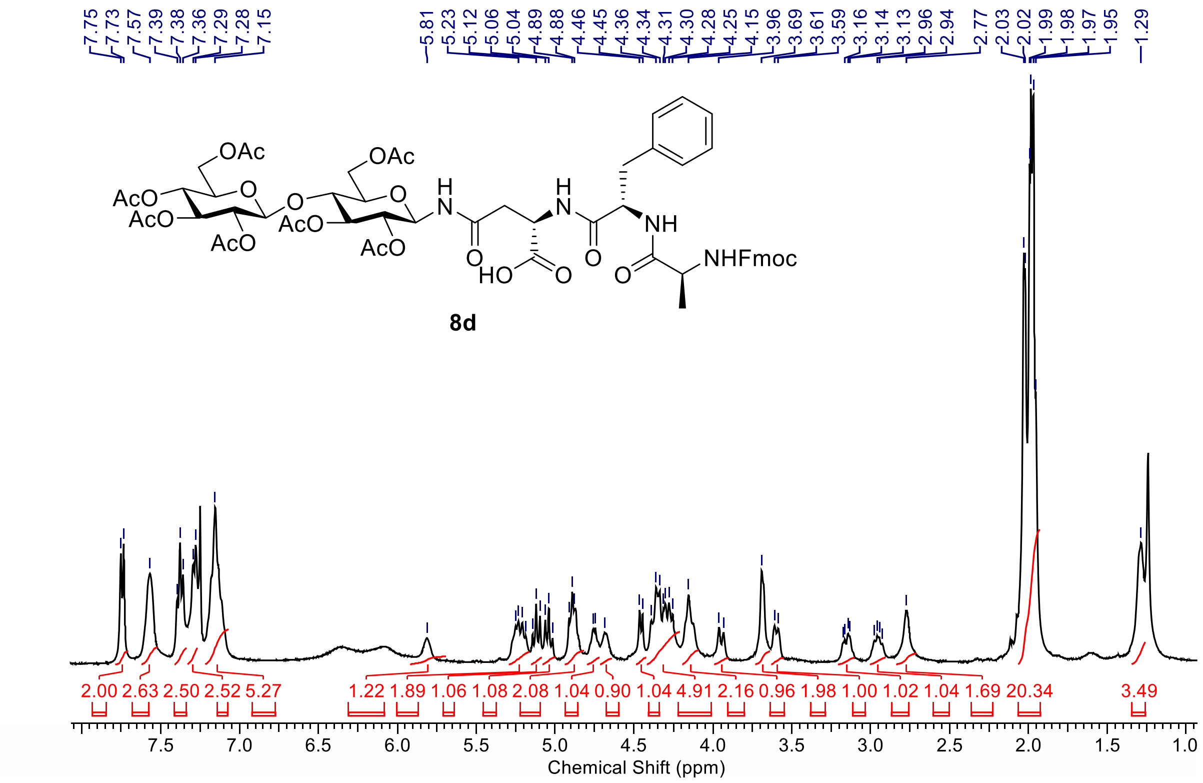
7.75
7.73
7.57
7.39
7.38
7.36
7.29
7.28
7.15
5.81
5.23
5.12
5.06
5.04
4.89
4.88
4.46
4.45
4.36
4.34
4.31
4.30
4.28
4.25
4.15
3.96
3.69
3.61
3.59
3.16
3.14
3.13
2.96
2.94
2.77
2.03
2.02
1.99
1.98
1.97
1.95
1.29
8d
2.00 2.63 2.50 2.52 5.27
1.22 1.89 1.06 1.08 2.08 1.04 0.90 1.04 4.91 2.16 0.96 1.98 1.00 1.02 1.04 1.69 20.34
3.49
Chemical Shift (ppm)

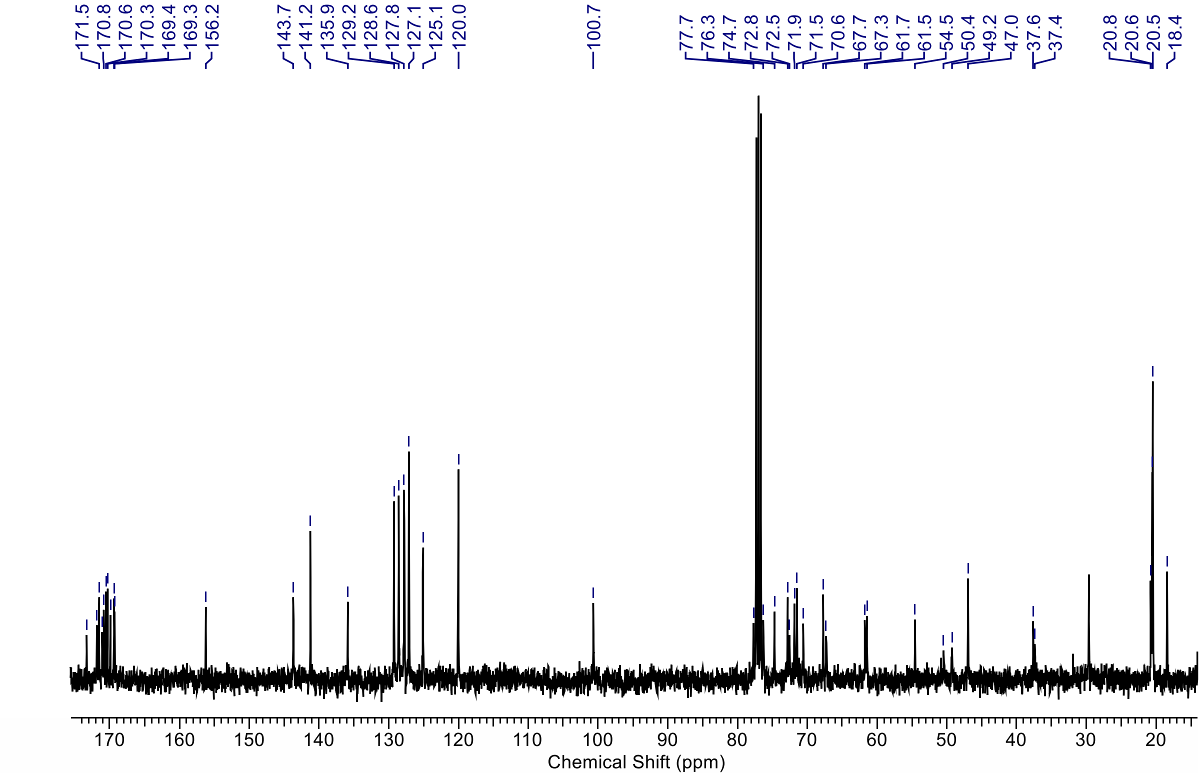
171.5
170.8
170.6
170.3
169.4
169.3
156.2
143.7
141.2
135.9
129.2
128.6
127.8
127.1
125.1
120.0
100.7
77.7
76.3
74.7
72.8
72.5
71.9
71.5
70.6
67.7
67.3
61.7
61.5
54.5
50.4
49.2
47.0
37.6
37.4
20.8
20.6
20.5
18.4
Chemical Shift (ppm)

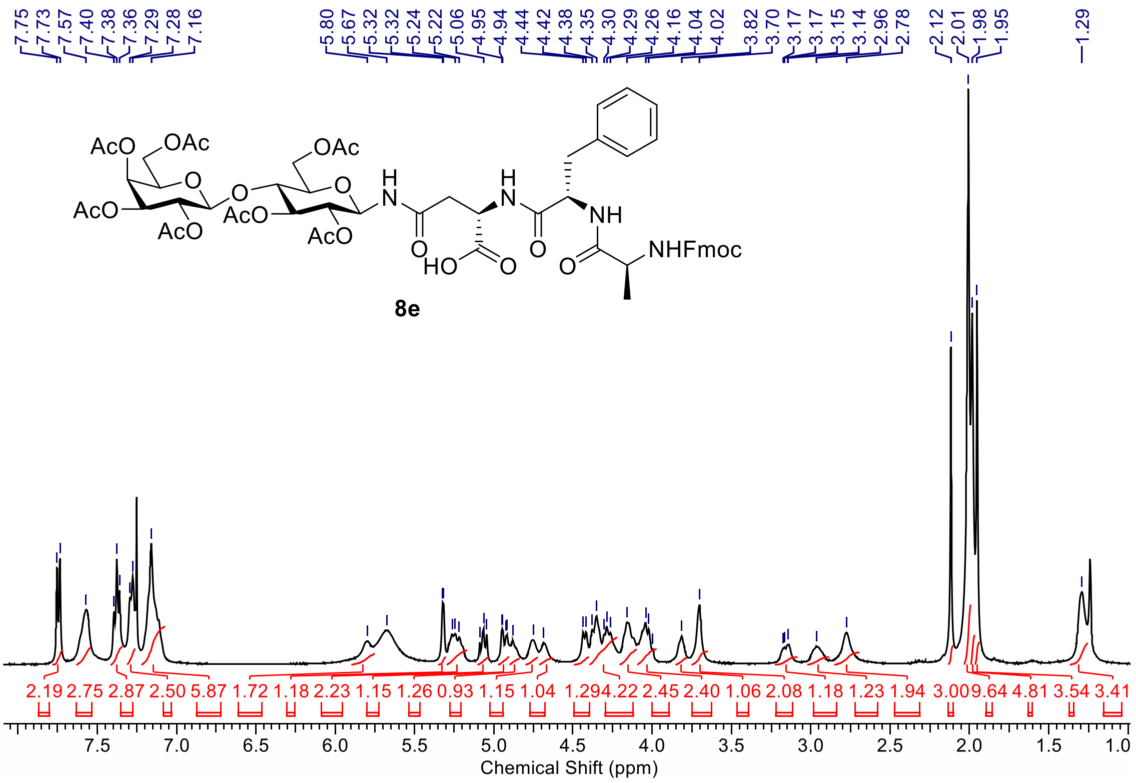
7.75 7.73 7.57 7.40 7.38 7.36 7.29 7.28 7.16
5.80 5.67 5.32 5.32 5.24 5.22 5.06 4.95 4.94 4.44 4.42 4.38 4.35 4.30 4.29 4.26 4.16 4.04 4.02 3.82 3.70 3.17 3.17 3.15 3.14 2.96 2.78
2.12 2.01 1.98 1.95
1.29
AcO
OAc
AcO
AcO
O
O
AcO
OAc
O
AcO
H
N
O
H
N
HO
O
O
NH
O
NHFmoc
8e
2.19 2.75 2.87 2.50 5.87 1.72 1.18 2.23 1.15 1.26 0.93 1.15 1.04 1.29 4.22 2.45 2.40 1.06 2.08 1.18 1.23 1.94 3.00 9.64 4.81 3.54 3.41
7.5 7.0 6.5 6.0 5.5 5.0 4.5 4.0 3.5 3.0 2.5 2.0 1.5 1.0
Chemical Shift (ppm)

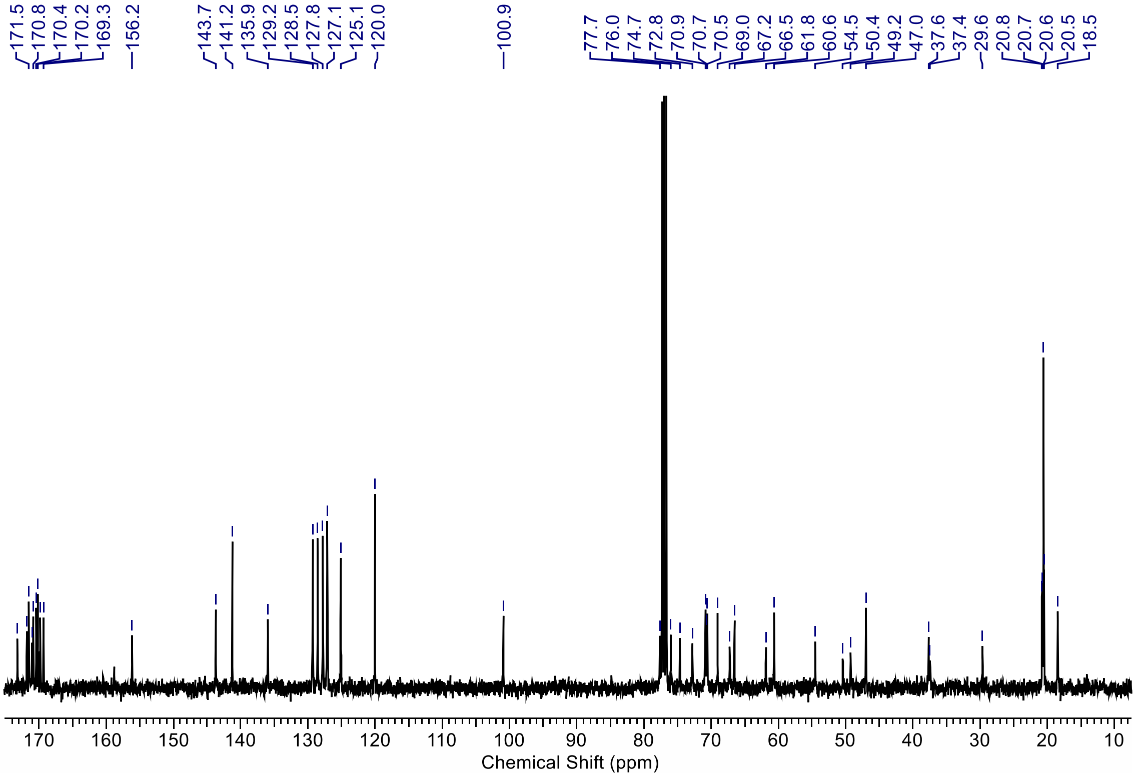
171.5 170.8 170.4 170.2 169.3 156.2
143.7 141.2 135.9 129.2 128.5 127.8 127.1 125.1 120.0
100.9
77.7 76.0 74.7 72.8 70.9 70.7 70.5 69.0 67.2 66.5 61.8 60.6 54.5 50.4 49.2 47.0 37.6 37.4 29.6 20.8 20.7 20.6 20.5 18.5
170 160 150 140 130 120 110 100 90 80 70 60 50 40 30 20 10
Chemical Shift (ppm)

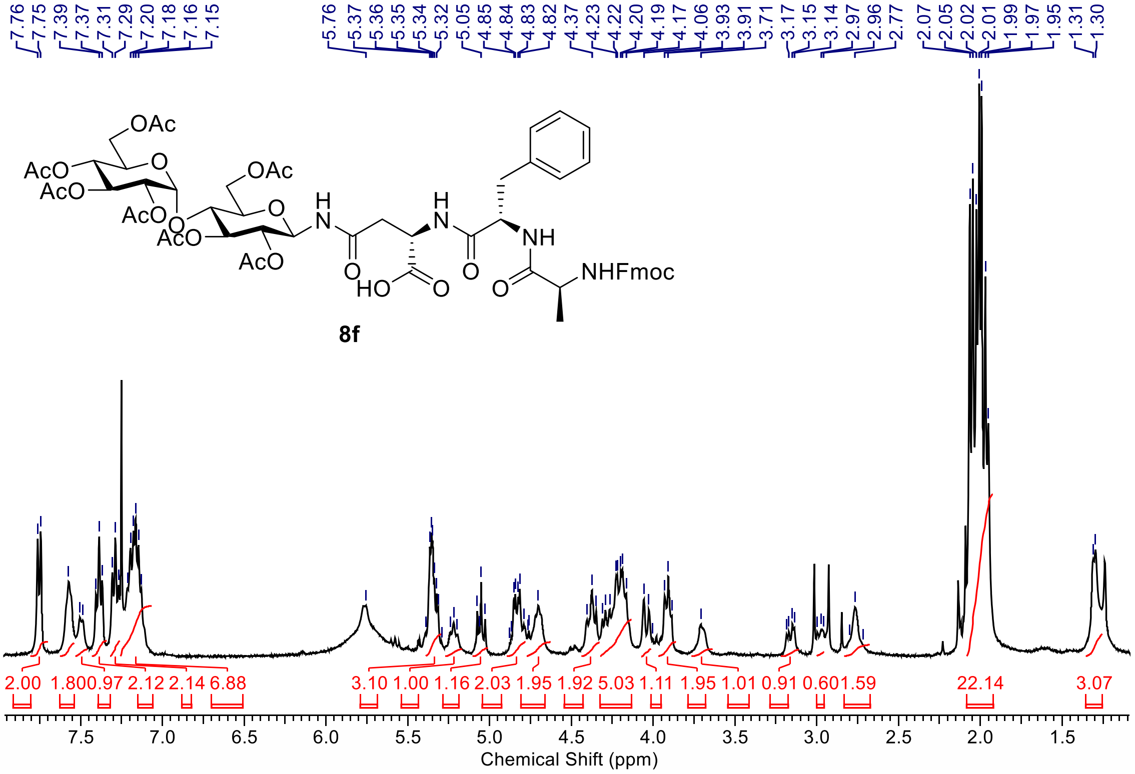
7.76
7.75
7.39
7.37
7.31
7.29
7.20
7.18
7.16
7.15
5.76
5.37
5.36
5.35
5.34
5.32
5.05
4.85
4.84
4.83
4.82
4.37
4.23
4.22
4.20
4.19
4.17
4.06
3.93
3.91
3.71
3.17
3.15
3.14
2.97
2.96
2.77
2.07
2.05
2.02
2.01
1.99
1.97
1.95
1.31
1.30
2.00 1.80 0.97 2.12 2.14 6.88
3.10 1.00 1.16 2.03 1.95 1.92 5.03 1.11 1.95 1.01 0.91 0.60 1.59
22.14
3.07
Chemical Shift (ppm)
OAc
AcO
AcO
AcO
O
OAc
AcO
O
AcO
H
N
O
N
H
NH
O
O
NHFmoc
HO
O

8f

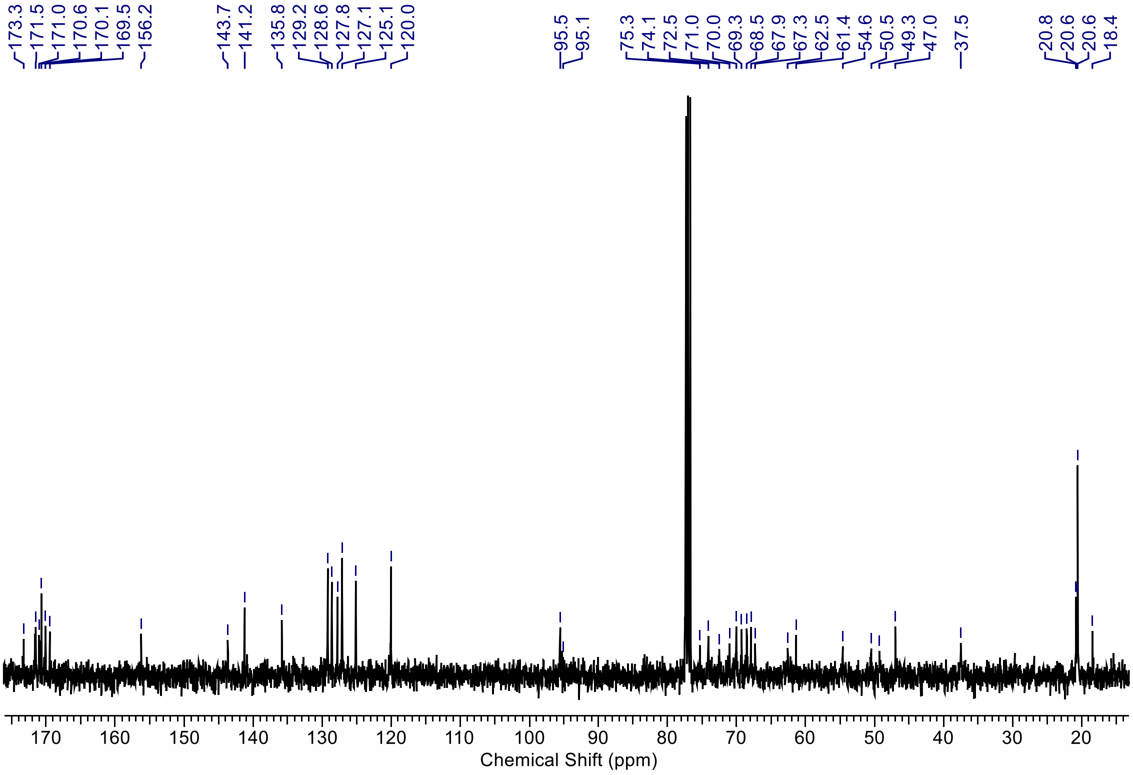
173.3
171.5
171.0
170.6
170.1
169.5
156.2
143.7
141.2
135.8
129.2
128.6
127.8
127.1
125.1
120.0
95.5
95.1
75.3
74.1
72.5
71.0
70.0
69.3
68.5
67.9
67.3
62.5
61.4
54.6
50.5
49.3
47.0
37.5
20.8
20.6
20.6
18.4
Chemical Shift (ppm)

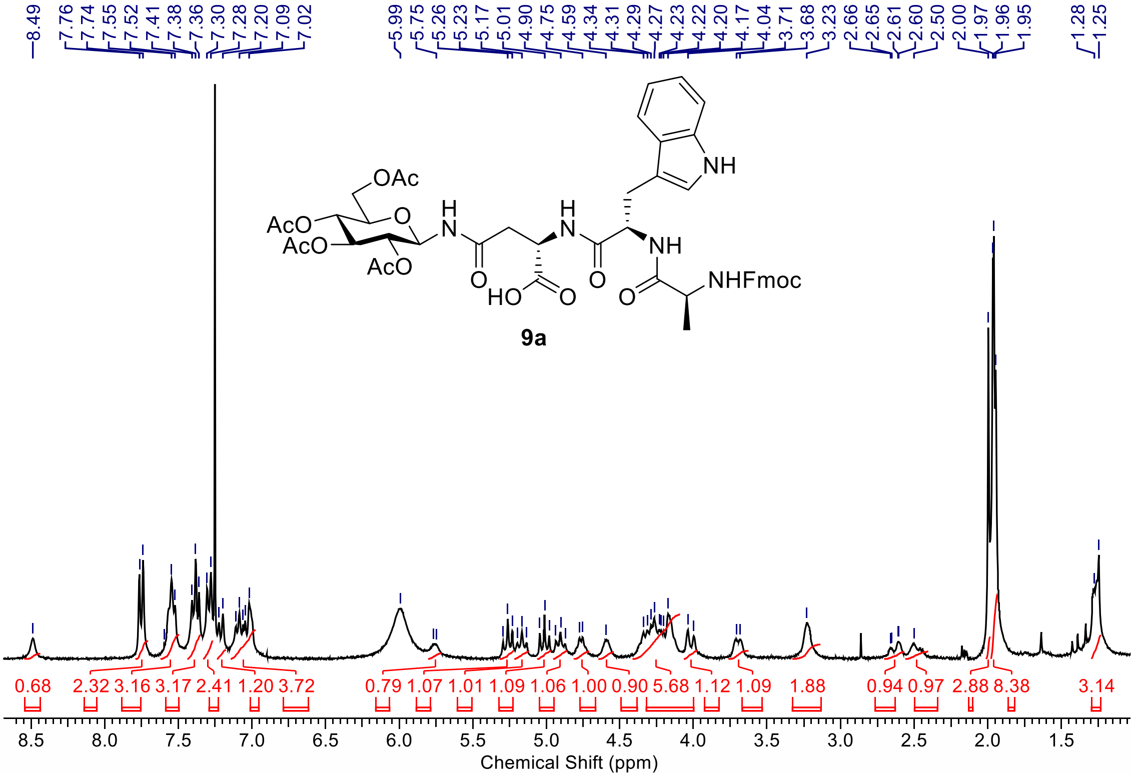

8.49 7.76 7.74 7.55 7.52 7.41 7.38 7.36 7.30 7.28 7.20 7.09 7.02 5.99 5.75 5.26 5.23 5.17 5.01 4.90 4.75 4.59 4.34 4.31 4.29 4.27 4.23 4.22 4.20 4.17 4.04 3.71 3.68 3.23 2.66 2.65 2.61 2.60 2.50 2.00 1.97 1.96 1.95 1.28 1.25
OAc
AcO
AcO
AcO
O
H
N
NH
HO
NHFmoc
9a
0.68 2.32 3.16 3.17 2.41 1.20 3.72 0.79 1.07 1.01 1.09 1.06 1.00 0.90 5.68 1.12 1.09 1.88 0.94 0.97 2.88 8.38 3.14
Chemical Shift (ppm)

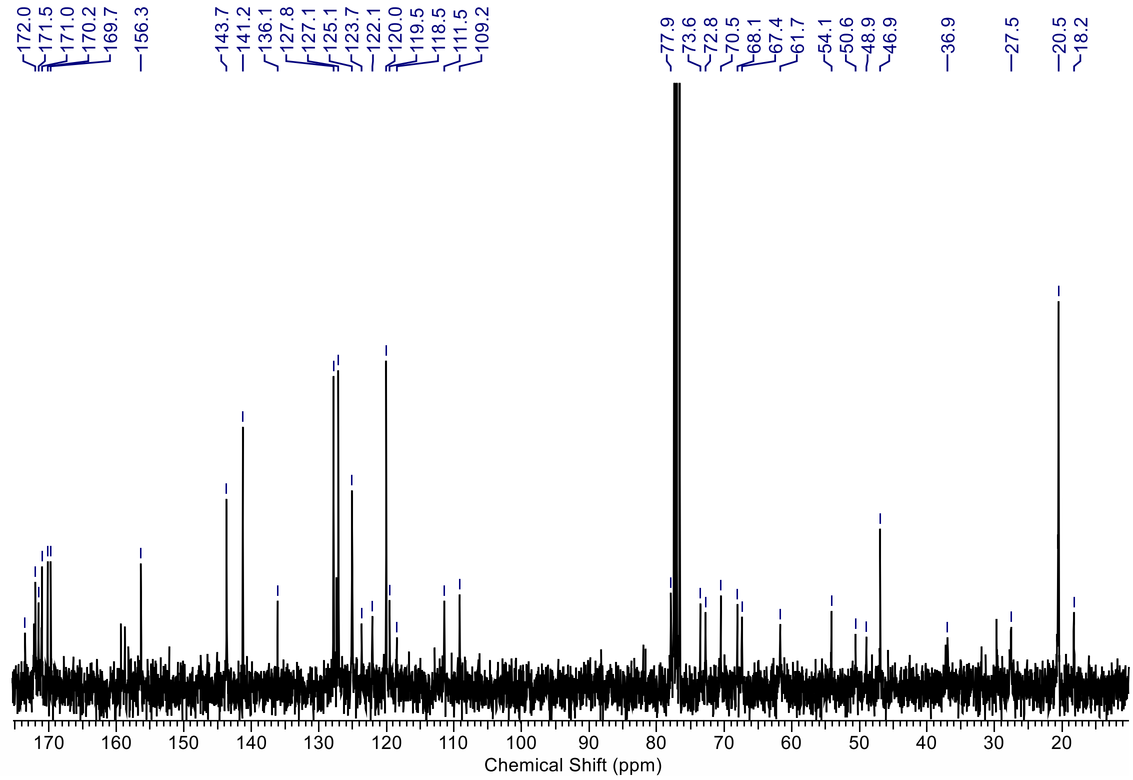

172.0 171.5 171.0 170.2 169.7 156.3 143.7 141.2 136.1 127.8 127.1 125.1 123.7 122.1 120.0 119.5 118.5 111.5 109.2 77.9 73.6 72.8 70.5 68.1 67.4 61.7 54.1 50.6 48.9 46.9 36.9 27.5 20.5 18.2
Chemical Shift (ppm)

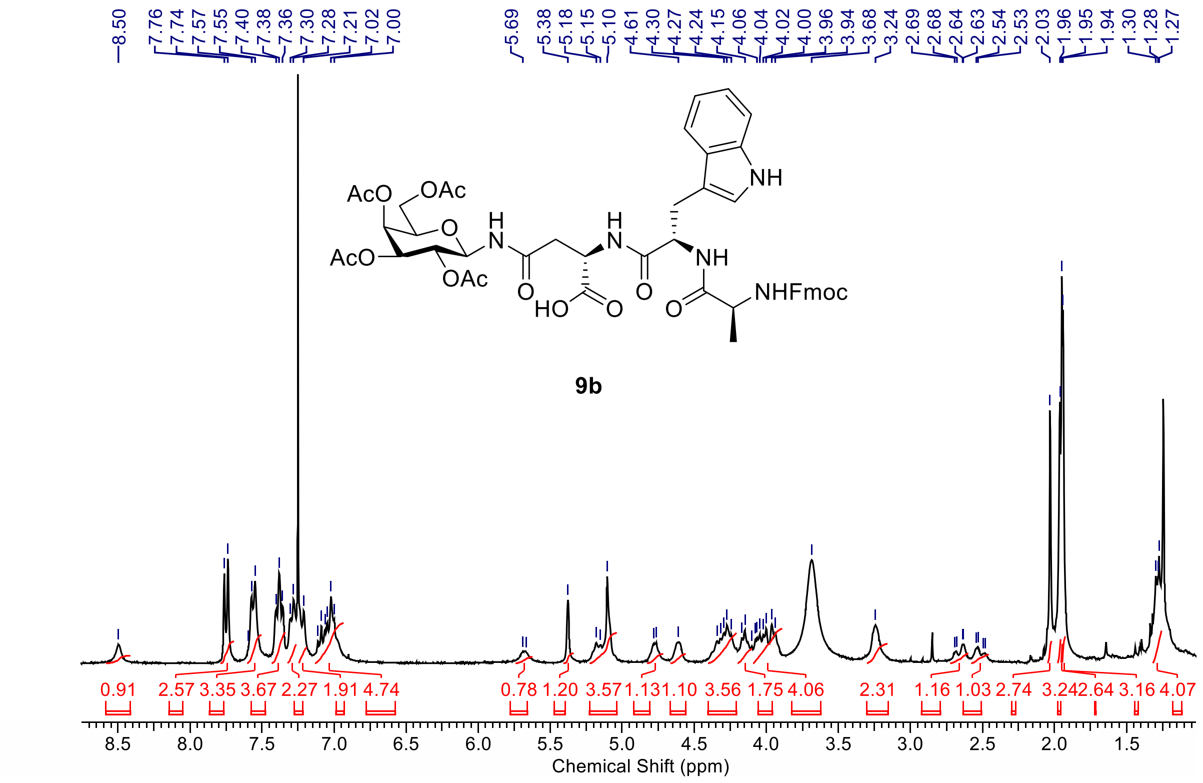
8.50
7.76
7.74
7.57
7.55
7.40
7.38
7.36
7.30
7.28
7.21
7.02
7.00
5.69
5.38
5.18
5.15
5.10
4.61
4.30
4.27
4.24
4.15
4.06
4.04
4.02
4.00
3.96
3.94
3.68
3.24
2.69
2.68
2.64
2.63
2.54
2.53
2.03
1.96
1.95
1.94
1.30
1.28
1.27
AcO
OAc
AcO
OAc
NH
NHFmoc
HO
9b
Chemical Shift (ppm)

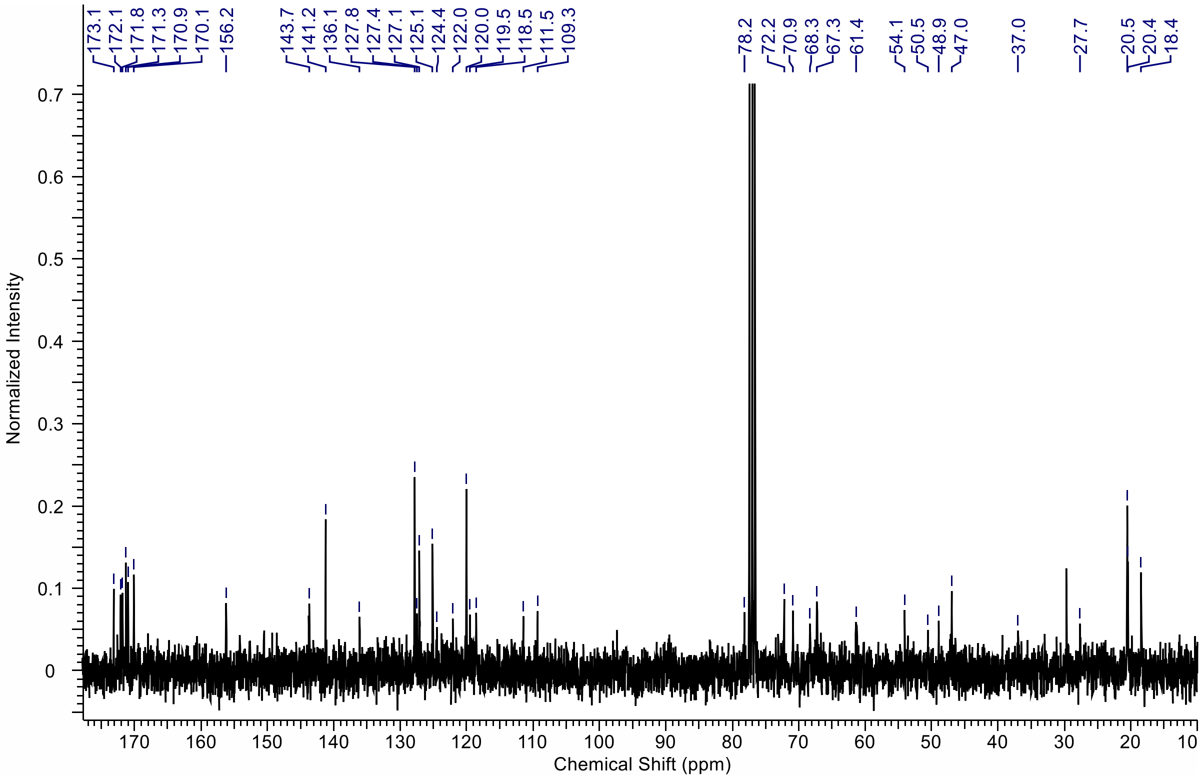
173.1
172.1
171.8
171.3
170.9
170.1
156.2
143.7
141.2
136.1
127.8
127.4
127.1
125.1
124.4
122.0
120.0
119.5
118.5
111.5
109.3
78.2
72.2
70.9
68.3
67.3
61.4
54.1
50.5
48.9
47.0
37.0
27.7
20.5
20.4
18.4
Normalized Intensity
Chemical Shift (ppm)

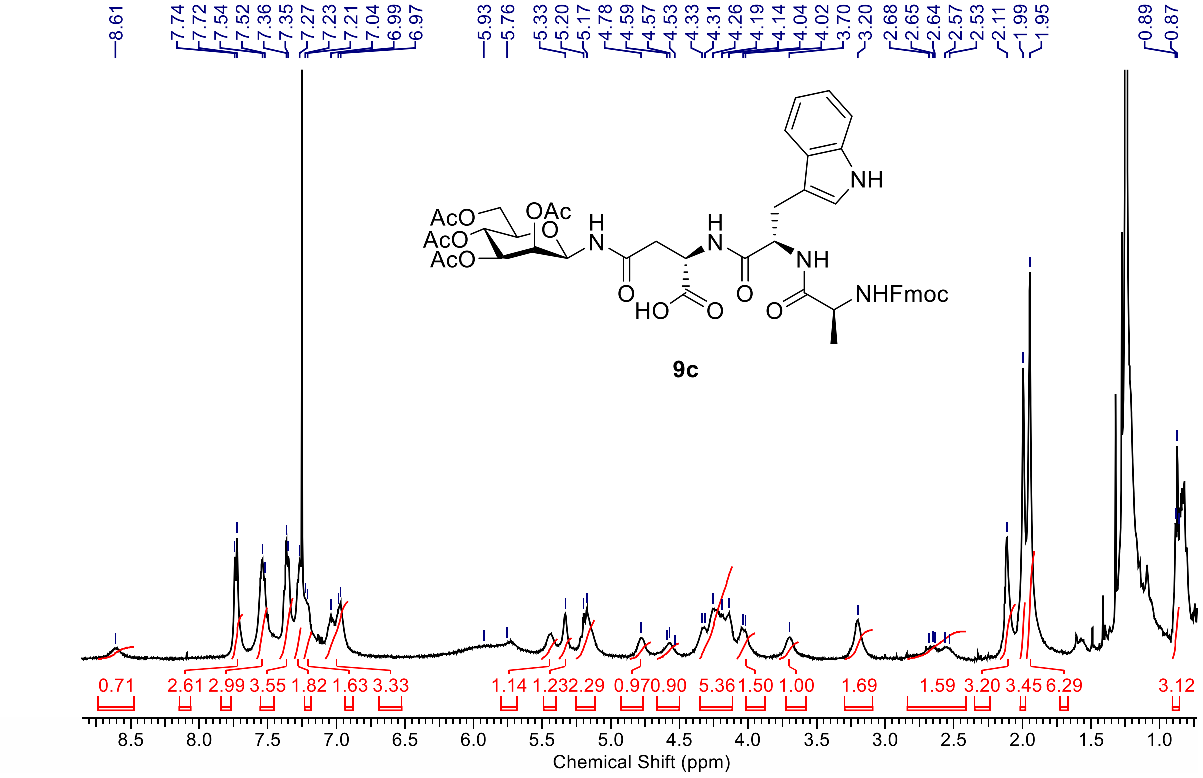
8.61
7.74 7.72 7.54 7.52 7.36 7.35 7.27 7.23 7.21 7.04 6.99 6.97
5.93 5.76
5.33 5.20 5.17 4.78 4.59 4.57 4.53 4.33 4.31 4.26 4.19 4.14 4.04 4.02 3.70 3.20 2.68 2.65 2.64 2.57 2.53 2.11 1.99 1.95
0.89 0.87
AcO
AcO
AcO
OAc
O
H
N
O
HO
O
H
N
O
NH
NH
O
NHFmoc
9c
0.71 2.61 2.99 3.55 1.82 1.63 3.33 1.14 1.23 2.29 0.97 0.90 5.36 1.50 1.00 1.69 1.59 3.20 3.45 6.29 3.12
8.5 8.0 7.5 7.0 6.5 6.0 5.5 5.0 4.5 4.0 3.5 3.0 2.5 2.0 1.5 1.0
Chemical Shift (ppm)

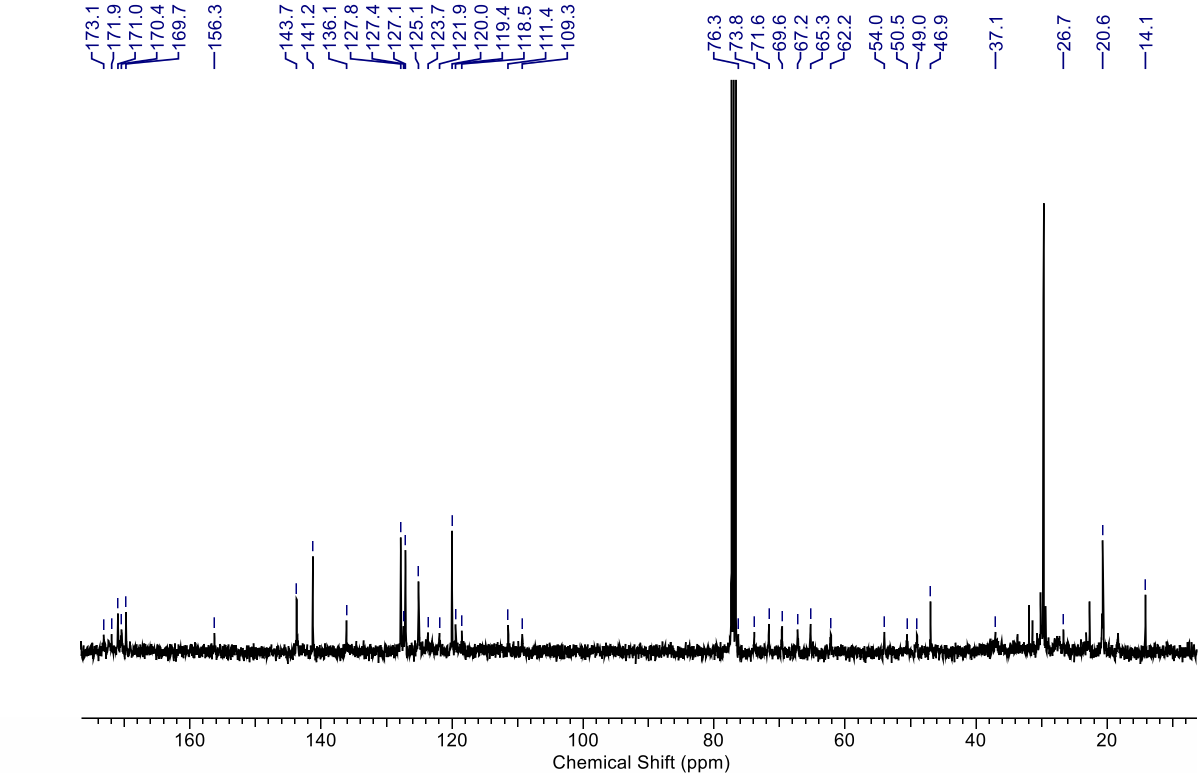
173.1 171.9 171.0 170.4 169.7 156.3
143.7 141.2 136.1 127.8 127.4 127.1 125.1 123.7 121.9 120.0 119.4 118.5 111.4 109.3
76.3 73.8 71.6 69.6 67.2 65.3 62.2 54.0 50.5 49.0 46.9 37.1 26.7 20.6 14.1
160 140 120 100 80 60 40 20
Chemical Shift (ppm)

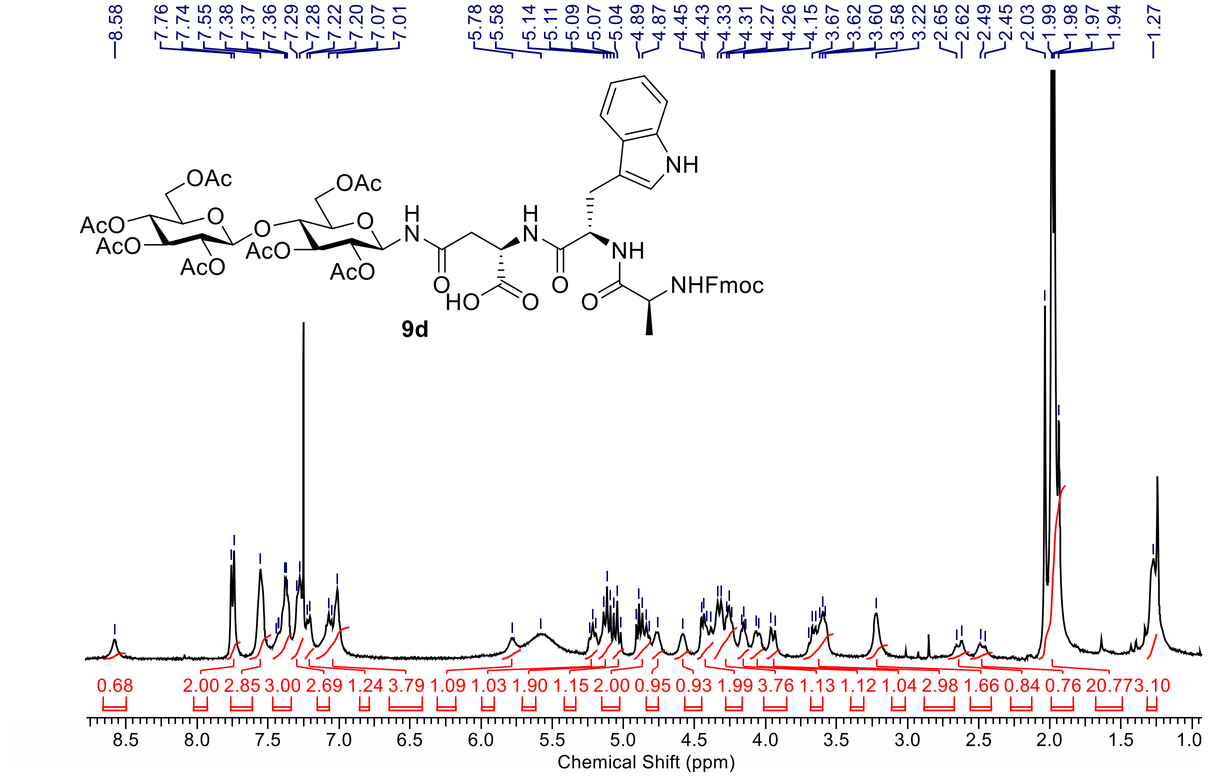
8.58
7.76 7.74 7.55 7.38 7.37 7.36 7.29 7.28 7.22 7.20 7.07 7.01
5.78 5.58 5.14 5.11 5.09 5.07 5.04 4.89 4.87 4.45 4.43 4.33 4.31 4.27 4.26 4.15 3.67 3.62 3.60 3.58 3.22 2.65 2.62 2.49 2.45 2.03 1.99 1.98 1.97 1.94
1.27
OAc
AcO
O
AcO
AcO
AcO
NH
NHFmoc
HO
9d
0.68 2.00 2.85 3.00 2.69 1.24 3.79 1.09 1.03 1.90 1.15 2.00 0.95 0.93 1.99 3.76 1.13 1.12 1.04 2.98 1.66 0.84 0.76 20.77 3.10
Chemical Shift (ppm)

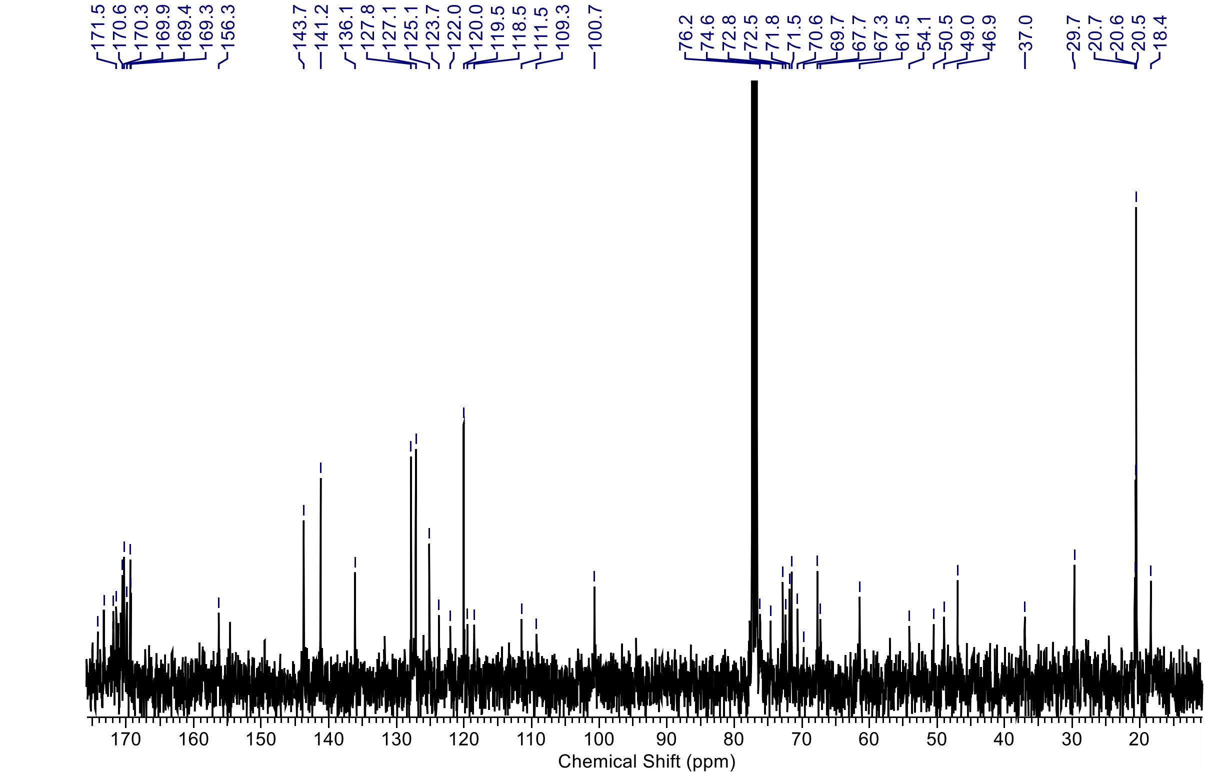
171.5 170.6 170.3 169.9 169.4 169.3 156.3
143.7 141.2 136.1 127.8 127.1 125.1 123.7 122.0 120.0 119.5 118.5 111.5 109.3
100.7
76.2 74.6 72.8 72.5 71.8 71.5 70.6 69.7 67.7 67.3 61.5 54.1 50.5 49.0 46.9
37.0
29.7 20.7 20.6 20.5 18.4
Chemical Shift (ppm)

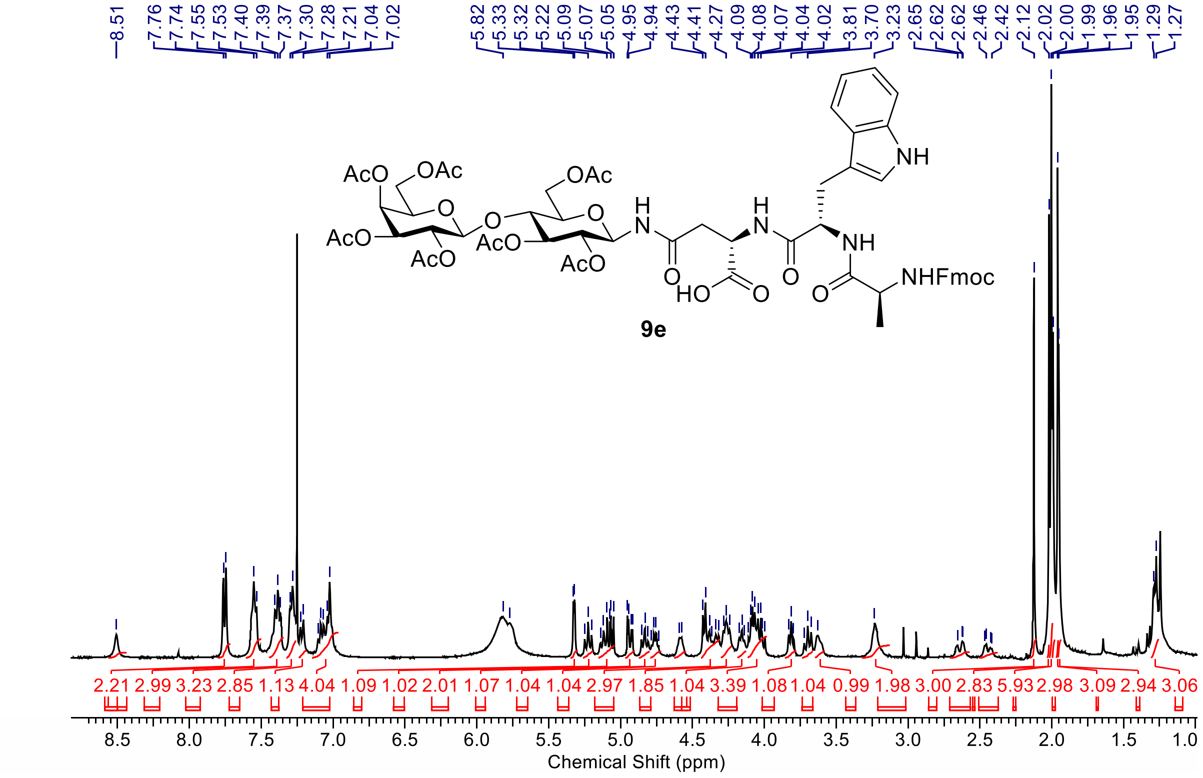

AcO
OAc
O
AcO
AcO
O
OAc
O
AcO
AcO
H
N
O
H
N
HO
O
O
NH
NH
O
NHFmoc
9e
Chemical Shift (ppm)

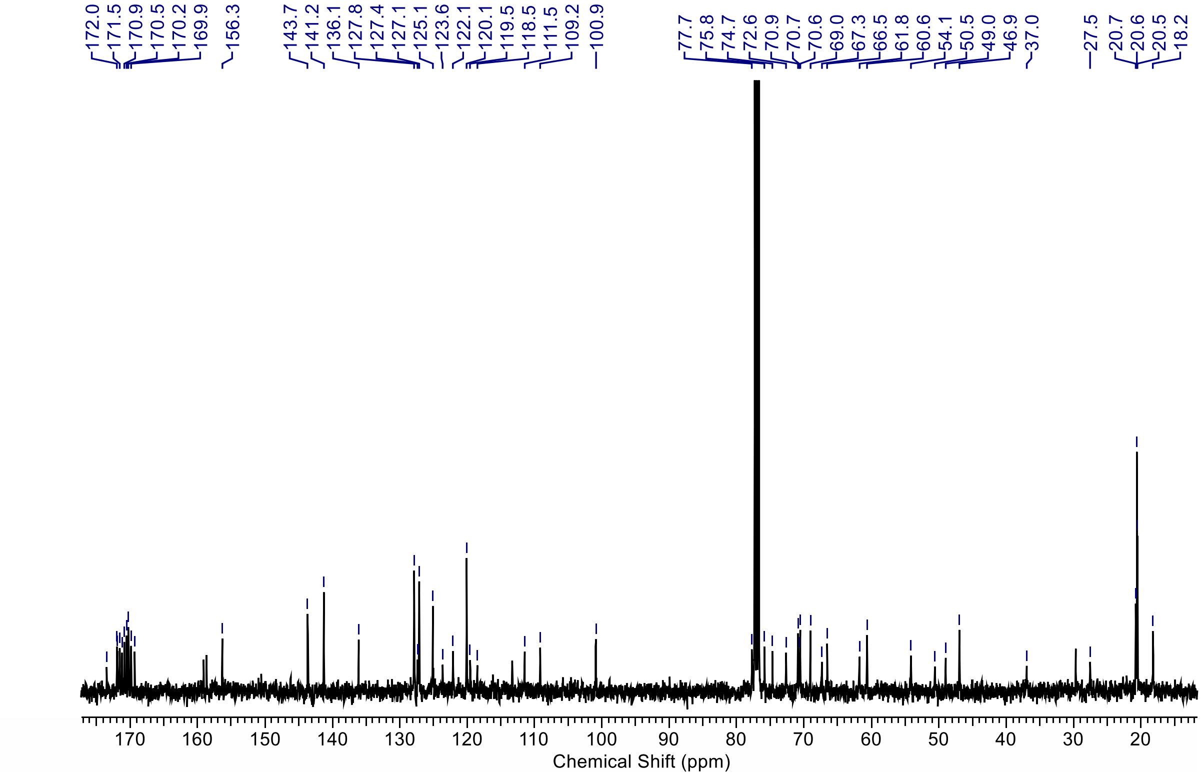

Chemical Shift (ppm)

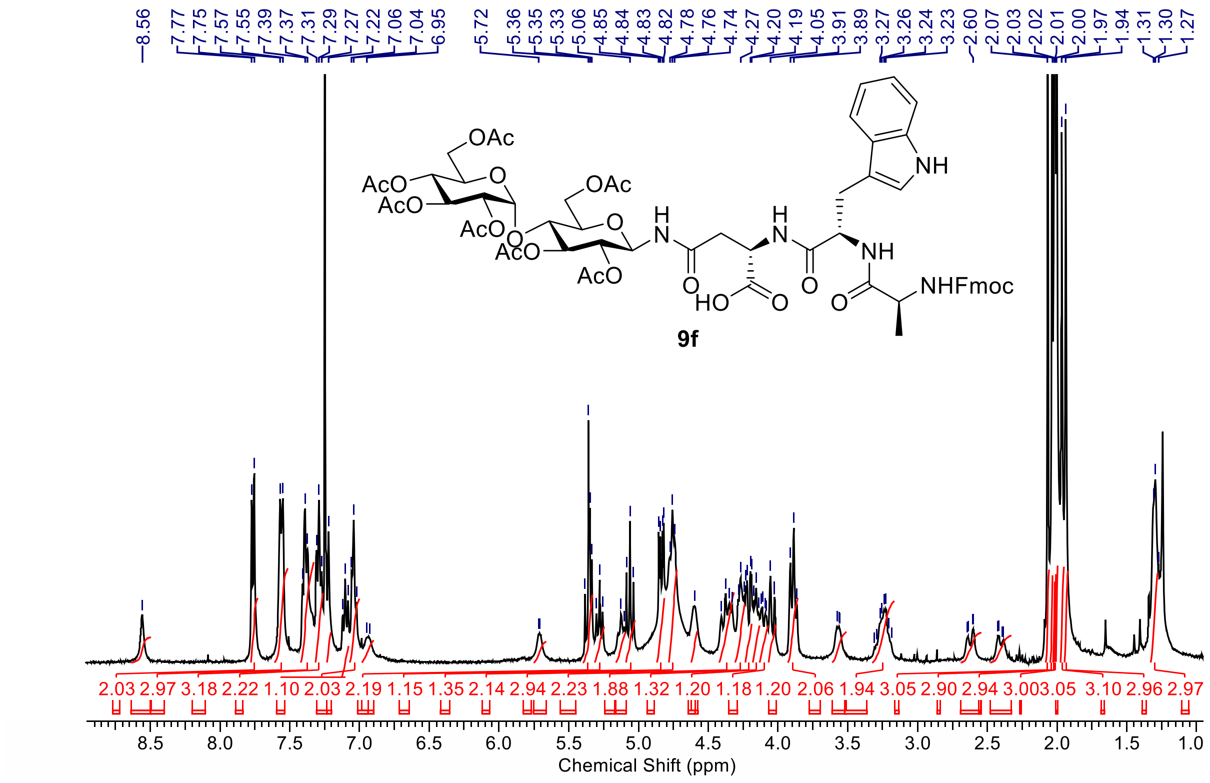

AcO
AcO
OAc
AcO
AcO
OAc
AcO
AcO
N
H
N
H
NH
NH
NHFmoc
HO
9f
Chemical Shift (ppm)

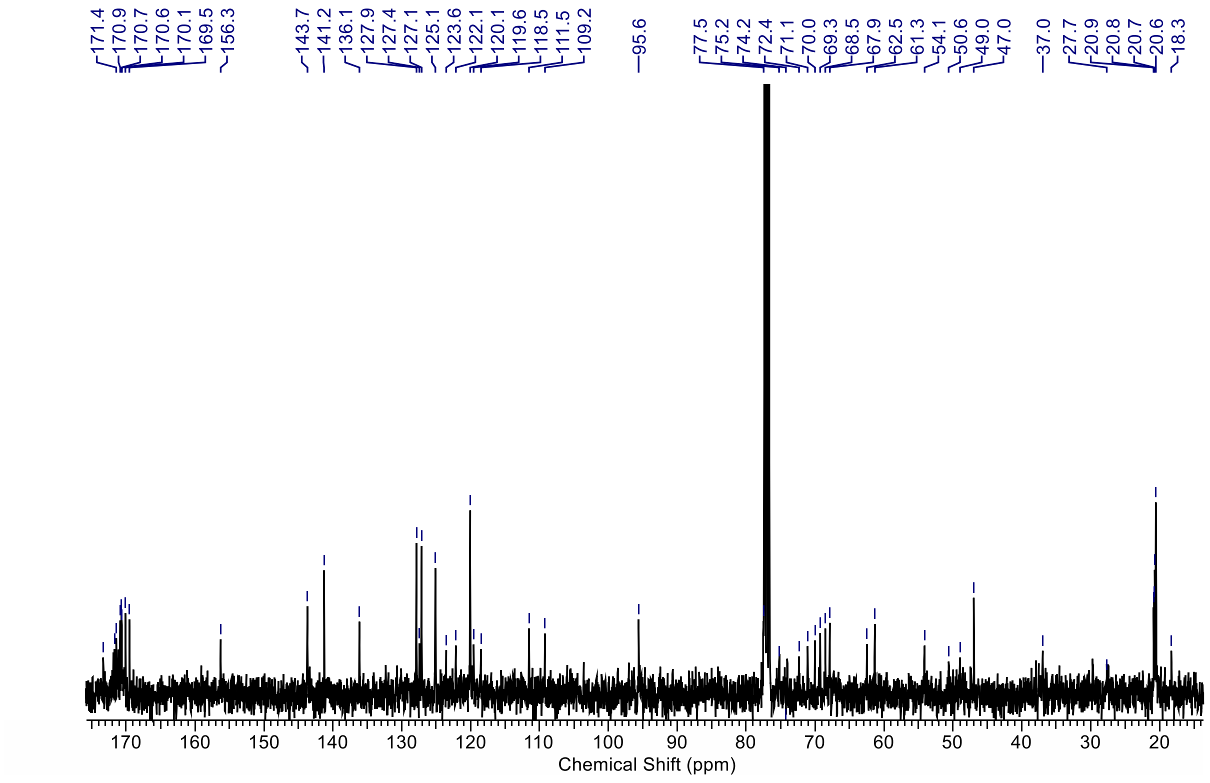

Chemical Shift (ppm)

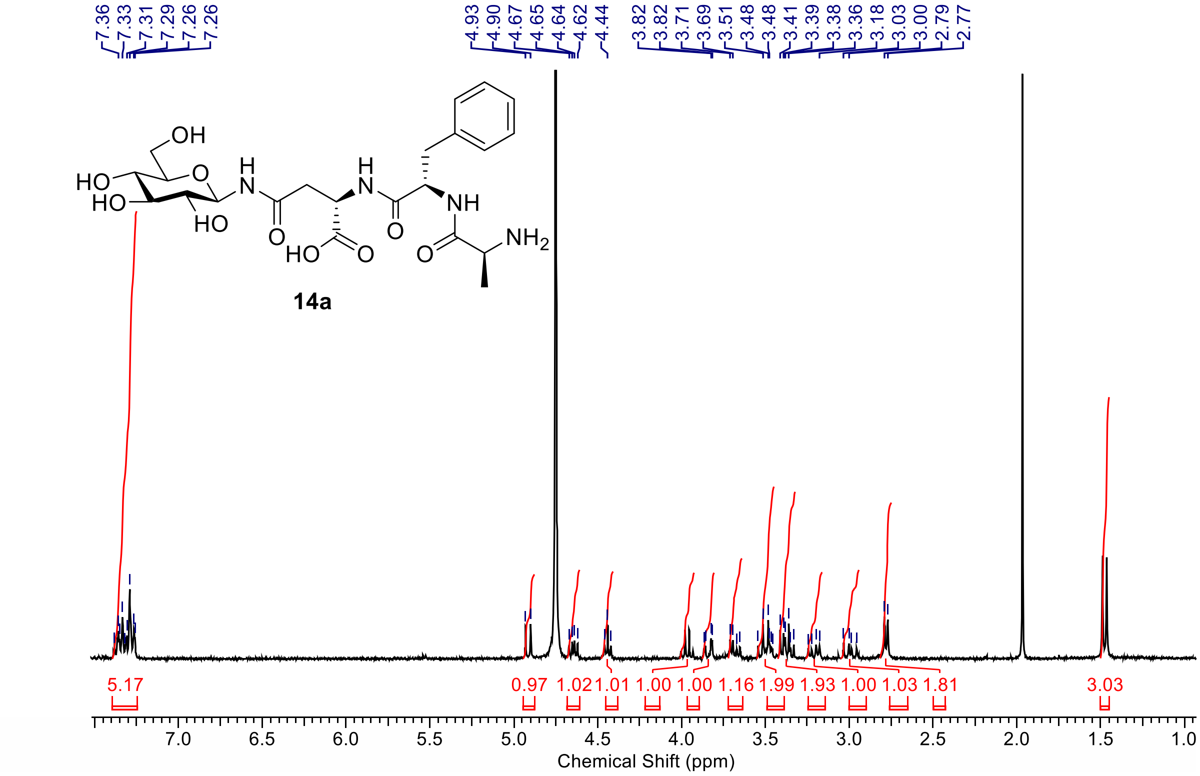
7.36
7.33
7.31
7.29
7.26
7.26
4.93
4.90
4.67
4.65
4.64
4.62
4.44
3.82
3.82
3.71
3.69
3.51
3.48
3.48
3.41
3.39
3.38
3.36
3.18
3.03
3.00
2.79
2.77
14a
5.17
0.97
1.02
1.01
1.00
1.00
1.16
1.99
1.93
1.00
1.03
1.81
3.03
Chemical Shift (ppm)

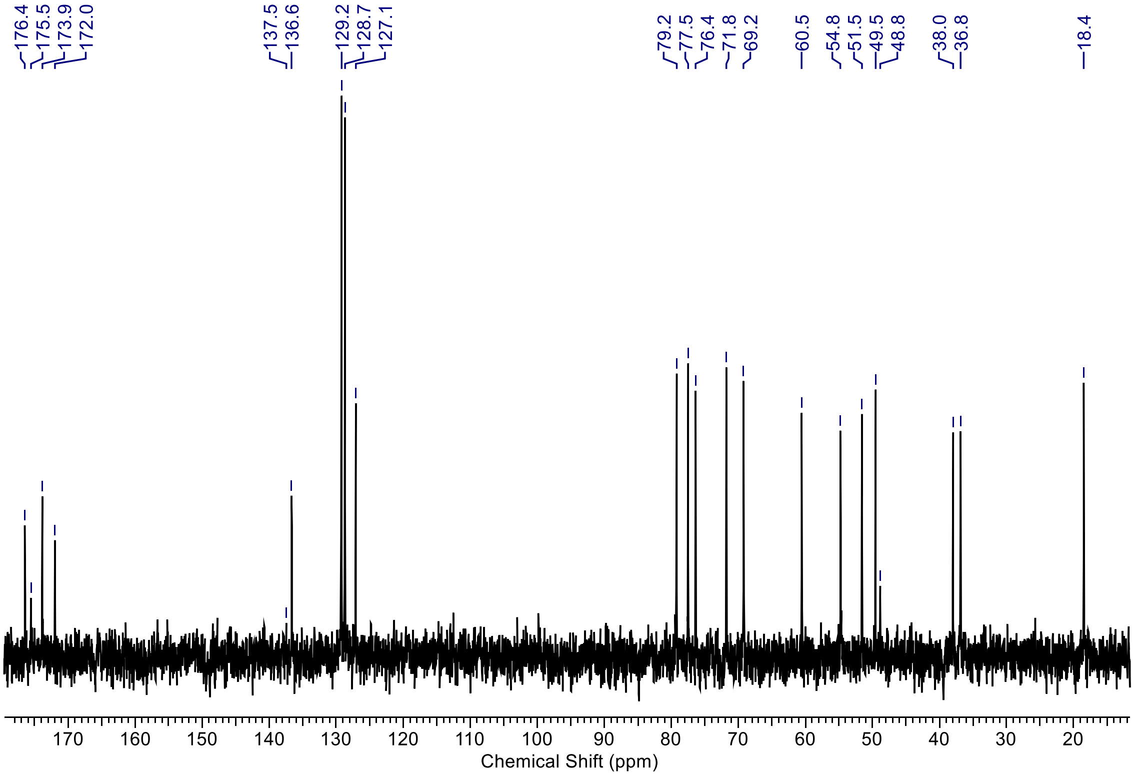
176.4
175.5
173.9
172.0
137.5
136.6
129.2
128.7
127.1
79.2
77.5
76.4
71.8
69.2
60.5
54.8
51.5
49.5
48.8
38.0
36.8
18.4
Chemical Shift (ppm)

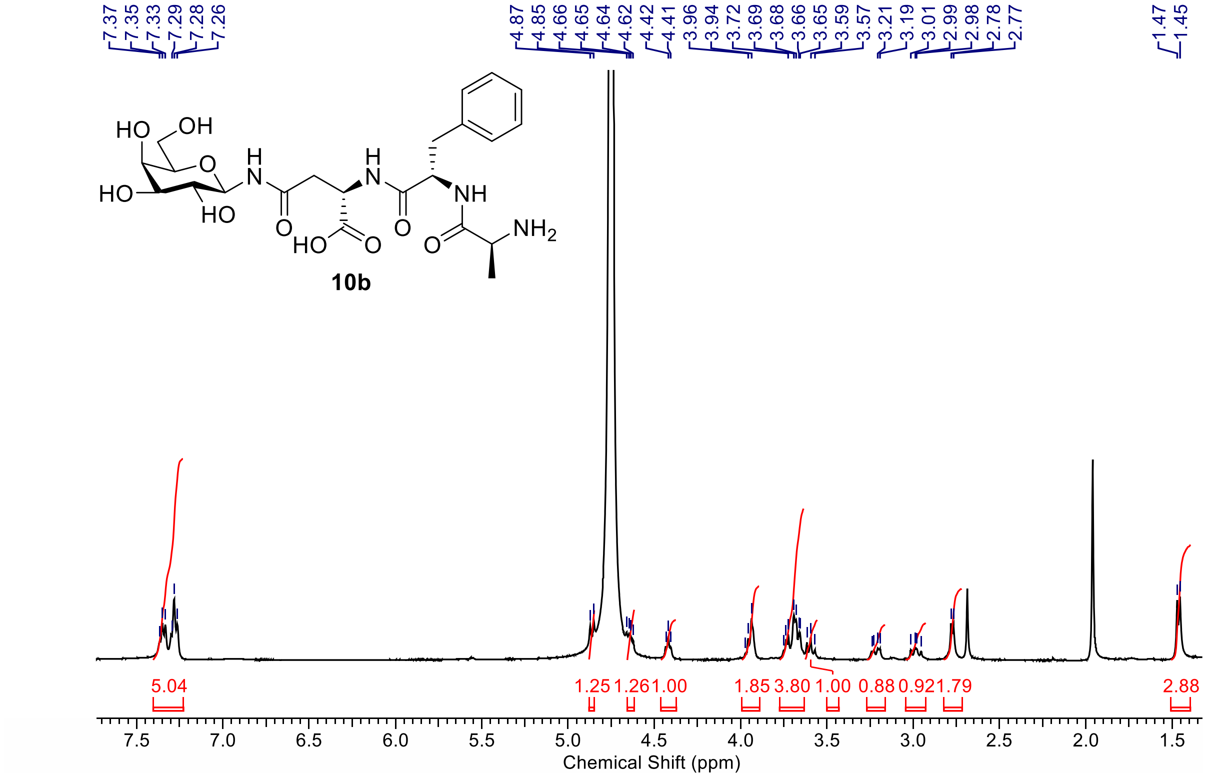
7.37
7.35
7.33
7.29
7.28
7.26
4.87
4.85
4.66
4.65
4.64
4.62
4.42
4.41
3.96
3.94
3.72
3.69
3.68
3.66
3.65
3.59
3.57
3.21
3.19
3.01
2.99
2.98
2.78
2.77
1.47
1.45
10b
5.04
1.25
1.26
1.00
1.85
3.80
1.00
0.88
0.92
1.79
2.88
Chemical Shift (ppm)

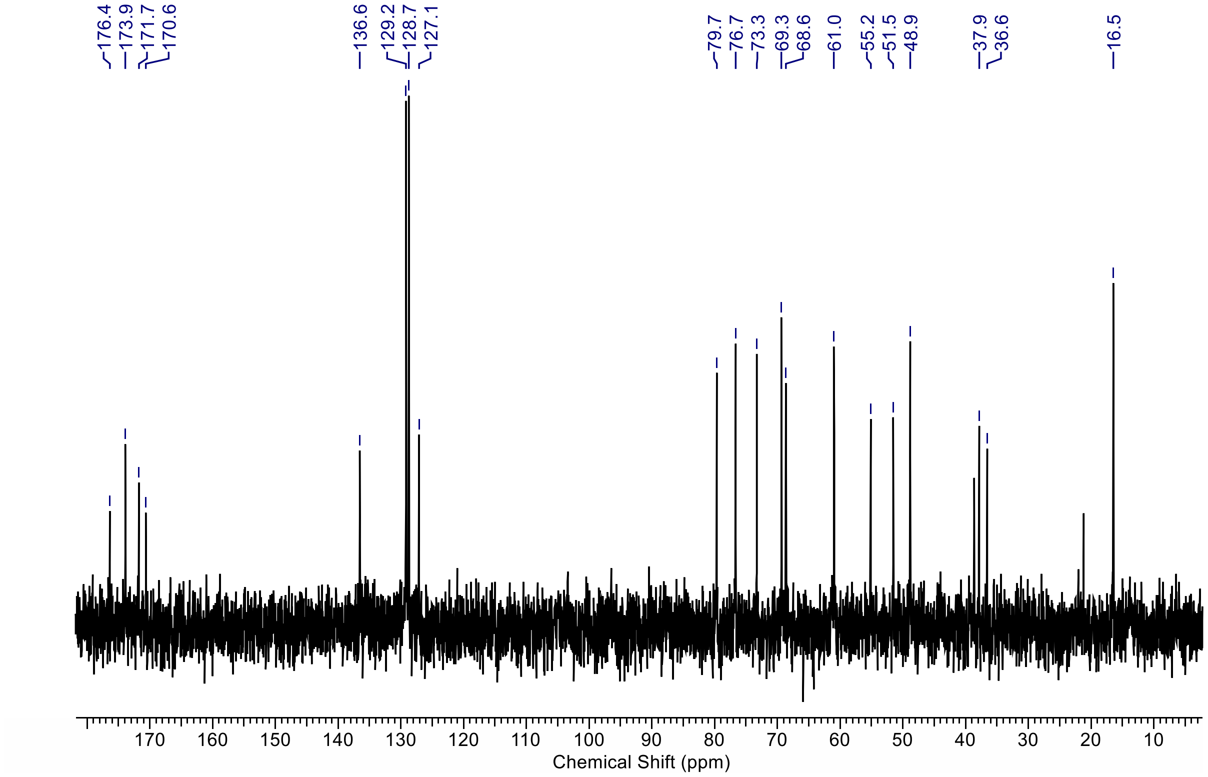
176.4
173.9
171.7
170.6
136.6
129.2
128.7
127.1
79.7
76.7
73.3
69.3
68.6
61.0
55.2
51.5
48.9
37.9
36.6
16.5
Chemical Shift (ppm)

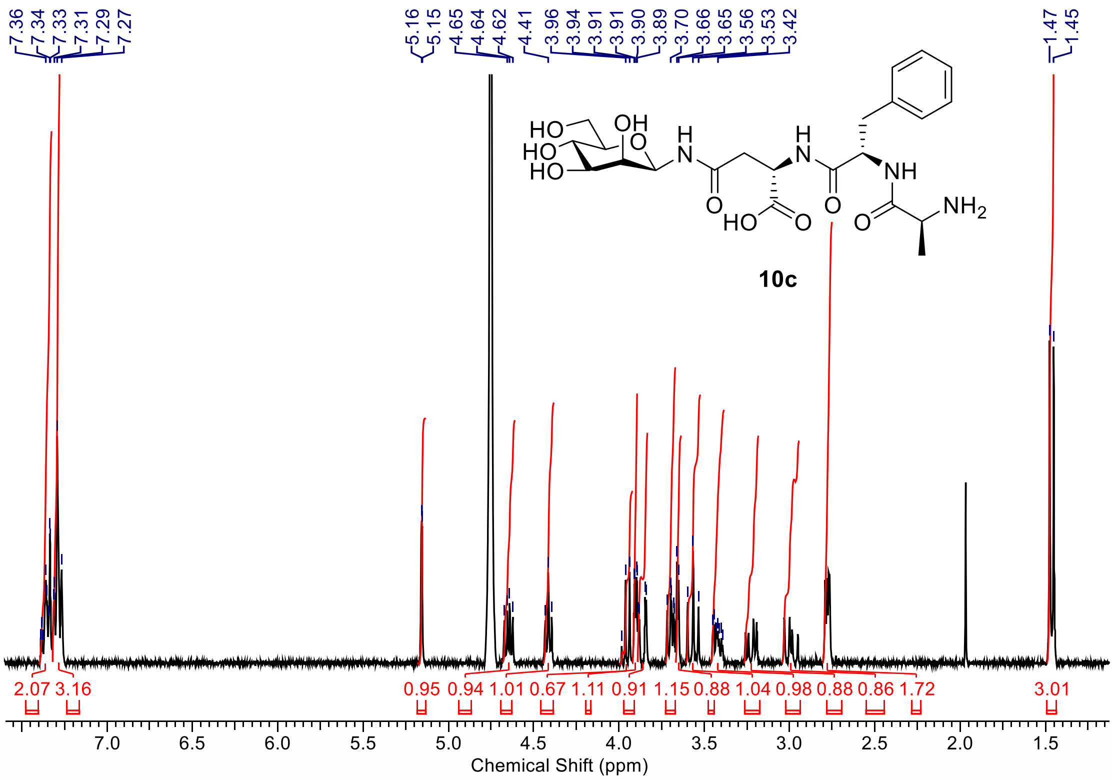
7.36 7.34 7.33 7.31 7.29 7.27
5.16 5.15 4.65 4.64 4.62 4.41 3.96 3.94 3.91 3.91 3.90 3.89 3.70 3.66 3.65 3.56 3.53 3.42
1.47 1.45
HO
OH
HO
O
HO
H
N
O
H
N
HO
O
O
NH
O
NH2
10c
2.07 3.16
0.95 0.94 1.01 0.67 1.11 0.91 1.15 0.88 1.04 0.98 0.88 0.86 1.72
3.01
7.0 6.5 6.0 5.5 5.0 4.5 4.0 3.5 3.0 2.5 2.0 1.5
Chemical Shift (ppm)

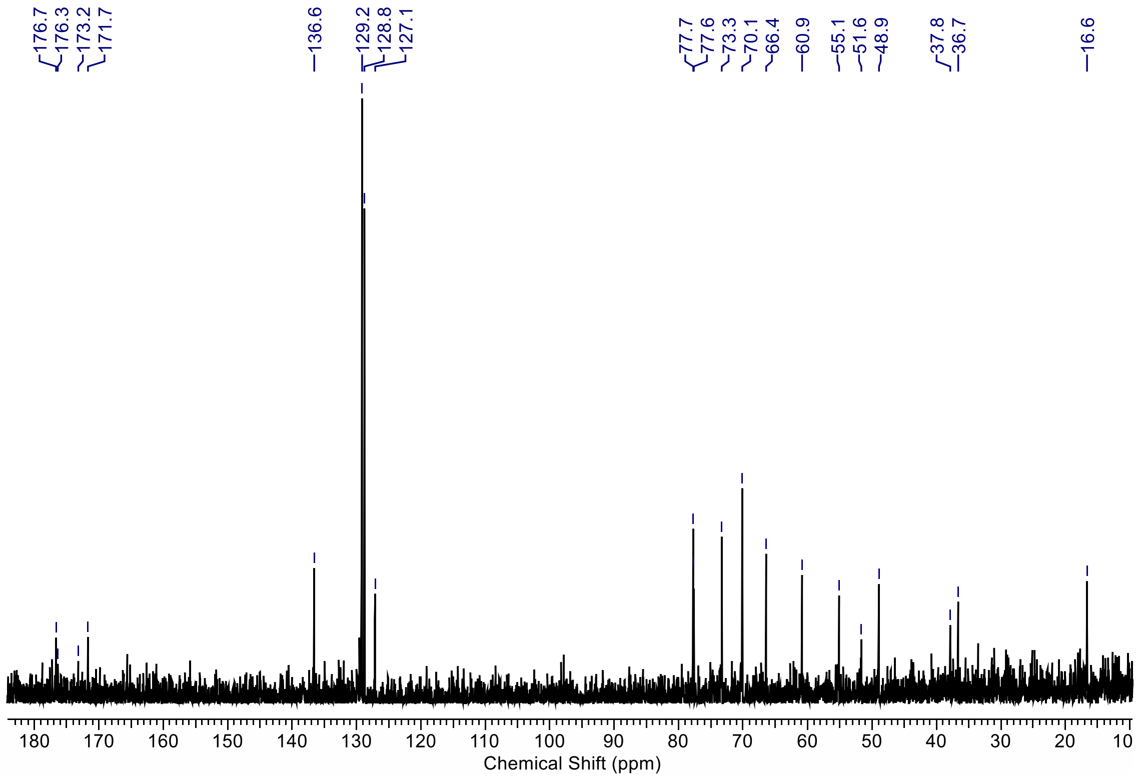
176.7 176.3 173.2 171.7
136.6
129.2 128.8 127.1
77.7 77.6 73.3 70.1 66.4 60.9 55.1 51.6 48.9 37.8 36.7
16.6
180 170 160 150 140 130 120 110 100 90 80 70 60 50 40 30 20 10
Chemical Shift (ppm)

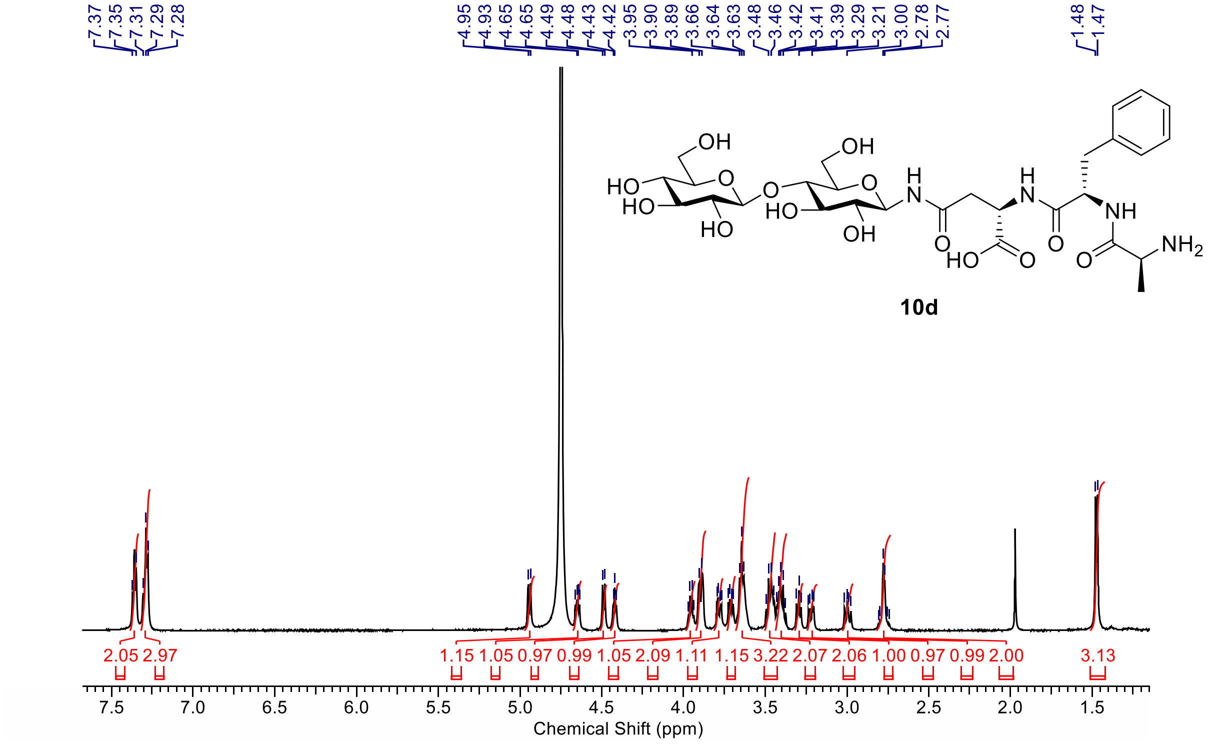
10d
Chemical Shift (ppm)

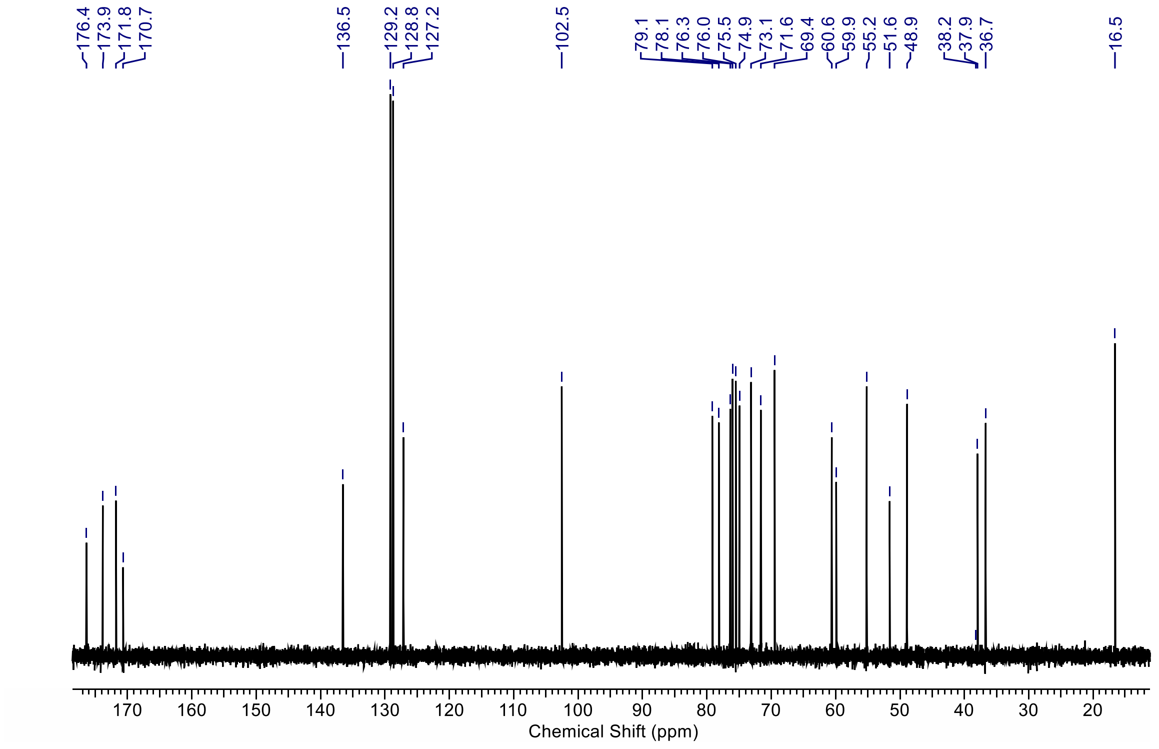
Chemical Shift (ppm)

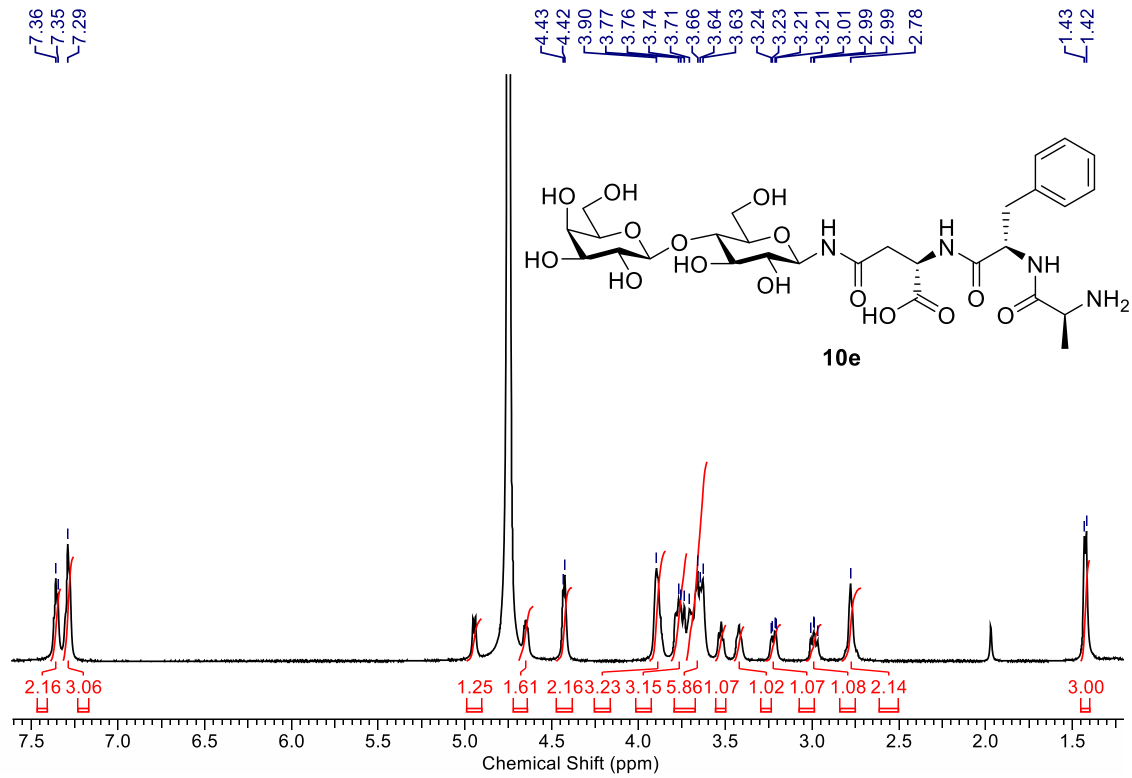
7.36
7.35
7.29
4.43
4.42
3.90
3.77
3.76
3.74
3.71
3.66
3.64
3.63
3.24
3.23
3.21
3.21
3.01
2.99
2.99
2.78
1.43
1.42
HO
OH
O
HO
HO
O
OH
O
HO
OH
H
N
O
H
N
O
HO
O
NH
O
NH2
10e
2.16
3.06
1.25
1.61
2.16
3.23
3.15
5.86
1.07
1.02
1.07
1.08
2.14
3.00
Chemical Shift (ppm)

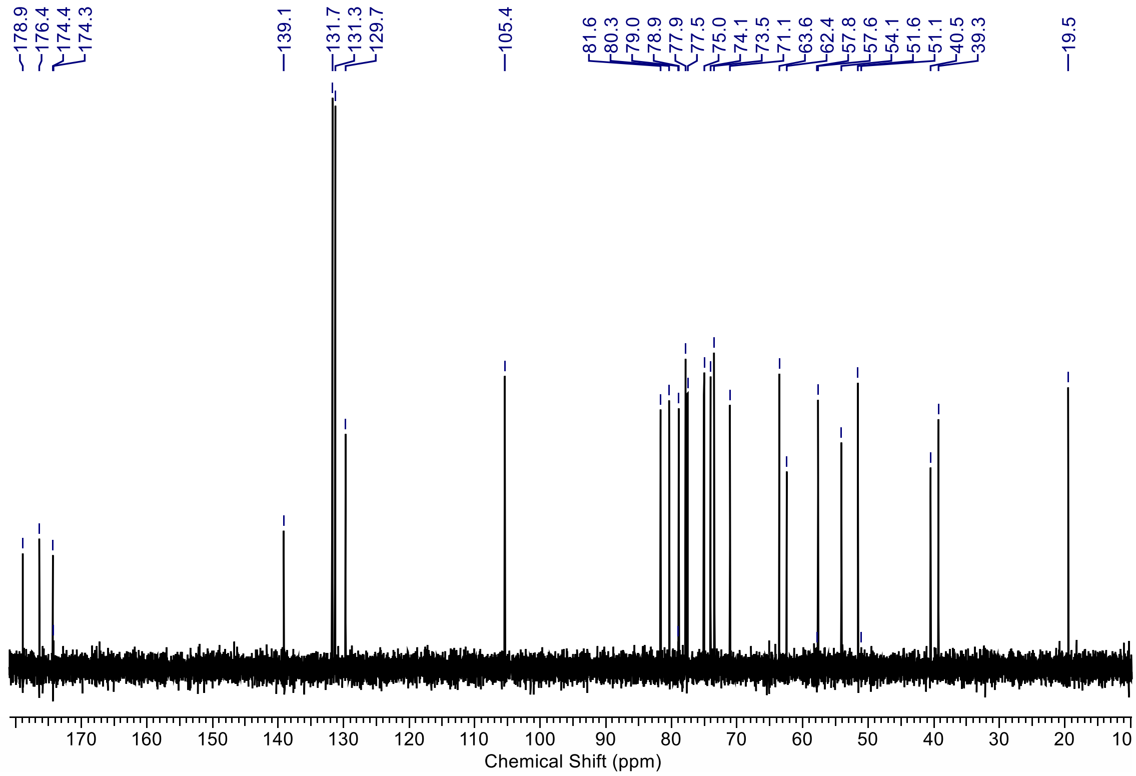
178.9
176.4
174.4
174.3
139.1
131.7
131.3
129.7
105.4
81.6
80.3
79.0
78.9
77.9
77.5
75.0
74.1
73.5
71.1
63.6
62.4
57.8
57.6
54.1
51.6
51.1
40.5
39.3
19.5
Chemical Shift (ppm)

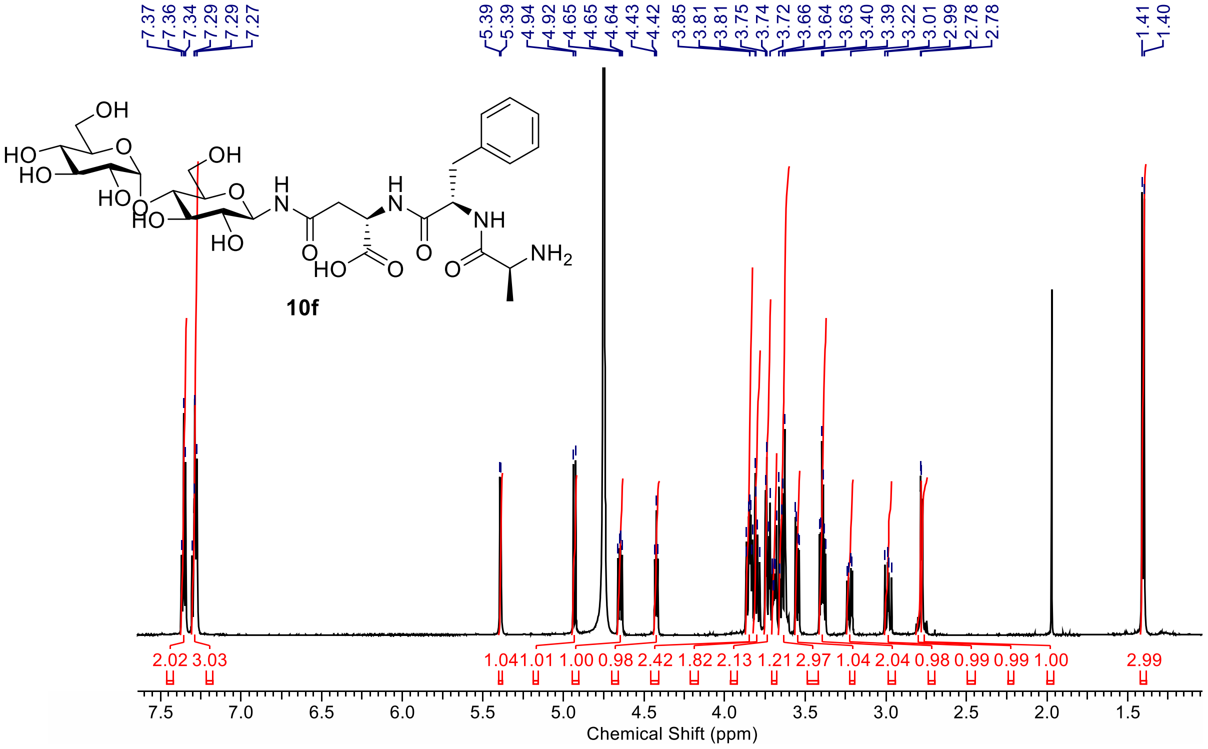
7.37 7.36 7.34 7.29 7.29 7.27
5.39 5.39 4.94 4.92 4.65 4.65 4.64 4.43 4.42
3.85 3.81 3.81 3.75 3.74 3.72 3.66 3.64 3.63 3.40 3.39 3.22 3.01 2.99 2.78 2.78
1.41 1.40
10f
2.02 3.03
1.04 1.01 1.00 0.98 2.42 1.82 2.13 1.21 2.97 1.04 2.04 0.98 0.99 0.99 1.00
2.99
7.5 7.0 6.5 6.0 5.5 5.0 4.5 4.0 3.5 3.0 2.5 2.0 1.5
Chemical Shift (ppm)

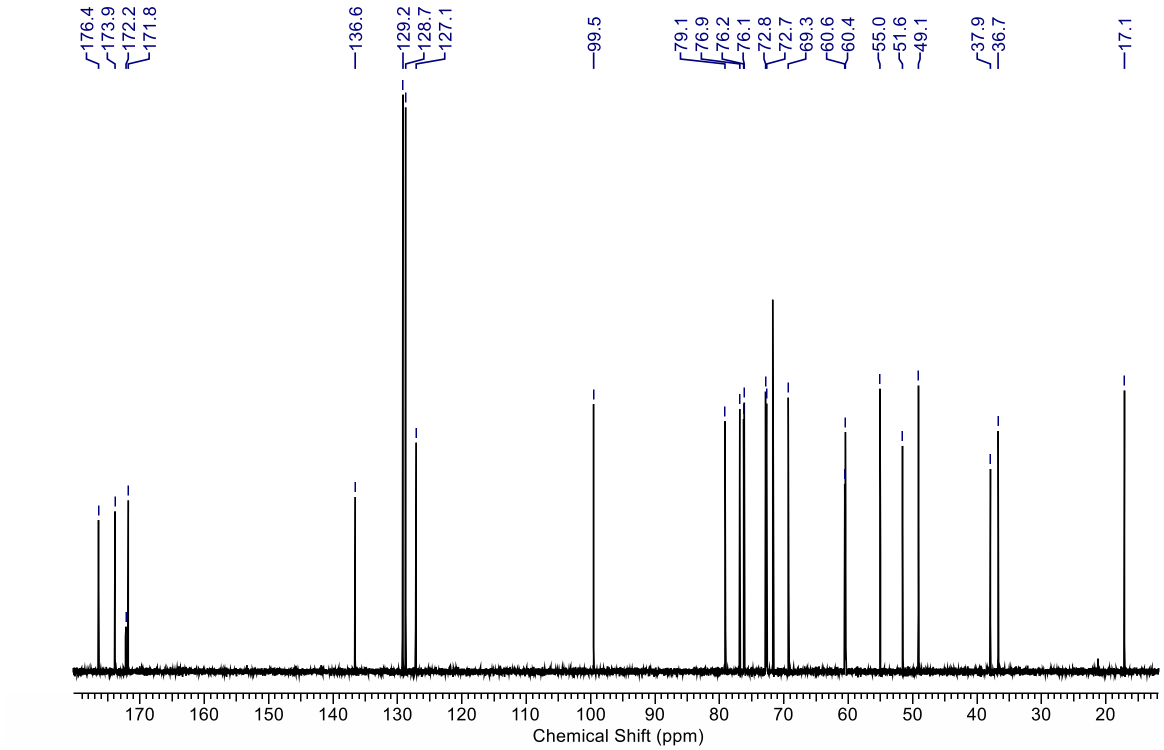
176.4 173.9 172.2 171.8
136.6
129.2 128.7 127.1
99.5
79.1 76.9 76.2 76.1 72.8 72.7 69.3 60.6 60.4 55.0 51.6 49.1 37.9 36.7
17.1
170 160 150 140 130 120 110 100 90 80 70 60 50 40 30 20
Chemical Shift (ppm)

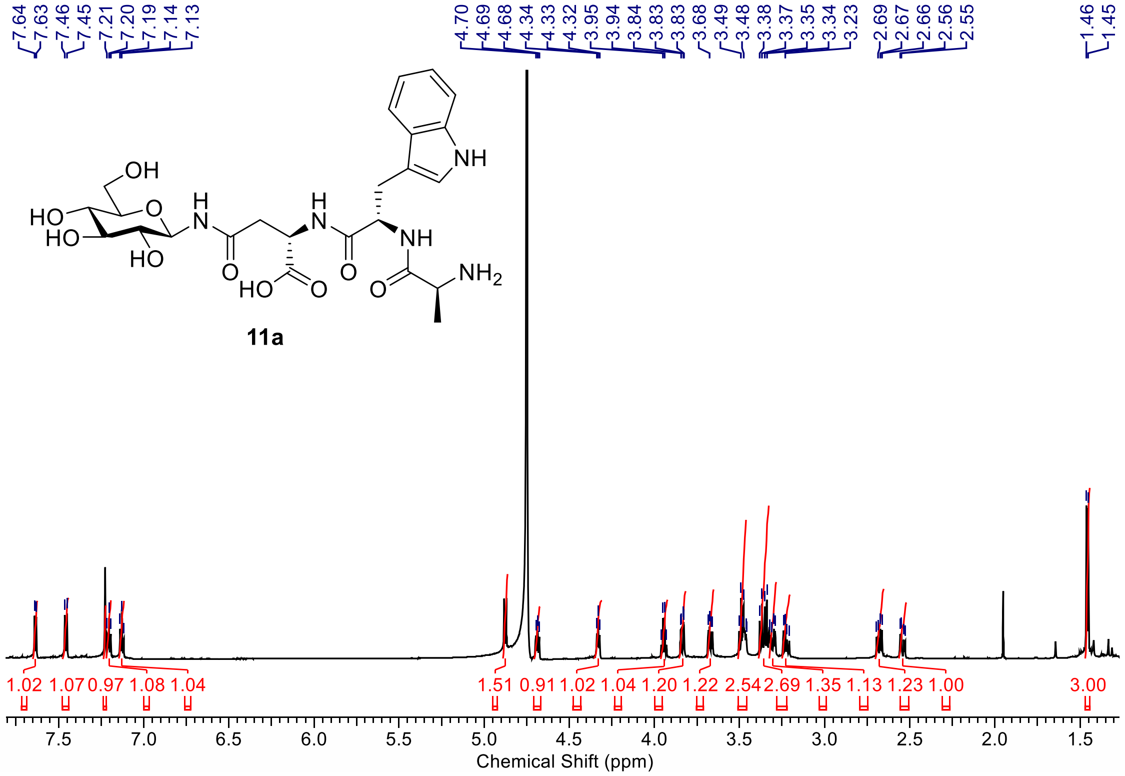
7.64 7.63 7.46 7.45 7.21 7.20 7.19 7.14 7.13
4.70 4.69 4.68 4.34 4.33 4.32 3.95 3.94 3.84 3.83 3.83 3.68 3.49 3.48 3.38 3.37 3.35 3.34 3.23 2.69 2.67 2.66 2.56 2.55
1.46 1.45
OH
HO
HO
HO
O
H
N
NH
NH
NH2
HO
O
11a
1.02 1.07 0.97 1.08 1.04
1.51 0.91 1.02 1.04 1.20 1.22 2.54 2.69 1.35 1.13 1.23 1.00
3.00
7.5 7.0 6.5 6.0 5.5 5.0 4.5 4.0 3.5 3.0 2.5 2.0 1.5
Chemical Shift (ppm)

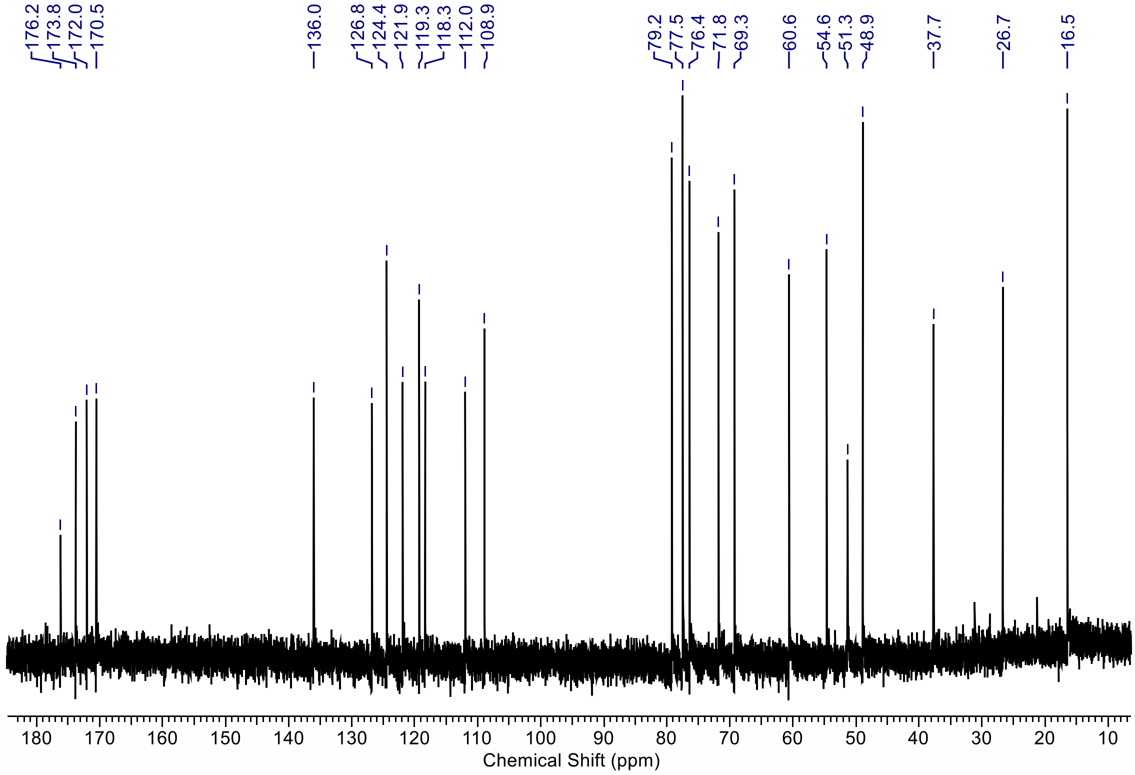
176.2 173.8 172.0 170.5
136.0
126.8 124.4 121.9 119.3 118.3 112.0 108.9
79.2 77.5 76.4 71.8 69.3
60.6 54.6 51.3 48.9
37.7
26.7
16.5
180 170 160 150 140 130 120 110 100 90 80 70 60 50 40 30 20 10
Chemical Shift (ppm)

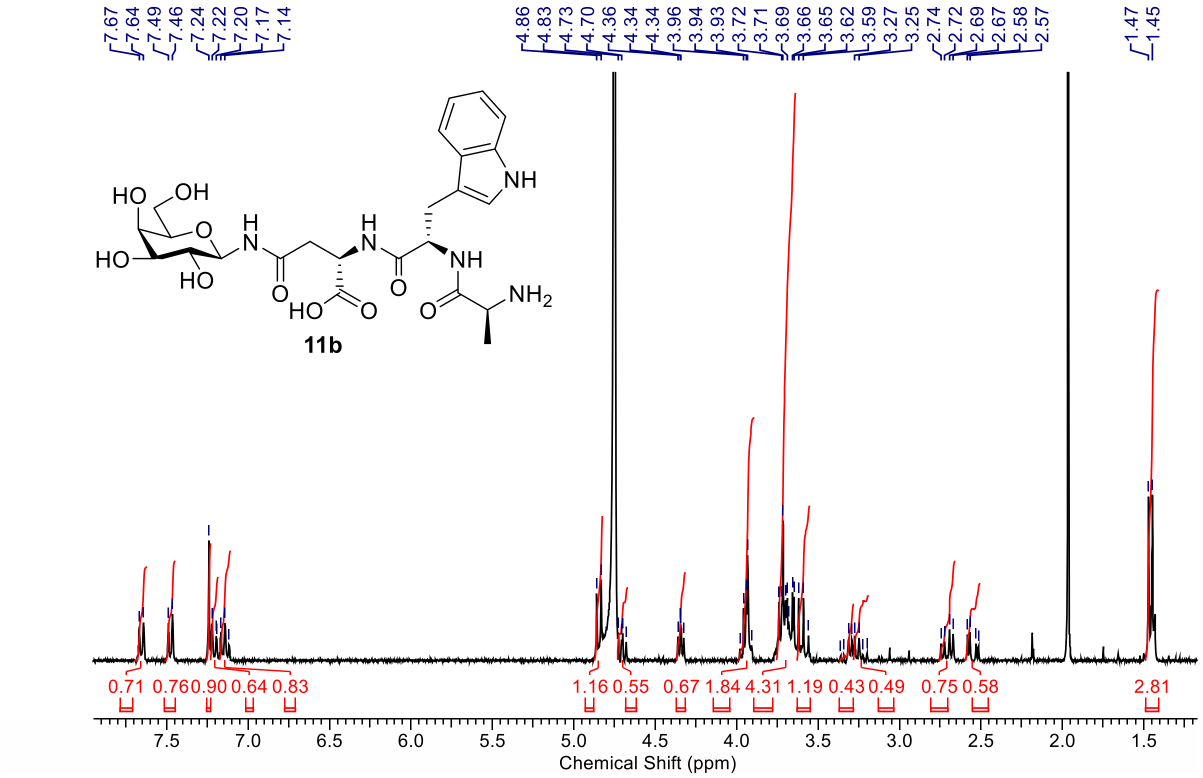
7.67 7.64 7.49 7.46 7.24 7.22 7.20 7.17 7.14
4.86 4.83 4.73 4.70 4.36 4.34 4.34 3.96 3.94 3.93 3.72 3.71 3.69 3.66 3.65 3.62 3.59 3.27 3.25 2.74 2.72 2.69 2.67 2.58 2.57
1.47 1.45
HO
OH
O
HO
HO
H
N
O
H
N
HO
O
O
NH
NH
O
NH2
11b
0.71 0.76 0.90 0.64 0.83 1.16 0.55 0.67 1.84 4.31 1.19 0.43 0.49 0.75 0.58 2.81
7.5 7.0 6.5 6.0 5.5 5.0 4.5 4.0 3.5 3.0 2.5 2.0 1.5
Chemical Shift (ppm)

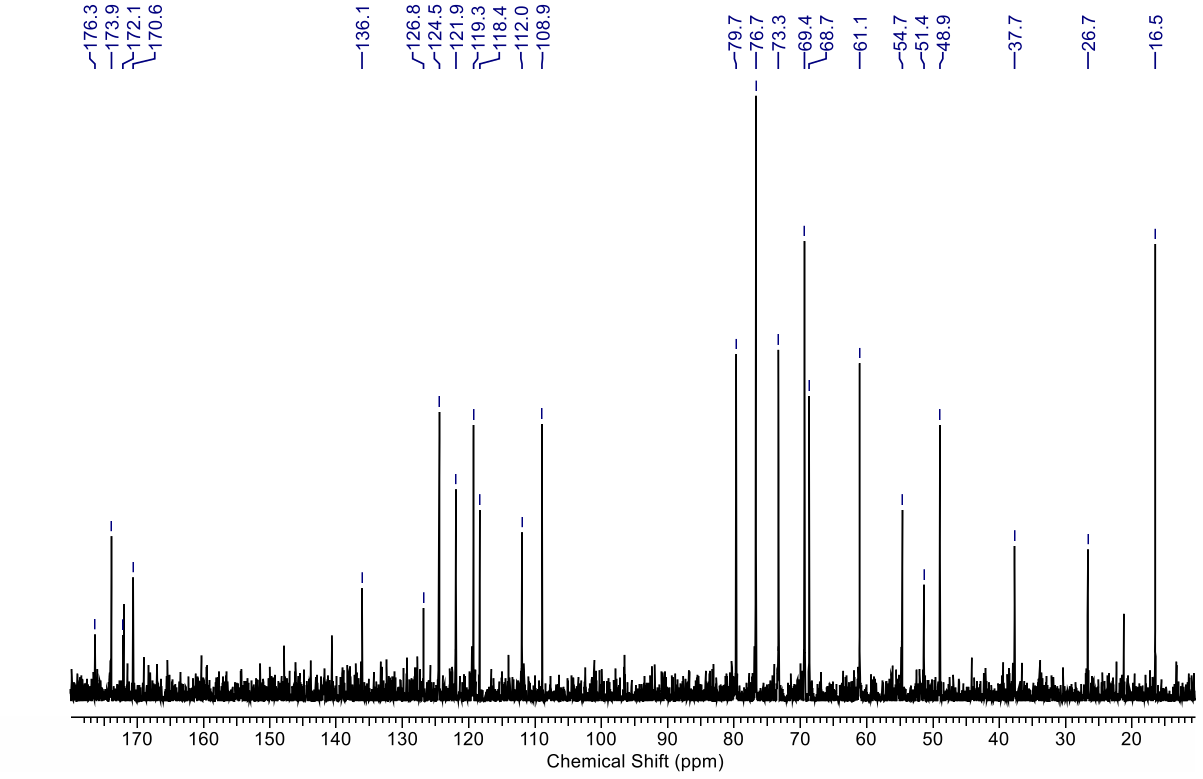
176.3 173.9 172.1 170.6
136.1
126.8 124.5 121.9 119.3 118.4 112.0 108.9
79.7 76.7 73.3 69.4 68.7 61.1 54.7 51.4 48.9 37.7 26.7 16.5
170 160 150 140 130 120 110 100 90 80 70 60 50 40 30 20
Chemical Shift (ppm)

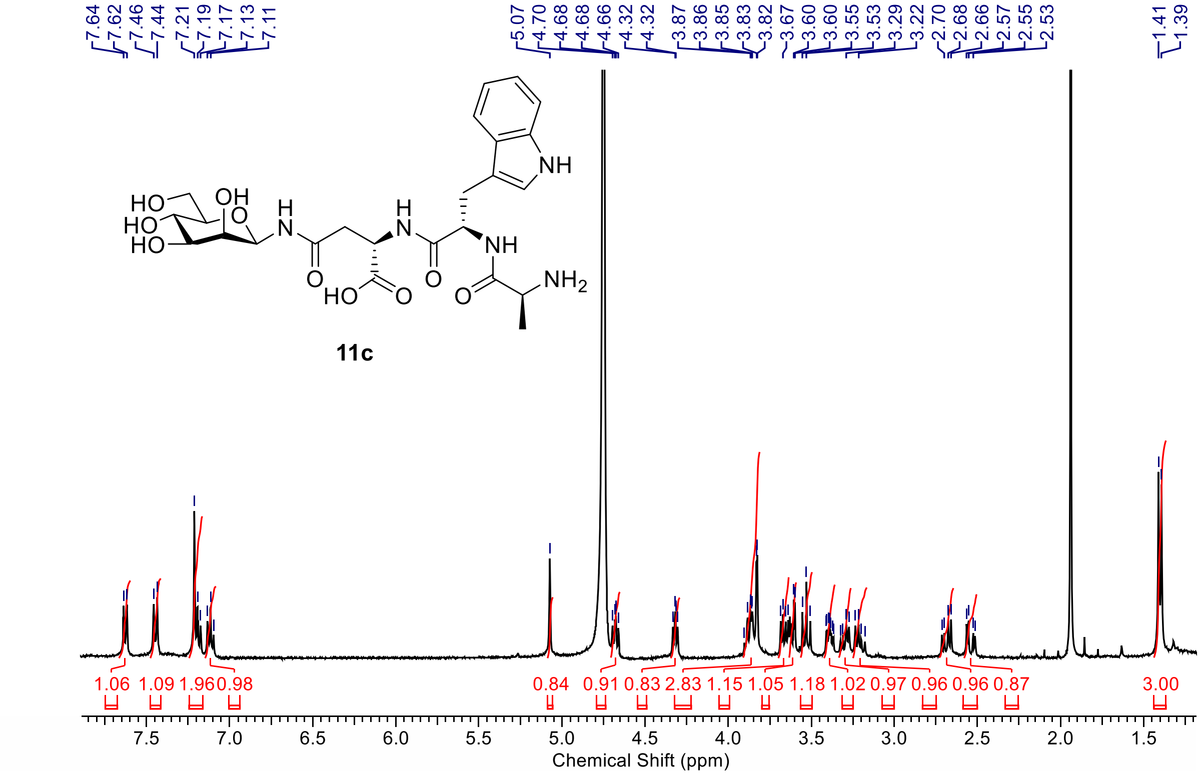
7.64
7.62
7.46
7.44
7.21
7.19
7.17
7.13
7.11
5.07
4.70
4.68
4.68
4.66
4.32
4.32
3.87
3.86
3.85
3.83
3.82
3.67
3.60
3.60
3.55
3.53
3.29
3.22
2.70
2.68
2.66
2.57
2.55
2.53
1.41
1.39
HO
HO
HO
OH
O
H
N
O
H
N
O
HO
O
NH
NH
NH2
O
11c
1.06
1.09
1.96
0.98
0.84
0.91
0.83
2.83
1.15
1.05
1.18
1.02
0.97
0.96
0.96
0.87
3.00
Chemical Shift (ppm)

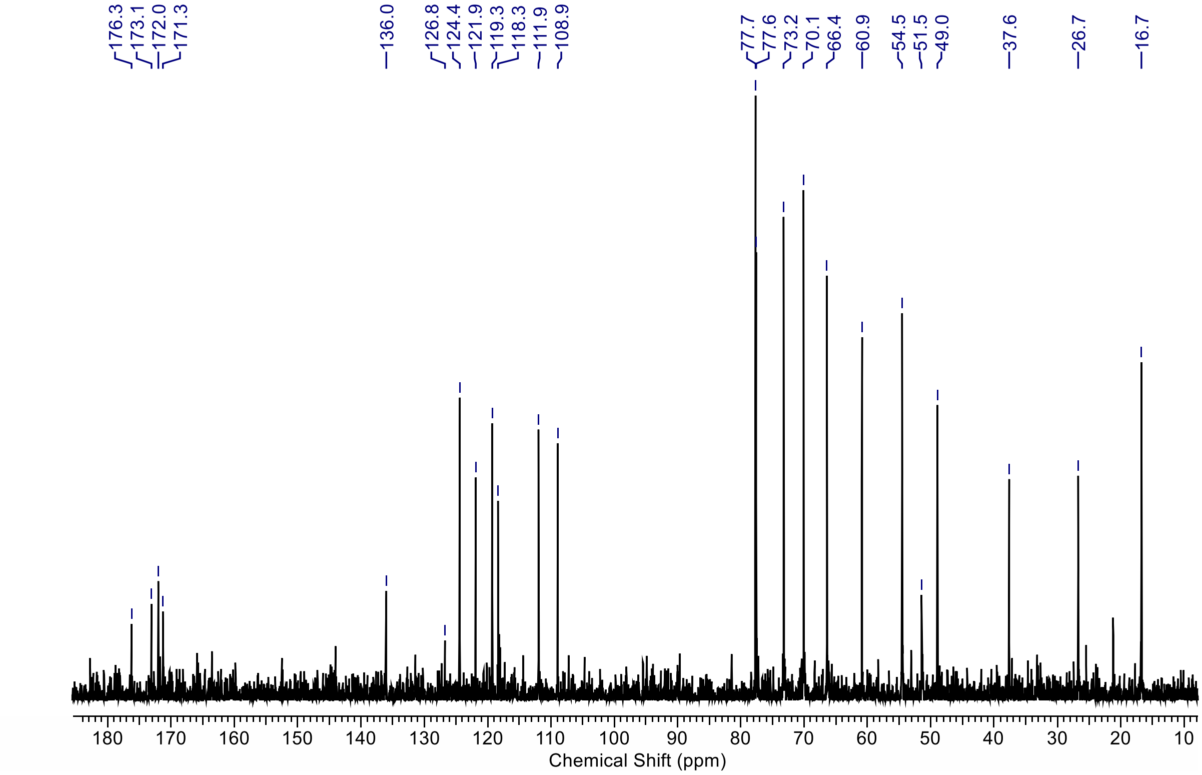
176.3
173.1
172.0
171.3
136.0
126.8
124.4
121.9
119.3
118.3
111.9
108.9
77.7
77.6
73.2
70.1
66.4
60.9
54.5
51.5
49.0
37.6
26.7
16.7
Chemical Shift (ppm)

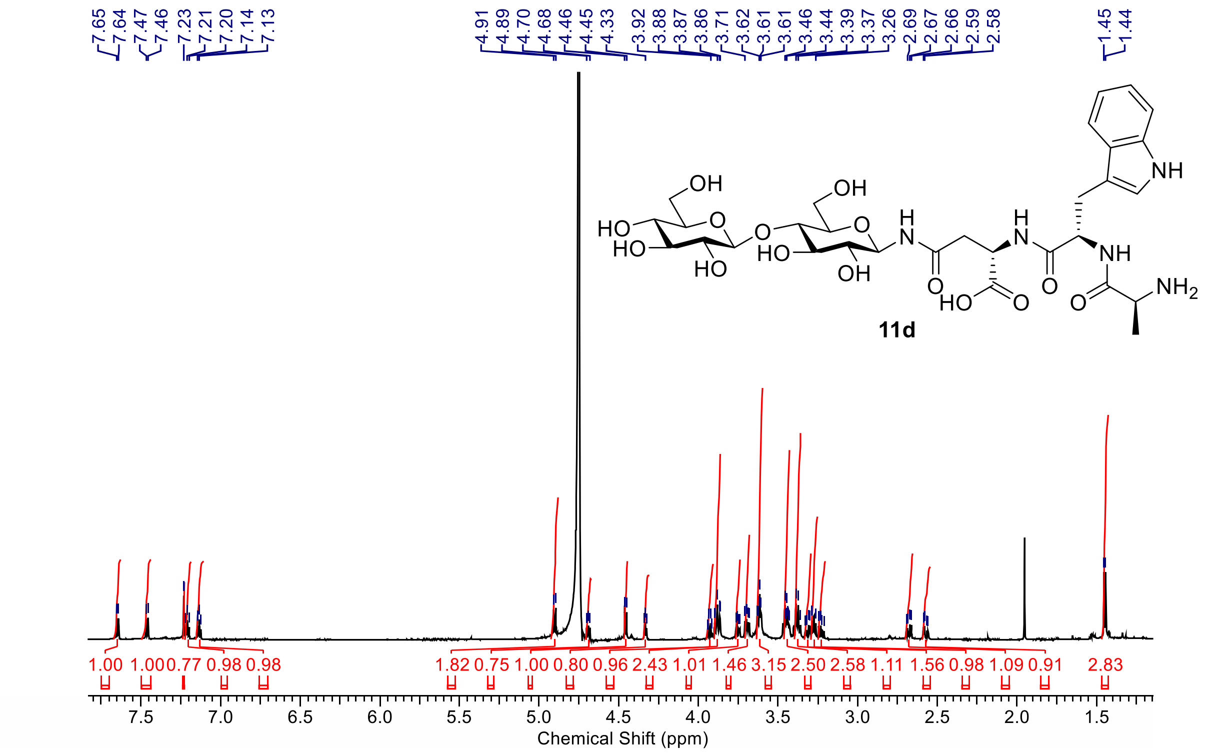
7.65 7.64 7.47 7.46 7.23 7.21 7.20 7.14 7.13
4.91 4.89 4.70 4.68 4.46 4.45 4.33 3.92 3.88 3.87 3.86 3.71 3.62 3.61 3.61 3.46 3.44 3.39 3.37 3.26 2.69 2.67 2.66 2.59 2.58
1.45 1.44
OH
HO
HO
HO
O
O
OH
HO
OH
H
N
O
H
N
HO
O
O
NH
NH
O
NH2
11d
1.00 1.00 0.77 0.98 0.98 1.82 0.75 1.00 0.80 0.96 2.43 1.01 1.46 3.15 2.50 2.58 1.11 1.56 0.98 1.09 0.91 2.83
7.5 7.0 6.5 6.0 5.5 5.0 4.5 4.0 3.5 3.0 2.5 2.0 1.5
Chemical Shift (ppm)

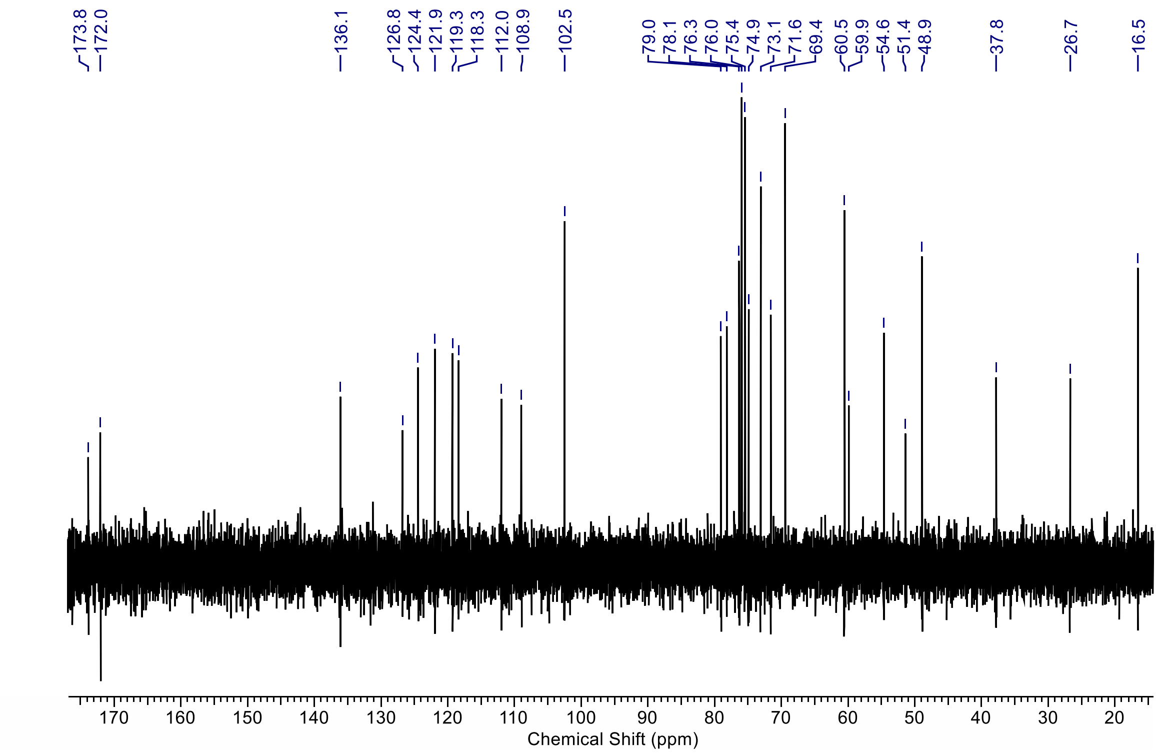
173.8 172.0 136.1 126.8 124.4 121.9 119.3 118.3 112.0 108.9 102.5 79.0 78.1 76.3 76.0 75.4 74.9 73.1 71.6 69.4 60.5 59.9 54.6 51.4 48.9 37.8 26.7 16.5
170 160 150 140 130 120 110 100 90 80 70 60 50 40 30 20
Chemical Shift (ppm)

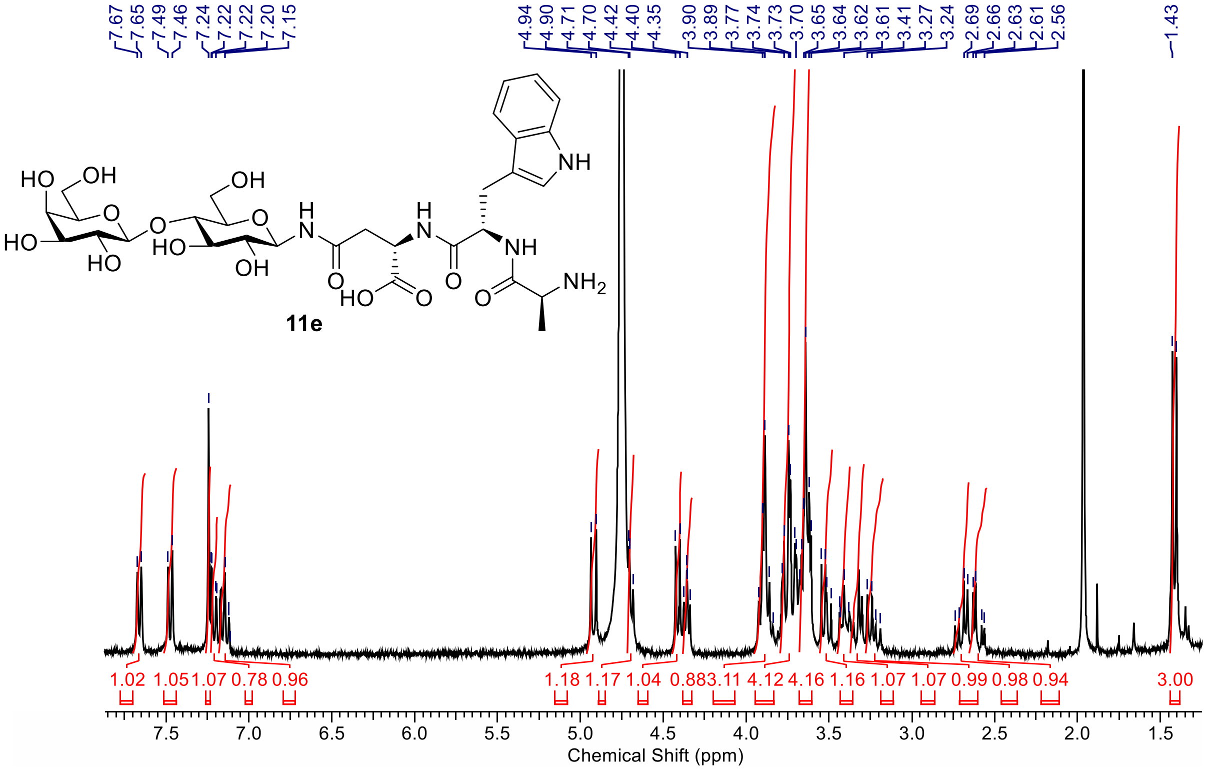
7.67 7.65 7.49 7.46 7.24 7.22 7.22 7.20 7.15
4.94 4.90 4.71 4.70 4.42 4.40 4.35 3.90 3.89 3.77 3.74 3.73 3.70 3.65 3.64 3.62 3.61 3.41 3.27 3.24 2.69 2.66 2.63 2.61 2.56
1.43
HO
OH
OH
O
HO
O
O
HO
HO
OH
H
N
O
H
N
HO
O
O
NH
NH
O
NH2
11e
1.02 1.05 1.07 0.78 0.96 1.18 1.17 1.04 0.88 3.11 4.12 4.16 1.16 1.07 1.07 0.99 0.98 0.94 3.00
7.5 7.0 6.5 6.0 5.5 5.0 4.5 4.0 3.5 3.0 2.5 2.0 1.5
Chemical Shift (ppm)

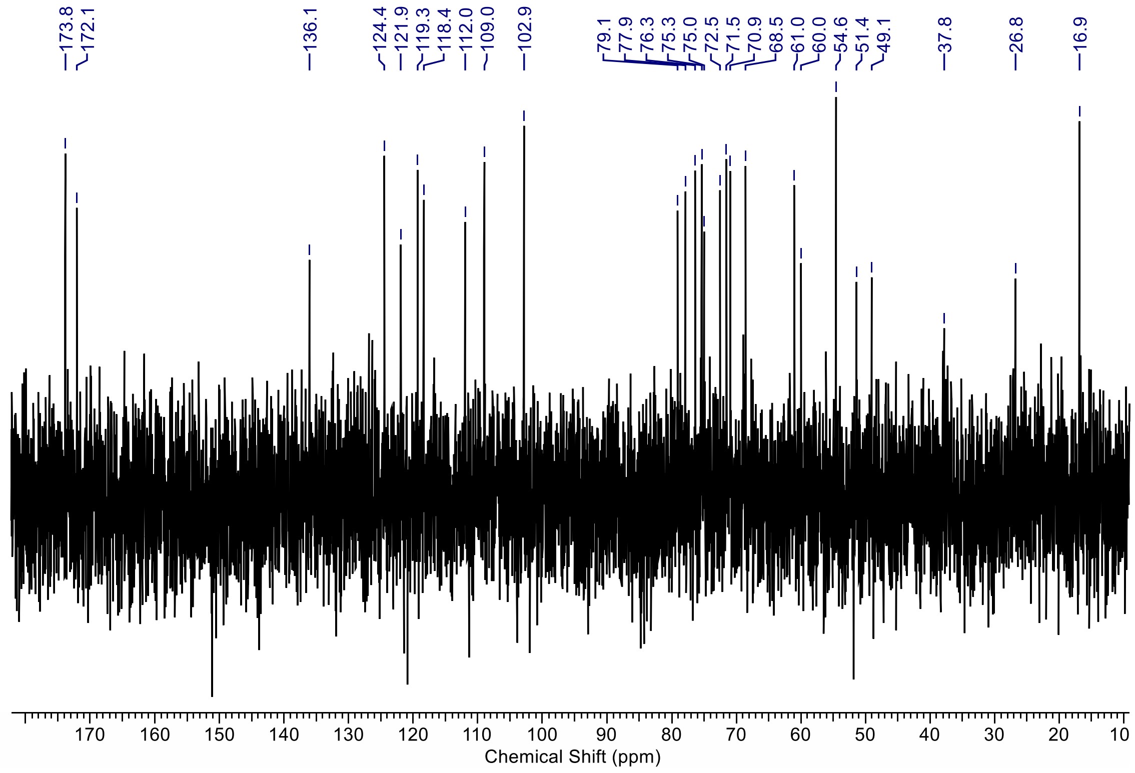
173.8 172.1 136.1 124.4 121.9 119.3 118.4 112.0 109.0 102.9 79.1 77.9 76.3 75.3 75.0 72.5 71.5 70.9 68.5 61.0 60.0 54.6 51.4 49.1 37.8 26.8 16.9
170 160 150 140 130 120 110 100 90 80 70 60 50 40 30 20 10
Chemical Shift (ppm)

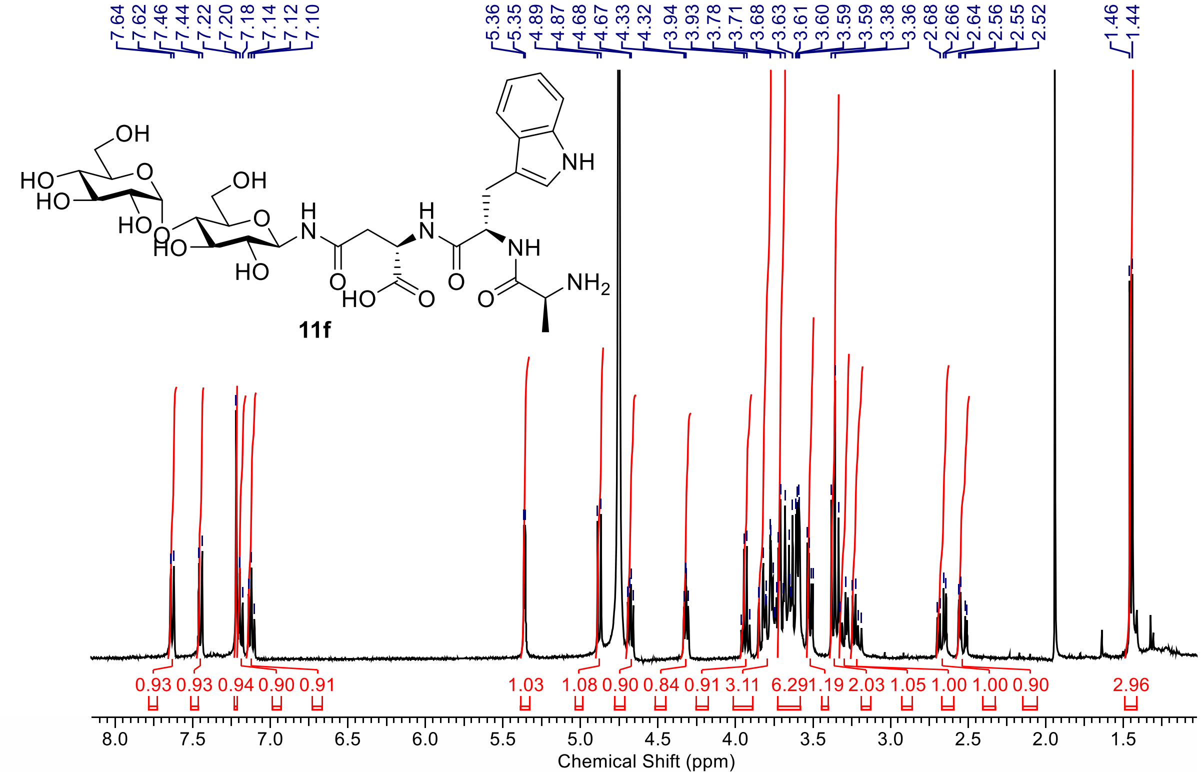
7.64
7.62
7.46
7.44
7.22
7.20
7.18
7.14
7.12
7.10
5.36
5.35
4.89
4.87
4.68
4.67
4.33
4.32
3.94
3.93
3.78
3.71
3.68
3.63
3.61
3.60
3.59
3.59
3.38
3.36
2.68
2.66
2.64
2.56
2.55
2.52
1.46
1.44
OH
HO
HO
HO
O
O
OH
O
HO
HO
H
N
H
N
NH
NH
NH2
O
HO
O
O
O
11f
0.93
0.93
0.94
0.90
0.91
1.03
1.08
0.90
0.84
0.91
3.11
6.29
1.19
2.03
1.05
1.00
1.00
0.90
2.96
Chemical Shift (ppm)

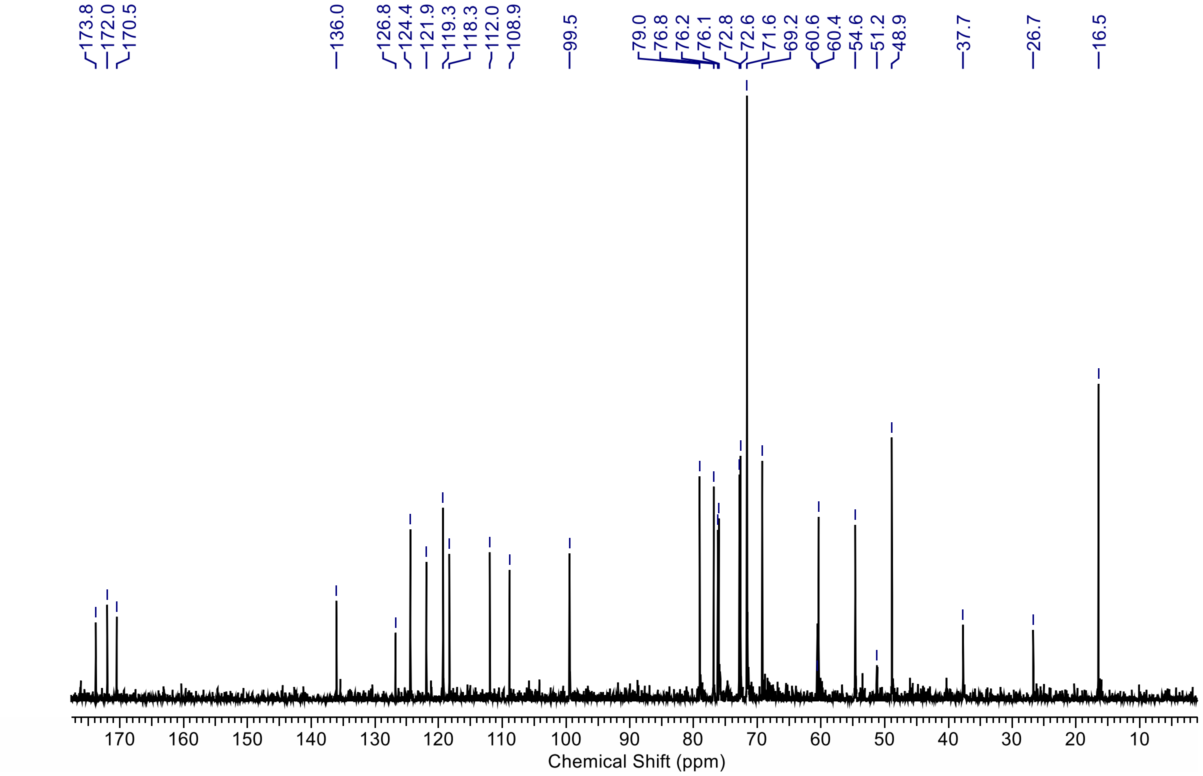
173.8
172.0
170.5
136.0
126.8
124.4
121.9
119.3
118.3
112.0
108.9
99.5
79.0
76.8
76.2
76.1
72.8
72.6
71.6
69.2
60.6
60.4
54.6
51.2
48.9
37.7
26.7
16.5
Chemical Shift (ppm)
